# Durability of protection and immunogenicity of AZD1222 (ChAdOx1 nCoV-19) COVID-19 vaccine over 6 months

**DOI:** 10.1172/JCI160565

**Published:** 2022-09-15

**Authors:** Magdalena E. Sobieszczyk, Jill Maaske, Ann R. Falsey, Stephanie Sproule, Merlin L. Robb, Robert W. Frenck, Hong-Van Tieu, Kenneth H. Mayer, Lawrence Corey, Kathleen M. Neuzil, Tina Tong, Margaret Brewinski Isaacs, Holly Janes, Himanshu Bansal, Lindsay M. Edwards, Justin A. Green, Elizabeth J. Kelly, Kathryn Shoemaker, Therese Takas, Tom White, Prakash Bhuyan, Tonya Villafana, and Ian Hirsch, on behalf of the AstraZeneca AZD1222 Clinical Study Group

**Affiliations:** 1 Division of Infectious Diseases, Department of Medicine, Vagelos College of Physicians and Surgeons, New York–Presbyterian/Columbia University Irving Medical Center, New York, New York, USA.; 2 Clinical Development, Vaccines & Immune Therapies, BioPharmaceuticals R&D, AstraZeneca, Gaithersburg, Maryland, USA.; 3 University of Rochester School of Medicine and Dentistry, Rochester Regional Health, Rochester, New York, USA.; 4 Biometrics, Vaccines & Immune Therapies, BioPharmaceuticals R&D, AstraZeneca, Gaithersburg, Maryland, USA.; 5 Walter Reed Army Institute of Research, Silver Spring, Maryland, USA.; 6 Division of Infectious Diseases, Cincinnati Children’s Hospital Medical Center, Cincinnati, Ohio, USA.; 7 Lindsley F. Kimball Research Institute, New York Blood Center, New York, New York, USA.; 8 Fenway Health, The Fenway Institute and Harvard Medical School, Boston, Massachusetts, USA.; 9 University of Washington, Seattle, Washington, USA.; 10 Fred Hutchinson Cancer Research Center, Seattle, Washington, USA.; 11 University of Maryland School of Medicine, Baltimore, Maryland, USA.; 12 National Institute of Allergy and Infectious Diseases (NIAID), NIH, Bethesda, Maryland, USA.; 13 Data Sciences and AI, BioPharmaceuticals R&D and; 14 Clinical Development, Vaccines & Immune Therapies, BioPharmaceuticals R&D, AstraZeneca, Cambridge, United Kingdom.; 15 Translational Medicine, Vaccines & Immune Therapies, BioPharmaceuticals R&D, AstraZeneca, Gaithersburg, Maryland, USA.; 16 Vaccines & Immune Therapies, BioPharmaceuticals R&D, AstraZeneca, Gaithersburg, Maryland, USA.; 17 Biometrics, Vaccines, & Immune Therapies, BioPharmaceuticals R&D, AstraZeneca, Cambridge, United Kingdom.; 18 The AstraZeneca AZD1222 Clinical Study Group is detailed in Supplemental Acknowledgments.

**Keywords:** COVID-19, Adaptive immunity

## Abstract

**Background:**

We report updated safety, efficacy, and immunogenicity of AZD1222 (ChAdOx1 nCoV-19) from an ongoing phase 3 trial.

**Methods:**

Adults at increased risk of SARS-CoV-2 infection were randomized (2:1), stratified by age, to receive 2 doses of AZD1222 or placebo. The primary efficacy end point was confirmed SARS-CoV-2 reverse-transcriptase PCR–positive (RT-PCR–positive) symptomatic COVID-19 at 15 or more days after a second dose in baseline SARS-CoV-2–seronegative participants. The 21,634 and 10,816 participants were randomized to AZD1222 and placebo, respectively.

**Findings:**

Data cutoff for this analysis was July 30, 2021; median follow-up from second dose was 78 and 71 days for the double-blind period (censoring at unblinding or nonstudy COVID-19 vaccination) and 201 and 82 days for the period to nonstudy COVID-19 vaccination (regardless of unblinding) in the AZD1222 and placebo groups, respectively. For the primary efficacy end point in the double-blind period (141 and 184 events; incidence rates: 39.2 and 118.8 per 1,000 person years), vaccine efficacy was 67.0% (*P* < 0.001). In the period to nonstudy COVID-19 vaccination, incidence of events remained consistently low and stable through 6 months in the AZD1222 group; for the primary efficacy end point (328 and 219 events; incidence rates: 36.4, 108.4) and severe/critical disease (5 and 13 events; incidence rates: 0.6, 6.4), respective vaccine efficacy estimates were 65.1% and 92.1%. AZD1222 elicited humoral immune responses over time, with waning at day 180. No emergent safety issues were seen.

**Conclusion:**

AZD1222 is safe and well tolerated, demonstrating durable protection and immunogenicity with median follow-up (AZD1222 group) of 6 months.

**Trial registration:**

ClinicalTrials.gov NCT04516746.

**Funding:**

AstraZeneca; US government.

## Graphical abstract

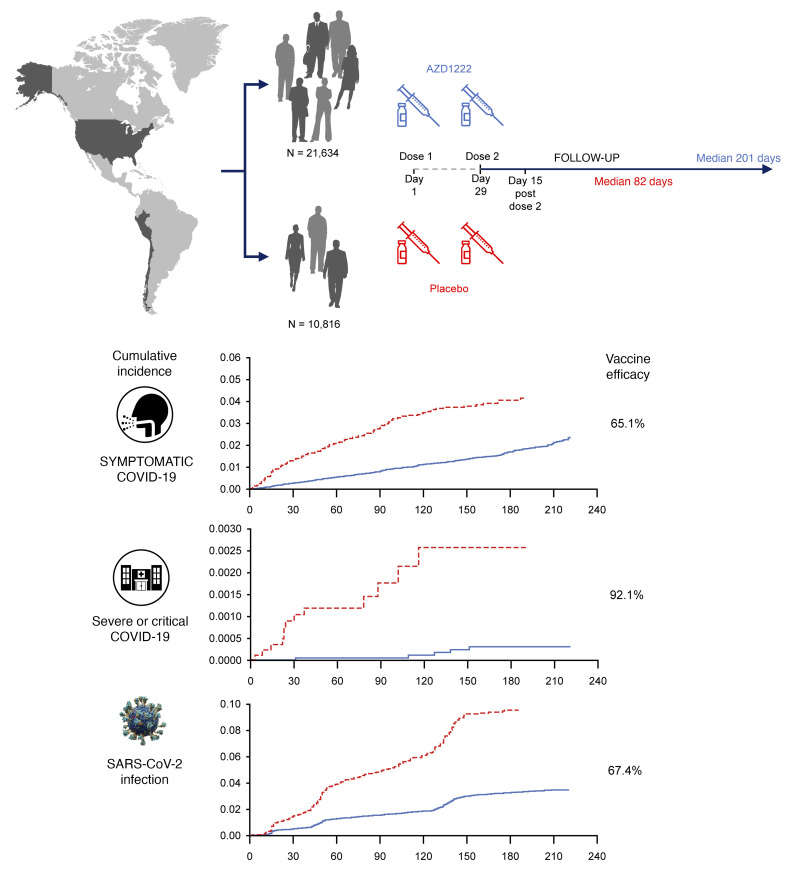

## Introduction

There is an ongoing critical need for vaccination in the fight against the global COVID-19 pandemic. Understanding the durability of vaccine efficacy (VE) and immunogenicity is important in the context of booster dosing in some geographies and populations (1, 2) given evidence of waning immunity with COVID-19 vaccines (2–4). Also of critical importance is understanding the growing prevalence of SARS-CoV-2 variants and the protection conferred by vaccines against severe or critical illness due to these emerging variants (5).

As of February 28, 2022, 28 COVID-19 vaccines had been authorized or approved worldwide (6). AZD1222 (ChAdOx1 nCoV-19) has been distributed to more than 170 countries (7) and is authorized or approved in more than 130 (6), with more than 2 billion doses supplied as of November 16, 2021 (7). The safety, efficacy, and immunogenicity of AZD1222 have been confirmed in an ongoing phase 3, randomized, placebo-controlled trial in the US, Chile, and Peru, in which a diverse population of participants was enrolled from August 2020 to January 2021 (8). Since the trial began, emergency use authorizations (EUAs) and approvals have been granted for 3, 6, and 4 COVID-19 vaccines in the US, Chile, and Peru, respectively (Supplemental Table 1; supplemental material available online with this article; https://doi.org/10.1172/JCI160565DS1) (6), requiring extensive unblinding of trial participants as they have become eligible to receive nonstudy COVID-19 vaccinations.

At the primary analysis, the primary objective was met (8), with VE of 74.0% (95% CI 65.3–80.5; *P* < 0.001) against reverse-transcriptase PCR–positive (RT-PCR–positive) symptomatic COVID-19. VE against severe or critical disease, a secondary end point, was 100% (8). At that data cut-off (March 5, 2021; median follow-up after second dose, 61 days), 35.3% and 38.4% of participants in the AZD1222 and placebo groups, respectively, had been unblinded or received a nonstudy COVID-19 vaccine after receiving their second dose (8). Here, we report the final efficacy analysis from the double-blind phase of the trial, with the majority of participants having been unblinded to treatment assignment. Importantly, we also report analyses for the period up to receipt of nonstudy COVID-19 vaccination, regardless of unblinding, to provide comprehensive safety information and data on durability of protection and immunogenicity of AZD1222 with a median follow-up in the AZD1222 group of 6 months.

## Results

### Participants and follow-up.

**Figure 1:**
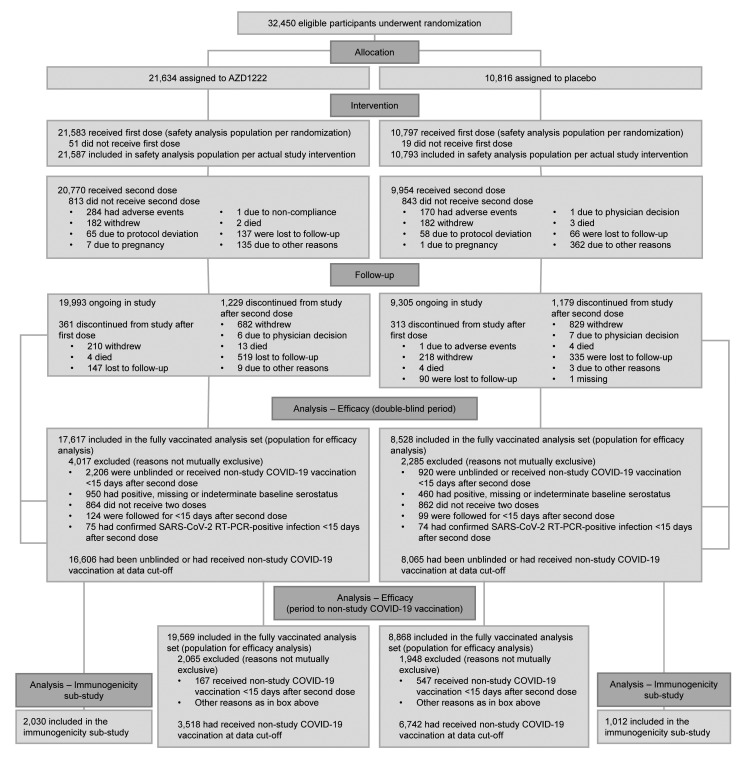
Participant disposition during trial. Overall number randomized and number randomized to AZD1222 are 1 lower than in the primary analysis (8) due to identification of double-counting of 1 participant. In the placebo arm, 1 participant was not included in the primary analysis due to record deactivation, but has been reinstated at this analysis. FVAS population numbers differ from those in the primary analysis because, with additional follow up, additional participants achieved the milestone of 15 days after the second dose and became eligible for these populations, but also some participants were excluded from these populations based on newly obtained information regarding prior infections, baseline serology and receipt of nonstudy COVID-19 vaccinations.

Between August 28, 2020, and January 15, 2021, 32,450 participants were randomized to receive AZD1222 (*n* = 21,634) or placebo (*n* = 10,816) (Figure 1). At the data cutoff (July 30, 2021), there were minimal updates to participant characteristics for the safety population (Supplemental Table 2). Median follow-up in the safety population for the whole study period, regardless of unblinding or receipt of nonstudy COVID-19 vaccination, was 236.0 and 235.0 days after the first dose compared with 92 and 91 days at the primary analysis in the AZD1222 and placebo groups, respectively (Table 1).

### Safety.

As an update to the primary analysis, all participants have now been followed for (or censored due to unblinding/nonstudy COVID-19 vaccination/discontinuation prior to) 28 days after the second dose (day 57) for analysis of nonserious adverse events (AEs). Overall, the majority of AEs were mild or moderate with an additional 81 (0.4%) participants in the AZD1222 group reporting related grade 3 or higher AEs (Supplemental Table 3). The most common unsolicited AEs were consistent with the primary analysis (Supplemental Table 4). No events of thrombosis with thrombocytopenia syndrome (TTS) were reported; thrombocytopenia was reported in 2 (<0.1%) and 0 participants, deep vein thrombosis in 3 (<0.1%) and 1 (<0.1%) participants, thrombosis in 1 (<0.1%) and 0 participants, and pulmonary embolism in 1 (<0.1%) and 0 participants in the AZD1222 and placebo groups, respectively.

For longer-term analysis of serious AEs (SAEs), medically attended AEs (MAAEs), and AEs of special interest (AESIs), median follow-up after the first dose, with censoring for nonstudy COVID-19 vaccination, was 228 and 103 days in the AZD1222 and placebo groups, respectively (Table 1). No emergent or unexpected longer-term safety signals were seen. SAEs were reported in an additional 252 (1.2%) and 68 (0.6%) participants in the AZD1222 and placebo groups, respectively (Supplemental Table 5), with 1 (<0.1%) additional related SAE (AZD1222 group, paresthesia) compared with data reported in the primary analysis (8). Related MAAEs were reported in an additional 22 (0.1%) and 5 (<0.1%) participants in the AZD1222 and placebo groups, respectively, with no previously unreported related MAAEs seen in more than 1 participant (Supplemental Table 6) (8). An additional 5 (<0.1%) and 1 (<0.1%) participants in the AZD1222 and placebo groups, respectively, reported related AESIs, which included 1 additional related potentially immune-mediated condition in each group (AZD1222, vasculitis; placebo, immune thrombocytopenia) (Supplemental Table 7). No related AEs with an outcome of death were reported in either group (Supplemental Table 5).

### Efficacy, double-blind period.

For efficacy analyses in the double-blind period, the fully vaccinated analysis set (FVAS) population comprised 17,617 and 8528 participants in the AZD1222 and placebo groups, respectively (Figure 1). Median follow-up after the second dose, with censoring for unblinding or nonstudy COVID-19 vaccination, was 78 and 71 days, respectively (Table 1). Overall, 16,606 (94.3%) and 8065 (94.6%) of these participants, respectively, had been unblinded or received nonstudy COVID-19 vaccination (Table 1), with similar rates over time in both arms, overall and stratified by age, Occupational Safety and Health Administration (OSHA) risk category (a marker of risk of exposure to COVID-19), and country (data not shown). Participant characteristics in the FVAS population for the double-blind period were consistent with the safety population and balanced between groups (Supplemental Table 8).

**Figure 2:**
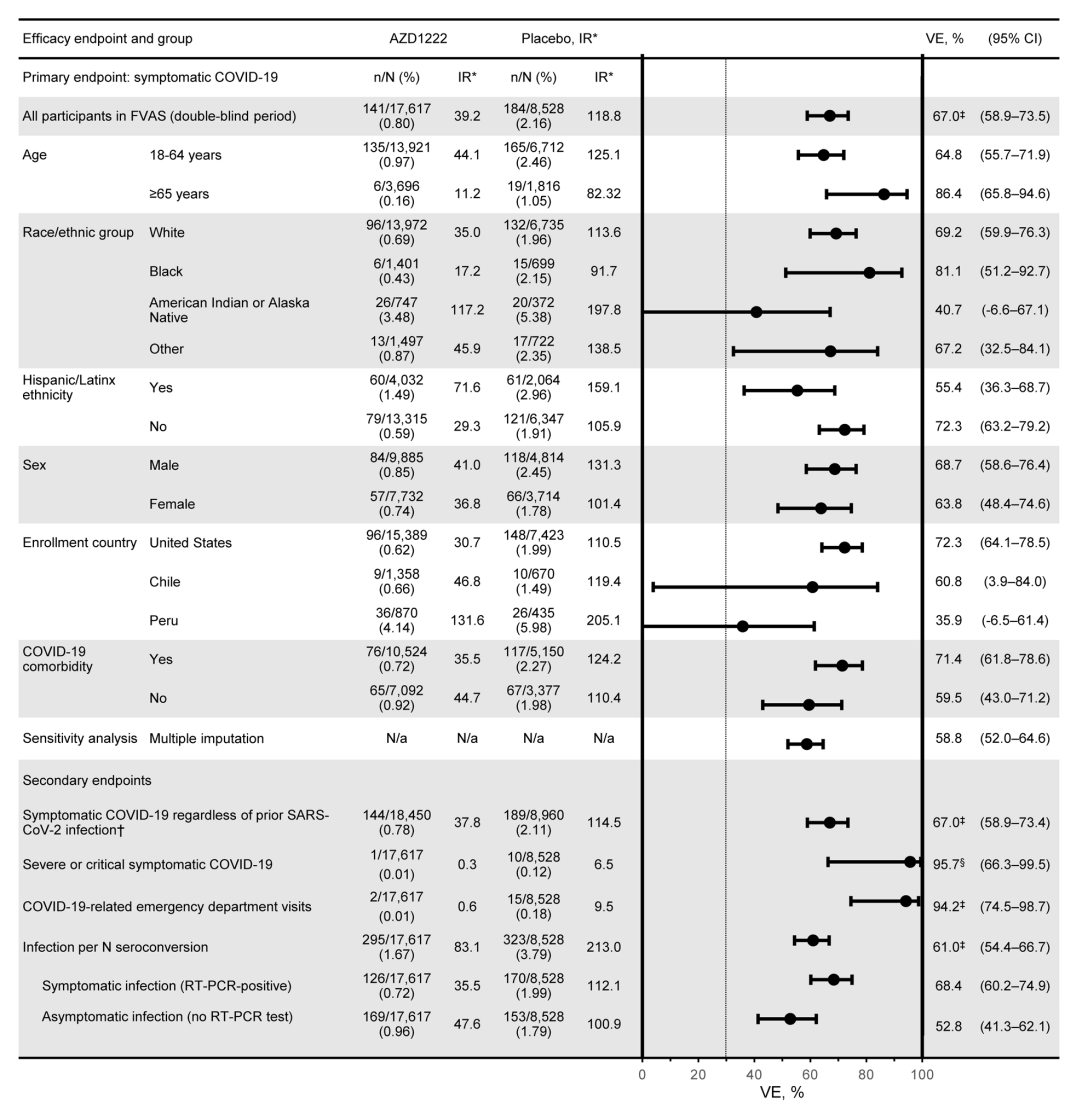
Forest plot of estimated efficacy of AZD1222 versus placebo in the double-blind period. Plot shows estimated AZD1222 efficacy 15 or more days after the second dose for the primary and secondary efficacy end points in the FVAS population for the double-blind period, with censoring for unblinding or nonstudy COVID-19 vaccination (AZD1222, *n* = 17,617; placebo, *n* = 8528). Total follow-up was 3.60 and 1.55 × 1000 person years in the AZD1222 and placebo groups. The dotted vertical line represents the nominally statistically significant criterion of a lower CI greater than 30% applicable to the primary end point and is shown for reference. VE was calculated as (1 minus relative risk) × 100, with relative risk estimated using Poisson’s regression model with robust variance adjusted for follow-up time and with trial group and age group (18–64 versus ≥65 years) as covariates. *Per 1000 person years. †The FVAS includes all participants who were SARS-CoV-2 seronegative at baseline; this population (*n* = 18,450 in AZD1222 group; *n* = 8960 in placebo group) includes participants regardless of prior SARS-CoV-2 infection. ‡*P* < 0.001; ^§^*P* = 0.03. IR, incidence rate.

For the primary efficacy end point in the double-blind period, with 141 and 184 events and estimated incidence rates of 39.2 and 118.8 per 1000 person years overall in the AZD1222 and placebo groups, respectively, VE was 67.0% (95% CI 58.9–73.5; *P* < 0.001) (Supplemental Figure 1A and Supplemental Figure 2). Incidence of events (Supplemental Figure 1B) was consistent and low over time in the AZD1222 group, but varied over time in the placebo group. Estimated incidence rates varied by time period analyzed following the first dose (Supplemental Table 9). Estimates of incidence rates beyond the 24-week time point become unreliable due to increasing attrition of the at-risk population as a result of censoring for unblinding or nonstudy COVID-19 vaccination. For the secondary efficacy end point of severe or critical symptomatic COVID-19, with 1 and 10 events, respectively, VE was 95.7% (95% CI 66.3–99.5; *P* < 0.001) (Supplemental Figure 1C and Figure 2). AZD1222 efficacy against acquisition of SARS-CoV-2 infection, as defined by rate of SARS-CoV-2 nucleocapsid antibody seroconversion from negative at baseline to positive at 15 days or more after the second dose, regardless of symptoms, was 61.0% (95% CI 54.4–66.7; *P* < 0.001) (Supplemental Figure 1D and Figure 2).

### Efficacy, period to nonstudy COVID-19 vaccination.

For efficacy analyses in the period to nonstudy COVID-19 vaccination, the FVAS population comprised 19,569 and 8868 participants in the AZD1222 and placebo groups, respectively (Figure 1). Participant characteristics were generally consistent with the safety population and balanced between groups (Supplemental Table 10). Median follow-up after the second dose, with censoring at nonstudy COVID-19 vaccination only, was 201.0 and 82.0 days in the AZD1222 and placebo groups, respectively (Table 1). Overall, 3518 (18.0%) and 6742 (76.0%) participants in the AZD1222 and placebo groups, respectively, had received nonstudy COVID-19 vaccination (Table 1). Kaplan-Meier analyses of nonstudy COVID-19 vaccination over time showed differential censoring according to age (with older participants unblinded earlier due to earlier eligibility for nonstudy COVID-19 vaccination), OSHA risk category (a marker of risk of exposure to COVID-19), and country (Supplemental Figure 2).

**Figure 3:**
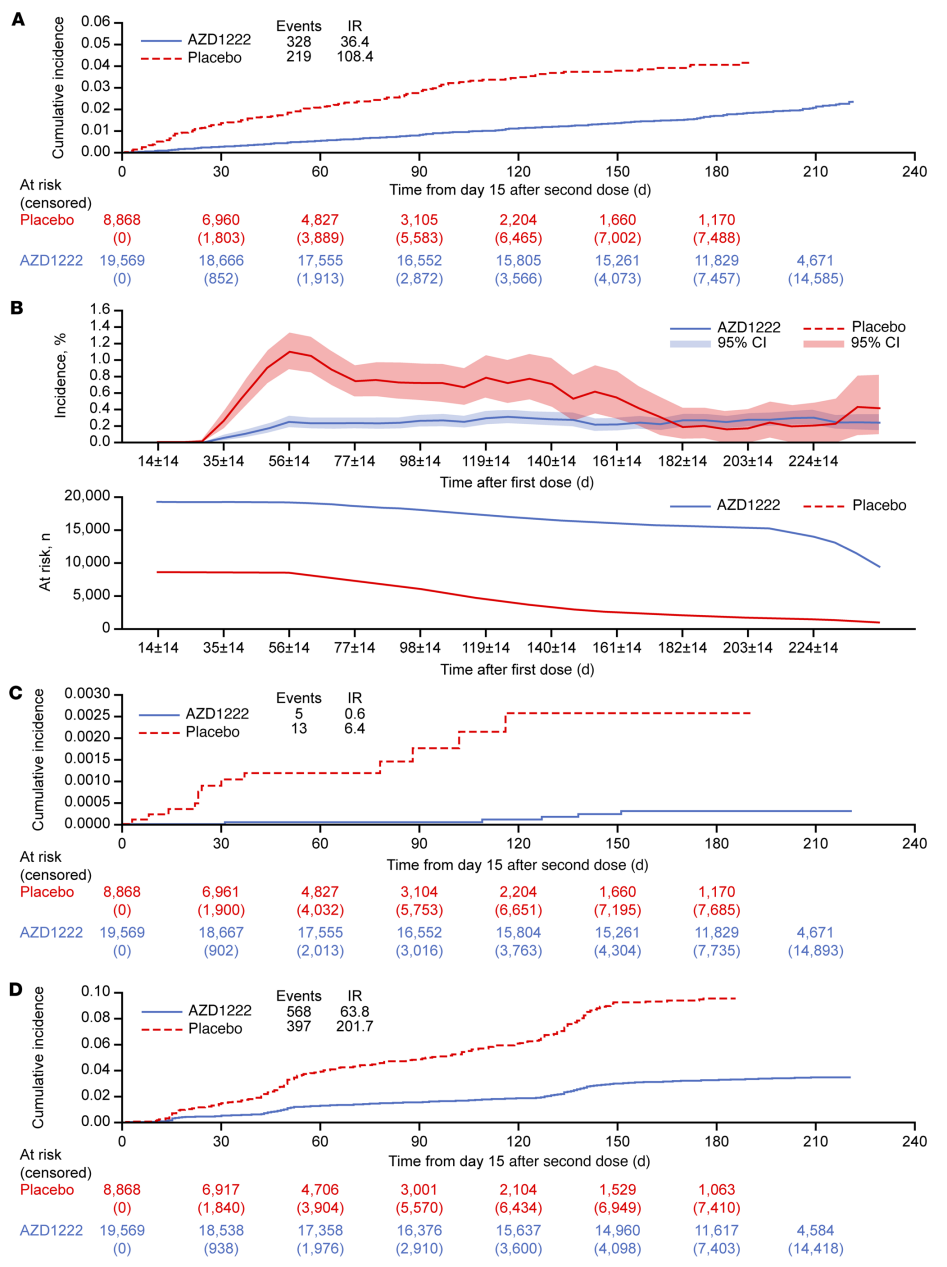
Estimated efficacy of AZD1222 versus placebo for the prevention of COVID-19 and SARS-CoV-2 infection during the period to nonstudy COVID-19 vaccination. (**A**) Cumulative incidence of SARS-CoV-2 RT-PCR–positive symptomatic illness occurring 15 or more days after the second dose (time 0 = day 15 after the second dose) in the FVAS population for the period to nonstudy COVID-19 vaccination (AZD1222, *n* = 19,569; placebo, *n* = 8868), with censoring for nonstudy COVID-19 vaccination, regardless of unblinding. (**B**) Incidence of SARS-CoV-2 RT-PCR–positive symptomatic illness events and decrease in the at-risk population over time from first dose during the period to nonstudy COVID-19 vaccination. The at-risk population curves show the numbers of participants in the FVAS who have not been censored and were available for analysis at the corresponding time point. Cumulative incidence of (**C**) severe or critical symptomatic COVID-19 and (**D**) SARS-CoV-2 infection, as defined by seroconversion rate from negative at baseline to positive for SARS-CoV-2 nucleocapsid antibody at 15 or more days after the second dose, regardless of symptoms, in the FVAS population for the period to nonstudy COVID-19 vaccination (AZD1222, *n* = 19,569; placebo, *n* = 8868), with censoring for nonstudy COVID-19 vaccination, regardless of unblinding. For panels **A**, **C**, and **D**, time to first event was from time of second dose administration, calculated as follows: (date of SARS-CoV-2-positive test) – (date of second dose of AZD1222 or placebo + 14 days) + 1. For censored participants, censoring time was from date of second dose of AZD1222 or placebo plus 14 days to the last time observed before data cutoff (July 30, 2021). Cumulative incidences were estimated using the Kaplan-Meier method. Cumulative incidence curves were truncated at the point at which less than 10% of the starting population remained at risk. IR, incidence rate per 1000 person years.

**Figure 4:**
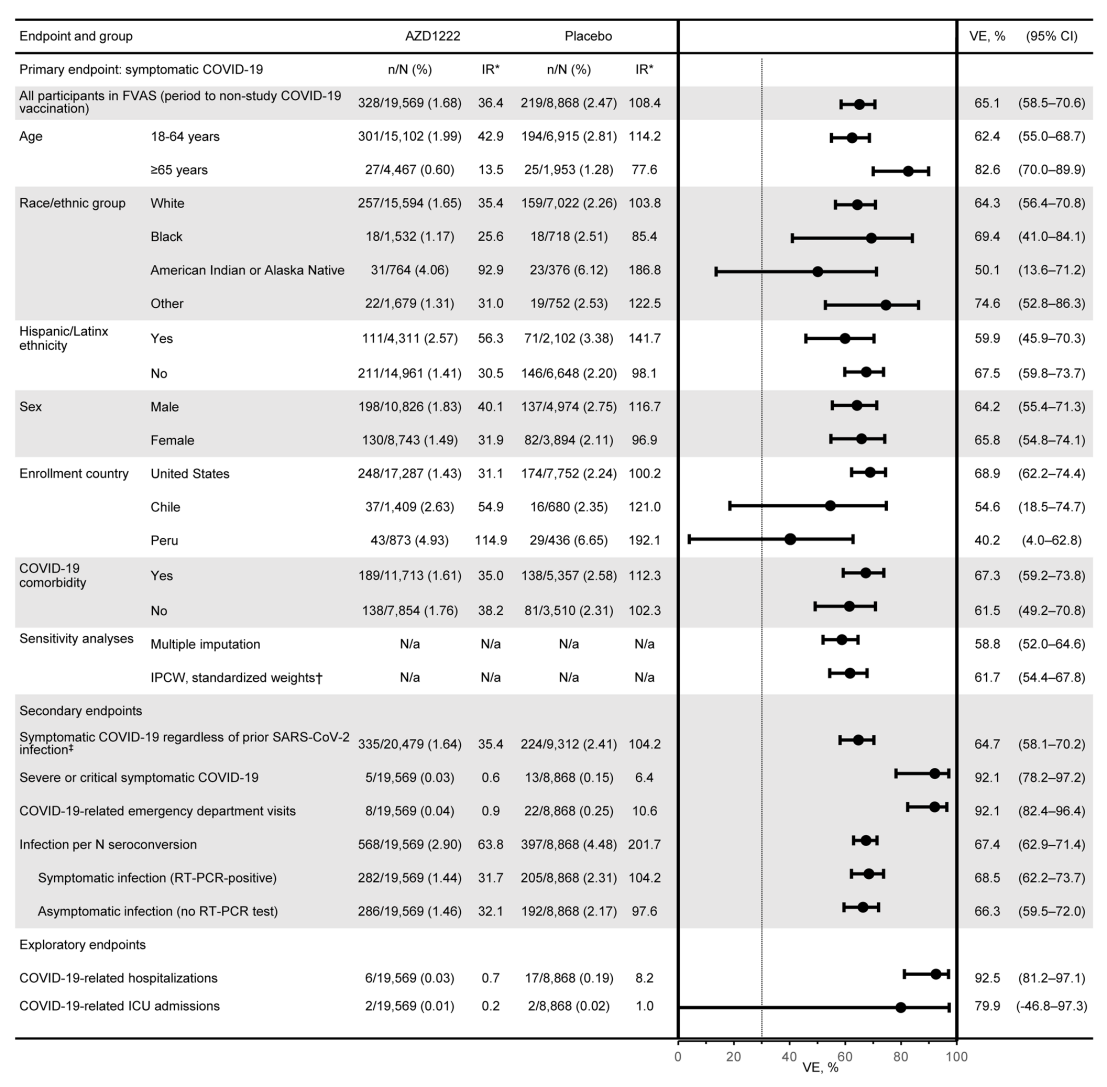
Forest plot of efficacy estimates of AZD1222 versus placebo in the period up to receipt of nonstudy COVID-19 vaccination. Plot shows estimated AZD1222 efficacy 15 or more days after the second dose for the primary, secondary, and exploratory efficacy end points in the FVAS population for the period to nonstudy COVID-19 vaccination, with censoring for nonstudy COVID-19 vaccination, regardless of unblinding (AZD1222, *n* = 19,569; placebo, *n* = 8868). Total follow-up was 9.01 and 2.02 × 1000 person years in the AZD1222 and placebo groups. The dotted vertical line represents the nominally statistically significant criterion of a lower CI greater than 30% applicable to the primary end point and is shown for reference. VE was calculated as (1 minus relative risk) × 100, with relative risk estimated using Poisson’s regression model with robust variance adjusted for follow-up time and with trial group and age group (18–64 versus ≥65 years) as covariates. *Per 1000 person-years. †Results from an IPCW method applied to right censoring and using standardized weights. See *Statistics* section of Methods for methodology and Supplemental Table 11 for additional analyses and information. ‡The FVAS includes all participants who were SARS-CoV-2 seronegative at baseline; this population (*n* = 20,479 in AZD1222 group; *n* = 9312 in placebo group) includes participants regardless of prior SARS-CoV-2 infection.

For the primary efficacy end point in the period to nonstudy COVID-19 vaccination, with 328 and 219 events and estimated incidence rates of 36.4 and 108.4 per 1000 person years overall in the AZD1222 and placebo groups, respectively, estimated efficacy was 65.1% (95% CI 58.5–70.6) (Figure 3A and Figure 4). Durable protection against symptomatic COVID-19 was seen, with a consistent low incidence of events over time in the AZD1222 group (Figure 3B). Incidence of events varied substantially over time in the placebo group (Figure 3B). The primary efficacy end point was also evaluated for the period from 15 days after the second dose to less than 6 months after the first dose. Estimated AZD1222 efficacy for this period was 70.2% (95% CI 63.9–75.4) (Supplemental Table 9). This analysis excluded data from 6 or more months after the first dose for 15,514 and 1896 participants in the AZD1222 and placebo groups, respectively, who had follow-up of 6 or more months. Of these participants, 110 and 14, respectively, had symptomatic COVID-19 events occurring 6 or more months after the first dose. Estimates of incidence rates beyond 24 weeks became unreliable due to attrition of participants in the placebo group and accumulating bias over the whole follow-up period resulting from nonrandom censoring. In sensitivity analyses for handling of missing data in the primary efficacy end point analysis for the period up to nonstudy COVID-19 vaccination, estimated AZD1222 efficacy was 58.8% (95% CI 52.0–64.6) using multiple imputation with age group as a risk covariate, and 61.7% (95% CI 54.4–67.8) using an inverse-probability-of-censoring weighting (IPCW) analysis (Figure 4 and Supplemental Table 11), supporting the finding that durability of protection is seen over the observed follow-up period.

For the secondary efficacy end point of severe or critical symptomatic COVID-19, with 5 and 13 cases and estimated incidence rates of 0.6 and 6.4 per 1000 person years in the AZD1222 and placebo groups, respectively, estimated efficacy for the period up to nonstudy COVID-19 vaccination was 92.1% (95% CI 78.2–97.2) (Figure 3C and Figure 4). Protection was similarly high against COVID-19–related hospitalization (92.5%; 95% CI 81.2–97.1), emergency department visits (92.1%; 95% CI 82.4–96.4), and intensive care unit (ICU) admissions (79.9%; 95% CI -46.8–97.3; Figure 4; numbers of ICU admissions were small, resulting in wide CIs). Estimated AZD1222 efficacy against acquisition of SARS-CoV-2 infection in the period up to nonstudy COVID-19 vaccination was 67.4% (95% CI 62.9–71.4) overall (Figure 3D and Figure 4) and 68.5% (95% CI 62.2–73.7) and 66.3% (95% CI 59.5–72.0) against symptomatic and asymptomatic infections, respectively (Figure 4).

### Nonstudy COVID-19 vaccinations.

In the immunogenicity substudy, neutralizing antibody and spike-binding antibody responses were seen in placebo participants at day 180 (Supplemental Figure 3), despite censoring at unblinding, reported nonstudy COVID-19 vaccination, or SARS-CoV-2 infection. A proportion of placebo participants were found to have a 4-fold or greater increase from baseline in spike-binding antibodies in the absence of a 4-fold or greater increase from baseline for SARS-CoV-2 nucleocapsid antibodies and in the absence of a reported nonstudy COVID-19 vaccination. Across all postbaseline visits, the incidence of such increases was 6.7% (64/957), which included 30 of 139 (21.6%) of evaluable participants on day 180.

### Immunogenicity.

**Figure 5:**
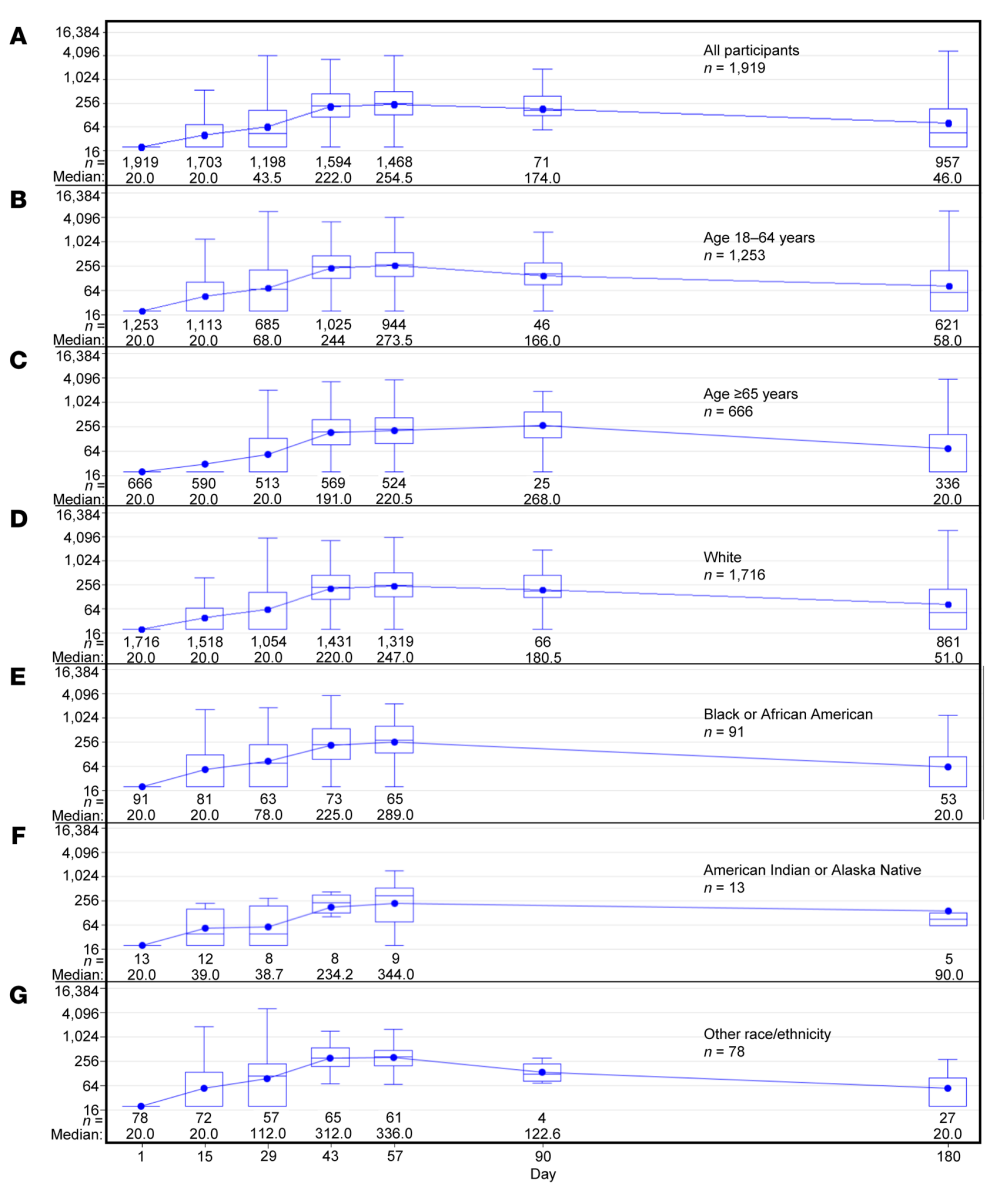
Neutralizing antibody responses over time in the AZD1222 group in the immunogenicity substudy population. Box and whisker plots showing SARS-CoV-2 neutralizing antibody quantitation over time, (**A**) overall, and by (**B** and **C**) age and (**D**–**G**) race/ethnicity (data on race/ethnicity missing for *n* = 21 participants). Participants were censored at the earliest date of nonstudy COVID-19 vaccination, positive test for SARS-CoV-2 nucleocapsid antibodies, RT-PCR–positive SARS-CoV-2 symptomatic infection, or last trial contact. The *y* axes shows 1/dilution; for conversion to the WHO International Standard, see Supplemental Methods. Box indicates interquartile range, whiskers indicate range, horizontal line in box indicates median, and dot in box indicates mean.

AZD1222 elicited humoral immune responses over time, as demonstrated by neutralizing antibody titers (Figure 5A), with some waning observed at day 180. This pattern was consistent regardless of age (Figure 5, B and C), race (Figure 5, D–G), or the presence of comorbidities at baseline (Supplemental Figure 4). In participants who were SARS-CoV-2–seropositive at baseline, humoral responses increased after the first dose, with minimal increases after the second dose. Waning was observed at day 180, but responses remained higher compared with those in participants who were seronegative at baseline (Supplemental Figure 4). Analyses of the correlation between antivector immune responses and induction of neutralizing antibodies showed very weak or no correlation at all time points (Supplemental Figure 5).

### SARS-CoV-2 genotypic evaluation.

**Figure 6:**
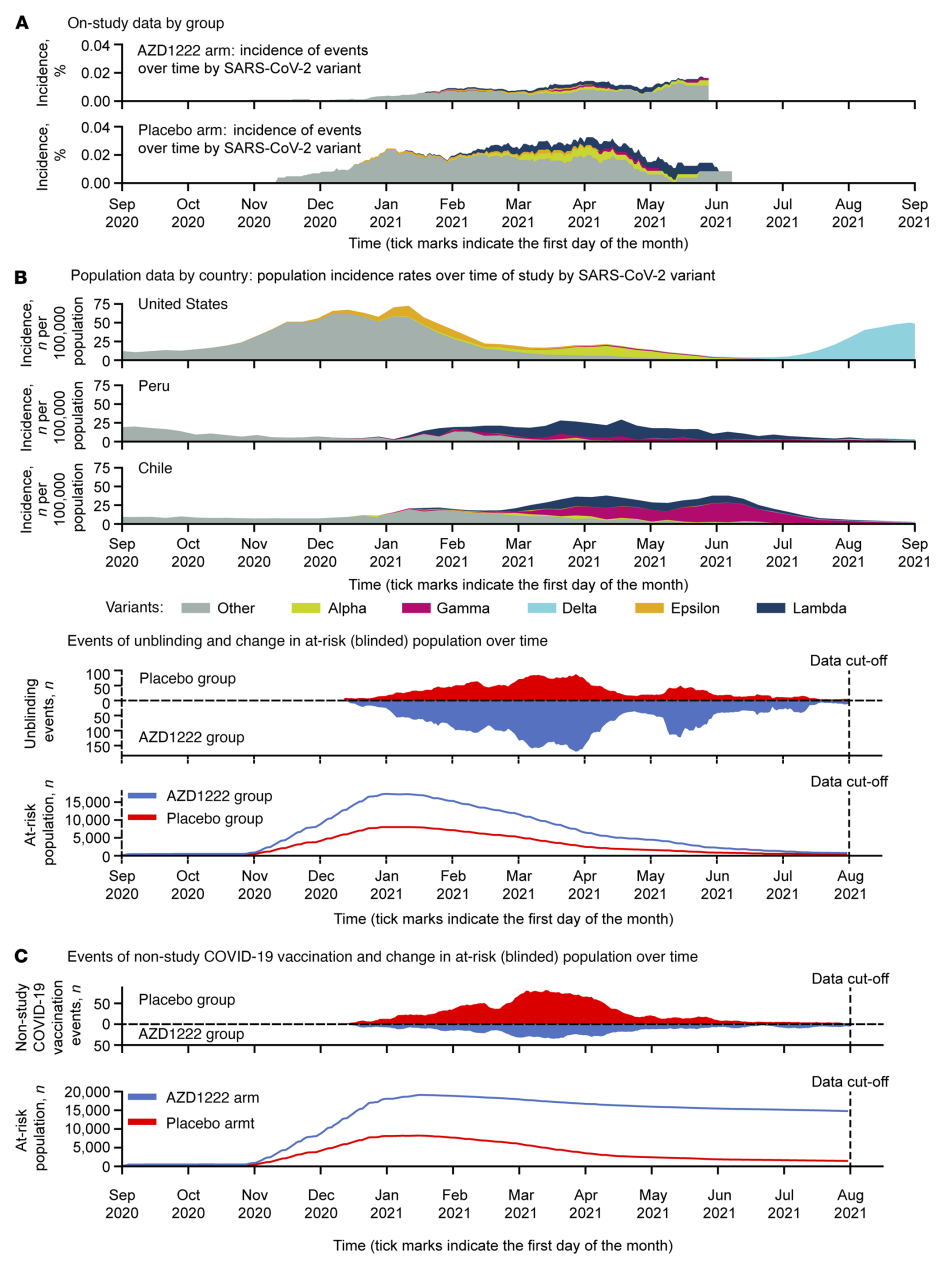
Incidence of SARS-CoV-2 variants, numbers at risk, and unblinding/receipt of nonstudy COVID-19 vaccination over the time course of the trial. (**A**) Incidence of variants observed in cases of RT-PCR–confirmed SARS-CoV-2 infection in the placebo and AZD1222 arms of the trial, truncated at the point at which less than 10% of the starting population remained at risk, and incidence of confirmed cases in population data from the US, Peru, and Chile during the time of study (data derived from COVID-19 Data Repository by the Center for Systems Science and Engineering [CSSE] at Johns Hopkins University, ref. 30, available at https://github.com/owid/covid-19-data/tree/71b0337018fe20d469aa9014e3a8003d900a2b5b, commit ID: 71b0337018fe20d469aa9014e3a8003d900a2b5b; and from GISAID, EPI_SET_220825fk, https://doi.org/10.55876/gis8.220825fk), along with timing of participants being on study, with censoring for (**B**) unblinding or nonstudy COVID-19 vaccination, or (**C**) nonstudy COVID-19 vaccination only in the FVAS populations for these 2 analyses.

Nasopharyngeal swab (*n* = 198) and/or saliva (*n* = 187) samples for SARS-CoV-2 genotypic evaluation were available from 250 of 325 adjudicated cases of SARS-CoV-2 RT-PCR–positive symptomatic illness in the double-blind period. Spike-specific next-generation sequencing (NGS) of nasopharyngeal swabs was reported for 81 and 115 cases in the AZD1222 and placebo groups, respectively (sequencing attempted, but quantity not sufficient in 2 cases; Supplemental Table 12). The most common variants identified were the Lambda variant of interest (VoI) in 17 (0.10%) and 18 (0.21%) participants in the AZD1222 and placebo groups, respectively, and the Alpha variant of concern (VoC) in 9 (0.05%) and 11 (0.13%) participants. VE against symptomatic COVID-19 (per the primary efficacy end point) for all variants identified was 69.7% (95% CI 59.7–77.2); efficacy was similar against the Alpha VoC and appeared slightly lower against specific VoIs and slightly higher against other variants with A_1 lineage (Supplemental Table 12). Variants identified in the trial were consistent with the primary circulating SARS-CoV-2 strains in the US, Chile, and Peru over time (Figure 6). Only 2 cases of Delta were identified in the analysis of the double-blind period, and Omicron had not been identified at the time of database lock.

## Discussion

This updated analysis demonstrates the continued safety, immunogenicity, and efficacy of AZD1222 for the prevention of symptomatic COVID-19 and severe or critical illness, with VE of 67.0% and 95.7%, respectively, in the double-blind period. Consistently low incidence of COVID-19 and severe or critical disease was seen for 6 months after the first dose in the AZD1222 group, with estimated efficacy of 65.1% and 92.1%, respectively, for the period up to nonstudy COVID-19 vaccination. Durable protection against SARS-CoV-2 infection was also seen, including substantial protection against asymptomatic infection. These findings are supported by data showing humoral immunogenicity, including in older participants, with 6-month immunogenicity data indicating the expected initial waning of humoral responses.

The durability of protection with AZD1222 through to 6 months, with an efficacy estimate of 70.2% for the period from 15 days after the second dose up to 6 months after the first dose, is consistent with findings after shorter follow-up from other trials (9) and real-world analyses (10) of AZD1222. Durable efficacy has also been demonstrated with BNT162b2 and mRNA-1273 through 6 months and more than 5 months, respectively (11, 12), as well as with Ad26.COV2.S (13), with a gradual decline in efficacy over time (12–15). Importantly, however, as shown in the present study, efficacy against severe or critical COVID-19 has been maintained (11–13). These findings may be valuable in determining optimal intervals for vaccine booster dosing.

Our data demonstrating the durability of immunogenicity with AZD1222 are similar to findings with other COVID-19 vaccines over a similar duration of follow-up (2, 3, 16–18). Analyses in small populations have shown antibody persistence through 6 months after the second dose with mRNA-1273 (17) and durable humoral and cellular responses, with limited decreases through 8 months after vaccination with Ad26.COV2.S (18). In contrast, a larger longitudinal study in Israel showed more substantial decreases in neutralizing antibody titers and IgG antibody levels at 6 months after the second dose of BNT162b2 (16). While correlates of protection against SARS-CoV-2 infection have yet to be determined, higher immune responses are associated with improved protection (19, 20). In our trial, antibody titers at 6 months after the first dose of AZD1222 remained similar to those seen in the immediate post-first-dose responses, and a consistent pattern of durable immunogenicity was demonstrated in subgroups according to age, race/ethnicity, and presence of comorbidities. Lower absolute titers were observed in older adults, but the difference at the time of analysis did not appear clinically meaningful in the context of AZD1222 VE findings by age subgroup (Figure 4).

The primary limitation of this updated analysis is the relatively short additional follow-up time for the double-blind period. For ethical reasons, there was extensive unblinding of participants after December 2020 associated with other COVID-19 vaccines becoming available through EUAs in the US, Chile, and Peru (Supplemental Table 1) (6). This exemplifies the broader challenge of conducting randomized, placebo-controlled trials while managing participant well-being during a pandemic (15, 21). To overcome this, we analyzed efficacy with participants censored only at the time of nonstudy COVID-19 vaccination, regardless of unblinding, allowing for a longer period of evaluation in participants who did not immediately receive a nonstudy COVID-19 vaccine. As would be expected, the latter occurred predominantly in the AZD1222-vaccinated group, resulting in substantially greater total follow-up in the AZD1222 group and greater attrition of the at-risk population in the placebo group. Notably, the composition of the ongoing populations in the study arms was altered due to nonrandom censoring associated with prioritized vaccine rollout to high-risk groups (e.g., healthcare workers, the elderly; Supplemental Figure 2).

This informative censoring associated with COVID-19 risk factors may potentially introduce bias for comparisons between groups (22), and so sensitivity analyses were conducted to explore these potential biases and provide supportive findings. Our multiple imputation analyses adjusting for single baseline covariates gave efficacy estimates of 58.8% to 59.5%. Our IPCW analysis, which was designed to overcome accumulating bias resulting from informative censoring, resulted in an efficacy estimate of 61.7% (Supplemental Table 11). An additional consideration is that participant unblinding may have given rise to behavioral changes prior to subsequent nonstudy COVID-19 vaccination, with unvaccinated participants potentially more likely to maintain more cautious behavior (22). Nevertheless, data from our analyses of the period to nonstudy COVID-19 vaccination were consistent with other efficacy and effectiveness data for AZD1222 (9, 10), supporting the value of these findings.

Another potential limitation is a possible confounding effect of underreporting of nonstudy COVID-19 vaccination in both arms, despite our extensive efforts to capture this information. While approximately 95% of participants in each group had been unblinded, only approximately three-quarters of participants in the placebo group were documented to have received nonstudy COVID-19 vaccination at the time of database lock. Unreported nonstudy COVID-19 vaccination might explain the enrichment of neutralizing antibody and spike-binding antibody responses seen in placebo participants at day 180 (Supplemental Figure 3), despite censoring for SARS-CoV-2 infection or known receipt of nonstudy COVID-19 vaccination. We found that 21.6% of evaluable placebo participants in the immunogenicity substudy had a 4-fold or greater increase from baseline in spike-binding antibodies, in the absence of a positive test for SARS-CoV-2 nucleocapsid antibodies and with no reported nonstudy COVID-19 vaccination, on day 180. Data are not available to extrapolate this finding to the overall placebo FVAS population. Nevertheless, it suggests a possible explanation for the reduction in the incidence of symptomatic COVID-19 in the placebo group after approximately 20 weeks following the first dose (Figure 3B and Supplemental Figure 1B), which attenuated efficacy estimates in this analysis.

The final efficacy data from the double-blind period of this phase 3 trial were consistent with the primary analysis (8), and findings for the primary efficacy end point across subgroups and against the Alpha VoC were also generally consistent (8). These analyses were conducted largely prior to the Delta variant surge in the US in July/August 2021 (Figure 6) as well as the more recent emergence of the Omicron variant, against which lower live virus neutralization titers compared with the early Victoria strain of SARS-CoV-2 have been reported following 2 doses of AZD1222 or BNT162b2 (23). At the time of enrollment, the original SARS-CoV-2 strain was predominant in all trial regions. During the course of follow-up, differing VoCs/VoIs emerged across countries (5), but study populations in Chile and Peru, where variants such as Gamma and Lambda were more prevalent, were comparatively small. Other real-world data support the effectiveness of AZD1222 against the Delta variant (24–26).

No new or emergent safety issues were seen at this updated analysis. Safety findings were consistent with the known safety profile of AZD1222. Overall, AZD1222 remains well tolerated up to 6 months after vaccination and through to nonstudy COVID-19 vaccination. A number of additional reports of TTS following AZD1222 vaccination have been published since the primary analysis of this trial (27); however, no cases of TTS were seen in this follow-up analysis, as expected given the extremely rare nature of these events and their occurrence primarily following the first dose of AZD1222 (27, 28). Vaccine hesitancy remains a problem worldwide, with concerns about the speed of development and lack of long-term follow-up from vaccine trials. These data therefore provide reassurance that no safety signals have emerged from this carefully monitored trial population.

In conclusion, AZD1222 is one of the most widely used COVID-19 vaccines globally, including in many resource-limited regions, and these findings reinforce the safety and efficacy of AZD1222 for protecting against symptomatic COVID-19, severe or critical disease, and SARS-CoV-2 infection. Widespread primary immunization remains a priority for controlling this global pandemic, but in the context of ongoing discussions regarding booster dosing, our findings indicate the durability of protection and immunogenicity of the primary AZD1222 series including, importantly, the maintained high level of protection against severe disease.

## Methods

### Study design and participants.

This ongoing, phase 3, randomized, placebo-controlled trial was conducted at 88 sites in the US (*n* = 82), Chile (*n* = 3), and Peru (*n* = 3). Full details of the trial design and participants have been reported previously (8). Briefly, participants were adults aged 18 or more years who were healthy or had medically stable chronic diseases and who were at increased risk of acquiring SARS-CoV-2 infection or becoming ill with COVID-19. Participants with a history of laboratory-confirmed SARS-CoV-2 infection, any confirmed or suspected immunosuppressive or immunodeficient state, or recurrent severe infections and use of immunosuppressant medication (except for HIV-positive participants on stable antiretroviral therapy) were excluded. Full eligibility criteria are provided in the protocol (Supplemental Methods 2). Participants received AZD1222 (5 × 10^10^ viral particles) or saline placebo via intramuscular injection on days 1 and 29. Full details of trial interventions are provided in the protocol (Supplemental Methods 2). The first 3000 participants randomized in the US (1500 participants aged 18–55 years, 750 aged 56–69 years, and 750 aged ≥70 years) were included in a substudy for further evaluation of immunogenicity and reactogenicity (substudy population) (8).

### Randomization.

Details of participant randomization have been reported previously (8). Briefly, participants were randomized to receive AZD1222 or saline placebo at a 2:1 ratio (Supplemental Methods) stratified by age (18–64 versus ≥65 years). This was designed as a double-blind study; AZD1222 dose preparation was done by an unblinded pharmacist or designee at each study site. Participants could be unblinded for safety or for potential receipt of a licensed or authorized COVID-19 vaccine once they became eligible.

### Procedures.

Study procedures have been described previously (8); full details are available in the protocol (Supplemental Methods 2). Participants were scheduled for study visits on days 1, 29, 57, 90, 180, 360, and 730. Serum samples for SARS-CoV-2 serology testing for nucleocapsid antibodies were collected at all visits (prior to dosing on days 1 and 29), and additional serum samples were collected prior to dosing on days 1 and 29 and on day 57 for exploratory immunogenicity assessments. Participants in the substudy attended 2 additional site visits on days 15 and 43; serum samples were collected at all site visits for SARS-CoV-2 serology testing, including assays for spike-binding antibodies, and assessment of SARS-CoV-2 and vector-neutralizing antibodies, as previously described (8). Participants self-monitored for COVID-19 qualifying symptoms (Supplemental Methods) through day 360 and received weekly automated electronic reminders from study sites. All participants with qualifying symptoms attended an illness visit, and those with a positive local SARS-CoV-2 RT-PCR result continued scheduled illness visits for up to 28 days (Supplemental Methods). In participants with RT-PCR–positive SARS-CoV-2 infections, nasopharyngeal swabs and saliva samples were analyzed for SARS-CoV-2 variant identification (Supplemental Methods). AEs were recorded through day 57 (28 days after the second dose) at study visits and via telephone, and SAEs, MAAEs, and AESIs continue at this writing to be recorded at each study visit through day 730 and via telephone, regardless of unblinding or receipt of nonstudy COVID-19 vaccination (Supplemental Methods).

### Outcomes.

The primary end points included reactogenicity (reported in full at the primary analysis, ref. 8), safety, and tolerability. The primary efficacy end point was the occurrence of confirmed SARS-CoV-2 RT-PCR–positive symptomatic COVID-19 at 15 or more days after the second dose in participants who were seronegative for SARS-CoV-2 at baseline (definition of symptomatic is provided in the Supplemental Methods). Secondary end points included incidence of the following: symptomatic illness 15 or more days after the second dose regardless of prior infection; severe or critical symptomatic COVID-19 (defined in Supplemental Methods); emergency department visits related to COVID-19; and SARS-CoV-2 infection regardless of symptoms or severity, defined as negative at baseline and positive after baseline for SARS-CoV-2 nucleocapsid antibodies. All trial objectives and end points are presented in the protocol (Supplemental Methods 2).

### Statistics.

The statistical design of the trial has been reported previously (8). Because formal statistical significance for the primary end point was achieved at the previous analysis (8), i.e., the success criterion (null hypothesis of VE = 30% rejected with an observed VE > 50%) had been met, this updated analysis of the primary and secondary efficacy end points used a nominal statistical significance level of 5%. VE for all primary and secondary end points was calculated as (1 minus relative risk) × 100, with relative risk estimated using Poisson’s regression model with robust variance, including trial group and age group (18–64 versus ≥65 years) at the time of informed consent as covariates, and the logarithm of follow-up time as an offset term to adjust for participants having different exposure times. To assess the durability of protection, the VE estimate was obtained from the model with follow-up time censored at the date of nonstudy COVID-19 vaccination (regardless of unblinding), non-COVID–related death, early termination, or data cutoff (July 30, 2021), whichever occurred first. Participants who were censored prior to having met the criteria for the efficacy end point were not counted as having the event. If the number of participants in any stratum was too small and/or convergence could not be achieved with Poisson’s regression analysis model, the model was reduced to exclude the age group covariate. If convergence was still not achieved, a stratified exact Poisson’s regression model was used. The primary end point was analyzed in subgroups (8) and by time period. The safety population comprised participants who received at least 1 dose of AZD1222 or placebo, analyzed according to intervention actually received. For analyses of safety, participants were censored at nonstudy COVID-19 vaccination or date of last trial contact, whichever occurred first. Frequencies of AEs were reported descriptively as numbers, percentages, and incidence rates, with no statistical analyses planned for comparisons between groups. The FVAS included all participants who were SARS-CoV-2–seronegative at baseline, received both doses, and remained in the trial for 15 or more days after the second dose without prior confirmed SARS-CoV-2 RT-PCR–positive infection. The FVAS population for the double-blind period excluded participants who were unblinded, received nonstudy COVID-19 vaccination, or had a confirmed SARS-CoV-2 RT-PCR–positive infection prior to 15 days after the second dose.

For analyses of efficacy in the double-blind period, participants were censored at unblinding, nonstudy COVID-19 vaccination, or date of last trial contact, whichever occurred first. For analyses of efficacy for the period to nonstudy COVID-19 vaccination, participants were censored at nonstudy COVID-19 vaccination date or date of last trial contact, regardless of unblinding. A higher proportion of participants in the placebo group received nonstudy COVID-19 vaccination and did so earlier following unblinding compared with those in the AZD1222 group. We therefore conducted sensitivity analyses to assess the impact of missing data in the context of the resultant difference between groups in follow-up time through to nonstudy COVID-19 vaccination. These included a multiple imputation approach for missing outcomes due to censoring or other reasons for truncated follow-up, an IPCW approach, and a multicovariate analysis using the primary analysis model (8).

As a sensitivity analysis to the handling of missing data in the evaluation of the primary efficacy end point, the analysis was repeated with multiple imputation for intercurrent events. For participants in the FVAS who did not meet the criteria for the primary end point prior to an intercurrent event (e.g., due to study withdrawal, being lost to follow-up, having died due to causes other than SARS-CoV-2, having been unblinded, or having received nonstudy COVID-19 vaccination), event status was imputed through to time of data cutoff assuming the observed event rate per study arm was conditional on age-group risk covariate (18–64 years, ≥65 years) using a multiple imputation approach at the participant level. The complete data set (including both observed and imputed events) was analyzed using Poisson’s regression model with robust variance, including age group at informed consent as a covariate, without log follow-up time as an offset. This process was repeated 20 times, and SAS PROC MIANALYZE 9 (https://support.sas.com/rnd/app/stat/procedures/mianalyze.html) was used to combine inferences from the 20 completed data sets, resulting in a combined point estimate for VE. The multiple imputation analysis was additionally conducted with imputation conditional on various other risk covariates, which included sex at birth (male, female), race (White, Black or African American, Asian, other), ethnicity (Hispanic or Latinx, not Hispanic or Latinx, not reported, unknown), body mass index (<40, ≥40, missing), comorbidities (yes, no, missing), OSHA risk category (very high, high, medium, lower exposure risk, missing), and region (East North Central, East South Central, Middle Atlantic, Mountain, New England, Pacific, South America, South Atlantic, West North Central, West South Central).

Uptake of nonstudy COVID-19 vaccination was observed at higher rates in participants in the placebo group. Consequently, longer-term estimates of VE may be biased, as informative censoring resulted in an analysis population disrupted from the original randomization schema. The IPCW technique was designed to create the counterfactual scenario of no censoring, i.e., to recover the information lost by informative censoring and provide an unbiased estimate of VE of AZD1222 compared with placebo in a hypothetical world without censoring. Valid estimation requires correct specification of the censoring model.

For the IPCW sensitivity analysis, counting process format data were generated for each participant by splitting follow-up time (days since 14 days following second dose of primary vaccination series) into subintervals during which the participant was at risk of COVID-19 and whose end points corresponded to each unique censoring time observed for the entire sample of participants; thus, subinterval lengths varied according to observed censoring times. A participant with a COVID-19 event had their last subinterval with upper limit equal to the time of COVID-19 diagnosis, while a censored participant had their last subinterval with upper limit equal to the time of censoring (which is one of the unique censoring times used to generate the counting process data).

This data format permits the estimation of probabilities of remaining uncensored at precisely the observed censoring times: for each participant and each subinterval, the cumulative probability of remaining uncensored up to and including the previous subinterval was calculated. Participant-level weights were then calculated as the inverse of these cumulative probabilities. Right censoring was defined as right censoring for any reason, which was predominantly due to use of nonstudy COVID-19 vaccination — COVID-19 events and administrative censoring were noncensoring events (administrative censoring assumed to be noninformative for infection events).

For each treatment group separately, the probabilities of remaining uncensored were estimated by fitting Cox’s proportional hazards (PH) model to the above counting process format data, where robust standard errors were used to account for the participant-level clustering. This censoring model adjusted for the following: treatment group; date of second dose of primary vaccination series (8 groups of 14 days in length beginning on November 1, 2020, and ending on February 28, 2021); age as a continuous variable; geographic region (9 US census divisions, per those used in the multiple imputation analyses, and Peru and Chile defined as South America); OSHA risk category (low, medium, high, and very high); comorbidities (yes, no); race (Black or African American, White, other); ethnicity (Hispanic or Latinx, not Hispanic or Latinx, not reported); and sex.

For all participants and for all at-risk subintervals, the censoring model was used to estimate the cumulative probability (*p*) of not being censored up to and including the previous subinterval, yielding 1 *p* and 1 inverse weight (*w* = 1/*p*) per at-risk subinterval per participant. For all participants until no longer at risk (due to COVID-19 event, right censoring, or administrative censoring), the time-varying weights were used in a weighted Cox PH model fitted to the same counting process format data as for the censoring model, with age (18–65 and >65 years) and treatment group (AZD1222 or placebo) as factor variables. Just as for the censoring model, robust standard errors were used to account for participant-level clustering. Results were reported based on standardized weights and truncated weights; standardizing and truncating weights are 2 strategies for minimizing the effects of extreme weights.

For analyses of immunogenicity in the substudy population, participants were planned to be censored at nonstudy COVID-19 vaccination date or date of last trial contact, regardless of unblinding, in order to provide comprehensive information on durability of immunogenicity after vaccination. However, data from the placebo group showed enrichment of antispike/neutralizing antibodies over time, suggesting potential unreported nonstudy COVID-19 vaccinations. Participants in both groups were thus censored at the earliest date of nonstudy COVID-19 vaccination, positive test for SARS-CoV-2 nucleocapsid antibodies, RT-PCR–positive SARS-CoV-2 symptomatic infection, or last trial contact, excluding or including date of unblinding, with the aim of excluding effects of unreported nonstudy COVID-19 vaccinations.

### Study approval.

The protocol and amendments for this trial (ClinicalTrials.gov NCT04516746) were approved by the ethics committee or institutional review board at each center, and the trial was conducted in compliance with the principles of the Declaration of Helsinki and the International Council for Harmonization Good Clinical Practice guidelines. Prior to enrolment, all participants provided informed consent.

### Data sharing.

Data underlying the findings described in this manuscript may be obtained in accordance with AstraZeneca’s data sharing policy described at https://astrazenecagrouptrials.pharmacm.com/ST/Submission/Disclosure

## Author contributions

The study was designed by MES, JM, ARF, JAG, EJK, TV, and IH in collaboration with the US government and the sponsor. All trial site investigators gathered the data in collaboration with IQVIA, a contract research organization, and AstraZeneca. Data were analyzed by IQVIA, AstraZeneca, and JM, SS, HJ, HB, LME, JAG, EJK, KS, TW, and IH, and data were interpreted by all the authors. Statistical analysis was done by SS, HJ, HB, LME, KS, and IH. SS, HB, EJK, KS, TW, and IH directly accessed and verified the underlying data reported in the manuscript. The first draft of the manuscript was written under the direction of the authors by a medical writer funded by AstraZeneca. All authors reviewed and provided substantive revisions to subsequent drafts, and all authors approved the final draft and the decision to submit for publication. The order for co–first authorship was agreed upon in a meeting between lead authors and in consideration of previous collaborative publications pertaining to this study.

## Supplementary Material

Trial reporting checklists

ICMJE disclosure forms

Supplemental data

Supplemental data 2

## Figures and Tables

**Table 1:**
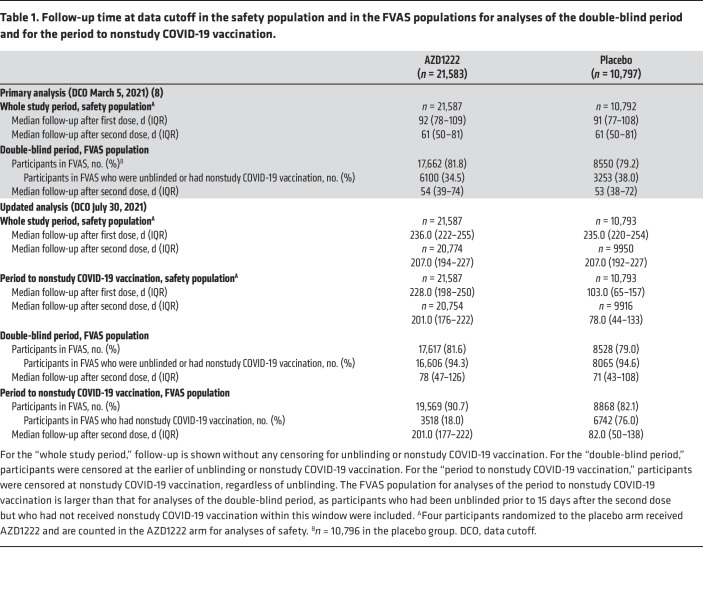
Follow-up time at data cutoff in the safety population and in the FVAS populations for analyses of the double-blind period and for the period to nonstudy COVID-19 vaccination.

## Version 1 09/15/2022

Electronic publication
