# Supplementary material for: Durability of protection and immunogenicity of AZD1222 (ChAdOx1 nCoV-19) COVID-19 vaccine over 6 months
Source: J Clin Invest. 2022 Sep 15;132(18):e160565. doi: 10.1172/JCI160565 (PMC9479753; doi:10.1172/JCI160565)
Supplement: Supplemental data [file jci-132-160565-s149.pdf]

Supplemental Methods, Tables, and Figures for:

Durability of protection and immunogenicity of AZD1222 (ChAdOx1 nCoV-19) COVID-19 vaccine over 6 months

Magdalena E. Sobieszczyk,* Jill Maaske,* Ann R. Falsey,* Stephanie Sproule, Merlin L. Robb, Robert W. Frenck Jr, Hong-Van Tieu, Kenneth H. Mayer, Lawrence Corey, Kathleen M. Neuzil, Tina Tong, Margaret Brewinski Isaacs, Holly Janes, Himanshu Bansal, Lindsay M. Edwards, Justin A. Green, Elizabeth J. Kelly, Kathryn Shoemaker, Therese Takas, Tom White, Prakash Bhuyan, Tonya Villafana, Ian Hirsch, on behalf of the AstraZeneca AZD1222 Clinical Study Group†
*Contributed equally
†AZD1222 Clinical Study Group members are listed in the authorship of and appendix to the primary manuscript (Falsey AR, et al, N Engl J Med 2021) (1); this list is reproduced below in the Supplemental Acknowledgments section. Group members contributing to the findings reported in this manuscript are named as authors or listed in the Acknowledgments section of the main manuscript.

Supplemental Methods, Tables, and Figures: Table of contents

Content	Page
**Supplemental Methods**	3
Randomization and masking	3
Qualifying symptoms for triggering participants to report to study sites for illness visits	3
Illness visits	3
SARS-CoV-2 variant identification	3
Safety monitoring	3
Definition of ‘symptomatic’ for the primary efficacy endpoint	3
Definition of ‘severe or critical’ COVID-19	4
Immunogenicity data: conversion to WHO International Standard	4
**Supplemental tables**	
Table 1. Dates of emergency use authorization (EUA) of COVID-19 vaccines in the United States, Chile, and Peru during the course of the phase 3 trial.	5
Table 2. Participant demographics and clinical characteristics, safety population.	6
Table 3. Summary of unsolicited AEs reported within 28 days of first, second, or either dose, safety population (per intervention received, with censoring at unblinding or receipt of non-study COVID-19 vaccination).	7
Table 4. Unsolicited AEs reported within 28 days of either dose with an incidence of ≥1% in either group, safety population (per intervention received; with censoring at unblinding or receipt of non-study COVID-19 vaccination).	8
Table 5. Summary of unsolicited AEs reported during the entire study period prior to non-study COVID-19 vaccination; safety population (per intervention received; with censoring at receipt of non-study COVID-19 vaccination, regardless of unblinding).	9
Table 6. Related MAAEs by system organ class and preferred term reported during the entire period of the study prior to non-study COVID-19 vaccination; safety population (per intervention received; with censoring at receipt of non-study COVID-19 vaccination, regardless of unblinding).	10
Table 7. Related AESIs by system organ class and preferred term reported during the entire period of the study prior to non-study COVID-19 vaccination; safety population (per intervention received; with censoring at receipt of non-study COVID-19 vaccination, regardless of unblinding).	13
Table 8. Participant demographics and clinical characteristics, FVAS population, double-blind period.	14
Table 9. Estimates of VE for the primary endpoint (RT-PCR-confirmed symptomatic COVID-19) by time period in the full analysis set for the double-blind period and for the period to non-study COVID-19 vaccination, and in the FVAS for the period to non-study COVID-19 vaccination.	15
Table 10. Participant demographics and clinical characteristics, FVAS population for analysis of period to non-study COVID-19 vaccination.	16
Table 11. Sensitivity analyses for estimates of efficacy with AZD1222 versus placebo for the primary efficacy endpoint in the FVAS population for the period up to receipt of non-study COVID-19 vaccination.	17
Table 12. Identification of VoC or VoI and VE against VoC/VoI with ≥5 events (FVAS population, double-blind period).	18

**Supplemental figures**	
Figure 1. Efficacy of AZD1222 versus placebo for the prevention of COVID-19 and SARS-CoV-2 infection.	19
Figure 2. Kaplan–Meier analysis of non-study COVID-19 vaccination over time, regardless of unblinding.	20
Figure 3. Neutralizing and spike-binding antibody responses over time in the placebo group.	21
Figure 4. Neutralizing antibody responses over time by baseline comorbidities and serostatus.	22
Figure 5. Correlation between anti-vector immune response after first dose and ability to boost after second dose.	23
**Supplemental references**	24
**Supplemental acknowledgments**	25

Supplemental Methods

Randomization and masking

Participants were randomized to receive AZD1222 or saline placebo in a 2:1 ratio, with central assignment via an interactive response technology (IRT) that was used to generate and implement the random allocation sequence and generate dose-tracking numbers.

Qualifying symptoms for triggering participants to report to study sites for illness visits

Participants with fever, shortness of breath, or difficulty breathing, of any duration, and/or with chills, cough, fatigue, muscle aches, body aches, headache, new loss of taste, new loss of smell, sore throat, congestion, runny nose, nausea, vomiting, or diarrhea lasting ≥2 days were to contact their study site.

Illness visits

All participants with qualifying symptoms underwent scheduled illness visits for up to 28 days, including site visits on days 1, 14, 21, and 28 for collection of nasopharyngeal swabs for reverse transcriptase (RT)-PCR testing and for sequencing (days 1 and 14 only; sequencing was performed on the first available RT-PCR-positive sample), and serum samples for exploratory immunogenicity assessments (days 1, 14, 28). Participants received a digital health device and illness eDiary on which to record symptoms. In the US, saliva samples for assessment of viral shedding and SARS-CoV-2 sequencing were collected at all site visits and by the participants at home on days 3, 5, 8, and 11. Once SARS-CoV-2 RT-PCR results were available, only participants who were positive continued with the illness visits.

SARS-CoV-2 variant identification

Next-generation sequencing (NGS) of viral spike protein from nasopharyngeal swabs was done to assess individual amino acid changes. The full-length SARS CoV-2 spike gene was amplified and sequenced using a validated GenoSure SARS-CoV-2 spike NGS assay (Monogram Biosciences, South San Francisco, CA) and assessed at a consensus allele fraction of ≥25%. To assign a lineage, a spike-only version of Pangolin COVID-19 lineage assigner (2) (https://github.com/aineniamh/hedgehog, version 1.0.2) was used to classify SARS-CoV-2 spike sequences to current Pango lineages (v.1.2.6) (3) or sets of lineages. To provide supportive SARS-CoV-2 genotypic analyses, whole genome sequencing (WGS) data from saliva samples were additionally assessed as previously described (1) using the Illumina COVIDSeq Test and software to identify SARS-CoV-2 lineage and clade (3, 4).

Safety monitoring

The safety of all AstraZeneca clinical studies is closely monitored on an ongoing basis by AstraZeneca representatives in consultation with Patient Safety. Issues identified are addressed; for instance, this could involve amendments to the Clinical Study Protocol and letters to investigators. A Protocol Safety Review Team (PSRT) comprised of AstraZeneca, COVID-19 Prevention Network, Biomedical Advanced Research and Development Authority, and NIAID medical officers oversaw blinded safety surveillance of participants during the study. A COVID-19 Vaccine Data Safety Monitoring Board (DSMB) organized by the National Institutes of Health, National Institute for Allergy and Infectious Diseases, comprised of independent experts, was convened to provide oversight, and to ensure safe and ethical conduct of the study. The COVID-19 Vaccine DSMB facilitated the interim analysis for safety and efficacy and had the responsibility of evaluating cumulative safety and other clinical study data at regular intervals and making appropriate recommendations based on the available data. During the study, the benefit/risk assessment was continuously monitored by the COVID-19 Vaccine DSMB to ensure that the balance remained favorable. For example, events of potential vaccine-associated enhanced respiratory disease were evaluated by periodic reviews of COVID-19 cases by the DSMB. Harm for severe COVID-19 cases was any vaccine efficacy (VE) ≤ 0 for which Fisher's exact test (1-sided) was statistically significant at the 5% level. This assessment began after 8 cases of severe COVID-19 had accrued in the study and was performed in real time as events occurred. Harm monitoring included all COVID-19 cases and all severe COVID-19 cases from day 1 for participants in the full analysis set. Harm monitoring for overall COVID-19 cases used the same boundary as severe COVID-19 cases (i.e., VE ≤ 0 for which Fisher's exact test [1-sided] was statistically significant at the 5% level) but was performed on a weekly basis. An independent Neurological Adverse Events of Special Interest (AESI) Expert Committee was available to review and provide advice to the PSRT and the COVID-19 Vaccine DSMB on request about the diagnosis and causality assessment of selected neurologic AESIs occurring in the AZD1222 clinical development program.

Definition of 'symptomatic' for the primary efficacy endpoint

For the primary efficacy endpoint, 'symptomatic' was defined as: i) one or more of: pneumonia diagnosed by chest x-ray or computed tomography scan; oxygen saturation of ≤94% on room air or requiring either new

initiation or escalation in supplemental O_2; new or worsening dyspnea/shortness of breath; or ii) two or more of: fever >100°F (>37.8°C) or feverishness; new or worsening cough; myalgia/muscle pain; fatigue that interferes with activities of daily living; vomiting and/or diarrhea; anosmia and/or ageusia.

Definition of 'severe or critical' COVID-19
'Severe or critical COVID-19' was defined as SARS-CoV-2 RT-PCR-positive symptomatic illness plus any of: clinical signs at rest indicative of severe systemic illness (respiratory rate ≥30 breaths per minute, heart rate ≥125 beats per minute, oxygen saturation ≤93% on room air at sea level, or partial pressure of oxygen to fraction of inspired oxygen ratio <300 mmHg); respiratory failure (defined as needing high-flow oxygen, noninvasive ventilation, mechanical ventilation, or extracorporeal membrane oxygenation); evidence of shock (systolic blood pressure <90 mm Hg, diastolic blood pressure <60 mmHg, or requiring vasopressors); significant acute renal, hepatic, or neurologic dysfunction; admission to an intensive care unit; death.

Immunogenicity data: conversion to WHO International Standard
The validated multiplexed electrochemiluminescence-based serology assay for measuring spike-binding antibodies was analyzed utilizing a standard of pooled convalescent sera as described previously (1) in its own scale. A bridging experiment was performed to convert spike-binding units in arbitrary units per milliliter (AU)/mL to the WHO international standard (NIBSC 20/136) binding units (BAU/mL). The following formula may be applied to convert spike-binding titers from AU/mL to BAU/mL: (BAU/mL) = AU/mL * 0.00645. Therefore, 1000 BAU/mL = 155,039 AU/mL.

The results of the pseudovirus neutralization assay performed at Monogram Biosciences are reported as an ID_{50} titer (1/dilution). Calibration factors to enable conversion from ID_{50} to the WHO international standard (NIBSC 20/136) in IU/mL were derived in a calibration study. The following formula may be applied to convert neutralizing antibody titers from ID_{50} to IU/mL: (IU/mL) = ID50 * 0.1428. Therefore, 1000 IU/mL = 7001.3 ID_{50}.

Table 1. Dates of emergency use authorization (EUA) of COVID-19 vaccines in the United States, Chile, and Peru during the course of the phase 3 trial.

Country	Vaccine	Date of EUA	Source
**United States**	BNT162b2	December 11, 2020	FDA
	mRNA-1273	December 18, 2020	FDA
	Ad26.COV2.S	February 27, 2021	FDA
**Chile**	BNT162b2	December 16, 2020	Reuters
	CoronaVac	January 20, 2021	Reuters
	AZD1222	January 27, 2021	Chile Reports
	Ad5-nCoV	April 7, 2021	Reuters
	Ad26.COV2.S	June 10, 2021	Reuters
	Sputnik V	July 21, 2021	Reuters
**Peru**	BNT162b2	February 2, 2021	Reuters
	BBIBP-CorV	January 27, 2021	Reuters
	AZD1222	September 1, 2021	Peru DigeMid
	Ad26.COV2.S	July 6, 2021	Peru DigeMid
	mRNA-1273	Not applicable	RPP Peru

Table 2. Participant demographics and clinical characteristics, safety population.

	AZD1222 (*n* = 21,587)	Placebo (*n* = 10,793)*
**Median age at screening, years (IQR)**	51.0 (38–63)	51.0 (38–63)
Age ≥18–64 years, no. (%)	16,759 (77.6)	8,382 (77.7)
Age ≥65 years, no. (%)	4,828 (22.4)	2,411 (22.3)
**Sex, no. (%)**		
Male	12,010 (55.6)	6,004 (55.6)
Female	9,577 (44.4)	4,789 (44.4)
**Race, no. (%)**		
White	17,062 (79.0)	8,523 (79.0)
Black or African American	1,793 (8.3)	892 (8.3)
Asian	947 (4.4)	482 (4.5)
American Indian or Alaska Native	853 (4.0)	428 (4.0)
Native Hawaiian or Other Pacific Islander	60 (0.3)	21 (0.2)
Multiple	511 (2.4)	257 (2.4)
Unknown or Not reported	361 (1.7)	190 (1.8)
**Hispanic or Latinx ethnicity, no. (%)**		
Yes	4,771 (22.1)	2,451 (22.7)
No	16,475 (76.3)	8,202 (76.0)
Unknown or not reported	341 (1.6)	140 (1.3)
**Country, no. (%)**		
United States	19,145 (88.7)	9,573 (88.7)
Chile	1,470 (6.8)	729 (6.8)
Peru	972 (4.5)	491 (4.5)
**Baseline SARS-CoV-2 serostatus, no. (%)**		
Negative	20,688 (95.8)	10,352 (95.9)
Positive	624 (2.9)	293 (2.7)
Missing or not done	275 (1.3)	148 (1.4)
**Baseline comorbidities, no. (%)**		
Yes	12,939 (59.9)	6,498 (60.2)
No	8,646 (40.1)	4,294 (39.8)
Missing	2	1
**COVID-19 exposure risk category (OSHA) , no. (%)**		
Very high	1,367 (6.4)	704 (6.6)
High	4,796 (22.4)	2,271 (21.3)
Medium	8,982 (42.0)	4,485 (42.0)
Lower	6,220 (29.1)	3,208 (30.1)
Missing	222	125
**Median dosing interval, days (IQR)**†	*n* = 20,774	*n* = 9,950
Overall	29.0 (29–30)	29.0 (29–30)
Participants randomized prior to clinical hold	*n* = 516	*n* = 259
	60.0 (57–63)	59.0 (57–62)
Participants randomized after clinical hold	*n* = 20,258	*n* = 9,691
	29.0 (29–30)	29.0 (29–30)

*1 participant was not included in the primary analysis (1) due to record deactivation but has been reinstated at this analysis.
†Because the trial was placed on clinical hold due to an event of transverse myelitis in a different study of AZD1222 (5), 775 participants received their second dose after a longer dosing interval (1).
IQR, interquartile range; OSHA, Occupational Safety and Health Administration.

Table 3. Summary of unsolicited AEs reported within 28 days of first, second, or either dose, safety population (per intervention received, with censoring at unblinding or receipt of non-study COVID-19 vaccination).

AE, no. (%)	AZD1222 post dose 1 (*n* = 21,587)	AZD1222 post dose 2 (*n* = 20,774)	AZD1222 post either dose (*n* = 21,587)	Placebo post dose 1 (*n* = 10,793)	Placebo post dose 2 (*n* = 9,950)	Placebo post either dose (*n* = 10,793)
**Any AE**	9,315 (43.2)	5,270 (25.4)	11,167 (51.7)	2,695 (25.0)	1,881 (18.9)	3,798 (35.2)
Any AE related to trial intervention	7,615 (35.3)	3,787 (18.2)	8,937 (41.4)	1,546 (14.3)	1,015 (10.2)	2,177 (20.2)
Severity						
Mild	5,762 (26.7)	3,222 (15.5)	6,681 (30.9)	1,305 (12.1)	866 (8.7)	1,804 (16.7)
Moderate	1,743 (8.1)	547 (2.6)	2,129 (9.9)	228 (2.1)	140 (1.4)	352 (3.3)
Grade ≥3	110 (0.5)	18 (<0.1)	127 (0.6)	13 (0.1)	9 (<0.1)	21 (0.2)
**Any SAE**	59 (0.3)	50 (0.2)	109 (0.5)	39 (0.4)	18 (0.2)	57 (0.5)
Any SAE related to trial intervention	1 (<0.1)	0	1 (<0.1)	1 (<0.1)	0	1 (<0.1)
**Any AE leading to discontinuation from trial intervention**	266 (1.2)	1 (<0.1)	267 (1.2)	160 (1.5)	0	160 (1.5)
Any related AE leading to discontinuation from trial intervention	22 (0.1)	1 (<0.1)	23 (0.1)	6 (<0.1)	0	6 (<0.1)
**Any AE leading to discontinuation from trial**	2 (<0.1)	1 (<0.1)	3 (<0.1)	5 (<0.1)	0	5 (<0.1)
**Any MAAE**	800 (3.7)	724 (3.5)	1,474 (6.8)	406 (3.8)	324 (3.3)	705 (6.5)
**Any AESI**	318 (1.5)	138 (0.7)	453 (2.1)	185 (1.7)	144 (1.4)	325 (3.0)
Any AESI related to trial intervention	42 (0.2)	22 (0.1)	62 (0.3)	17 (0.2)	12 (0.1)	26 (0.2)
**Any AE with outcome of death**	2 (<0.1)	1 (<0.1)	3 (<0.1)	5 (<0.1)	0	5 (<0.1)

AE, adverse event; AESI, adverse event of special interest; MAAE, mediy attended adverse event; SAE, serious adverse event.

Table 4. Unsolicited AEs reported within 28 days of either dose with an incidence of ≥1% in either group, safety population (per intervention received; with censoring at unblinding or receipt of non-study COVID-19 vaccination).

	AZD1222 (*n* = 21,587)		Placebo (*n* = 10,793)	
AE, no. (%)	All events	Related events	All events	Related events
Injection site pain	3,571 (16.5)	3,128 (14.5)	478 (4.4)	410 (3.8)
Headache	3,382 (15.7)	2,975 (13.8)	962 (8.9)	732 (6.8)
Fatigue	2,744 (12.7)	2,553 (11.8)	818 (7.6)	682 (6.3)
Chills	2,138 (9.9)	2,050 (9.5)	238 (2.2)	205 (1.9)
Pain	1,801 (8.3)	1,709 (7.9)	253 (2.3)	211 (2.0)
Myalgia	1,588 (7.4)	1,451 (6.7)	265 (2.5)	209 (1.9)
Pyrexia	1,126 (5.2)	1,077 (5.0)	64 (0.6)	39 (0.4)
Body temperature increased	769 (3.6)	737 (3.4)	94 (0.9)	75 (0.7)
Nausea	659 (3.1)	523 (2.4)	215 (2.0)	149 (1.4)
Diarrhea	552 (2.6)	328 (1.5)	243 (2.3)	126 (1.2)
Rhinorrhea	494 (2.3)	243 (1.1)	256 (2.4)	107 (1.0)
Oropharyngeal pain	432 (2.0)	194 (0.9)	235 (2.2)	91 (0.8)
Nasal congestion	350 (1.6)	158 (0.7)	223 (2.1)	90 (0.8)
Cough	340 (1.6)	125 (0.6)	190 (1.8)	68 (0.6)
Injection-related reaction	330 (1.5)	326 (1.5)	66 (0.6)	66 (0.6)
Reactogenicity event	322 (1.5)	317 (1.5)	50 (0.5)	49 (0.5)
Pain in extremity	305 (1.4)	225 (1.0)	75 (0.7)	46 (0.4)
COVID-19	296 (1.4)	0	265 (2.5)	0
Malaise	265 (1.2)	246 (1.1)	68 (0.6)	60 (0.6)
Arthralgia	251 (1.2)	156 (0.7)	59 (0.5)	17 (0.2)

AE, adverse event.

Table 5. Summary of unsolicited AEs reported during the entire study period prior to non-study COVID-19 vaccination; safety population (per intervention received; with censoring at receipt of non-study COVID-19 vaccination, regardless of unblinding).

AE	AZD1222 (*n* = 21,587) (Total follow-up: 12.19 x 1,000 person-years) No. (%)	IR*	Placebo (*n* = 10,793) (Total follow-up: 1.47 x 1,000 person-years) No. (%)	IR*
**Any AE**	12,062 (55.9)	0.99	4,171 (38.6)	1.19
Any grade ≥3 AE	611 (2.8)	0.05	213 (2.0)	0.06
Any AE related to trial intervention	8,988 (41.6)	0.74	2,181 (20.2)	0.62
Severity				
Mild	6,706 (31.1)	0.55	1,806 (16.7)	0.52
Moderate	2,151 (10.0)	0.18	352 (3.3)	0.10
Grade ≥3	131 (0.6)	0.01	23 (0.2)	<0.01
**Any SAE**	353 (1.6)	0.03	121 (1.1)	0.03
Any SAE related to trial intervention	2 (<0.1)	<0.01	2 (<0.1)	<0.01
Chronic inflammatory demyelinating polyradiculoneuropathy	1 (<0.1)	<0.01	0	0
Hypoesthesia	1 (<0.1)	<0.01	0	0
Neurosensory hypoacusis	0	0	1 (<0.1)	<0.01
Optic ischemic neuropathy	0	0	1 (<0.1)	<0.01
Paresthesia	1 (<0.1)	<0.01	0	0
**Any AE leading to discontinuation from trial intervention**	286 (1.3)	0.02	172 (1.6)	0.05
Any related AE leading to discontinuation from trial intervention	23 (0.1)	<0.01	6 (<0.1)	<0.01
**Any AE leading to discontinuation from trial**	10 (<0.1)	<0.01	7 (<0.1)	<0.01
**Any MAAE**	3,162 (14.6)	0.26	1,135 (10.5)	0.32
Any related MAAE	100 (0.5)	<0.01	32 (0.3)	<0.01
**Any AESI**	932 (4.3)	0.08	528 (4.9)	0.15
Any AESI related to trial intervention	63 (0.3)	<0.01	27 (0.3)	<0.01
Any potentially immune-mediated condition (PIMC)	736 (3.4)	0.06	471 (4.4)	0.13
Any related	5 (<0.1)	<0.01	2 (<0.1)	<0.01
Any neurologic and/or neuroinflammatory AESI	148 (0.7)	0.01	51 (0.5)	0.01
Any related	60 (0.3)	<0.01	26 (0.2)	<0.01
Any vascular AESI	68 (0.3)	<0.01	14 (0.1)	<0.01
Any related	0	0	0	0
Any hematologic AESI	4 (<0.1)	<0.01	1 (<0.1)	<0.01
Any related	0	0	1 (<0.1)	<0.01
**Any AE with outcome of death**	14 (<0.1)	<0.01	8 (<0.1)	<0.01
Any related AE with outcome of death	0	0	0	0

*Exposure-adjusted incidence rate, per person-years.
AE, adverse event; AESI, adverse event of special interest; IR, incidence rate; MAAE, medically attended adverse event; PIMC, potentially immune-mediated condition; SAE, serious adverse event.

Table 6. Related MAAEs by system organ class and preferred term reported during the entire period of the study prior to non-study COVID-19 vaccination; safety population (per intervention received; with censoring at receipt of non-study COVID-19 vaccination, regardless of unblinding).

Related MAAEs	AZD1222 (*n* = 21,587) (Total follow-up: 12.19 x 1,000 person-years) No. (%)	IR*	Placebo (*n* = 10,793) (Total follow-up: 1.47 x 1,000 person-years) No. (%)	IR*
**Any**	100 (0.5)	<0.01	32 (0.3)	<0.01
**Blood and lymphatic system disorders**	0	0	1 (<0.1)	<0.01
Immune thrombocytopenia	0	0	1 (<0.1)	<0.01
**Cardiac disorders**	2 (<0.1)	<0.01	0	0
Palpitations	1 (<0.1)	<0.01	0	0
Tachycardia	1 (<0.1)	<0.01	0	0
**Ear and labyrinth disorders**	4 (<0.1)	<0.01	2 (<0.1)	<0.01
Tinnitus	2 (<0.1)	<0.01	0	0
Vertigo	2 (<0.1)	<0.01	0	0
Deafness bilateral	1 (<0.1)	<0.01	0	0
Neurosensory hypoacusis	0	0	1 (<0.1)	<0.01
Sudden hearing loss	0	0	1 (<0.1)	<0.01
**Eye disorders**	0	0	2 (<0.1)	<0.01
Eye swelling	0	0	1 (<0.1)	<0.01
Optic ischemic neuropathy	0	0	1 (<0.1)	<0.01
**Gastrointestinal disorders**	11 (<0.1)	<0.01	4 (<0.1)	<0.01
Diarrhea	4 (<0.1)	<0.01	1 (<0.1)	<0.01
Vomiting	4 (<0.1)	<0.01	0	0
Paresthesia oral	2 (<0.1)	<0.01	1 (<0.1)	<0.01
Nausea	2 (<0.1)	<0.01	0	0
Abdominal pain upper	0		1 (<0.1)	<0.01
Bowel movement irregularity	1 (<0.1)	<0.01	0	0
Gastro-esophageal reflux disease	1 (<0.1)	<0.01	0	0
Lip swelling	1 (<0.1)	<0.01	0	0
Parotid gland enlargement	0	0	1 (<0.1)	<0.01
Stomatitis	1 (<0.1)	<0.01	0	0
**General disorders and administration site conditions**	35 (0.2)	<0.01	5 (<0.1)	<0.01
Fatigue	9 (<0.1)	<0.01	1 (<0.1)	<0.01
Pyrexia	9 (<0.1)	<0.01	0	0
Pain	7 (<0.1)	<0.01	0	0
Chills	6 (<0.1)	<0.01	1 (<0.1)	<0.01
Injection site pain	3 (<0.1)	<0.01	0	0
Influenza-like illness	2 (<0.1)	<0.01	0	0
Injection site erythema	2 (<0.1)	<0.01	0	0
Reactogenicity event	1 (<0.1)	<0.01	1 (<0.1)	<0.01
Asthenia	1 (<0.1)	<0.01	0	0
Chest pain	0	0	1 (<0.1)	<0.01
Discomfort	1 (<0.1)	<0.01	0	0
Feeling abnormal	1 (<0.1)	<0.01	0	0
Feeling hot	1 (<0.1)	<0.01	0	0
Injection site paresthesia	1 (<0.1)	<0.01	0	0
Injection site pruritus	1 (<0.1)	<0.01	0	0
Injection site reaction	1 (<0.1)	<0.01	0	0
Injection site swelling	1 (<0.1)	<0.01	0	0
Injury associated with device	0	0	1 (<0.1)	<0.01
Non-cardiac chest pain	1 (<0.1)	<0.01	0	0
Peripheral swelling	1 (<0.1)	<0.01	0	0
Swelling	1 (<0.1)	<0.01	0	0
**Immune system disorders**	1 (<0.1)	<0.01	0	0
Drug hypersensitivity	1 (<0.1)	<0.01	0	0
**Infections and infestations***	7 (<0.1)	<0.01	1 (<0.1)	<0.01
Herpes zoster	4 (<0.1)	<0.01	0	0

Related MAAEs	AZD1222 (*n* = 21,587) (Total follow-up: 12.19 x 1,000 person-years) No. (%)	IR*	Placebo (*n* = 10,793) (Total follow-up: 1.47 x 1,000 person-years) No. (%)	IR*
Cellulitis	1 (<0.1)	<0.01	0	0
Injection site cellulitis	1 (<0.1)	<0.01	0	0
Nasopharyngitis	0	0	1 (<0.1)	<0.01
Oral herpes	1 (<0.1)	<0.01	0	0
**Injury, poisoning and procedural complications**	3 (<0.1)	<0.01	2 (<0.1)	<0.01
Injection-related reaction	0	0	2 (<0.1)	<0.01
Chilblains	1 (<0.1)	<0.01	0	0
Seroma	1 (<0.1)	<0.01	0	0
Skin laceration	1 (<0.1)	<0.01	0	0
**Investigations**	3 (<0.1)	<0.01	0	0
Body temperature increased	3 (<0.1)	<0.01	0	0
**Metabolism and nutrition disorders**	2 (<0.1)	<0.01	0	0
Dehydration	1 (<0.1)	<0.01	0	0
Hyperlactacidemia	1 (<0.1)	<0.01	0	0
Hypokalemia	1 (<0.1)	<0.01	0	0
**Musculoskeletal and connective tissue disorders**	13 (<0.1)	<0.01	6 (<0.1)	<0.01
Myalgia	3 (<0.1)	<0.01	1 (<0.1)	<0.01
Arthralgia	3 (<0.1)	<0.01	0	0
Back pain	0	0	3 (<0.1)	<0.01
Muscle fatigue	0	0	1 (<0.1)	<0.01
Muscle spasms	1 (<0.1)	<0.01	0	0
Muscular weakness	1 (<0.1)	<0.01	0	0
Musculoskeletal pain	1 (<0.1)	<0.01	0	0
Neck pain	1 (<0.1)	<0.01	0	0
Pain in extremity	1 (<0.1)	<0.01	0	0
Pain in jaw	0	0	1 (<0.1)	<0.01
Polymyalgia rheumatica	1 (<0.1)	<0.01	0	0
Rheumatoid arthritis	1 (<0.1)	<0.01	0	0
Tendonitis	1 (<0.1)	<0.01	0	0
**Nervous system disorders**	30 (0.1)	<0.01	6 (<0.1)	<0.01
Headache	10 (<0.1)	<0.01	3 (<0.1)	<0.01
Paresthesia	9 (<0.1)	<0.01	2 (<0.1)	<0.01
Dizziness	4 (<0.1)	<0.01	1 (<0.1)	<0.01
Hypoesthesia	4 (<0.1)	<0.01	1 (<0.1)	<0.01
Syncope	2 (<0.1)	<0.01	0	0
Ageusia	1 (<0.1)	<0.01	0	0
Chronic inflammatory demyelinating polyradiculoneuropathy	1 (<0.1)	<0.01	0	0
Facial paralysis	1 (<0.1)	<0.01	0	0
Guillain-Barré syndrome	1 (<0.1)	<0.01	0	0
Migraine	1 (<0.1)	<0.01	0	0
Occipital neuralgia	1 (<0.1)	<0.01	0	0
Tremor	1 (<0.1)	<0.01	0	0
**Psychiatric disorders**	2 (<0.1)	<0.01	0	0
Anxiety	1 (<0.1)	<0.01	0	0
Depression	1 (<0.1)	<0.01	0	0
Insomnia	1 (<0.1)	<0.01	0	0
**Respiratory, thoracic and mediastinal disorders**	9 (<0.1)	<0.01	4 (<0.1)	<0.01
Nasal congestion	3 (<0.1)	<0.01	1 (<0.1)	<0.01
Oropharyngeal pain	2 (<0.1)	<0.01	2 (<0.1)	<0.01
Cough	2 (<0.1)	<0.01	1 (<0.1)	<0.01
Dyspnea	2 (<0.1)	<0.01	1 (<0.1)	<0.01
Sinus congestion	1 (<0.1)	<0.01	0	0
Sneezing	0	0	1 (<0.1)	<0.01
**Skin and subcutaneous tissue disorders**	8 (<0.1)	<0.01	6 (<0.1)	<0.01
Dermatitis allergic	1 (<0.1)	<0.01	1 (<0.1)	<0.01

Related MAAEs	AZD1222 (*n* = 21,587) (Total follow-up: 12.19 x 1,000 person-years) No. (%)	IR*	Placebo (*n* = 10,793) (Total follow-up: 1.47 x 1,000 person-years) No. (%)	IR*
Rash maculo-papular	1 (<0.1)	<0.01	1 (<0.1)	<0.01
Seborrheic dermatitis	2 (<0.1)	<0.01	0	0
Urticaria	2 (<0.1)	<0.01	0	0
Dermatitis	0	0	1 (<0.1)	<0.01
Hyperhidrosis	0	0	1 (<0.1)	<0.01
Idiopathic urticaria	1 (<0.1)	<0.01	0	0
Neurodermatitis	0	0	1 (<0.1)	<0.01
Petechiae	0	0	1 (<0.1)	<0.01
Pruritus	1 (<0.1)	<0.01	0	0
**Vascular disorders**	2 (<0.1)	<0.01	0	0
Hypertension	2 (<0.1)	<0.01	0	0

*Exposure-adjusted incidence rate, per person-years
IR, incidence rate; MAAE, medically attended adverse event.

Table 7. Related AESIs by system organ class and preferred term reported during the entire period of the study prior to non-study COVID-19 vaccination; safety population (per intervention received; with censoring at receipt of non-study COVID-19 vaccination, regardless of unblinding).

	**AZD1222 (*n* = 21,587) (Total follow-up: 12.19 x 1,000 person-years)**		**Placebo (*n* = 10,793) (Total follow-up: 1.47 x 1,000 person-years)**	
**Related AESIs**	**No. (%)**	**IR***	**No. (%)**	**IR***
**Any**	63 (0.3)	<0.01	27 (0.3)	<0.01
Any neurologic and/or neuroinflammatory	60 (0.3)	<0.01	26 (0.2)	<0.01
**Hematologic**	0	0	1 (<0.1)	<0.01
Immune thrombocytopenia	0	0	1 (<0.1)	<0.01
**Neurologic**	59 (0.3)	<0.01	26 (0.2)	<0.01
Paresthesia	37 (0.2)	<0.01	16 (0.1)	<0.01
Hypoesthesia	15 (<0.1)	<0.01	4 (<0.1)	<0.01
Muscular weakness	7 (<0.1)	<0.01	1 (<0.1)	<0.01
Dysesthesia	0	0	3 (<0.1)	<0.01
Hyperesthesia	3 (<0.1)	<0.01	0	0
Chronic inflammatory demyelinating polyradiculoneuropathy	1 (<0.1)	<0.01	0	0
Guillain-Barré syndrome	1 (<0.1)	<0.01	0	0
Neuritis	0	0	1 (<0.1)	<0.01
Neuropathy peripheral	1 (<0.1)	<0.01	0	0
Polyneuropathy	0	0	1 (<0.1)	<0.01
Sensory disturbance	0	0	1 (<0.1)	<0.01
**Potentially immune-mediated conditions (PIMC)**	5 (<0.1)	<0.01	2 (<0.1)	<0.01
PIMC – Musculoskeletal disorders	2 (<0.1)	<0.01	0	0
Polymyalgia rheumatica	1 (<0.1)	<0.01	0	0
Rheumatoid arthritis	1 (<0.1)	<0.01	0	0
PIMC – Neuroinflammatory disorders†	2 (<0.1)	<0.01	1 (<0.1)	<0.01
Chronic inflammatory demyelinating polyradiculoneuropathy	1 (<0.1)	<0.01	0	0
Facial paralysis	1 (<0.1)	<0.01	0	0
Guillain-Barré syndrome	1 (<0.1)	<0.01	0	0
Polyneuropathy	0	0	1 (<0.1)	<0.01
PIMC – Vasculitides	1 (<0.1)	<0.01	0	0
Vasculitis	1 (<0.1)	<0.01	0	0
PIMC – Others	0	0	1 (<0.1)	<0.01
Immune thrombocytopenia	0	0	1 (<0.1)	<0.01

*Exposure-adjusted incidence rate, per person-years

†The PTs in this category are included in the neurologic and potential immune-mediated conditions category of neuroinflammatory events.

AESI, adverse event of special interest; IR, incidence rate; PIMC, potentially immune-mediated condition.

Table 8. Participant demographics and clinical characteristics, FVAS population, double-blind period.

	AZD1222 (*n* = 17,617)	Placebo (n = 8528)
**Median age at screening, years (IQR)**	51.0 (38–63)	51.0 (38–63)
Age ≥18–64 years, no. (%)	13,921 (79.0)	6,712 (78.7)
Age ≥65 years, no. (%)	3,696 (21.0)	1,816 (21.3)
**Sex, no. (%)**		
Male	9,885 (56.1)	4,814 (56.4)
Female	7,732 (43.9)	3,714 (43.6)
**Race, no. (%)**		
White	13,972 (79.3)	6,735 (79.0)
Black or African American	1,401 (8.0)	699 (8.2)
Asian	738 (4.2)	355 (4.2)
American Indian or Alaska Native	747 (4.2)	372 (4.4)
Native Hawaiian or Other Pacific Islander	50 (0.3)	15 (0.2)
Multiple	421 (2.4)	203 (2.4)
Unknown or Not reported	288 (1.6)	149 (1.7)
**Hispanic or Latinx ethnicity, no. (%)**		
Yes	4,032 (22.9)	2,064 (24.2)
No	13,315 (75.6)	6,347 (74.4)
Unknown or not reported	270 (1.5)	117 (1.4)
**Country, no. (%)**		
United States	15,389 (87.4)	7,423 (87.0)
Chile	1,358 (7.7)	670 (7.9)
Peru	870 (4.9)	435 (5.1)
**Baseline comorbidities, no. (%)**		
Yes	10,524 (59.7)	5,150 (60.4)
No	7,092 (40.3)	3,377 (39.6)
**COVID-19 exposure risk category (OSHA) , no. (%)**	,	
Very high	956 (5.5)	446 (5.3)
High	3,723 (21.4)	1,651 (19.6)
Medium	7,544 (43.3)	3,659 (43.4)
Lower	5,204 (29.9)	2,666 (31.7)
Missing	190	106
**Median dosing interval, days (IQR)***	*n* = 17,617	*n* = 8,528
Overall	29.0 (29–30)	29.0 (29–30)
Participants randomized prior to clinical hold	*n* = 500	*n* = 248
	60.0 (57–63)	59.0 (57–62)
Participants randomized after clinical hold	*n* = 17,117	*n* = 8,280
	29.0 (29–30)	29.0 (29–30)

*Because the trial was placed on clinical hold due to an event of transverse myelitis in a different study of AZD1222 (5), 775 participants received their second dose after a longer dosing interval (1).

FVAS, fully vaccinated analysis set; IQR, interquartile range; OSHA, Occupational Safety and Health Administration.

Table 9. Estimates of VE for the primary endpoint (RT-PCR-confirmed symptomatic COVID-19) by time period in the full analysis set for the double-blind period and for the period to non-study COVID-19 vaccination, and in the FVAS for the period to non-study COVID-19 vaccination; analyses are restricted to participant–time within a given time period.

Population / Time period	Events, no./No. AZD1222	Events, no./No. Placebo	Incidence rate* AZD1222	Incidence rate* Placebo	VE (95% CI)
**Full analysis set, double-blind period (censoring at unblinding or non-study COVID-19 vaccination)†**					
Any time post first dose	374/21,583	370/10,797	59.7	129.3	53.9 (46.7–60.1)
<15 days post first dose	96/21,583	45/10,797	116.9	109.6	-6.7 (-51.9–25.1)
15 days post first dose to second dose	92/21,159	82/10,557	104.3	185.5	43.8 (24.3–58.3)
Second dose to <15 days post second dose	36/20,131	49/9,878	48.8	135.9	64.2 (44.9–76.7)
≥15 days post second dose	143/18,384	189/8,889	37.8	115.8	67.4 (59.4–73.8)
≥15 days post second dose to <6 months	134/18,384	186/8,889	37.9	121.3	68.7 (60.9–75.0)
6–12 months	9/2736	3/1,034	36.0	30.3	-18.6 (-335.7–67.7)
**Full analysis set, period to non-study COVID-19 vaccination (censoring at non-study COVID-19 vaccination only)†**					
Any time post first dose	571/21,583	408/10,797	47.7	120.9	59.7 (54.2–64.5)
<15 days post first dose	96/21,583	45/10,797	116.6	109.4	-6.6 (-51.8–25.2)
15 days post first dose to second dose	93/21,319	84/10,605	103.6	188.2	44.9 (26.0–59.0)
Second dose to <15 days post second dose	40/20,727	50/9,905	50.7	135.5	62.5 (43.2–75.2)
≥15 days post second dose	334/20,407	224/9,240	35.5	105.5	65.0 (58.5–70.5)
≥15 days post second dose to <6 months	222/20,407	210/9,240	32.7	114.2	70.3 (64.1–75.4)
6–12 months	112/16,209	14/2,015	42.7	49.1	7.7 (-61.1–47.1)
**FVAS population‡, period to non-study COVID-19 vaccination (censoring at non-study COVID-19 vaccination only)†**					
≥15 days post second dose	328/19,569	219/8,868	36.4	108.4	65.1 (58.5–70.6)
≥15 days post second dose to <6 months post first dose	218/19,569	205/8,868	33.6	117.0	70.2 (63.9–75.4)
≥6 months post first dose	110/15,514	14/1,896	43.7	52.2	11.1 (-55.2–49.1)

*per 1,000 person-years. †Regardless of serostatus at baseline. ‡Seronegative at baseline.
FVAS, fully vaccinated analysis set; RT-PCR, reverse transcriptase-PCR; VE, vaccine efficacy.

Table 10. Participant demographics and clinical characteristics, FVAS population for analysis of period to non-study COVID-19 vaccination.

	AZD1222 (*n* = 19,569)	Placebo (*n* = 8,868)
**Median age at screening, years (IQR)**	51.0 (38–63)	51.0 (38–63)
Aged ≥18–64 years, no. (%)	15,102 (77.2)	6,915 (78.0)
Aged ≥65 years, no. (%)	4,467 (22.8)	1,953 (22.0)
**Sex, no. (%)**		
Male	10,826 (55.3)	4,974 (56.1)
Female	8,743 (44.7)	3,894 (43.9)
**Race, no. (%)**		
White	15,594 (79.7)	7,022 (79.2)
Black or African American	1,532 (7.8)	718 (8.1)
Asian	850 (4.3)	370 (4.2)
American Indian or Alaska Native	764 (3.9)	376 (4.2)
Native Hawaiian or Other Pacific Islander	54 (0.3)	18 (0.2)
Multiple	453 (2.3)	210 (2.4)
Unknown or Not reported	322 (1.6)	154 (1.7)
**Hispanic or Latinx ethnicity, no. (%)**		
Yes	4,311 (22.0)	2,102 (23.7)
No	14,961 (76.5)	6,648 (75.0)
Unknown or not reported	297 (1.5)	118 (1.3)
**Country, no. (%)**		
United States	17,287 (88.3)	7,752 (87.4)
Chile	1,409 (7.2)	680 (7.7)
Peru	873 (4.5)	436 (4.9)
**Baseline COVID-19 comorbidities, no. (%)***		
Yes	11,713 (59.9)	5,357 (60.4)
No	7,854 (40.1)	3,510 (39.6)
Missing	2	1
**COVID-19 exposure risk category (OSHA), no. (%)**		
Very high	1,171 (6.0)	472 (5.4)
High	4,245 (21.9)	1,729 (19.7)
Medium	8,222 (42.4)	3,793 (43.3)
Lower	5,738 (29.6)	2,768 (31.6)
Missing	193	106
**Median dosing interval, days (IQR)†**	*n* = 19,569	*n* = 8,868
Overall	29.0 (29–30)	29.0 (29–30)
Participants randomized prior to clinical hold	*n* = 501<br>60.0 (57–63)	*n* = 248<br>59.0 (57–62)
Participants randomized after clinical hold	*n* = 19,068<br>29.0 (29–30)	*n* = 8,620<br>29.0 (29–30)

*COVID-19 comorbidities included chronic kidney disease, chronic obstructive pulmonary disease, lower immune health because of a solid organ transplant, history of obesity (BMI > 30), serious heart conditions, sickle cell disease, type 2 diabetes, asthma, dementia, cerebrovascular disease, cystic fibrosis, high blood pressure, liver disease, scarring in the lungs (pulmonary fibrosis), type 1 diabetes, thalassemia, history of smoking. †Because the trial was placed on clinical hold due to an event of transverse myelitis in a different study of AZD1222 (5), 775 participants received their second dose after a longer dosing interval (1).
BMI, body mass index; FVAS, fully vaccinated analysis set; IQR, interquartile range; OSHA, Occupational Safety and Health Administration.

Table 11. Sensitivity analyses for estimates of efficacy with AZD1222 versus placebo for the primary efficacy endpoint in the FVAS population for the period up to receipt of non-study COVID-19 vaccination.

Method	Estimate, % (95% CI)
**Primary analysis without imputation**	65.05 (58.46–70.60)
**Multiple imputation analysis that adjusts for single baseline covariate:**	
Age group at informed consent (18–64, ≥65 years)	58.75 (51.99–64.56)
Sex at birth (male, female)	59.40 (52.88–65.02)
Race (white, black or African American, Asian, other)	59.06 (52.39–64.79)
Ethnicity (Hispanic or Latinx, not Hispanic or Latinx, not reported, unknown)	59.16 (52.07–65.20)
Body mass index (<40, ≥40, missing)	59.19 (52.30–65.09)
Comorbidities (yes, no, missing)	59.10 (51.85–65.27)
OSHA risk category (very high, high, medium, lower exposure risk, missing)	59.51 (53.05–65.08)
Region (East North Central, East South Central, Middle Atlantic, Mountain, New England, Pacific, South America, South Atlantic, West North Central, West South Central)	58.93 (52.19–64.73)
**IPCW*†**	
IPCW using standardized weights†	61.7 (54.4–67.8)
IPCW (0.1%)†	53.4 (42.8–62.1)
IPCW (1%)†	55.2 (45.5–63.2)

*For the IPCW analyses, all results come from fitting a Cox Proportional Hazards (PH) model to the data (for the IPCW results this model is fitted to the counting process format data). All models use data only from participants with a complete set of covariate information as used in the previously described Cox PH model for right-censoring. All model results use all data up until a participant was right-censored, administratively censored, or had an infection event.
†Three versions of the IPCW method were used: IPCW using standardized weights and IPCW using unstandardized weights but truncating the weights at the 0.1% and 1% level – IPCW (0.1%) and IPCW (1%), respectively. The standardized weights are calculated as $w_s=p_{trt}/p$, where p is the cumulative probability of remaining uncensored (in each subinterval) estimated from the previously described Cox model for censoring and p_{trt} is the probability of remaining uncensored (in each subinterval) estimated from an unadjusted Cox model fitted to each treatment group separately. Truncation was defined as setting any weights below the X% percentile to be equal to the X% percentile and setting any weights above the (100-X)% percentile to be equal to the (100-X)% percentile. The CIs for the IPCW models are 95% intervals using robust standard error estimates to account for multiple participant records, although these ignore the uncertainty in estimating the weights.
FVAS, fully vaccinated analysis set; IPCW, inverse probability of censoring weighting; OSHA, Occupational Safety and Health Administration.

Table 12. Identification of VoC or VoI and VE against VoC/VoI with ≥5 events (FVAS population, double-blind period).

	AZD1222 (*n* = 17,617)		Placebo (*n* = 8,528)		
**VoC or VoI with ≥3 events**[†‡]	**no. (%)**	**IR***	**no. (%)**	**IR***	**VE, % (95% CI)**
**All variants (all cases sequenced)**[†]	81 (0.46)	22.53	115 (1.35)	74.22	69.7 (59.7–77.2)
**VoC**[‡]					
**Alpha**	9 (0.05)	2.50	11 (0.13)	7.10	64.8 (15.0–85.4)
**Gamma**	4 (0.02)	1.11	1 (0.01)	0.65	-72.4 (-1442.2–80.7)
**VoI**					
**Epsilon**	7 (0.04)	1.95	7 (0.08)	4.52	56.9 (–22.9–84.9)
**Lambda**	17 (0.10)	4.73	18 (0.21)	11.62	59.3 (21.0–79.0)
**All cases not sequenced**[§]	60 (0.34)	16.69	69 (0.81)	44.53	62.5 (47.0–73.5)

*Incidence rate per 1,000 person-years. Total follow-up was 3.60 and 1.55 x 1,000 person-years in the AZD1222 and placebo groups, respectively. [†]Includes 30 and 54 cases with A_1 lineage (IR: 8.34 and 34.85; VE: 76.1% [95% CI 62.6–84.7]), 9 and 21 with B.1 lineages (VE values not shown due to low case numbers with each individual variant), and 1 and 3 with R.1 lineage (IR: 0.28 and 1.94; VE: 85.6% [95% CI -38.4–98.5]). [‡]There were also 2 cases of Beta and 2 cases of Delta in the AZD1222 group; VE could not be estimated due to low case numbers. [§]'Not sequenced' includes 1 case in each group in which sequencing was attempted but the quantity was not sufficient.
FVAS, fully vaccinated analysis set; IR, incidence rate; VE, vaccine efficacy; VoC, variant of concern; VoI, variant of interest.

Figure 1. Efficacy of AZD1222 vs placebo for the prevention of COVID-19 and SARS-CoV-2 infection. (A) Cumulative incidence of SARS-CoV-2 RT-PCR-positive symptomatic illness occurring ≥15 days post second dose (time 0 = day 15 post second dose) in the FVAS population for the double-blind period of the study (AZD1222, n = 17,617; placebo, n = 8528). (B) Incidence of SARS-CoV-2 RT-PCR-positive symptomatic illness events and decrease in the at-risk population over time during the double-blind period. The at-risk population curves show the numbers of participants in the FVAS who have not been censored and are available for analysis at the corresponding time point. Cumulative incidence of (C) severe or critical symptomatic COVID-19 and (D) SARS-CoV-2 infection, as defined by seroconversion rate from negative at baseline to positive for SARS-CoV-2 nucleocapsid antibody at ≥15 days post second dose, regardless of symptoms, in the FVAS population for the double-blind period of the study (AZD1222, n = 17,617; placebo, n = 8528). For panels A, C and D, time to first event was from time of second dose administration, calculated as: (date of SARS-CoV-2-positive test) – (date of second dose of AZD1222 or placebo + 14 days) + 1. For censored participants, censoring time was from date of second dose of AZD1222 or placebo + 14 days to the last time observed before data cut-off (July 30, 2021). Cumulative incidences were estimated using the Kaplan–Meier method. Cumulative incidence curves were truncated at the point at which <10% of the starting population remained at risk.
CI, confidence interval; FVAS, fully vaccinated analysis set; IR, incidence rate per 1000 person-years.

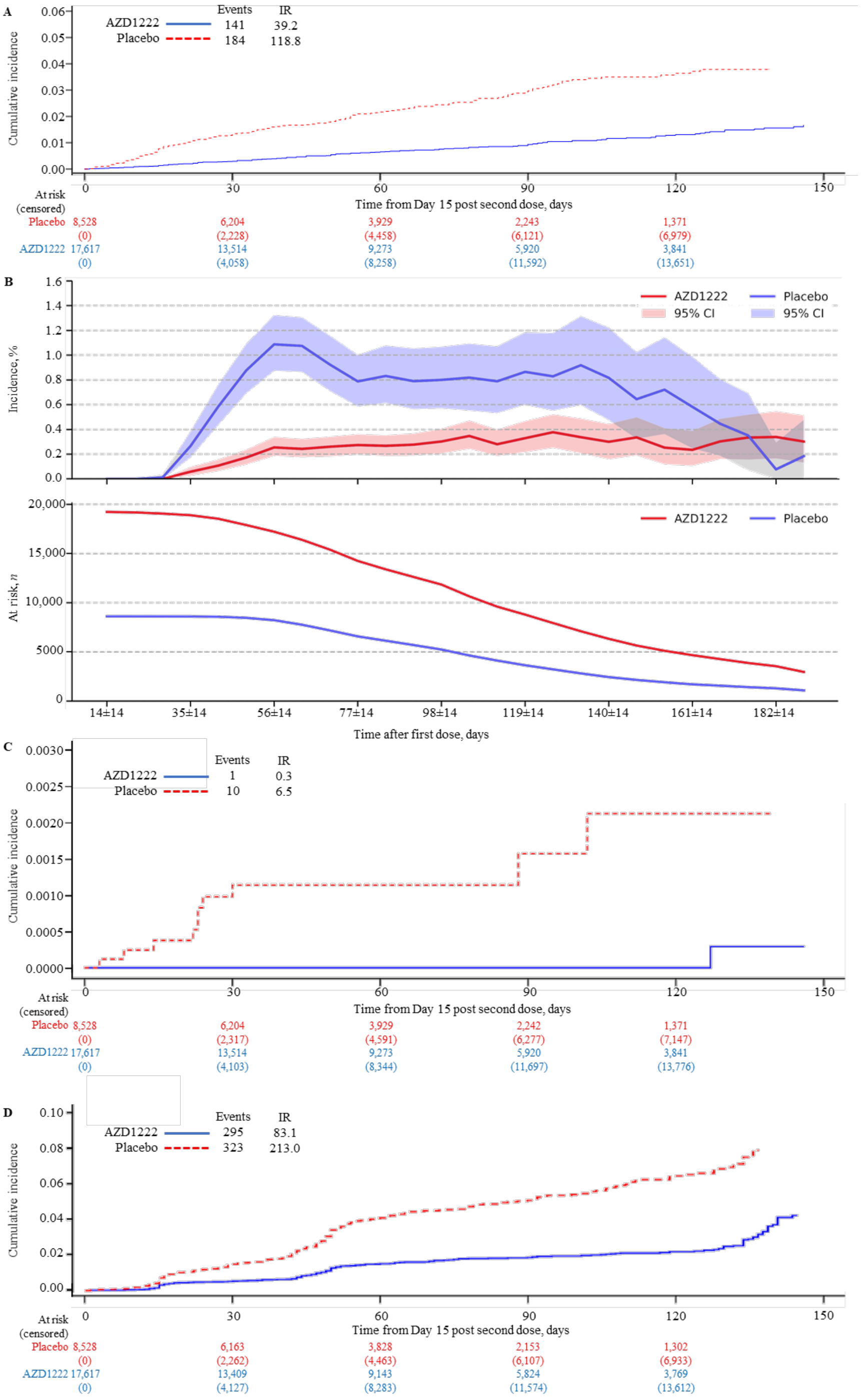

Figure 2. Kaplan–Meier analysis of non-study COVID-19 vaccination over time, regardless of unblinding. Occurrence of events of non-study COVID-19 vaccination stratified by arm and age group, OSHA exposure risk category or country. OSHA divides job tasks into four potential SARS-CoV-2 risk exposure levels dependent on aspects including the workplace environment, the feasibility of mask-wearing, the type of work activity, and the need for close contact with other people, including those with, or suspected to have, COVID-19 (https://www.osha.gov/coronavirus/hazards).

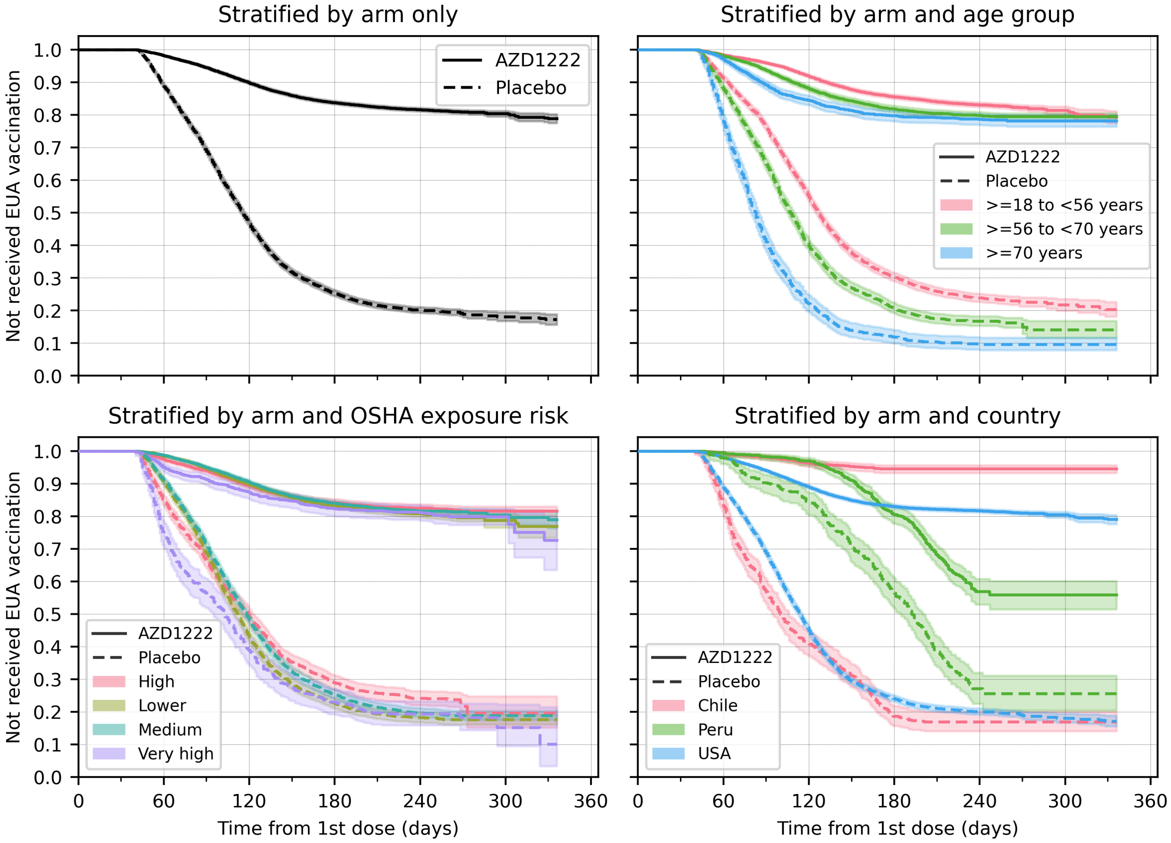

EUA, emergency use authorization; OSHA, Occupational Safety and Health Administration.

Figure 3. Neutralizing and spike-binding antibody responses over time in the placebo group.
Box and whisker plots showing (A) SARS-CoV-2 neutralizing antibody quantitation and (B, C) MSD spike antigen quantitation over time in the placebo group, with participants censored at (A, B) date of non-study COVID-19 vaccination, positive test for SARS-CoV-2 nucleocapsid antibodies or RT-PCR-positive SARS-CoV-2 symptomatic infection, or (C) date of unblinding, non-study COVID-19 vaccination, positive test for SARS-CoV-2 nucleocapsid antibodies, RT-PCR-positive SARS-CoV-2 symptomatic infection or last trial contact. Y-axes show 1/dilution for neutralizing antibodies or spike antigen titers in AU/mL – for conversion to the WHO International Standard, see supplemental methods (appendix, p 4). For panel A, boxes and whiskers do not appear in full due to the generally minimal level of antibodies and/or the small population size at later time points. The proportion of participants with a ≥4-fold increase from baseline in spike-binding antibodies, in the absence of a positive test for SARS-CoV-2 nucleocapsid antibodies, was 6.7% (64/957) across all post-baseline visits; this proportion was higher at later time points: 5/909 (0.6%) on day 15, 4/674 (0.6%) on day 29, 9/858 (1.0%) on day 43, 7/779 (0.9%) on day 57, 19/584 (3.3%) on day 90, and 30/139 (21.6%) on day 180.

A

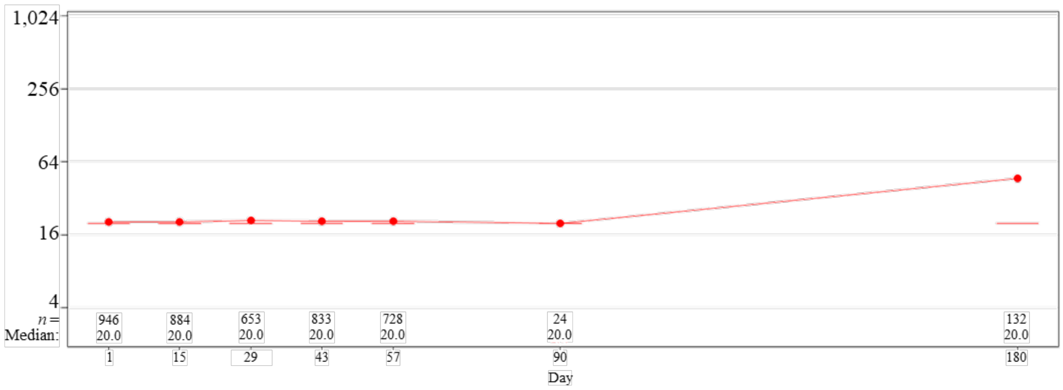

B

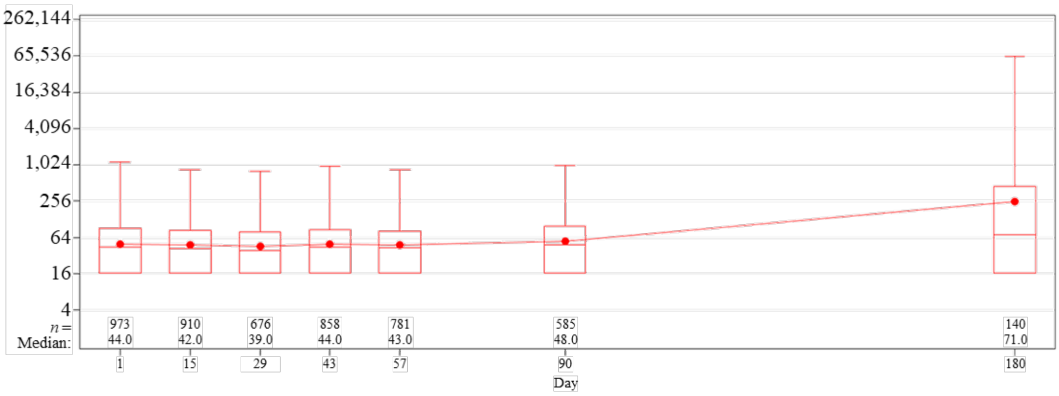

C

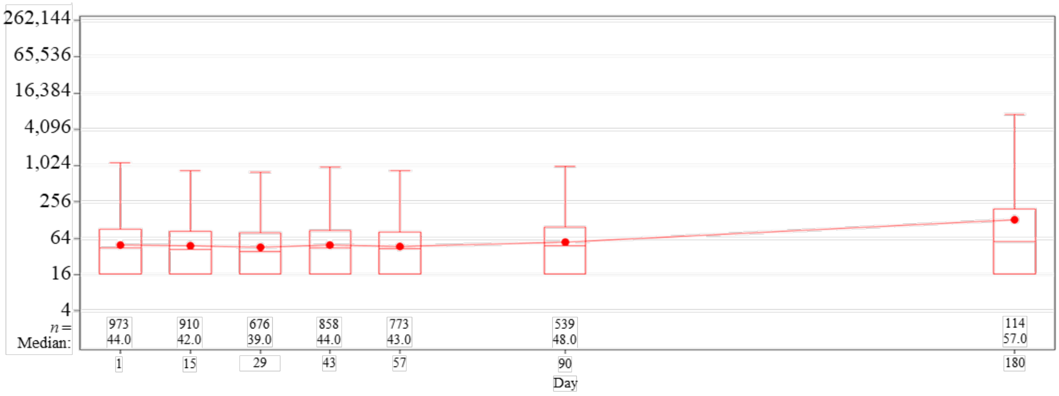

MSD, Meso Scale Diagnostics; RT-PCR, reverse transcriptase-PCR.

Figure 4. Neutralizing antibody responses over time by baseline comorbidities and serostatus.
Box and whisker plots showing SARS-CoV-2 neutralizing antibody quantitation over time by (A) comorbidities, AZD1222 group only, and (B) serostatus at baseline, both groups. Participants were censored at the earliest date of non-study COVID-19 vaccination, positive test for SARS-CoV-2 nucleocapsid antibodies or RT-PCR-positive SARS-CoV-2 symptomatic infection. Y-axes show 1/dilution for neutralizing antibodies – for conversion to the WHO International Standard, see supplemental methods (p 4).

A

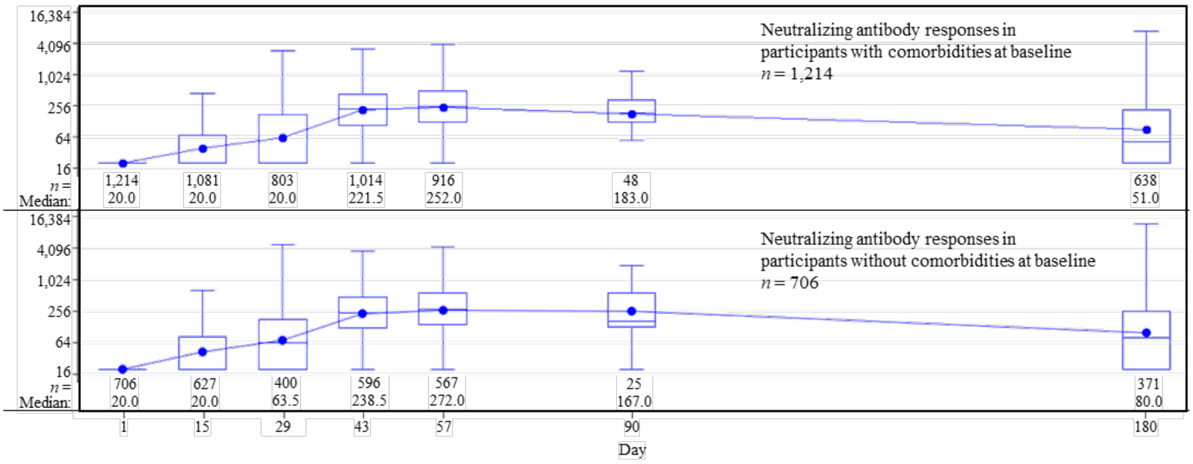

B

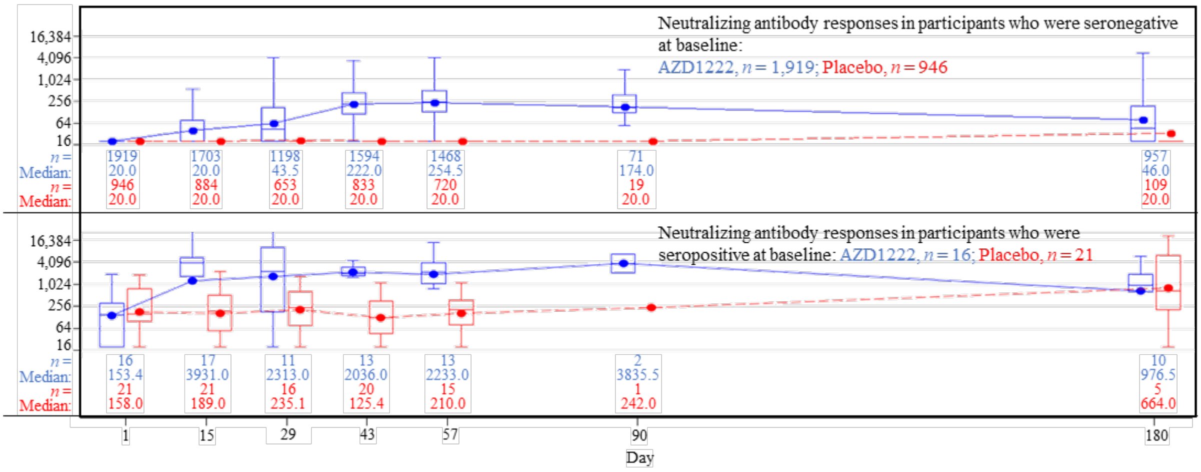

AU, arbitrary units; MSD, Meso Scale Diagnostics.

Figure 5. Correlation between anti-vector immune response after first dose and ability to boost after second dose.
Comparison of anti-vector neutralizing antibodies (horizontal axis) versus MSD spike antigen quantitation (vertical axis) on days 1, 29, 57, and 180; participants were censored at the time of non-study COVID-19 vaccination, regardless of unblinding, and were excluded if they tested positive for SARS-CoV-2 nucleocapsid antibody at any time. Y-axes show spike antigen titers in AU/mL – for conversion to the WHO International Standard, see supplemental methods (p 4).

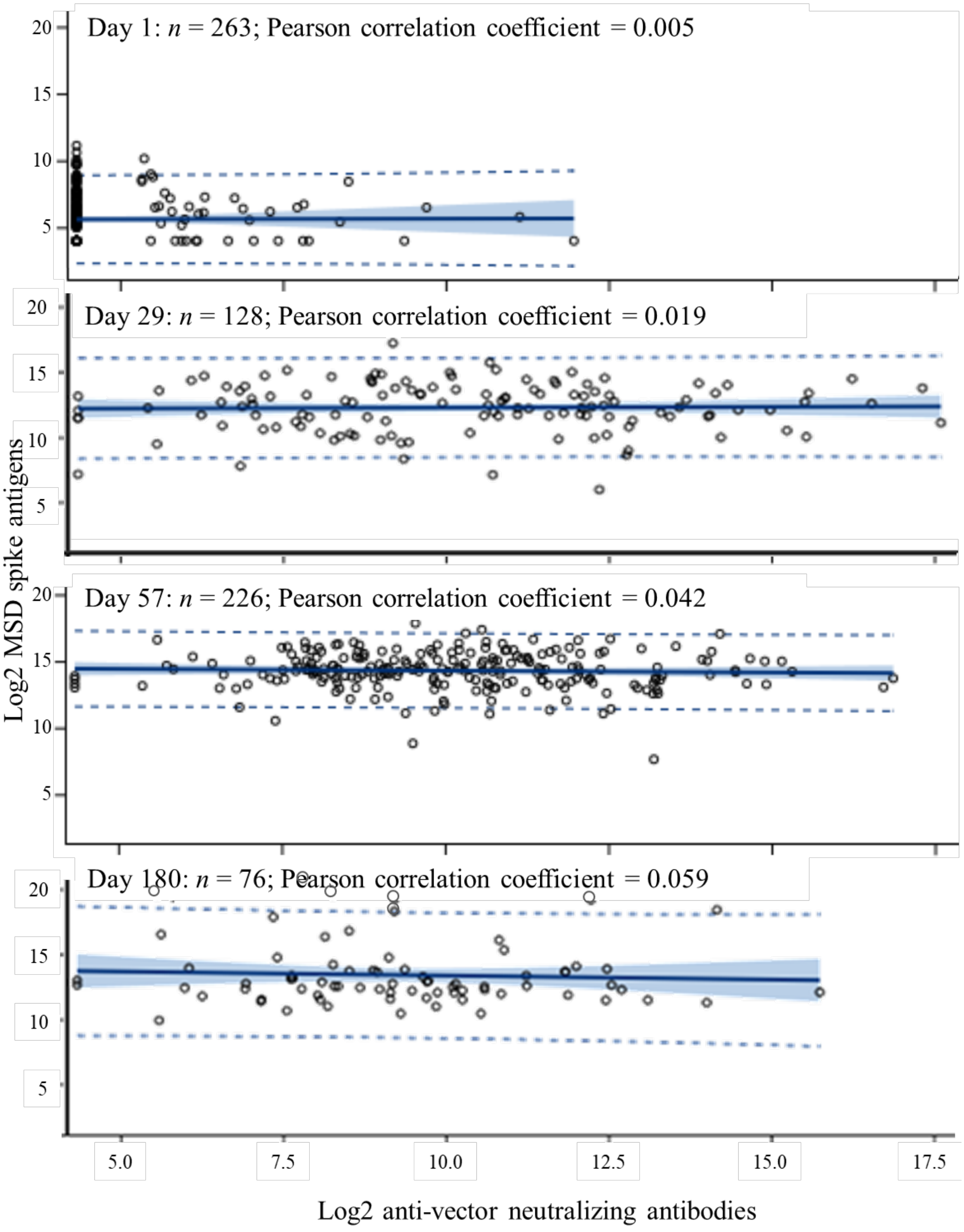

MSD, Meso Scale Diagnostics.

Supplemental References

1. Falsey AR, et al. Phase 3 safety and efficacy of AZD1222 (ChAdOx1 nCoV-19) Covid-19 vaccine. *N Engl J Med*. 2021;385(25):2348-2360.
2. O'Toole A, et al. Assignment of epidemiological lineages in an emerging pandemic using the pangolin tool. *Virus Evol*. 2021;7(2):veab064.
3. Rambaut A, et al. A dynamic nomenclature proposal for SARS-CoV-2 lineages to assist genomic epidemiology. *Nat Microbiol*. 2020;5(11):1403-1407.
4. Bedford T, et al. Updated Nextstrain SARS-CoV-2 clade naming strategy. Available at: https://nextstrain.org/blog/2021-01-06-updated-SARS-CoV-2-clade-naming. Last accessed 6 December 2021.
5. Voysey M, et al. Safety and efficacy of the ChAdOx1 nCoV-19 vaccine (AZD1222) against SARS-CoV-2: an interim analysis of four randomised controlled trials in Brazil, South Africa, and the UK. *Lancet*. 2021;397(10269):99-111.

Supplemental Acknowledgments

The following list of AZD1222 Clinical Study Group members is reproduced from the authorship of and appendix to the primary manuscript (Falsey AR, et al, N Engl J Med 2021) (1). Group members contributing to the findings reported in this manuscript are named as authors or listed in the Acknowledgments section of the main manuscript.

**Authorship of the primary manuscript (Falsey AR, et al, N Engl J Med 2021) (1)**	
**Name**	**Affiliation**
Ann R. Falsey	University of Rochester School of Medicine and Dentistry, Rochester, NY
Magdalena E. Sobieszczyk	Vagelos College of Physicians and Surgeons, New York-Presbyterian Columbia University Irving Medical Center, New York, NY
Ian Hirsch	AstraZeneca, Cambridge, UK
Stephanie Sproule	AstraZeneca, Gaithersburg, MD
Merlin L. Robb	Walter Reed Army Institute of Research, Silver Spring, MD
Lawrence Corey	University of Washington, Seattle, WA; Fred Hutchinson Cancer Research Center, Seattle, WA
Kathleen M. Neuzil	University of Maryland School of Medicine, Baltimore, MD
William Hahn	University of Washington, Seattle, WA; Fred Hutchinson Cancer Research Center, Seattle, WA
Julie Hunt	University of Maryland School of Medicine, Baltimore, MD
Mark J. Mulligan	New York University Vaccine Center, New York, NY
Charlene McEvoy	HealthPartners Institute, St Paul, MN
Edwin DeJesus	Orlando Immunology Center, Orlando, FL
Michael Hassman	Hassman Research Institute, Berlin, NJ
Susan J. Little	University of California San Diego, San Diego, CA
Barbara A. Pahud	Children's Mercy Kansas City, Kansas City, MO
Anna Durbin	Johns Hopkins Bloomberg School of Public Health, Baltimore, MD
Paul Pickrell	Tekton Research, Austin, TX
Eric S. Daar	Lundquist Institute at Harbor-UCLA Medical Center, Torrance, CA
Larry Bush	JEM Headlands Research, Palm Beach County, FL
Joel Solis	Centex Studies, McAllen, TX
Quito Osuna Carr	Medpharmics LLC, Albuquerque, NM
Temitope Oyedele	John H. Stroger Jr., Hospital of Cook County, Chicago, IL
Susan Buchbinder	Department of Public Health, San Francisco, CA
Jessica Cowden	Joint Program Executive Office for Chemical, Biological, Radiological and Nuclear Defense, Edgewood, MD
Sergio L. Vargas	Instituto de Ciencias Biomédicas, Facultad de Medicina Universidad de Chile, Santiago, Chile
Alfredo Guerreros Benavides	Clínica Internacional Sede Lima, Perú
Robert Call	Clinical Research Partners, Richmond, VA
Michael C. Keefer	University of Rochester School of Medicine and Dentistry, Rochester, NY
Beth D. Kirkpatrick	University of Vermont Larner College of Medicine and UVM Medical Center, Burlington, VT
John Pullman	Mercury Street Medical Group, Butte, MT
Tina Tong	National Institute of Allergy and Infectious Diseases, National Institutes of Health, Bethesda, MD
Margaret Brewinski Isaacs	National Institute of Allergy and Infectious Diseases, National Institutes of Health, Bethesda, MD
David Benkeser	Rollins School of Public Health at Emory University, Atlanta, GA
Holly E. Janes	Fred Hutchinson Cancer Research Center, Seattle, WA
Martha C. Nason	National Institute of Allergy and Infectious Diseases, National Institutes of Health, Bethesda, MD
Justin A. Green	AstraZeneca, Cambridge, UK
Elizabeth J. Kelly	AstraZeneca, Gaithersburg, MD
Jill Maaske	AstraZeneca, Gaithersburg, MD
Nancy Mueller	AstraZeneca, Gaithersburg, MD

Kathryn Shoemaker	AstraZeneca, Gaithersburg, MD
Therese Takas	AstraZeneca, Gaithersburg, MD
Richard P. Marshall	AstraZeneca, London, UK
Menelas N. Pangalos	AstraZeneca, Cambridge, UK
Tonya Villafana	AstraZeneca, Gaithersburg, MD
Antonio Gonzalez-Lopez	AstraZeneca, Gaithersburg, MD
**AZD1222 Clinical Study Group members listed in the appendix to the primary manuscript (Falsey AR, et al, N Engl J Med 2021) (1)**	
**Name**	**Affiliation**
Jeremy Ackermann	Clinical Trials of South Carolina, SC, USA
Mark S. Adams	Aalliance for multispecialty research LLC, Lexington, KY, USA
Nathan Alderson	The Pain Center of Arizona, Phoenix, AZ, USA
José O. Alemán	VA New York Harbor Healthcare System, New York, NY, USA
Mohamed S. Al-Ibrahim	Pharmaron SNBL Clinical Pharmacology Center, Baltimore, MD, USA
David R. Andes	University Hospital and UW Health Clinics, Madison, WI, USA
Jeb Andrews	Clinical Trials of America LLC, Monroe, LA, USA
Roberto C. Arduino	Clinical Research Unit- UTHSC-Houston, Houston, TX, USA
Martín Bäcker	NYU Langone Health Winthrop Hospital , Mineola, NY, USA
Diana Badillo	NYU Langone Health Winthrop Hospital , Mineola, NY, USA
Emma Bainbridge	Zuckerberg San Francisco General Hospital, UCSF Positive Health Program, San Francisco, CA, USA
Teresa A. Batteiger	Indiana University Health University Hospital, Indianapolis, IN, USA
Jose A. Bazan	The Ohio State University Wexner Medical Center Infectious Disease Clinic, Columbus, OH, USA
Roger J. Bedimo	Trinity Health and Wellness Centre CRS AIDS Arms Inc Prism health North Texas, TX, USA
Jorge A. Benitez	Naval Medical Center, Sandiego, CA, USA
Annette R. Bennett	Clinical Research Partners, LLC, Richmond, VA, USA
Catherine Berjohn	Naval Medical Center, San Diego, CA, USA
David I. Bernstein	Cincinnati Children's Hospital Medical Center, OH, USA
Kristin Bialobok	Oregon Health and Science University, Portland, OR, USA
Rebecca Boas	Bellevue Hospital, New York, NY, USA
Judith Brady	VA New York Harbor Healthcare System, New York, NY, USA
Angela R. Branche	Rochester Regional Health System, Rochester, NY, USA
Cynthia Brown	Indiana University Health University Hospital, Indianapolis, IN, USA
Catherine A. Bunce	University of Rochester School of Medicine and Dentistry, Rochester, NY, USA
Abram Burgher	The Pain Center of Arizona, Phoenix, AZ, USA
Wesley Campbell	Walter Reed National Military Medical Center at Bethesda, Bethesda, MD, USA
Ellie Carmody	NYU Langone Health, Brooklyn, NY, USA
Christopher Carpenter	William Beaumont Hospital, Royal Oak, MI, USA
Steven E. Carsons	NYU Lagone Health Winthrop Hospital , Mineola, NY, USA
Marvin Castellon	New York-Presbyterian Columbia University Irving Medical Center, New York, NY, USA
Mario Castro	University of Kansas Medical Center, Kansas City, KS, USA
Hannah Catan	New York-Presbyterian Columbia University Irving Medical Center, New York, NY, USA
Jennifer Chang	New York-Presbyterian Columbia University Irving Medical Center, New York, NY, USA
Mouna Gharib Chebib	West Virginia Research Institute PLLC, South Charleston, WV, USA
Corey M. Chen	West Virginia Research Institute PLLC, South Charleston, WV, USA
Margaret Cheng	Montefiore Medical Center/Albert Einstein, Bronx, NY, USA
Brian D. W. Chow	Tufts Medical Center, Boston, MA, USA
Annie Ciambruschini	Johns Hopkins Bloomberg School of Public Health, Baltimore, MD, USA
Joseph P. Connor	New York-Presbyterian Columbia University Irving Medical Center, New York, NY, USA
James H. Conway	University Hospital and UW Health Clinics, Madison, WI, USA
Maureen Cooney	William Beaumont Hospital, Royal Oak, MI, USA

Marcel Curlin	Oregon Health and Science University, Portland, OR, USA
Claudia De La Matta Rodriguez	NYU Langone Health, Brooklyn, NY, USA
Jon F. Dedon	University of Kansas Health System, Fairway, KS, USA
Emily Degan	ActivMed Practices and Research Inc, Portsmouth, NH, USA
Dinuli Delpachitra	NYU Langone Vaccine Center, New York, NY, USA
Michelle Dickey	Cincinnati Children's Hospital Medical Center, Cincinnati, OH, US
Craig Dietz	University of Kansas Health System, Fairway, KS, USA
Jennifer Lee Dong	Bellevue Hospital, New York, NY, USA
Brenda Dorcely	VA New York Harbor Healthcare System, New York, NY, USA
Matthew W. Doust	The Pain Center of Arizona, Phoenix, AZ, USA
Michael P. Dube	University of Southern California, Los Angeles, CA, USA
Carmel B. Dyer	Clinical Research Unit- UTHSC-Houston, Houston, TX, USA
Benjamin Eckhardt	NYU School of Medicine / Bellevue Hospital, New York, NY, USA
Edward Ellerbeck	University of Kansas Health System, Kansas City, KS, USA
Evan C. Ewers	Walter Reed National Military Medical Center, Bethesda, MD, USA
Amy Falk	Health Partnership Clinic, Olathe, KS, USA
Brittany Feijoo	Johns Hopkins Bloomberg School of Public Health, Baltimore, MD, USA
Uriel R. Felsen	Montefiore Medical Center/Albert Einstein, Bronx, NY, USA
Sabrina Felson	Department of Medicine at NYU Grossman School of Medicine, New York, NY, USA
Tom Fiel	CCT Research, Phoenix, AZ, USA
David Fitz-Patrick	East-West Medical Research Institute, Honolulu, HI, USA
Charles M. Fogarty	Spartanburg Medical Research, Spartanburg, SC, USA
Stacy Ford	Hassman Research Institute, Berlin, NJ, USA
Lina M. Forero	New York-Presbyterian Columbia University Irving Medical Center, New York, NY, USA
Elizabeth Formentini	Biomedical Advanced Research and Development Authority, Washington, DC, USA
Doris Franco-Vitteri	Clinica Ricardo Palma, Av. Javier Prado Este 1066, Lima, Peru
Robert W. Frenck Jr.	Cincinnati Children's Hospital Medical Center, Cincinnati, OH, USA
Elie Gharib	West Virginia Research Institute PLLC, South Charleston, WV, USA
Suzanne Gharib	West Virginia Research Institute PLLC, South Charleston, WV, USA
Rola Gharib Rucker	West Virginia Research Institute PLLC, South Charleston, WV, USA
James N. Goldenberg	JEM Headlands Research, Palm Beach County, FL, USA
Luis H. González	Instituto de Ciencias Biomédicas, Facultad de Medicina Universidad de Chile, Santiago, Chile
Brett Gray	New York-Presbyterian Columbia University Irving Medical Center, New York, NY, USA
Rusty Greene	VA New York Harbor Healthcare System, New York, NY, USA
Robert M. Grossberg	Montefiore Medical Center/Albert Einstein, Bronx, NY, USA
Juan Vicente Guanira Carranza	Clinica Ricardo Palma, Lima, Peru
Clint C. Guillory	Clinical Trials of America LLC, Monroe, LA, USA
Shauna H Gunaratne	New York-Presbyterian Columbia University Irving Medical Center, New York, NY, USA
David Halpert	JEM Headlands Research, Palm Beach County, FL, USA
Holli Hamilton	The Pain Center of Arizona, Phoenix, AZ, USA
William R. Hartman	University Hospital and UW Health Clinics, Madison, WI, USA
Timothy J. Hatlen	Lundquist Institute at Harbor-UCLA Medical Center, Torrance, CA, USA
Gary F. Headden	Medical University of South Carolina, Charleston, SC, USA
Sheryl L. Henderson	University of Wisconsin School of Medicine and Public Health, Madison, WI, USA
Ramin Herati	NYU Langone Vaccine Center, New York, NY, USA
Laura Hernandez Guarin	Rush University Medical Center, Section of Infectious Diseases, Chicago, IL, USA
Janette Hernandez Torres	NYU Langone Health, Brooklyn, NY, USA
Robin Hilder	Ronald Reagan UCLA Medical Center, Los Angeles, CA, USA

Ken Ho	University of Pittsburgh Medical Center, Pittsburgh, PA, USA
Leila Hojat	Case Clinical Research, Cleveland, OH, USA
Sybil G. Hosek	John H. Stroger Jr., Hospital of Cook County, Chicago, IL, USA
Jeffrey M. Jacobson	Case Clinical Research, Cleveland, OH, USA
Melanie Jay	VA New York Harbor Healthcare System, New York, NY, USA
Diane H. Johnson	NYU Langone Health Winthrop Hospital , Mineola, NY, USA
Kathleen S. Jones	The Iowa Clinic, West De Moines, IO, USA
Edward C. Jones-López	University of Southern California, Los Angeles, CA, USA
Jessica E. Justman	Bronx Prevention Center, New York, NY, USA
Scott Kahney	Javara Inc, The Woodlands, TX, USA
Lois Katz	VA New York Harbor Healthcare System, New York, NY, USA
Melinda Katz	Bellevue Hospital, New York, NY, USA
Daniel Kaul	Michigan Medicine, Ann Arbor, MI, USA
Ashley Kennedy	ActivMed Practices and Research Inc, Portsmouth, NH, USA
Jennifer Knishinsky	Bellevue Hospital, New York, NY, USA
Laura Kogelman	Tufts Medical Center, Boston, MA, USA
Susan L. Koletar	The Ohio State University Wexner Medical Center Infectious Disease Clinic, Columbus, OH, USA
Angelica Kottkamp	NYU Langone Vaccine Center, New York, NY, USA
Maryrose Laguio-Vila	Rochester Regional Health System, Rochester, NY, USA
Raphael J. Landovitz	Ronald Reagan UCLA Medical Center, Los Angeles, CA, USA
Jessica L. Lee	Clinical Research Unit- UTHSC-Houston, Houston, TX, USA
Albert Liu	Department of Public Health, San Francisco, CA, USA
Eneyda Giuvanela Llerena Zegarra	Clinica Internacional, Lima, Peru
Anna S. Lok	Michigan Medicine, An Arbor, MI, USA
James Lovell	The Iowa Clinic, West De Moines, IO, USA
Ronald Lubelchek	John H. Stroger Jr., Hospital of Cook County, Chicago, IL, USA
John Lucaj	Montefiore Medical Center/Albert Einstein, Bronx, NY, USA
Gary Luckasen	Medical Center of the Rockies, Loveland, CO, USA
Annie Luetkemeyer	Zuckerberg San Francisco General Hospital, UCSF Positive Health Program, San Francisco, CA, USA
Njira Lucia Lugogo	Michigan Medicine, Ann Arbor, MI, USA
Janine Maenza	Fred Hutchinson Cancer Research Center/University of Washington Vaccine Trials Unit, Seattle, WA, USA
Carlos Malvestutto	The Ohio State University Wexner Medical Center Infectious Disease Clinic, Columbus, OH, USA
Robin Mason	Biomedical Advanced Research and Development Authority, Washington, DC, USA
Paul Matherne	MedPharmics LLC, Gulfport, MS, USA
Monica Mauri	Alliance for Multispecialty Research LLC, Coral Gables, FL, USA
Ryan C. Maves	Naval Medical Center, San Diego, CA , USA
Kenneth H. Mayer	Fenway Health, Boston, MA, USA
Michael J. McCartney	ActivMed Practices and Research Inc, Portsmouth, NH, USA
Margaret E. McCort	Montefiore Medical Center/Albert Einstein, Bronx, NY, USA
M. Juliana McElrath	Fred Hutchinson Cancer Research Center/University of Washington Vaccine Trials Unit, Seattle, WA, USA
Alexander McMeeking	Department of Medicine at NYU Grossman School of Medicine, New York, NY, USA
Meredith McNairy	New York-Presbyterian Columbia University Irving Medical Center, New York, NY, USA
Fernando L. Merino	University of Kansas Health System, Fairway, KS, USA
Eric A. Meyerowitz	Montefiore Medical Center/Albert Einstein, Bronx, NY, USA
Carol L. Mitchell	University of Pittsburgh Medical Center, Pittsburgh, PA, USA
Cynthia L. Monaco	University of Rochester School of Medicine and Dentistry, Rochester, NY, USA
Sauda Muhammad	New York Blood Center, New York, NY, USA
Sigridh Muñoz-Gómez	NYU Lagone Health Winthrop Hospital, Mineola, NY, USA
Sonal Munsiff	University of Rochester School of Medicine and Dentistry, Rochester, NY, USA

Paul Nee	Danbury Hospital, Danbury, CT, USA
Nicole L. Nollen	University of Kansas School of Medicine, Kansas City, KS, USA
Asif Noor	NYU Langone Health Winthrop Hospital , Mineola, NY, USA
Claudio Nuñez Lagos	Instituto de Ciencias Biomédicas, Facultad de Medicina Universidad de Chile, Santiago, Chile
Jason F. Okulicz	Wilford Hall Medical Center, LBSA Lackland, TX, USA
Patrick A. Oliver	Clinical Research Partners, LLC, Richmond, VA, USA
Mary Olson	Department of Medicine, NYU Hepatology Associates, NYU Langone Health, New York, NY, USA
Jessica Ortega	Instituto de Ciencias Biomédicas, Facultad de Medicina Universidad de Chile, Santiago, Chile
Steven Palmer	New York-Presbyterian Columbia University Irving Medical Center, New York, NY, USA
Lalitha Parameswaran	NYU Langone Health Winthrop Hospital , Mineola, NY, USA
Purvi Parikh	NYU Langone Vaccine Center, New York, NY, USA
Susan Parker	Cincinnati Children's Hospital Medical Center, Cincinnati, OH, USA
Reza Parungao	Bellevue Hospital, New York, NY, USA
Juana Rosa Pavie	Centro Respiratorio Integral, Quillota, Chile
Rebecca Pellett Madan	NYU Langone Health, Brooklyn, NY, USA
Jay Pendse	NYU Langone Vaccine Center, New York, NY, USA
Henry Peralta	New York-Presbyterian Columbia University Irving Medical Center, New York, NY, USA
Jennifer Petts	The Iowa Clinic, West De Moines, IO, USA
Kristen K. Pierce	University of Vermont Larner College of Medicine and UVM Medical Center, Burlington, VT, USA
Terry L. Poling	Alliance for Multispecialty Research LLC, Wichita, KS, USA
E. Javier Pretell Alva	Policlinico especializado en Neurologia, Callao, Peru
Lawrence J. Purpura	New York-Presbyterian Columbia University Irving Medical Center, New York, NY, USA
Vanessa Raabe	NYU Langone Vaccine Center, New York, NY, USA
Sergio E. Recuenco	Policlinico especializado en Neurologia, Callao, Peru
Tamara Richards	Alliance for Multispecialty Research LLC, Coral Gables, FL, USA
Sharon A. Riddler	University of Pittsburgh Medical Center, Pittsburgh, PA, USA
Barbara Rizzardi	Advanced Clinical Research, West Jordan, UT, USA
Rachel Rokser	Johns Hopkins Bloomberg School of Public Health, Baltimore, MD, USA
Charlotte-Paige Rolle	Orlando Immunology Center, Orlando, FL, USA
Adam Rosen	Alliance for Multispecialty Research LLC, Coral Gables, FL, USA
Jeffrey Rosen	Alliance for Multispecialty Research LLC, Coral Gables, FL, USA
Lena Rydberg Freese	The Iowa Clinic, West De Moines, IO, USA
María E. Santolaya	Hospital Luis Calvo Mackenna, Santiago, Chile
Daniel Sartori	NYU Grossman School of Medicine, NYU Langone Hospital – Brooklyn, New York, NY, USA
Linda Moore Schipani	Clinical Research Associates Inc., Nashville, TN, USA
Adam Schwartz	Bellevue Hospital, New York, NY, USA
Tiffany Schwasinger-Schmidt	University of Kansas Medical Center, Kansas City, KS, USA
Hyman Scott	Department of Public Health, San Francisco, CA, USA
Beverly E. Sha	Rush University Medical Center, Section of Infectious Diseases, Chicago, IL, USA
Shivanjali Shankaran	Rush University Medical Center, Section of Infectious Diseases, Chicago, IL, USA
Adrienne E. Shapiro	Fred Hutchinson Cancer Research Center/University of Washington Vaccine Trials Unit, Seattle, WA, USA
Stephan C. Sharp	Clinical Research Associates Inc., Nashville, TN, USA
Bo Shopsin	NYU Langone Vaccine Center, New York, NY, USA
Matthew D. Sims	William Beaumont Hospital, Royal Oak, MI, USA
Stephanie Skipper	Orlando Immunology Center, Orlando, FL, USA
Derek M. Smith	Wilford Hall Medical Center, LBSA Lackland, TX, USA

Michael J. Smith	Duke University Medical Center, Durham, NC, USA
William B. Smith	Alliance for Multispecialty Research LLC, Knoxville, TN, USA
M. Mahdee Sobhanie	The Ohio State University Wexner Medical Center Infectious Disease Clinic, Columbus, OH, USA
Brit Sovic	New York-Presbyterian Columbia University Irving Medical Center, New York, NY, USA
Stephanie Sterling	NYU Langone Health, Brooklyn, NY, USA
Robert Striker	University Hospital and UW Health Clinics, Madison, WI, USA
Karla Beatriz Tafur Bances	Clinica Ricardo Palma, Lima, Peru
Kawsar R. Talaat	Johns Hopkins Bloomberg School of Public Health, Baltimore, MD, USA
Edward M. Tavel Jr.	Clinical Trials of South Carolina, SC, USA
Deborah A Theodore	New York Blood Center, New York, NY, USA
Hong Van Tieu	New York Blood Center, New York, NY, USA
Christian Tomaszewski	El Centro Regional Medical Center, El Centro, CA, USA
Ryan Tomlinson	The Iowa Clinic, West De Moines, IO, USA
Juan P. Torres	Hospital Luis Calvo Mackenna, Santiago, Chile
Julian A. Torres	Montefiore medical Center/Albert Einstein, Bronx, NY, USA
John Jay Treanor	Tunnell Government Services, Contractor Supporting Biomedical Advanced Research and Development Authority, Washington, DC, USA
Sade Tukuru	New York-Presbyterian Columbia University Irving Medical Center, New York, NY, USA
Mark Turner	Advanced Clinical Research, Meridian, ID, USA
Robert J. Ulrich	VA New York Harbor Healthcare System, New York, NY, USA
Gregory C. Utz	Naval Medical Center, San Diego, CA, USA
Veronica Viar	Zuckerberg San Francisco General Hospital, UCSF Positive Health Program, San Francisco, CA, USA
Roberto A. Viau Colindres	Tufts Medical Center, Boston, MA, USA
Edward E. Walsh	University of Rochester School of Medicine and Dentistry, Rochester, NY, USA
Mary Claire Walsh	University of Vermont Larner College of Medicine and UVM Medical Center, Burlington, VT, USA
Emmanuel B. Walter	Duke University Medical Center, Durham, NC, USA
Jessica L. Weidler	New York-Presbyterian Columbia University Irving Medical Center, New York, NY, USA
Yi H. Wu	New York-Presbyterian Columbia University Irving Medical Center, New York, NY, USA
Kinara S. Yang	New York-Presbyterian Columbia University Irving Medical Center, New York, NY, USA
Juan Luis Yrivarren Giorza	Clinica Ricardo Palma, Av. Javier Prado Este 1066, Lima, Peru
Arthur L. Zemanek	Rochester Regional Health System, Rochester, NY, USA
Kevin Zhang	VA New York Harbor Healthcare System, New York, NY, USA
Barry S. Zingman	Montefiore Medical Center/Albert Einstein, Bronx, NY, USA
