# Supplementary material for: Durability of protection and immunogenicity of AZD1222 (ChAdOx1 nCoV-19) COVID-19 vaccine over 6 months
Source: J Clin Invest. 2022 Sep 15;132(18):e160565. doi: 10.1172/JCI160565 (PMC9479753; doi:10.1172/JCI160565)
Supplement: Supplemental data 2 [file jci-132-160565-s150.pdf]

Durability of protection and immunogenicity of AZD1222 (ChAdOx1 nCoV-19) COVID-19 vaccine over 6 months

Magdalena E. Sobieszczyk,* Jill Maaske,* Ann R. Falsey,* Stephanie Sproule, Merlin L. Robb, Robert W. Frenck Jr, Hong-Van Tieu, Kenneth H. Mayer, Lawrence Corey, Kathleen M. Neuzil, Tina Tong, Margaret Brewinski Isaacs, Holly Janes, Himanshu Bansal, Lindsay M. Edwards, Justin A. Green, Elizabeth J. Kelly, Kathryn Shoemaker, Therese Takas, Tom White, Prakash Bhuyan, Tonya Villafana, Ian Hirsch, on behalf of the AstraZeneca AZD1222 Clinical Study Group†
*Contributed equally
†AZD1222 Clinical Study Group members are listed in the appendix to the primary manuscript (Falsey AR, et al, N Engl J Med 2021).[1] Additional group members contributing to the findings reported in this manuscript are named in the Acknowledgments section of the main manuscript.

1. Falsey AR, Sobieszczyk ME, Hirsch I, et al. Phase 3 safety and efficacy of AZD1222 (ChAdOx1 nCoV-19) Covid-19 vaccine. *N Engl J Med* 2021; **385**(25): 2348-60.

Supplementary Appendix 2

Table of contents

Content	Page
**Final Clinical Study Protocol Amendment 6**	2
**Final Statistical Analysis Plan Version 3.0**	95

Clinical Study Protocol	
Study Intervention	AZD1222
Study Code	D8110C00001
Version	Amendment 6
Date	19 February 2021

TITLE PAGE

A Phase III Randomized, Double-blind, Placebo-controlled Multicenter Study in Adults to Determine the Safety, Efficacy, and Immunogenicity of AZD1222, a Non-replicating ChAdOx1 Vector Vaccine, for the Prevention of COVID-19

Sponsor Name: **AstraZeneca AB**

Legal Registered Address: [redacted] Södertälje, Sweden

Regulatory Agency Identifier Number(s): **IND number 23522**
NCT04516746
EudraCT number 2020-005226-28

This Clinical Study Protocol has been subject to a peer review according to AstraZeneca Standard procedures. The Clinical Study Protocol is publicly registered and the results are disclosed and/or published according to the AstraZeneca Global Policy on Bioethics and in compliance with prevailing laws and regulations.

Protocol Number: D8110C00001

Amendment Number: 6

Study Intervention: AZD1222

Study Phase: III

Short Title: Phase III Double-blind, Placebo-controlled Study of AZD1222 for the Prevention of COVID-19 in Adults

Study Physician Name and Contact Information will be provided separately

USA Coordinating Investigators:

[redacted]
Rochester, NY

[redacted]
New York, NY

PROTOCOL AMENDMENT SUMMARY OF CHANGES TABLE

DOCUMENT HISTORY	
**Document**	**Date**
Amendment 6	19 February 2021
Amendment 5	18 January 2021
Amendment 4	14 December 2020
Amendment 3	26 October 2020
Amendment 2	17 September 2020
Amendment 1	10 August 2020
Original Protocol	15 July 2020

Amendment 6 (19 February 2021)

This amendment is considered to be substantial based on the criteria set forth in Article 10(a) of Directive 2001/20/EC of the European Parliament and the Council of the European Union.

Overall Rationale for the Amendment:

The principal reason for this amendment was to remove the extended dosing interval assessment and to remove adolescents from the study population that were added to Amendment 5. (Note: Amendment 5 was not implemented.)

Section # and Name	Description of Change	Brief Rationale	Substantial/ Non-substantial
Title Page,<br>1.1 Synopsis	Removed “and Adolescents” from the title	Given removal of adolescents from the study population	Substantial
1.1 Synopsis,<br>2.1 Study Rationale	Removed text from the study rationale regarding adolescents		
1.1 Synopsis	Reduced the number of study sites from 300 to 150	To more realistically align with the actual number of sites	Non-substantial
1.1 Synopsis,<br>3 Objective and Endpoints,<br>Appendix F (Table 15)	Upgraded exploratory endpoint 5 to secondary endpoint 9	To gain information on efficacy following a single dose of AZD1222	Substantial
1.1 Synopsis,<br>1.2 Schema,<br>1.3 Schedule of Activities<br>3 Objectives and Endpoints,<br>4.1 Overall Design,<br>4.2 Scientific Rationale for Study Design,<br>4.3 Justification for Dose,<br>4.4 End of Study Definition,<br>5 Study Population,<br>6.1.2 Dosing Instructions,<br>6.3 Measures to Minimize Bias: Randomization and Blinding,<br>7.1 Discontinuation of Study Intervention<br>8 Study Assessments	Removed Appendix G Part 2 and any cross-references to Appendix G Part 2	Given removal of Part 2 from the protocol	Substantial
	Removed the “Part 1” label from the main study and corresponding substudy and immunogenicity cohort		Substantial

Section # and Name	Description of Change	Brief Rationale	Substantial/ Non-substantial
and Procedures,<br>9 Statistical Considerations,<br>Appendix G Part 2			
1.1 Synopsis,<br>Appendix F<br>(Table 15)	Removed specification that primary objective 3 and secondary objectives 7-8 apply to both Part 1 and Part 2	Given removal of Part 2 from the protocol	Substantial
3 Objectives and Endpoints	Removed specification that primary objective 3, secondary objectives 7-8, and exploratory objectives 5-13 apply to both Part 1 and Part 2<br>Removed footnote d from Table 5 that indicated exploratory endpoint 5 is an exploratory endpoint for Part 1, but a secondary endpoint for Part 2	Given removal of Part 2 from the protocol	Substantial
4.2.2 Rationale for Study Endpoints,<br>8.3.9 Adverse Events of Special Interest,<br>11 References,<br>Appendix E	Removed the Serazin et al 2020 and Vogel et al 2020 references	Given removal of the multisystem inflammatory syndrome in children and adults (MIS-C/A) and acute respiratory distress syndrome (ARDS) terms from Appendix E, Table 13	Non-substantial
8.1.2.1 SARS-CoV-2 Testing and Other Virology Assessments	Added text to explain that genotypic evaluation of SARS-CoV-2 is planned at Days 1 and 14 illness visits but may be performed at Days 21 and 28 illness visits based upon logistical constraints, safety signals, or emerging data on efficacy of AZD1222 against viral variants	Clarification	Non-substantial
9.4.2.2 Secondary Endpoints,<br>Appendix F (Table 15)	Revised the list of secondary endpoints to align with the Synopsis and Section 3 of the protocol	Consistency	Non-substantial

Section # and Name	Description of Change	Brief Rationale	Substantial/ Non-substantial
A5 Committee Structure	Revised this section	To align with the current version of the statistical analysis plan	Non-substantial
Appendix E (Table 13)	Removed reference to MIS-C/A and ARDS	Given removal of the adolescent population	Non-substantial
Appendix G Part 2	Removed this appendix	Given removal of Part 2 from the protocol	Substantial
Throughout	Minor editorial revisions	Minor, therefore, were not summarized	Non-substantial

TABLE OF CONTENTS

TITLE PAGE ... 1
PROTOCOL AMENDMENT SUMMARY OF CHANGES TABLE ... 3
TABLE OF CONTENTS ... 7
1 PROTOCOL SUMMARY ... 12
1.1 Synopsis ... 12
1.2 Schema ... 18
1.3 Schedule of Activities ... 18
2 INTRODUCTION ... 27
2.1 Study Rationale ... 27
2.2 Background ... 27
2.3 Benefit/Risk Assessment ... 29
2.3.1 Risk Assessment ... 30
2.3.2 Benefit Assessment ... 30
2.3.3 Overall Benefit: Risk Conclusion ... 30
3 OBJECTIVES AND ENDPOINTS ... 30
4 STUDY DESIGN ... 34
4.1 Overall Design ... 34
4.2 Scientific Rationale for Study Design ... 36
4.2.1 Rationale for Study Design and Participant Population ... 36
4.2.2 Rationale for Study Endpoints ... 37
4.3 Justification for Dose ... 37
4.4 End of Study Definition ... 38
5 STUDY POPULATION ... 39
5.1 Inclusion Criteria ... 39
5.2 Exclusion Criteria ... 41
5.3 Lifestyle Considerations ... 42
5.4 Screen Failures ... 42
6 STUDY INTERVENTION ... 43
6.1 Study Interventions Administered ... 43
6.1.1 Investigational Products ... 43
6.1.2 Dosing Instructions ... 44
6.2 Preparation/Handling/Storage/Accountability ... 44
6.2.1 Dose Preparation and Administration ... 45
6.2.1.1 AZD1222 ... 45
6.2.1.2 Placebo ... 45
6.3 Measures to Minimize Bias: Randomization and Blinding ... 45
6.3.1 Randomization ... 45

6.3.2 Blinding ... 46
6.3.3 Procedures for Unblinding ... 46
6.4 Study Intervention Compliance ... 47
6.5 Concomitant Therapy ... 47
6.5.1 Permitted Concomitant Medications ... 47
6.5.2 Prohibited Concomitant Medications ... 47
6.6 Dose Modification ... 48
6.7 Intervention After the End of the Study ... 48
7 DISCONTINUATION OF STUDY INTERVENTION AND PARTICIPANT DISCONTINUATION/WITHDRAWAL ... 49
7.1 Discontinuation of Study Intervention ... 49
7.1.1 Study Suspension or Termination ... 49
7.2 Participant Withdrawal from the Study ... 50
7.3 Lost to Follow-up ... 50
8 STUDY ASSESSMENTS AND PROCEDURES ... 50
8.1 Efficacy Assessments ... 51
8.1.1 Monitoring COVID-19 Symptoms ... 51
8.1.2 Illness Visits ... 52
8.1.2.1 SARS-CoV-2 Testing and Other Virology Assessments ... 53
8.1.2.2 Digital Health Device ... 53
8.1.2.3 Illness e-Diary ... 54
8.1.3 Severe COVID-19 ... 55
8.1.4 Medical Notes Review ... 55
8.1.5 Monitoring for Asymptomatic Infection ... 55
8.2 Safety Assessments ... 55
8.2.1 Physical Examinations ... 56
8.2.2 Vital Signs ... 56
8.2.3 Clinical Laboratory Assessments ... 56
8.3 Adverse Events and Serious Adverse Events ... 57
8.3.1 Time Period and Frequency for Collecting AE and SAE Information ... 57
8.3.2 Follow-up of AEs and SAEs ... 57
8.3.3 Causality Collection ... 58
8.3.4 Adverse Events Based on Signs and Symptoms ... 59
8.3.5 Adverse Events Based on Examinations and Tests ... 59
8.3.6 Hy's Law ... 59
8.3.7 Solicited Adverse Events (Only for Substudy) ... 60
8.3.8 Medically Attended Adverse Events ... 61
8.3.9 Adverse Events of Special Interest ... 61
8.3.9.1 Potential Neurological AESIs ... 61
8.3.10 Reporting of Serious Adverse Events ... 63
8.3.11 Pregnancy ... 63
8.3.11.1 Maternal Exposure ... 63
8.3.12 Medication Error ... 64

8.3.13 Medical Device Deficiencies ... 64
8.4 Overdose ... 65
8.5 Human Biological Samples ... 65
8.5.1 Pharmacokinetics ... 65
8.5.2 Immunogenicity Assessments ... 66
8.5.2.1 SARS-CoV-2 Serology Assessments ... 66
8.5.2.2 SARS-CoV-2 Neutralizing Antibody Assessments ... 66
8.5.2.3 Assessment of Mucosal Responses ... 66
8.5.2.4 Assessment of Cell-mediated Immune Responses ... 66
8.5.2.5 Additional Serum Immunogenicity ... 67
8.5.3 Pharmacodynamics ... 67
8.6 Human Biological Sample Biomarkers ... 67
8.6.1 Collection of Mandatory Samples for Biomarker Analysis ... 67
8.6.1.1 Virologic Assessments ... 67
8.6.1.2 Assessment of Viral Shedding ... 68
8.6.2 Other Study-related Biomarker Research ... 68
8.7 Optional Genomics Initiative Sample ... 68
8.8 Medical Resource Utilization and Health Economics ... 68
9 STATISTICAL CONSIDERATIONS ... 68
9.1 Statistical Hypotheses ... 68
9.2 Sample Size Determination ... 69
9.3 Populations for Analyses ... 70
9.4 Statistical Analyses ... 71
9.4.1 General Considerations ... 72
9.4.2 Efficacy ... 73
9.4.2.1 Primary Endpoint ... 73
9.4.2.2 Secondary Endpoints ... 75
9.4.2.3 Exploratory Endpoints ... 77
9.4.3 Safety ... 77
9.4.3.1 Primary Endpoints ... 77
9.4.3.2 Other Safety Endpoints ... 77
9.4.4 Methods for Multiplicity Control ... 78
9.4.5 Sensitivity Analyses ... 79
9.4.6 Subgroup Analyses ... 79
9.5 Interim Analyses ... 79
9.6 Data Safety Monitoring Committee ... 80
10 SUPPORTING DOCUMENTATION AND OPERATIONAL CONSIDERATIONS ... 80
11 REFERENCES ... 129

LIST OF FIGURES

Figure 1 Study Design 18
Figure 2 Neurology Testing Algorithm 62

LIST OF TABLES

Table 1 Schedule of Activities: Screening Period 19
Table 2 Schedule of Activities: Treatment and Follow-up Period – Main Study (Excluding Substudy and Immunogenicity Cohort Participants) 20
Table 3 Schedule of Activities: Treatment and Follow-up Period – Substudy and Immunogenicity Cohort (USA Participants Only) 22
Table 4 Schedule of Activities: Illness Visits (Participants with Qualifying Clinical Symptoms) 25
Table 5 Objectives and Endpoints 30
Table 6 Highly Effective Methods of Contraception 40
Table 7 Investigational Products 43
Table 8 List of Predefined Solicited Adverse Events for Reactogenicity Assessment 60
Table 9 Populations for Analysis 70
Table 10 Tables for Clinical Abnormalities: Local Reactions to Injectable Product 94
Table 11 Tables for Clinical Abnormalities: Vital Signs 95
Table 12 Tables for Clinical Abnormalities: Systemic (General or Illness) 96
Table 13 Adverse Events of Special Interest 97
Table 14 List of Potential Immune-mediated Medical Conditions 98
Table 15 Overview of Primary and Secondary Efficacy Objectives, Endpoints, and Associated Case Definitions 102

LIST OF APPENDICES

Appendix A Regulatory, Ethical, and Study Oversight Considerations 81
Appendix B Adverse Events: Definitions and Procedures for Recording, Evaluating, Follow-up, and Reporting 87
Appendix C Handling of Human Biological Samples 91
Appendix D Toxicity Grading Scales for Solicited Adverse Events 93
Appendix E Adverse Events of Special Interest 97

Appendix F Overview of Primary and Secondary Efficacy Objectives, Endpoints, and Associated Case Definitions 101
Appendix G Abbreviations 109
Appendix H Protocol Amendment History 111

1 PROTOCOL SUMMARY

1.1 Synopsis

Protocol Title: A Phase III Randomized, Double-blind, Placebo-controlled Multicenter Study in Adults to Determine the Safety, Efficacy, and Immunogenicity of AZD1222, a Non-replicating ChAdOx1 Vector Vaccine, for the Prevention of COVID-19

Short Title: Phase III Double-blind, Placebo-controlled Study of AZD1222 for the Prevention of COVID-19 in Adults

Rationale: The aim of the study is to assess the safety, efficacy, and immunogenicity of AZD1222 for the prevention of COVID-19. The COVID-19 pandemic has caused major disruption to healthcare systems with significant socioeconomic impacts. Currently, there are no specific treatments available against COVID-19 and accelerated vaccine development is urgently needed. A safe and effective vaccine for COVID-19 prevention would have significant public health impact.

Objectives and Endpoints

Objective [a]	Estimand [b] Description/Endpoint
**PRIMARY**	
1 To estimate the efficacy of 2 IM doses of AZD1222 compared to saline placebo for the prevention of COVID-19	**Population:** Fully vaccinated analysis set
	**Endpoint:** A binary response, whereby a participant with negative serostatus at baseline is defined as a COVID-19 case if their first case of SARS-CoV-2 RT-PCR-positive symptomatic illness occurs ≥ 15 days post second dose of study intervention. Otherwise, a participant is not defined as a COVID-19 case.
	**Intercurrent events**: For participants who withdraw from the study prior to having met the criteria for the primary efficacy endpoint, absence of data following these participants' withdrawal will be treated as missing (ie, counted as not having met the criteria); participants who withdraw before 15 days post second dose or who have a case prior to 15 days post second dose will be excluded from primary endpoint analysis. Participants unblinded to treatment assignment prior to having met the criteria for the primary efficacy endpoint will be censored at the date of unblinding.
	**Summary measure:** VE, calculated as 1-relative risk. (Relative risk is the incidence in the vaccine group relative to the incidence in the control group.)

Objective [a]	Estimand [b] Description/Endpoint
2 To assess the safety and tolerability of 2 IM doses of AZD1222 compared to saline placebo	a) Incidence of AEs for 28 days post each dose of study intervention
	b) Incidence of SAEs, MAAEs, and AESIs from Day 1 post treatment through Day 730
3 To assess the reactogenicity of 2 IM doses of AZD1222 compared to saline placebo (Substudy only)	Incidence of local and systemic solicited AEs for 7 days post each dose of study intervention
**SECONDARY**	
1 To estimate the efficacy of 2 IM doses of AZD1222 compared to saline placebo for the prevention of SARS-CoV-2 infection	The incidence of the first post-treatment response (negative at baseline to positive post treatment with study intervention) for SARS-CoV-2 Nucleocapsid antibodies occurring ≥ 15 days post second dose of study intervention [c]
2 To estimate the efficacy of 2 IM doses of AZD1222 compared to saline placebo for the prevention of symptomatic COVID-19 using CDC criteria	The incidence of the first case of SARS-CoV-2 RT-PCR-positive symptomatic illness occurring ≥ 15 days post second dose of study intervention using CDC criteria
3 To estimate the efficacy of 2 IM doses of AZD1222 compared to saline placebo for the prevention of University of Oxford-defined symptomatic COVID-19	The incidence of the first case of SARS-CoV-2 RT-PCR-positive symptomatic illness occurring ≥ 15 days post second dose of study intervention using University of Oxford-defined symptom criteria
4 To estimate the efficacy of 2 IM doses of AZD1222 compared to saline placebo in the prevention of COVID-19 in all study participants, regardless of evidence of prior SARS-CoV-2 infection	The incidence of the first case of SARS-CoV-2 RT-PCR-positive symptomatic illness occurring ≥ 15 days post second dose of study intervention regardless of evidence of prior SARS-CoV-2 infection [c]
5 To estimate the efficacy of 2 IM doses of AZD1222 compared to saline placebo for the prevention of severe or critical symptomatic COVID-19	a) The incidence of SARS-CoV-2 RT-PCR-positive severe or critical symptomatic illness occurring ≥ 15 days post second dose of study intervention [c]
	b) The incidence of SARS-CoV-2 RT-PCR-positive severe or critical symptomatic illness occurring post first dose of study intervention
6 To estimate the efficacy of 2 IM doses of AZD1222 compared to saline placebo for the prevention of COVID-19-related Emergency Department visits	The incidence of COVID-19-related Emergency Department visits occurring ≥ 15 days post second dose of study intervention [c]
7 To assess antibody responses to AZD1222 S antigen following 2 IM doses of AZD1222 or saline placebo (Substudy and Illness Visits only)	a) Post-treatment GMTs and GMFRs from day of dosing baseline value to 28 days post each dose in SARS-CoV-2 S, RBD antibodies (MSD serology assay)
	b) The proportion of participants who have a post-treatment seroresponse (≥ 4-fold rise in titers from day of dosing baseline value to 28 days post each dose) to the S, RBD antigens of AZD1222 (MSD serology assay)

Objective [a]	Estimand [b] Description/Endpoint
8 To determine anti-SARS-CoV-2 neutralizing antibody levels in serum following 2 IM doses of AZD1222 or saline placebo<br>(Substudy and Illness Visits only)	a) Post-treatment GMTs and GMFRs from day of dosing baseline value to 28 days post each dose in SARS-CoV-2 neutralizing antibodies (wild-type assay or pseudo-neutralization assay)
	b) Proportion of participants who have a post-treatment seroresponse (≥ 4-fold rise in titers from day of dosing baseline value to 28 days post each dose) to AZD1222 as measured by SARS-CoV-2 neutralizing antibodies (wild-type assay or pseudo-neutralization assay)
9 To estimate the efficacy of AZD1222 compared to saline placebo for the prevention of COVID-19 following the first dose	The incidence of SARS-CoV-2 RT-PCR-positive symptomatic illness occurring post first dose of study intervention

[a] Substudy: The substudy will be conducted only in the USA. The first participants randomized in each age group in the USA, including 1 500 participants 18 to 55 years of age, 750 participants 56 to 69 years of age, and 750 participants ≥ 70 years of age, will also participate in a substudy assessing the reactogenicity and immunogenicity of AZD1222.
Illness Visits: Participants who present with qualifying symptoms will be tested for SARS-CoV-2 and if positive, will complete illness visits.

[b] Estimand is the target of estimation to address the scientific question of interest posed by the primary objective. Attributes of an estimand include the population of interest, the variable (or endpoint) of interest, the specification of how intercurrent events are reflected in the scientific question of interest, and the population-level summary for the variable.

[c] Key secondary endpoint.

AE = adverse event; AESI = adverse event of special interest; CDC = Centers for Disease Control and Prevention; COVID-19 = coronavirus disease 2019; GMFR = geometric mean fold rise; GMT = geometric mean titer; IFN-γ = interferon-gamma; IM = intramuscular; MAAE = medically attended adverse event; MSD = Meso Scale Discovery; RT-PCR = reverse transcriptase polymerase chain reaction; RBD = receptor binding domain; S = Spike; SAE = serious adverse event; SARS-CoV-2 = severe acute respiratory syndrome-coronavirus-2; USA = United States of America; VE = vaccine efficacy.

For exploratory objectives, see Section 3.

Overall Design: D8110C00001 is a Phase III randomized, double-blind, placebo-controlled, multicenter study assessing the safety, efficacy, and immunogenicity of AZD1222 compared to saline placebo for the prevention of COVID-19. Approximately 150 sites globally will participate in this study.

Participants will be adults ≥ 18 years of age who are healthy or have medically-stable chronic diseases, and are at increased risk for SARS-CoV-2 acquisition and COVID-19. Approximately 30 000 participants will be randomized in a 2:1 ratio to receive 2 IM doses of either 5×10^{10} vp (nominal, $\pm 1.5 \times 10^{10}$ vp) AZD1222 (n = approximately 20 000) or saline placebo (n = approximately 10 000) 4 weeks apart, on Days 1 and 29. Randomization will be stratified by age (≥ 18 to < 65 years, and ≥ 65 years), with at least 25% of participants to be enrolled in the older age stratum. Participants who received their first dose of study intervention between 28 August 2020 and 06 September 2020 will receive their second dose of study intervention outside of the study window.

All participants will be assessed for efficacy and safety. The first participants randomized in each age group in the USA, including 1 500 participants 18 to 55 years of age, 750 participants 56 to 69 years of age, and 750 participants ≥ 70 years of age, will also participate in a substudy assessing the reactogenicity and immunogenicity of AZD1222.

To further investigate cell-mediated immunogenicity, in particular Th1/Th2 responses, after AZD1222 or saline placebo administration, an immunogenicity cohort of approximately 300 participants was added.

A PSRT will provide support for blinded safety surveillance during the study. Additionally, an independent COVID-19 Vaccine DSMB organized by the National Institutes of Health, National Institute for Allergy and Infectious Diseases, will provide oversight, to ensure safe and ethical conduct of the study. An independent Neurological AESI Expert Committee will be available to review and provide advice to the PSRT and the COVID-19 Vaccine DSMB on request about the diagnosis and causality assessment of selected neurological AESIs.

Participants will remain on study for 2 years following administration of the first dose of study intervention (Day 730). If AZD1222 is proven to be safe and efficacious based on the primary endpoint analysis, following discussion at that time with the US FDA, other Regulators if appropriate, and the COVID-19 Vaccine DSMB, participants allocated to the saline placebo group will be offered AZD1222 when doses are available. Saline placebo participants treated with AZD1222 will continue to be followed in the study.

Disclosure Statement: This is a parallel-group preventive study with 2 arms that is participant-, investigator-, and Sponsor-blinded.

Number of Participants: Approximately 33 000 participants will be screened to achieve up to approximately 30 000 participants randomly assigned to study intervention, including approximately 20 000 participants randomized to the AZD1222 arm and approximately 10 000 participants randomized to the saline placebo arm.

Note: ‘Enrolled’ means a participant's, or their legally acceptable representative’s, agreement to participate in a clinical study following completion of the informed consent process. Potential participants who are screened for the purpose of determining eligibility for the study, but are not randomly assigned in the study, are considered ‘screen failures’.

Intervention Groups and Duration: Participants will be randomized in a 2:1 ratio to receive 2 IM doses of 5×10^{10} vp (nominal, $\pm 1.5 \times 10^{10}$ vp) AZD1222 or saline placebo. Study intervention will be administered on Days 1 and 29.

Data Monitoring Committee: Yes

Statistical Methods

Primary efficacy endpoint

The primary efficacy endpoint is a binary response, whereby a participant with negative serostatus at baseline is defined as a COVID-19 case if their first case of SARS-CoV-2 RT-PCR-positive symptomatic illness occurs ≥ 15 days post second dose of study intervention. Otherwise, a participant is not defined as a COVID-19 case.

Sample size

Approximately 33 000 participants will be screened such that approximately 30 000 participants will be randomized in a 2:1 ratio to receive 2 IM doses of either 5×10^{10} vp (nominal, $\pm 1.5 \times 10^{10}$ vp) AZD1222 (the active group, n = approximately 20 000) or saline placebo (the control group, n = approximately 10 000) 4 weeks apart, on Days 1 and 29.

The sample size calculations are based on the primary efficacy endpoint and were derived following a modified Poisson regression approach (Zou 2004). The calculations account for an interim and primary analysis, and the timing of these analyses will be driven by the number of events observed in the study. The interim analysis will be carried out when approximately 50% of the total amount of statistical information is available. A Lan-DeMets alpha-spending function has been used to control the overall type I error at 5% with 0.31% alpha at the interim analysis and 4.9% at the primary analysis. The calculations assume minimal loss to follow-up as it is anticipated that participants will remain engaged in the study. All participants will be followed for the entire duration of the study.

For the primary efficacy analysis, approximately 150 events meeting the primary efficacy endpoint definition within the population of participants who are seronegative at baseline are required across the active and control groups to detect a VE of 60% with > 90% power. These calculations assume an observed attack rate of approximately 0.8% and are based on a 2-sided test, where the lower bound of the 2-sided 95.10% CI for VE is required to be greater than 30% with an observed point estimate of at least 50%.

An interim efficacy analysis will be conducted when approximately 75 events meeting the primary endpoint definition have been reported across the active and control groups within the population of participants who are seronegative at baseline, which will give > 70% power to detect a VE of 70% and > 90% power to detect a VE of 75%. These calculations assume an observed attack rate of approximately 0.4% and are based on a 2-sided test, where the lower bound of the 2-sided 99.69% CI for VE is required to be greater than 30% with an observed point estimate of at least 50%. A statistically significant finding at the interim analysis will not be considered a reason to stop the study, but instead will be interpreted as early assessment of efficacy.

Primary estimand

The primary estimand will be used for the analysis of the primary efficacy endpoint. It will be based on participants in the fully vaccinated analysis set, defined as all randomized participants who are seronegative at baseline, receive 2 doses of study intervention, and who remain on-study 15 days after their second dose without having had a prior SARS-CoV-2 RT-PCR-positive confirmed COVID-19 infection, analyzed according to their randomized treatment. For participants with multiple events, only the first occurrence will be used for the primary efficacy endpoint analysis. The set of intercurrent events for this estimand consists of participants who withdraw from the study or are unblinded to treatment assignment prior to having met the primary efficacy endpoint. The intercurrent event of early study withdrawal will be handled using the treatment policy strategy and the absence of data following these participants' withdrawal will be treated as missing (ie, counted as not having met the criteria). Participants unblinded to treatment assignment prior to having met the criteria for the primary efficacy endpoint will be censored at the date of unblinding. Participants who withdraw before 15 days post second dose or who have a case prior to 15 days post second dose will be excluded from primary endpoint analysis.

Primary efficacy analysis

As the primary efficacy analysis, the plan is to use the primary estimand and a Poisson regression model with robust variance (Zou 2004) to analyze the primary efficacy endpoint, including study arm and age as covariates as well as the log of the follow-up time as an offset. The VE will be estimated from the model, which will give the RRR in the incidence of SARS-CoV-2 RT-PCR-positive symptomatic illness. VE is calculated as RRR = 100*(1-relative risk), which is the incidence in the vaccine group relative to the incidence in the control group expressed as a percentage. At the interim analysis, the VE will be presented with a 2-sided 99.69% CI, and statistical significance will be achieved if the 2-sided 99.69% CI is > 30%. The success criterion for the interim analysis will be statistical significance with an observed VE point estimate of at least 50%. At the primary analysis, VE will be presented with a 2-sided 95.10% CI, and statistical significance will be achieved if the 2-sided 95.10% CI is > 30%. The success criterion for the primary analysis of the study will be statistical significance with an observed VE point estimate of at least 50%.

Model assumptions will be checked and the robustness of the primary analysis will be assessed. The Poisson regression model with robust variance has the flexibility for exploring multiple imputation approaches using, eg, the observed placebo attack rate to impute missing data. If the Poisson regression model with robust variance fails to converge, an alternative approach will be implemented. Full details will be documented in the SAP.

1.2 Schema

Figure 1 Study Design

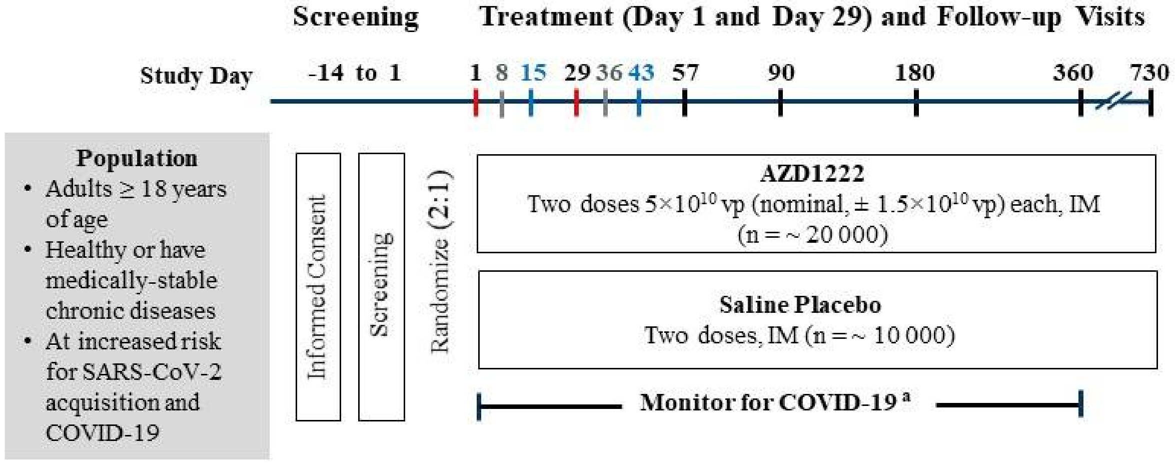

[a] Participants who present with qualifying symptoms will be tested for SARS-CoV-2 and if positive, will complete illness visits.
Red bars (Day 1 and Day 29): Administration of study intervention.
Gray bars (Day 8 and Day 36): Visits will be telephone contacts, not study site visits.
Blue bars (Day 15 and Day 43): Visits will only be for participants in the substudy. The first participants randomized in each age group in the USA, including 1 500 participants 18 to 55 years of age, 750 participants 56 to 69 years of age, and 750 participants ≥ 70 years of age, will also participate in the substudy assessing the reactogenicity and immunogenicity of AZD1222.
COVID-19 = coronavirus disease 2019; IM = intramuscular; SARS-CoV-2 = severe acute respiratory syndrome-coronavirus-2; USA = United States of America; vp = viral particles.

1.3 Schedule of Activities

The SoA tables include:

- Table 1, Screening Period
- Table 2, Treatment and Follow-up Period – Main Study (Excluding Substudy and Immunogenicity Cohort Participants)
- Table 3, Treatment and Follow-up Period – Substudy and Immunogenicity Cohort (USA Participants Only)
- Table 4, Illness Visits (Participants with Qualifying Clinical Symptoms)

Table 1 Schedule of Activities: Screening Period

Procedure / Study Day	Day -14 to Day 1 [a]	For details see Section
Informed consent: main study	X	5.1
Assignment SID number	X	6.3
Medical history	X	5.1, 5.2
Complete physical examination, including height and weight	X	8.2.1
Vital signs (including pulse oximetry)	X	8.2.2
Pregnancy test – urine or serum (WOCBP only) [b]	X	8.2.3
Assessment of SAEs	X	8.3
Concomitant medications	X	6.5
Verify eligibility criteria	X	5.1, 5.2

[a] If screening and dosing occur at the same visit, only one evaluation is required.

[b] If urine tests positive or indeterminate, a quantitative serum β-hCG will be performed for confirmation.

β-hCG = beta-human chorionic gonadotropin; SAE = serious adverse event; SID = subject identification; WOCBP = women of childbearing potential.

Table 2 Schedule of Activities: Treatment and Follow-up Period – Main Study (Excluding Substudy and Immunogenicity Cohort Participants)

Procedure	Treatment and Follow-up Period									For details see Section
**Day**	**1**	**8 [a]**	**29**	**36 [a]**	**57**	**90**	**180**	**360**	**730**	
**Window (days)**	**NA**	**± 3**	**-3 to +7**	**± 3**	**± 3**	**± 5**	**± 10**	**± 15**	**± 30**	
Medical history	X									5.1, 5.2
Targeted physical examination	X									8.2.1
Vital signs (including pulse oximetry)	X									8.2.2
Pregnancy test – urine or serum (WOCBP only) [b]	X (predose)		X (predose)							8.2.3
Concomitant medications	X	X	X	X	X	As applicable, for treatment of SAE, MAAE, or AESI [c]				6.5
Verify eligibility criteria	X									5.1, 5.2
**Study intervention administration**	X		X							6.1, 6.2
**Efficacy assessments**										
Weekly telephone/email/text contacts - monitoring for COVID-19 qualifying symptoms [d]	←							→		8.1.1
Nasal swab for SARS-CoV-2 RT-PCR (local laboratory)	X (predose)									
Serum sample for SARS-CoV-2 serology testing	X (predose)		X (predose)		X	X	X	X	X	8.5.2
**Immunogenicity assessments**										
Serum sample for exploratory assessment	X (predose)		X (predose)		X					8.5.2.5
**Safety assessments**										
AEs	X	X	X	X	X					8.3
SAEs, MAAEs, and AESIs	X	X	X	X	X	X	X	X	X	
Telephone contact for safety monitoring		X		X						

Table 2 Schedule of Activities: Treatment and Follow-up Period – Main Study (Excluding Substudy and Immunogenicity Cohort Participants)

Procedure	Treatment and Follow-up Period									For details see Section
Day	1	8 [a]	29	36 [a]	57	90	180	360	730	
Window (days)	NA	± 3	-3 to +7	± 3	± 3	± 5	± 10	± 15	± 30	

[a] Not a study site visit; participants will be contacted by telephone for safety monitoring.

[b] If urine tests positive or indeterminate, a quantitative serum β-HCG will be performed for confirmation.

[c] Vaccinations, other than AZD1222, for prevention of SARS-CoV-2 or COVID-19 should be recorded if administered at any time during the study.

[d] Weekly contact with participants to remind them to present to the study site for SARS-CoV-2 testing if they have qualifying symptoms.

AE = adverse event; AESI = adverse event of special interest; β-hCG = beta-human chorionic gonadotropin; MAAE = medically attended adverse event; NA = not applicable; SAE = serious adverse event; SARS-CoV-2 = severe acute respiratory syndrome-coronavirus-2; WOCBP = women of childbearing potential.

Table 3 Schedule of Activities: Treatment and Follow-up Period – Substudy and Immunogenicity Cohort (USA Participants Only)

**Procedure**	**Treatment and Follow-up Period**											**For details see Section**
**Day**	**1**	**8** [a]	**15**	**29**	**36** [a]	**43**	**57**	**90**	**180**	**360**	**730**	
**Window (days)**	**NA**	**± 3**	**± 1**	**-3 to +7**	**± 3**	**± 3**	**± 3**	**± 5**	**± 10**	**± 15**	**± 30**	
Medical history	X											5.1, 5.2
Targeted physical examination	X											8.2.1
Vital signs (including pulse oximetry)	X											8.2.2
Pregnancy test – urine or serum (WOCBP only) [b]	X (predose)			X (predose)								8.2.3
Concomitant medications	X	X	X	X	X	X	X	As applicable, for treatment of SAE, MAAE, or AESI [c]				6.5
Verify eligibility criteria	X											5.1, 5.2
**Study intervention administration**	X			X [d]								6.1.1
**Efficacy assessments**												
Weekly telephone/email/text contacts - monitoring for COVID-19 qualifying symptoms [e]	←→											8.1.1
Nasal swab for SARS-CoV-2 RT-PCR (local laboratory)	X (predose)											8.1.1
Serum sample for SARS-CoV-2 serology testing	X (predose)		X	X (predose)		X	X	X	X	X	X	8.5.2
**Immunogenicity assessments**												
Serum sample for ChAdOx1 nAbs assessment	X (predose)			X (predose)			X		X	X	X	8.5.2
Serum sample for seasonal CoV serology testing	X (predose)			X (predose)			X		X	X		8.5.2

Table 3 Schedule of Activities: Treatment and Follow-up Period – Substudy and Immunogenicity Cohort (USA Participants Only)

**Procedure**	**Treatment and Follow-up Period**											**For details see Section**
**Day**	**1**	**8 [a]**	**15**	**29**	**36 [a]**	**43**	**57**	**90**	**180**	**360**	**730**	
**Window (days)**	**NA**	**± 3**	**± 1**	**-3 to +7**	**± 3**	**± 3**	**± 3**	**± 5**	**± 10**	**± 15**	**± 30**	
PBMCs for assessment of B-cell and T-cell responses [f]	X (predose)		X			X			X			8.5.2
Serum sample for SARS-CoV-2 nAbs assessment	X (predose)		X	X (predose)		X	X		X	X		8.5.2
Nasal adsorption for SARS-CoV-2 mucosal responses (optional)	X (predose)		X	X (predose)		X	X		X	X		8.5.2
Serum sample for ACE2 competition serology	X (predose)			X (predose)			X		X			8.5.2
**Safety assessments**												
Local and systemic predefined solicited AEs (recorded daily by participant in Solicited AE e-Diary) – Substudy only	X (through Day 8)			X (through Day 36)								8.3.7
AEs	X	X	X	X	X	X	X					8.3
SAEs, MAAEs, and AESIs	X	X	X	X	X	X	X	X	X	X	X	8.3
Telephone contact for safety monitoring		X			X							8.3

[a] Not a study site visit; participants will be contacted by telephone for safety monitoring.

[b] If urine tests positive or indeterminate, a quantitative serum β-hCG will be performed for confirmation.

[c] Vaccinations, other than AZD1222, for prevention of SARS-CoV-2 or COVID-19 should be recorded if administered at any time during the study.

[d] Participants who received their first dose of study intervention between 28 August 2020 and 06 September 2020, will receive their second dose of study intervention outside of the study window.

[e] Weekly contact with participants to remind them to present to the study site for SARS-CoV-2 testing if they have qualifying symptom.

[f] PBMCs will be isolated from up to 300 participants in the substudy and approximately 300 participants in the immunogenicity cohort at select study sites, as outlined in the laboratory manual.

Table 3 Schedule of Activities: Treatment and Follow-up Period – Substudy and Immunogenicity Cohort (USA Participants Only)

Procedure	Treatment and Follow-up Period											For details see Section
Day	1	8 [a]	15	29	36 [a]	43	57	90	180	360	730	
Window (days)	NA	± 3	± 1	-3 to +7	± 3	± 3	± 3	± 5	± 10	± 15	± 30	

ACE2 = angiotensin-converting enzyme 2; AE = adverse event (treatment-emergent); AESI = adverse event of special interest; β-hCG = beta-human chorionic gonadotropin; CoV = coronavirus; COVID-19 = coronavirus disease 2019; MAAE = medically attended adverse event; NA = not applicable; nAb = neutralizing antibody; PBMC = peripheral blood mononuclear cell; SAE = serious adverse event; SARS-CoV-2 = severe acute respiratory syndrome-coronavirus-2; USA = United States of America; WOCBP = women of childbearing potential.

Table 4 Schedule of Activities: Illness Visits (Participants with Qualifying Clinical Symptoms)

Procedure [a]	Site Visit	Home Collection by Participant				Site Visit for SARS-CoV-2 Positive Participants Only			For details see Section
**Day**	**1**	**3**	**5**	**8**	**11**	**14**	**21**	**28**	
**Window (days)**	**NA**	**± 1**	**± 1**	**± 2**	**± 2**	**± 2**	**± 2**	**± 2**	
Medical history	X					X	X	X	8.1
Brief physical examination	X					X	X	X	8.2.1
Vital signs (including pulse oximetry)	X					X	X	X	8.2.2
Concomitant medication	←——————————————————————→								6.5
**Efficacy assessments**									
Digital health device	←——————————————————————→								8.1.2.2
Symptoms associated with COVID-19 (recorded daily by participant in Illness e-Diary)	←——————————————————————→								8.1.2.3
**Virology assessments**									
Nasal swab for SARS-CoV-2 RT-PCR (local laboratory)	X								8.1.2.1
Nasopharyngeal swab									
SARS-CoV-2 RT-PCR (central laboratory)	X					X	X	X	8.1.2.1
SARS-CoV-2 sequencing (central laboratory)	X					X			
Respiratory panel	X								8.6.1.1
Saliva sample for viral shedding [b]	X	X	X	X	X	X	X	X	8.6.1.2
**Immunogenicity assessments**									
PBMCs for B-cell and T-cell responses	X [c]					X			8.5.2
Serum sample for SARS-CoV-2 nAbs assessment	X					X		X	
Nasal adsorption for SARS-CoV-2 mucosal responses (optional)	X					X		X	

Table 4 Schedule of Activities: Illness Visits (Participants with Qualifying Clinical Symptoms)

Procedure [a]	Site Visit	Home Collection by Participant				Site Visit for SARS-CoV-2 Positive Participants Only			For details see Section
Day	1	3	5	8	11	14	21	28	
Window (days)	NA	± 1	± 1	± 2	± 2	± 2	± 2	± 2	
Serum sample for ACE2 competition serology	X					X		X	8.5.2
Serum sample for exploratory assessments	X					X		X	
**Safety assessments**									
SAEs, MAAEs, and AESIs	⟵	⟶							8.3
Telephone contact for safety monitoring		X		X					

[a] Following availability of the SARS-CoV-2 RT-PCR results, only participants who test positive will continue with the illness visits, including any home collection requirements. Participants who test negative for SARS-CoV-2 will be instructed to stop all illness visit assessments and return the digital health device (see Section 8.1.2.2).

[b] Collection of saliva sample for viral shedding is optional in non-USA sites and may occur after a feasibility assessment has been completed.

[c] PBMCs will be collected from up to approximately the first 3 000 participants, where operationally feasible, on Day 1 illness visit and any participants with a Day 1 SARS-CoV-2 positive test result by local RT-PCR test (see Section 8.5.2.4).

ACE2 = angiotensin-converting enzyme 2; AESI = adverse events of special interest; CoV = coronavirus; COVID-19 = coronavirus disease 2019; MAAE = medically attended adverse event; NA = not applicable; nAb = neutralizing antibody; PBMC = peripheral blood mononuclear cell; RT-PCR = reverse transcriptase polymerase chain reaction; SAE = serious adverse event; SARS-CoV-2 = severe acute respiratory syndrome-coronavirus-2.

2 INTRODUCTION

AZD1222 is being developed for the prevention of COVID-19. AZD1222 is a recombinant replication-defective chimpanzee adenovirus expressing the SARS-CoV-2 S surface glycoprotein driven by the human cytomegalovirus major immediate early promoter that includes intron A with a human tPA leader sequence at the N terminus.

2.1 Study Rationale

The aim of the study is to assess the safety, efficacy, and immunogenicity of AZD1222 for prevention of COVID-19. The COVID-19 pandemic has caused major disruption to healthcare systems with significant socioeconomic impacts. Currently, there are no licensed preventions available against COVID-19 and accelerated vaccine development is urgently needed. A safe and effective vaccine for COVID-19 prevention would have significant global public health impact.

2.2 Background

In December 2019, a cluster of patients with pneumonia of unknown cause was linked to a seafood wholesale market in Wuhan, China and were later confirmed to be infected with a novel coronavirus, known as 2019-nCoV (Zhu et al 2020). The virus was subsequently renamed to SARS-CoV-2 because it is similar to the coronavirus responsible for SARS-CoV, a lineage B *Betacoronavirus*. SARS-CoV-2 shares more than 79% of its sequence with SARS-CoV, and 50% with the coronavirus responsible for MERS-CoV, a member of the lineage C *Betacoronavirus* (Lu et al 2020). COVID-19 is the infectious disease caused by SARS-CoV-2. By January 2020 there was increasing evidence of human-to-human transmission as the number of cases rapidly began to increase in China. Spread of the virus has been rapid and now encompasses the globe. The WHO declared the novel coronavirus a pandemic on 11 March 2020. As of 18 October 2020, there have been more than 40 million confirmed cases and > 1.1 million deaths globally (WHO 2020). It is believed that evolution of the pandemic will vary across countries, affected in part by different containment strategies ranging from extreme lockdown to relative inaction. As a result, there may be regional waves of the disease and pockets of vulnerable populations. Globally, governments have acknowledged that an effective vaccine against COVID-19 is the only way to guarantee a safe and sustained exit strategy from repeated lockdowns.

CoVs are spherical, enveloped viruses with positive-sense single-stranded RNA genomes. One fourth of their genome is responsible for coding structural proteins, such as the S glycoprotein, envelope, membrane, and nucleocapsid proteins. Envelope, membrane, and nucleocapsid proteins are mainly responsible for virion assembly whilst the S protein is involved in receptor binding, mediating virus entry into host cells during CoVs infection via different receptors (Li 2016). SARS-CoV-2 belongs to the phylogenetic lineage B of the genus *Betacoronavirus* and it recognizes the ACE2 as the entry receptor (Zhou et al 2020). It

is the seventh CoV known to cause human infections and the third known to cause severe disease after SARS-CoV and MERS-CoV.

AZD1222 is a recombinant replication-defective chimpanzee adenovirus expressing the SARS-CoV-2 S surface glycoprotein. Development of AZD1222, previously referred to as ChAdOx1 nCoV-19, was initiated by the University of Oxford with subsequent transfer of development activities to the Sponsor. Nonclinical studies found AZD1222 to be immunogenic in BALB/c and CD-1 mice, ferret, porcine, and NHP models. Whilst a single dose of AZD1222 induced antigen-specific antibody and T-cell responses, a booster immunization enhanced antibody responses, particularly in pigs, with significant increases in SARS-CoV-2 neutralising antibody titres (Graham et al 2020). Further, in a SARS-CoV-2 challenge NHP model, a single administration of AZD1222 significantly reduced viral load in bronchoalveolar lavage fluid and respiratory tract tissue of vaccinated rhesus macaques as compared to vector controls. Importantly, no evidence of VAERD following SARS-CoV-2 challenge in vaccinated rhesus macaques was observed (van Doremalen et al 2020).

The clinical development program for AZD1222 was initiated by the University of Oxford, and currently has 4 ongoing studies being conducted in the UK (FIH COV001 [NCT04324606], COV002 [NCT04400838]), Brazil (COV003 [ISRCTN89951424], and South Africa (COV005 [NCT04444674]). The Sponsor, in addition to this study, has 2 ongoing studies in Russia (D8111C00001 [NCT04540393]) and Japan (D8111C00002).

Preliminary unblinded data are available for the FIH Study COV001 and Study COV002. Study COV001 enrolled the first participant on 23 April 2020 and completed enrollment on 21 May 2020 with 1 077 participants. Preliminary data as of 24 July 2020 from 544 participants who received at least one dose of 5×10^{10} vp AZD1222 and 10 participants who received a second dose of 5×10^{10} vp AZD1222 4 weeks later found the vaccine was generally tolerated, with no treatment-related SAEs reported through 28 days post dose. The most common local solicited AEs were vaccination site pain and tenderness. The most common systemic solicited AEs were chills, feverishness, fever, headache, malaise, and myalgia. The majority of events were mild or moderate in severity and resolved within 1 to 7 days. Following the second dose, a general attenuation in the incidence and severity of local and systemic solicited AEs was observed.

Preliminary immunogenicity data from Study COV001 suggest that a single dose can elicit both humoral and cellular immunogenicity responses and that antibody responses are boosted after a second dose. S-specific T-cell responses peaked on Day 14. Anti-S IgG responses rose by Day 28, and were boosted 3-fold following a second dose.

Neutralizing antibody responses against SARS-CoV-2 were detected in 32 (91%) of 35 participants after a single dose when measured in microneutralization assay (MNA_{80}) and in 35 (100%) participants when measured in plaque reduction neutralization test ($PRNT_{50}$).

After the second dose, all participants had neutralizing activity (9 of 9 in MNA_{80} at Day 42 and 10 of 10 in Marburg virus neutralization assay on Day 56). Neutralizing antibody responses correlated strongly with antibody levels measured by ELISA (Folegatti et al 2020b).

As of 01 October 2020, Study COV002 enrolled 10 013 participants, including 5 152 participants who received at least one dose of AZD1222 ranging from 2.2×10^{10} vp to 5×10^{10} vp. Based on preliminary data as of 24 July 2020, the local and systemic solicited-AE profile following a single dose of 5×10^{10} vp in participants 18 to 55 years of age was generally comparable to results in Study COV001. In general, a decline in the incidence and severity of solicited AEs was observed across the age groups (18-55, 56-69, and ≥ 70 years).

Another ChAdOx1-vectored vaccine expressing the full-length S protein from a related betacoronavirus, MERS-CoV, has been given to 53 participants as part of 2 ongoing dose-escalation Phase I studies (MERS001 [NCT03399578] and MERS002 [NCT04170829], sponsored by the University of Oxford) at doses ranging from 5×10^{9} vp to 5×10^{10} vp. Preliminary immunogenicity data from MERS001 suggested that a single dose of ChAdOx1 MERS can elicit both humoral and cellular responses. Overall, the vaccine was safe and generally well tolerated, with no serious adverse reactions reported in either study.

The ChAdOx1 platform has been used in 14 clinical studies sponsored by the University of Oxford with immunogens from multiple pathogens such as influenza, tuberculosis, malaria, chikungunya, Zika, MERS-CoV, and Meningitis B. Over 360 healthy adult participants have received ChAdOx1-vectored vaccines in these studies. These vaccines demonstrated robust immunogenicity after a single dose and favorable safety profiles, with no vaccine-related SAEs.

See the AZD1222 IB, Sections 4 and 5 for additional information on nonclinical and clinical studies, respectively, of AZD1222 and related ChAdOx1-vectored vaccines. Detail on the development and chemistry of AZD1222 is provided in the IB, Section 3.

Overall, the preliminary data from the AZD1222 clinical and nonclinical studies, and the acceptable safety and efficacy data for the MERS-CoV vaccine and other ChAdOx1-vectored vaccines, support further development of AZD1222 for the prevention of COVID-19.

2.3 Benefit/Risk Assessment

More detailed information about the known and expected benefits and potential risks of AZD1222 can be found in the AZD1222 IB.

2.3.1 Risk Assessment

AZD1222

Based on preliminary clinical data from Study COV001 and Study COV002, the most common local solicited AEs were vaccination site pain and tenderness. Common systemic solicited AEs across the 2 studies included chills, feverishness, fever, fatigue, headache, joint pain, malaise, and myalgia. The majority of events were mild or moderate in severity and resolved within 1 to 7 days (Section 2.2 and AZD1222 IB).

There are no identified risks for AZD1222. Important potential risks are immunologic reactions: serious hypersensitivity (anaphylactic reactions) and vasculitides; neurologic reactions: demyelinating diseases; and vaccine-associated enhanced respiratory disease. A summary of risks associated with AZD1222 and safety information reported across the AZD1222 clinical studies are provided in the current version of the AZD1222 IB.

2.3.2 Benefit Assessment

Recipients of AZD1222 do not have any guaranteed benefit, however, AZD1222 may be efficacious and offer participants protection from COVID-19. The information gained from this study will inform development decisions.

2.3.3 Overall Benefit: Risk Conclusion

For the safety of participants, the protocol has incorporated various risk mitigation measures including appropriate inclusion and exclusion criteria, close monitoring of participants, and stopping criteria. An independent DSMB will provide study oversight, evaluating cumulative safety and other clinical data at regular intervals. Taking these measures into account, the potential risks identified in association with AZD1222 are justified by the anticipated benefit that may be afforded to participants for the prevention of COVID-19.

3 OBJECTIVES AND ENDPOINTS

Table 5 Objectives and Endpoints

Objective [a]	Estimand [b] Description/Endpoint
**PRIMARY**	
1 To estimate the efficacy of 2 IM doses of AZD1222 compared to saline placebo for the prevention of COVID-19	**Population:** Fully vaccinated analysis set
	**Endpoint:** A binary response, whereby a participant with negative serostatus at baseline is defined as a COVID-19 case if their first case of SARS-CoV-2 RT-PCR-positive symptomatic illness occurs ≥ 15 days post second dose of study intervention. Otherwise, a participant is not defined as a COVID-19 case.

Table 5 Objectives and Endpoints

Objective [a]	Estimand [b] Description/Endpoint
	**Intercurrent events**: For participants who withdraw from the study prior to having met the criteria for the primary efficacy endpoint, absence of data following these participants' withdrawal will be treated as missing (ie, counted as not having met the criteria); participants who withdraw before 15 days post second dose or who have a case prior to 15 days post second dose will be excluded from primary endpoint analysis. Participants unblinded to treatment assignment prior to having met the criteria for the primary efficacy endpoint will be censored at the date of unblinding.
	**Summary measure:** VE, calculated as 1-relative risk. (Relative risk is the incidence in the vaccine group relative to the incidence in the control group.)
2 To assess the safety and tolerability of 2 IM doses of AZD1222 compared to saline placebo	a) Incidence of AEs for 28 days post each dose of study intervention
	b) Incidence of SAEs, MAAEs, and AESIs from Day 1 post treatment through Day 730
3 To assess the reactogenicity of 2 IM doses of AZD1222 compared to saline placebo<br>(Substudy only)	Incidence of local and systemic solicited AEs for 7 days post each dose of study intervention
**SECONDARY**	
1 To estimate the efficacy of 2 IM doses of AZD1222 compared to saline placebo for the prevention of SARS-CoV-2 infection	The incidence of the first post-treatment response (negative at baseline to positive post treatment with study intervention) for SARS-CoV-2 Nucleocapsid antibodies occurring ≥ 15 days post second dose of study intervention [c]
2 To estimate the efficacy of 2 IM doses of AZD1222 compared to saline placebo for the prevention of symptomatic COVID-19 using CDC criteria	The incidence of the first case of SARS-CoV-2 RT-PCR-positive symptomatic illness occurring ≥ 15 days post second dose of study intervention using CDC criteria
3 To estimate the efficacy of 2 IM doses of AZD1222 compared to saline placebo for the prevention of University of Oxford-defined symptomatic COVID-19	The incidence of the first case of SARS-CoV-2 RT-PCR-positive symptomatic illness occurring ≥ 15 days post second dose of study intervention using University of Oxford-defined symptom criteria
4 To estimate the efficacy of 2 IM doses of AZD1222 compared to saline placebo in the prevention of COVID-19 in all study participants, regardless of evidence of prior SARS-CoV-2 infection	The incidence of the first case of SARS-CoV-2 RT-PCR-positive symptomatic illness occurring ≥ 15 days post second dose of study intervention regardless of evidence of prior SARS-CoV-2 infection [c]
5 To estimate the efficacy of 2 IM doses of AZD1222 compared to saline placebo for the prevention of severe or critical symptomatic COVID-19	a) The incidence of SARS-CoV-2 RT-PCR-positive severe or critical symptomatic illness occurring ≥ 15 days post second dose of study intervention [c]
	b) The incidence of SARS-CoV-2 RT-PCR-positive severe or critical symptomatic illness occurring post first dose of study intervention

Table 5 Objectives and Endpoints

Objective [a]	Estimand [b] Description/Endpoint
6 To estimate the efficacy of 2 IM doses of AZD1222 compared to saline placebo for the prevention of COVID-19-related Emergency Department visits	The incidence of COVID-19-related Emergency Department visits occurring ≥ 15 days post second dose of study intervention [c]
7 To assess antibody responses to AZD1222 S antigen following 2 IM doses of AZD1222 or saline placebo (Substudy and Illness Visits only)	a) Post-treatment GMTs and GMFRs from day of dosing baseline value to 28 days post each dose in SARS-CoV-2 S, RBD antibodies (MSD serology assay)
	b) The proportion of participants who have a post-treatment seroresponse (≥ 4-fold rise in titers from day of dosing baseline value to 28 days post each dose) to the S, RBD antigens of AZD1222 (MSD serology assay)
8 To determine anti-SARS-CoV-2 neutralizing antibody levels in serum following 2 IM doses of AZD1222 or saline placebo (Substudy and Illness Visits only)	a) Post-treatment GMTs and GMFRs from day of dosing baseline value to 28 days post each dose in SARS-CoV-2 neutralizing antibodies (wild-type assay or pseudo-neutralization assay)
	b) Proportion of participants who have a post-treatment seroresponse (≥ 4-fold rise in titers from day of dosing baseline value to 28 days post each dose) to AZD1222 as measured by SARS-CoV-2 neutralizing antibodies (wild-type assay or pseudo-neutralization assay)
9 To estimate the efficacy of AZD1222 compared to saline placebo for the prevention of COVID-19 following the first dose	The incidence of SARS-CoV-2 RT-PCR-positive symptomatic illness occurring post first dose of study intervention
**EXPLORATORY**	
1 To estimate the efficacy of 2 IM doses of AZD1222 compared to saline placebo for the all-cause mortality	The incidence of all-cause mortality from Day 1 through Day 730
2 To estimate the efficacy of 2 IM doses of AZD1222 compared to saline placebo for COVID-19-related deaths	The incidence of COVID-19-related deaths occurring from Day 1 through Day 730
3 To estimate the efficacy of 2 IM doses of AZD1222 compared to saline placebo for the prevention of COVID-19-related hospitalizations	a) The incidence of COVID-19-related hospitalizations occurring ≥ 15 days post second dose of study intervention
	b) The incidence of COVID-19-related hospitalizations occurring post first dose of study intervention
4 To estimate the efficacy of 2 IM doses of AZD1222 compared to saline placebo for the prevention of COVID-19-related ICU admissions	a) The incidence of COVID-19-related ICU admissions occurring ≥ 15 days post second dose of study intervention
	b) The incidence of COVID-19-related ICU admissions occurring post first dose of study intervention
5 To quantify SARS-Cov-2 viral loads in infected participants treated with 2 IM doses of AZD1222 or saline placebo (Illness Visits only)	Viral genome copies in NP swabs collected at Illness Visits as determined by qRT-PCR

Table 5 Objectives and Endpoints

Objective [a]	Estimand [b] Description/Endpoint
6 To characterize sequence variations in SARS-CoV-2 through genotypic analyses in participants treated with 2 IM doses of AZD1222 or saline placebo (Illness Visits only)	Genotypic analysis of SARS-CoV-2 from NP swabs collected on Day 1 illness visit
7 To quantify duration of viral shedding in symptomatic SARS-CoV-2 infected participants treated with 2 IM doses of AZD1222 or saline placebo (Illness Visits only)	Duration of SARS-CoV-2 shedding in saliva over time
8 To assess the biometric profiles associated with COVID-19 using a biosensor in participants treated with 2 IM doses of AZD1222 or saline placebo (Illness Visits only)	Biophysical parameters, including but not limited to serial measurements of skin temperature, heart rate, respiratory rate, blood oxygen saturation, and physical activity, recorded using a biosensor from illness visits Day 1 through Day 28
9 To assess symptoms associated with COVID-19 using an e-Diary in participants treated with 2 IM doses of AZD1222 or saline placebo (Illness Visits only)	Symptoms recorded by participants in an Illness e-Diary from illness visits Day 2 through Day 28
10 To assess SARS-CoV-2 specific antibodies in an ACE2 competition assay following 2 IM doses of AZD1222 or saline placebo (Substudy only)	a) Post-treatment GMTs and GMFRs from Day 1 baseline value to 28 days post each dose in ACE2 competing antibodies from serum samples
	b) Proportion of participants who have a post-treatment seroresponse (≥ 4-fold rise in titers from day of dosing baseline value to 28 days post each dose) in ACE2 competing antibodies
11 To assess B- and T-cell responses following 2 IM doses of AZD1222 or saline placebo (Substudy only)	a) Quantification of (IFN-γ) ELISpot responses to SARS-CoV-2 S protein from day of dosing baseline to 14 days post each dose
	b) Intracellular cytokine staining and flow cytometry for B- and T-cell responses from day of dosing baseline to 14 days post each dose
12 To assess SARS-CoV-2 antibodies in nasal secretions following 2 IM doses of AZD1222 or saline placebo (Substudy only)	a) Post-treatment GMTs and GMFRs from Day 1 baseline value to 28 days post each dose in SARS-CoV-2 S, RBD, and Nucleocapsid antibodies (MSD serology assay)
	b) Proportion of participants who have a post-treatment seroresponse (≥ 4-fold rise in titers from Day 1 baseline value to 28 days post each dose) to SARS-CoV-2 S, RBD, and Nucleocapsid antigens (MSD serology assay)
13 To assess anti-vector responses to the ChAdOx-1 adenovirus vector following 2 IM doses of AZD1222 or saline placebo (Substudy only)	Proportion of participants who have a post-treatment seroresponse (≥ 4-fold rise in titers from Day 1 baseline value to 28 days post each dose) to AZD1222 as measured by ChAdOx1 neutralizing antibodies

Table 5 Objectives and Endpoints

Objective [a]	Estimand [b] Description/Endpoint
14 To assess additional immune responses following 2 IM doses of AZD1222 or saline placebo	Other exploratory assays for humoral and cellular immune responses may be performed based upon emerging safety, efficacy, and immunogenicity data

[a] Substudy: The substudy will be conducted only in the USA. The first participants randomized in each age group in the USA, including 1 500 participants 18 to 55 years of age, 750 participants 56 to 69 years of age, and 750 participants ≥ 70 years of age, will also participate in a substudy assessing the reactogenicity and immunogenicity of AZD1222.
Illness Visits: Participants who present with qualifying symptoms will be tested for SARS-CoV-2 and if positive, will complete illness visits.

[b] Estimand is the target of estimation to address the scientific question of interest posed by the primary objective. Attributes of an estimand include the population of interest, the variable (or endpoint) of interest, the specification of how intercurrent events are reflected in the scientific question of interest, and the population-level summary for the variable.

[c] Key secondary endpoint.

ACE2 = angiotensin-converting enzyme 2; AE = adverse event; AESI = adverse event of special interest; CDC = Centers for Disease Control and Prevention; COVID-19 = coronavirus disease 2019; ELISpot = enzyme-linked immunospot; GMFR = geometric mean fold rise; GMT = geometric mean titer; ICU = intensive care unit; IFN-γ = interferon-gamma; IM = intramuscular; MAAE = medically attended adverse event; MSD = Meso Scale Discovery; NP = nasopharyngeal; RT-PCR = reverse transcriptase polymerase chain reaction; qRT-PCR = quantitative reverse transcriptase polymerase chain reaction, RBD = receptor binding domain; S = Spike; SAE = serious adverse event; SARS-CoV-2 = severe acute respiratory syndrome-coronavirus-2; USA = United States of America; VE = vaccine efficacy.

4 STUDY DESIGN

4.1 Overall Design

D8110C00001 is a Phase III randomized, double-blind, placebo-controlled, multicenter study assessing the safety, efficacy, and immunogenicity of AZD1222 compared to saline placebo for the prevention of COVID-19. Participants will be adults ≥ 18 years of age who are healthy or have medically-stable chronic diseases, and are at increased risk for SARS-CoV-2 acquisition and COVID-19. Approximately 30 000 participants will be randomized in a 2:1 ratio to receive 2 IM doses of either 5×10^{10} vp (nominal, $\pm 1.5 \times 10^{10}$ vp) AZD1222 (n = approximately 20 000) or saline placebo (n = approximately 10 000) 4 weeks apart, on Days 1 and 29. Randomization will be stratified by age (≥ 18 and < 65 years, and ≥ 65 years), with at least 25% of participants to be enrolled in the older age stratum. Participants who received their first dose of study intervention between 28 August 2020 and 06 September 2020 will receive their second dose of study intervention outside of the study window.

Participants who present with at least one of the qualifying symptoms listed below through Day 360 will be assessed for COVID-19. With the exception of fever, shortness of breath, or difficulty breathing, the symptom must be present for 2 or more days. Participants with a COVID-19 qualifying symptom(s) will be tested for SARS-CoV-2, and if positive will

complete illness visit assessments, as presented in Table 4. See Section 8.1 for details on COVID-19 assessments.

COVID-19 Qualifying Symptoms

Participant must present with at least one of the following symptoms	
**Duration**	**Symptom**
No minimum duration	Fever
	Shortness of breath
	Difficulty breathing
Must be present for ≥ 2 consecutive days	Chills
	Cough
	Fatigue
	Muscle aches
	Body aches
	Headache
	New loss of taste
	New loss of smell
	Sore throat
	Congestion
	Runny nose
	Nausea
	Vomiting
	Diarrhea

Adapted from (CDC 2020).

Safety will be assessed for the duration of the study. AEs will be recorded for 28 days post each dose of study intervention (ie, until Day 29 post first dose and Day 57 post second dose), and SAEs, MAAEs, and AESIs will be recorded through Day 730. See Sections 8.3, 8.3.8, and 8.3.9 for definitions of these events.

The first participants randomized in each age group in the USA, including 1 500 participants 18 to 55 years of age, 750 participants 56 to 69 years of age, and 750 participants ≥ 70 years of age, will also participate in a substudy assessing the reactogenicity and immunogenicity of AZD1222. These 3 000 participants in the substudy will have additional assessments for predefined solicited AEs for 7 days post each dose of study intervention and for humoral and cellular immune responses. Solicited AEs are defined in Section 8.3.7.

Table 2 and Table 3 provide the SoA for the main study and substudy, respectively.

To further investigate cell-mediated immunogenicity, in particular Th1/Th2 responses, after AZD1222 or saline placebo administration, an immunogenicity cohort of approximately 300 participants will be added. Participants will follow the SoA in Table 3 except they will not record reactogenicity.

All participants will remain on study for 2 years following administration of first dose of study intervention (Day 730). If AZD1222 is proven to be safe and efficacious based on the primary endpoint analysis (see Section 9.4.2.1), following discussion at that time with the US FDA, other Regulators if appropriate, and the COVID-19 Vaccine DSMB, participants allocated to the saline placebo group will be offered AZD1222 when doses are available. Saline placebo participants treated with AZD1222 will continue to be followed in the study.

A PSRT will provide support for blinded safety surveillance during the study. Additionally, an independent COVID-19 Vaccine DSMB will provide oversight, to ensure safe and ethical conduct of the study. The COVID-19 Vaccine DSMB will facilitate the interim analysis for efficacy and have the responsibility of evaluating cumulative safety and other clinical study data at regular intervals and for making appropriate recommendations based on the available data. An independent Neurological AESI Expert Committee will be available to review and provide advice to the PSRT and the COVID-19 Vaccine DSMB on request about the diagnosis and causality assessment of selected neurological AESIs occurring in the AZD1222 clinical development program. See Appendix A 5 for additional detail.

4.2 Scientific Rationale for Study Design

4.2.1 Rationale for Study Design and Participant Population

The participant population is male and female adults ≥ 18 years of age who are not immunosuppressed, but are at increased risk of SARS-Cov-2 infection due to their locations or circumstances. Inclusion of older adults is based on data that are being gathered from the ongoing University of Oxford-sponsored studies. Study COV001 (NCT04324606) enrolled adults 18 to 55 years of age. Study COV002 (NCT04400838) enrolled adults 18 years of age and older.

Adults with medically-stable chronic diseases may participate if, according to the judgement of the investigator, hospitalization within the study period is not anticipated and the participant appears likely to be able to remain on study through the end of protocol-specified follow-up.

The study will exclude females who are pregnant or breast-feeding and individuals less than 18 years of age. Women who are pregnant or breast-feeding are excluded at this point as nonclinical developmental and reproductive toxicity studies to support vaccinating these individuals have yet to be performed. Additionally, it is planned that children and adolescents

will be evaluated for their response to the vaccine once safety and efficacy have been established in adults.

Participants who have been previously diagnosed with laboratory-confirmed SARS-CoV-2 infection are excluded from study participation. Participants with previous asymptomatic or undiagnosed infection are not excluded. Participant's baseline serostatus will be determined but baseline serostatus will not be used as a basis for exclusion from the study. Participants who are seropositive at baseline are enrolled in order to gather safety data in this group as it is anticipated that, if proven to be efficacious, the vaccine will rapidly be distributed to millions of individuals and that these individuals will not be tested for serologic evidence of previous infection prior to vaccination. Participant's baseline serostatus will be determined so that subgroup analyses for both safety and efficacy can be performed by baseline serostatus.

The study population represents the initial target population for AZD1222. If AZD1222 demonstrates efficacy for the prevention of COVID-19, the safety and immunogenicity of the vaccine in additional groups such as the immunosuppressed, pregnant women, and children and adolescents may be assessed in future studies.

4.2.2 Rationale for Study Endpoints

The efficacy endpoints in this study are analogous to endpoints used for evaluating the efficacy of influenza vaccines. These definitions have 4 components: (1) a definition of clinical illness; (2) a method of respiratory specimen sampling for the detection of associated shedding of the relevant virus; (3) an assay method for laboratory confirmation; and (4) a defined surveillance period. Assessment of AZD1222 efficacy will begin ≥ 15 days after the second dose of study intervention as this time period is considered necessary for the vaccine to induce protective immune responses.

In the substudy, solicited AEs will be collected for 7 days post each dose of study intervention, a period that has proven adequate to describe reactogenicity events in previous vaccine studies. For all participants, AEs will be collected through 28 days post each dose of study intervention. SAEs, MAAEs, and AESIs and will be collected from Day 1 through end of the study. AESIs include terms identified by the Brighton Collaboration involving events associated with vaccination in general (SPEAC 2020).

4.3 Justification for Dose

The AZD1222 dose of 5×10^{10} vp was selected based on accumulated clinical experience with this vaccine in ongoing clinical studies sponsored by the University of Oxford (see Section 2.2). Safety and immunogenicity data from an additional clinical study, MERS001 (NCT03399578), using the same ChAdOx1 vector, also helped inform dose selection.

MERS001 was the first clinical study of a ChAdOx1-vectored vaccine expressing the full-length S protein from a separate, but related, betacoronavirus. ChAdOx1 MERS has been given to 31 participants to date at doses ranging from 5×10^9 vp to 5×10^{10} vp. Despite higher reactogenicity observed at the 5×10^{10} vp, this dose was safe, with self-limiting AEs and no serious adverse reactions recorded. The 5×10^{10} vp was the most immunogenic, in terms of inducing neutralizing antibodies against MERS-CoV using a live virus assay (Folegatti et al 2020a). Given the immunogenicity findings and safety profile observed with the ChAdOx1-vectored vaccine against MERS-CoV, the 5×10^{10} vp dose was chosen for AZD1222. See the AZD1222 IB.

Based on accumulating nonclinical and clinical data gathered for AZD1222 as well as for other SARS-CoV-2 vaccines in development, a 2-dose regimen was selected for the study in order to enhance the immune responses to the virus (AZD1222 IB).

In an NHP challenge study, 6 macaques received a second dose of AZD1222 4 weeks after the first dose. The second dose resulted in increases in both ELISA and neutralizing antibody titers, and fewer areas of the lung contained viral RNA in prime boost group compared to the prime group. In a porcine model, 3 pigs also received a second dose of AZD1222 4 weeks after the first dose. In the animals that received the booster dose, both antibodies to the SARS-CoV-2 RBD and neutralizing antibodies were boosted after the second dose.

In Study COV001, 10 participants received a second dose of AZD1222 4 weeks after the first dose. Antibody responses to both the first and second doses were evaluated using an ELISA assay, through the MSD platform and by both neutralization and pseudo-neutralization assays. Notable increases in antibody levels to the S protein were seen with the ELISA assay and increases to both the S protein and RBD were noted using the MSD platform. Similarly, increases in antibody levels following the second dose were also seen with neutralization and pseudo-neutralization assays. Though based on small numbers, the second dose of the vaccine appeared to result in a lower rate of systemic solicited AEs.

The 4-week interval was selected based on the interval used in the nonclinical studies and Study COV001.

4.4 End of Study Definition

A participant is considered to have completed the study if he/she has completed all phases of the study including the last scheduled procedure shown in the SoA (Section 1.3).

The end of the study is defined as the date of the last scheduled procedure shown in the SoA (Section 1.3) for the last participant in the study globally.

5 STUDY POPULATION

Prospective approval of protocol deviations to recruitment and enrollment criteria, also known as protocol waivers or exemptions, is not permitted.

5.1 Inclusion Criteria

Participants are eligible to be included in the study only if all of the following criteria apply:

Age

1 Adult, ≥ 18 years of age at the time of consent

Type of Participant

2 Increased risk of SARS-CoV-2 infection

– Defined as adults whose locations or circumstances put them at appreciable risk of exposure to SARS-CoV-2 and COVID-19, based on available risk assessment contemporaneous to enrollment (believed to be at risk/exposure)

3 Medically stable such that, according to the judgment of the investigator, hospitalization within the study period is not anticipated and the participant appears likely to be able to remain on study through the end of protocol-specified follow-up

– A stable medical condition is defined as disease not requiring significant change in therapy or hospitalization for worsening disease during the 3 months prior to enrollment

4 Able to understand and comply with study requirements/procedures (if applicable, with assistance by caregiver, surrogate, or legally authorized representative) based on the assessment of the investigator

Reproduction

5 Contraceptive use by women should be consistent with local regulations regarding the methods of contraception for those participating in clinical studies

6 Female participants

(a) Women of childbearing potential must:

- Have a negative pregnancy test on the day of screening and on Day 1
- Use one highly effective form of birth control for at least 28 days prior to Day 1 and agree to continue using one highly effective form of birth control through 60 days following administration of the second dose of study intervention.
  A highly effective method of contraception is defined as one that can achieve a failure rate of less than 1% per year when used consistently and correctly (see Table 6). Periodic abstinence, the rhythm method, and withdrawal are NOT acceptable methods of contraception.

(b) Women are considered of childbearing potential unless they meet either of the following criteria:

- Surgically sterilized (including bilateral tubal ligation, bilateral oophorectomy, or hysterectomy), or
- Post-menopausal
  - For women aged < 50 years, post-menopausal is defined as having both:
    - A history of ≥ 12 months amenorrhea prior to randomization, without an alternative cause, following cessation of exogenous sex-hormonal treatment, and
    - A follicle-stimulating hormone level in the post-menopausal range

    Until follicle-stimulating hormone is documented to be within menopausal range, the participant is to be considered of childbearing potential
  - For women aged ≥ 50 years, post-menopausal is defined as having a history of ≥ 12 months amenorrhea prior to randomization, without an alternative cause, following cessation of exogenous sex-hormonal treatment

Table 6 Highly Effective Methods of Contraception

Barrier Methods	Hormonal Methods
• Intrauterine device<br>• Intrauterine hormone-releasing system (IUS) [a]<br>• Bilateral tubal occlusion<br>• Vasectomized partner [b]<br>• Sexual abstinence [c]	• Combined (estrogen- and progestogen-containing hormonal contraception)<br>Oral (combined pill)<br>Intravaginal<br>Transdermal (patch)<br>• Progestogen-only hormonal contraception<br>Oral<br>Injectable<br>Implantable

a This is also considered a hormonal method
b Provided that partner is the sole sexual partner of the woman of childbearing potential study participant and that the vasectomized partner has received medical assessment of the surgical success
c Sexual abstinence is considered a highly effective method only if defined as refraining from heterosexual intercourse from 28 days prior to Day 1 through 60 days following administration of the second dose of study intervention, and if it is the preferred and usual lifestyle of the participant

Informed Consent

7 Capable of giving signed informed consent as described in Appendix A, which includes compliance with the requirements and restrictions listed in the ICF and in this protocol

5.2 Exclusion Criteria

Participants are excluded from the study if any of the following criteria apply:

Medical Conditions

1 History of allergy to any component of the vaccine

2 History of Guillain-Barré syndrome or any other demyelinating condition

3 Significant infection or other acute illness, including fever > 100 °F (> 37.8 °C) on the day prior to or day of randomization

4 History of laboratory-confirmed SARS-CoV-2 infection

5 Any confirmed or suspected immunosuppressive or immunodeficient state, including asplenia

6 Recurrent severe infections and use of immunosuppressant medication within the past 6 months (≥ 20 mg per day of prednisone or its equivalent, given daily or on alternate days for ≥ 15 days within 30 days prior to administration of study intervention)
The following exceptions are permitted:

- Topical/inhaled steroids or short-term oral steroids (course lasting ≤ 14 days)
- Human immunodeficiency virus-positive stable participants on stable antiretroviral therapy, eg, (NIH 2020, Waldrop et al 2016)

7 History of primary malignancy except for:

(a) Malignancy with low potential risk for recurrence after curative treatment (for example, history of childhood leukaemia) or metastasis (for example, indolent prostate cancer) in the opinion of the site investigator.

(b) Adequately treated non-melanoma skin cancer or lentigo maligna without evidence of disease

(c) Adequately treated uterine cervical carcinoma in situ without evidence of disease

(d) Localized prostate cancer

8 Clinically significant bleeding disorder (eg, factor deficiency, coagulopathy, or platelet disorder), or prior history of significant bleeding or bruising following IM injections or venepuncture

9 Severe and/or uncontrolled cardiovascular disease, respiratory disease, gastrointestinal disease, liver disease, renal disease, endocrine disorder, and neurological illness, as judged by the Investigator (mild/moderate well-controlled comorbidities are allowed)

10 Any other significant disease, disorder, or finding that may significantly increase the risk to the participant because of participation in the study, affect the ability of the participant to participate in the study, or impair interpretation of the study data
Note: The AESIs as outlined in Appendix E (including Table 14) should be considered when evaluating a participant for Exclusion Criterion 10, as the presence of these AESIs,

especially if untreated or uncontrolled, may be a safety risk to the participant, affect the ability of the participant to participate in the study, or impair interpretation of the study data. Investigators should review and consider the list of conditions in Appendix F. If any of these conditions are present in a participant, the Investigator is asked to utilize his/her clinical judgment in determining the participant's eligibility for the study. Should the participant have conditions as outlined in Appendix E and the participant is enrolled, the Investigator is asked to document notes on site regarding the final rationale for enrollment.

Prior/Concomitant Therapy

11 Receipt of, or planned receipt of investigational products indicated for the treatment or prevention of SARS-CoV-2 or COVID-19
Note: For participants who become hospitalized with COVID-19, receipt of licensed treatment options and/or participation in investigational treatment studies is permitted

12 Receipt of any vaccine (licensed or investigational) other than licensed influenza vaccines within 30 days prior to and after administration of study intervention

13 Receipt of immunoglobulins and/or any blood products within 3 months prior to administration of study intervention or expected receipt during the period of study follow-up

Other Exclusions

14 Involvement in the planning and/or conduct of this study (applies to both Sponsor staff and/or staff at the study site)

15 For women only - currently pregnant (confirmed with positive pregnancy test) or breast-feeding

16 Has donated ≥ 450 mL of blood products within 30 days prior to randomization or expects to donate blood within 90 days of administration of second dose of study intervention

5.3 Lifestyle Considerations

1 Participants must follow the contraception requirements outlined in Section 5.1

2 Restrictions relating to concomitant medications are described in Section 6.5

3 Agree to wear digital health device if diagnosed with COVID-19 as described in Section 8.1.2.2

5.4 Screen Failures

Screen failures are defined as participants who consent to participate in the clinical study but are not subsequently randomly assigned to study intervention. A minimal set of screen failure information is required to ensure transparent reporting of screen failure participants to meet the Consolidated Standards of Reporting Trials publishing requirements and to respond to

queries from regulatory authorities. Minimal information includes demography, screen failure details, eligibility criteria, and any SAEs.

Individuals who do not meet the criteria for participation in this study (screen failure) may be rescreened. Only a single rescreening is allowed in the study. Rescreened participants are required to sign a new ICF (Appendix A 3), and will be assigned a new participant number.

6 STUDY INTERVENTION

In general, study intervention is defined as any investigational intervention(s), marketed product(s), or placebo intended to be administered to or medical device(s) utilized by a study participant according to the study protocol. For this study, study intervention is defined as AZD1222 or saline placebo (Table 7). The third party medical device used for assessment of COVID-19 symptoms (ie, digital health device [Section 8.1.2.2]) is not considered a study intervention.

6.1 Study Interventions Administered

6.1.1 Investigational Products

Table 7 Investigational Products

Intervention Name	AZD1222	Placebo
**Type**	Vaccine	Placebo
**Dose Formulation**	10 mM histidine, 7.5% (w/v) sucrose, 35 mM sodium chloride, 1 mM magnesium chloride, 0.1% (w/v) polysorbate 80, 0.1 mM edetate disodium, 0.5% (w/v) ethanol, at pH 6.6.	0.9% (w/v) saline
**Unit Dose Strength(s)**	$\geq 0.7 \times 10^{11}$ vp/mL	
**Dosage Level(s)**	5×10^{10} vp (nominal, $\pm 1.5 \times 10^{10}$ vp)	
**Route of Administration**	Intramuscular	Intramuscular
**Use**	Experimental	Placebo
**IMP and NIMP**	IMP	IMP
**Sourcing**	Provided centrally by the Sponsor	Sourced locally
**Packaging and Labeling**	Will be provided in vials within a carton. Each carton and vial will be labelled as required per country requirement	Not applicable
**Current/Former Name or Alias**	ChAdOx1 nCoV-19	Not applicable

IMP = investigational medicinal product; NIMP = non-investigational medical product; vp = viral particles; w/v = weight/volume.

AZD1222

AZD1222 will be supplied by the Sponsor as a vial solution for injection. It is a sterile, clear to slightly opaque solution, practically free from visible particles, with a label-claim volume of 5 mL.

Unopened vials of AZD1222 vials must be stored at 2-8 °C (36-46 °F) for the duration of assigned shelf-life and must not be frozen. AZD1222 must be kept in original packaging until use to prevent prolonged light exposure.

Placebo

Commercially available 0.9% (w/v) saline for injection will be sourced locally for placebo.

6.1.2 Dosing Instructions

Participants will receive 2 doses of either AZD1222 or saline placebo. The first dose will be administered on Day 1 and the second dose on Day 29 (see Table 2 and Table 3).

It is recommended that the study interventions be administered as an IM injection into the deltoid of the non-dominant arm. Other injection sites may be used if necessary.

All study participants will be observed in the clinic for at least 15 minutes after vaccination.

Allergic reactions to vaccines are possible. Therefore, appropriate drugs and medical equipment to treat acute anaphylactic reactions must be immediately available, and study personnel must be trained to recognize and treat anaphylaxis.

6.2 Preparation/Handling/Storage/Accountability

1. The investigator or designee must confirm appropriate temperature conditions have been maintained during transit for all study intervention received and any discrepancies are reported and resolved before use of the study intervention.
2. Only participants enrolled in the study may receive study intervention and only authorized site staff may supply or administer study intervention. All study intervention must be stored in a secure, environmentally controlled, and monitored (manual or automated) area in accordance with the labelled storage conditions with access limited to the investigator and authorized site staff.
3. The investigator, institution, or the head of the medical institution (where applicable) is responsible for study intervention accountability, reconciliation, and record maintenance (ie, receipt, reconciliation, and final disposition records).
4. Further guidance and information for the final disposition of unused study interventions are provided in the Pharmacy Manual or specified handling instructions.

6.2.1 Dose Preparation and Administration

6.2.1.1 AZD1222

Doses of AZD1222 must be prepared by the unblinded pharmacist (or designee in accordance with local and institutional regulations) using aseptic technique. Each dose is prepared by withdrawing 0.5 mL from a vial of AZD1222 in a sterile syringe.

AZD1222 does not contain preservatives. Each vial must be assigned a beyond-use-date of 6 hours from first needle puncture of the AZD1222 vial, after which any unused portion must be discarded.

Once an AZD1222 dose is drawn into a syringe for administration, the dose must be administered within the beyond-use-date of the vial. If AZD1222 dose administration is not completed within the 6-hour vial beyond-use-date, a new dose must be prepared from a new vial.

Each vial of AZD1222 has a label-claim volume of 5 mL and can provide up to ten 0.5 mL doses.

6.2.1.2 Placebo

Doses of saline placebo must be prepared by the unblinded pharmacist (or designee in accordance with local and institutional regulations) using aseptic technique. Each saline placebo dose is prepared by withdrawing 0.5 mL from a 0.9% (w/v) saline vial or IV bag in a sterile syringe. If 0.9% (w/v) saline is extracted from IV bags, the manufacturers' recommendation for maximum number of needle punctures of the IV bag port must not be exceeded.

Saline (0.9% [w/v]) does not contain preservatives. Each vial or IV bag must be assigned a beyond-use-date of 6 hours from first needle puncture, after which any unused portion must be discarded.

Once a saline placebo dose is drawn into a syringe for administration, the dose must be administered within the beyond-use-date of the vial/IV bag. If saline placebo dose administration is not completed within the 6-hour vial/IV bag beyond-use-date, a new dose must be prepared from a new vial or IV bag.

6.3 Measures to Minimize Bias: Randomization and Blinding

6.3.1 Randomization

All participants will be centrally assigned to randomized study intervention using an IRT. Before the study is initiated, user guides, the log in information, and directions for the IRT will be provided to each study site. Randomization will be stratified by age (≥ 18 to

< 65 years, and ≥ 65 years), with at least 25% of participants to be enrolled in the older age stratum.

Where a participant does not meet all the eligibility criteria but incorrectly received study intervention, the investigator should inform the Study Physician immediately, and a discussion should occur between the Study Physician and the investigator regarding whether to continue or discontinue the participant.

6.3.2 Blinding

Neither the participant nor any of the investigators or Sponsor staff who are involved in the treatment or clinical evaluation and monitoring of the participants will be aware of the study intervention received. Since AZD1222 and saline placebo are visually distinct prior to dose preparation (due to differences in container closure), IMP will be handled by an unblinded pharmacist (or designee in accordance with local and institutional regulations) at the study site. Once drawn into syringes for administration, AZD1222 and saline placebo are not visually distinct from each other.

The IRT will provide the investigator(s) or pharmacists a dose tracking number to be allocated to the participant at the dispensing visit. Routines for this will be described in the IRT user manual that will be provided to each study site.

The randomization code should not be broken except in medical emergencies when the appropriate management of the participant requires knowledge of the treatment randomization. The investigator documents and reports the action to the Sponsor, without revealing the treatment given to participant to the Sponsor staff.

The Sponsor retains the right to break the code for SAEs that are unexpected and are suspected to be causally related to an investigational medicinal product and that potentially require expedited reporting to regulatory authorities. Randomization codes will not be broken for the planned analyses of data until all decisions on the evaluability of the data from each individual participant have been made and documented.

6.3.3 Procedures for Unblinding

The IRT will be programmed with blind-breaking instructions. In case of an emergency, in which the knowledge of the specific blinded study intervention will affect the immediate management of the participant's condition (eg, antidote available), the investigator has the sole responsibility for determining if unblinding of a participants' intervention assignment is warranted. Participant safety must always be the first consideration in making such a determination. If a participant's intervention assignment is unblinded for safety, the Sponsor must be notified within 24 hours after breaking the blind.

In the event that a study participant is contacted about receiving a licensed and/or authorized COVID-19 vaccine, unblinding instructions are being provided to the sites. If the participant is unblinded, the Sponsor needs to be notified within 24 hours and this should be documented in the site source documents. In addition, a participant information sheet has been developed for guidance on decision discussions between the participant and Investigator.

6.4 Study Intervention Compliance

When participants are dosed at the study site, they will receive study intervention directly from the investigator or designee, under medical supervision. The date, and time if applicable, of dose administered will be recorded in the source documents and recorded in the eCRF. The dose of study intervention and study participant identification will be confirmed at the time of dosing by a member of the study site staff other than the person administering the study intervention.

6.5 Concomitant Therapy

Any medication or vaccine (including over-the-counter or prescription medicines) that the participant is receiving at the time of enrollment or receives during the period specified in the SoA (Section 1.3), must be recorded in the eCRF along with the information listed below. Vitamins and/or herbal supplements are not to be recorded.

- Reason for use
- Dates of administration including start and end dates
- Dosage information including dose and frequency

The Study Physician should be contacted if there are any questions regarding concomitant or prior therapy.

6.5.1 Permitted Concomitant Medications

Participants may take concomitant medications prescribed by their primary care provider for management of chronic medical conditions and/or for health maintenance. Primary care providers, or where appropriate investigators, should prescribe appropriate concomitant medications or treatments deemed necessary to provide full supportive care and comfort during the study. Participants who develop COVID-19 after receiving study intervention should be treated with licensed medications and interventions according to standard of care. All routine vaccinations other than influenza are permitted beginning > 30 days after last dose of study intervention. Licensed influenza vaccines are permitted at any time.

6.5.2 Prohibited Concomitant Medications

The following medications are prohibited and the Sponsor must be notified if a participant receives any of these prohibited medications. The use of the following concomitant

medications and/or vaccines, however, will not definitively require withdrawal of the participant from the study, but may determine a participant's eligibility to receive a second dose or evaluability in the per-protocol analysis set.

If a participant receives a prohibited concomitant medication, the investigator in consultation with the Sponsor will evaluate any potential impact on receipt of study intervention based on time the medication was administered, the medication's pharmacology and pharmacokinetics, and whether the medication will compromise the participant's safety or interpretation of the data (see Section 7.1).

- Investigational products indicated for the treatment of SARS-CoV-2 or COVID-19
  Note: For participants who become hospitalized with COVID-19, receipt of licensed treatment options and/or participation in investigational treatment studies is permitted.
- Experimental vaccinations, other than AZD1222, for prevention of SARS-CoV-2 or COVID-19**.**

  Note: Participants choosing to receive a licenced and/or authorized COVID-19 vaccine should inform the Investigator so it can be properly documented. Participants, who receive a licenced and/or authorized COVID-19 vaccine outside the study, should be encouraged to continue study conduct to be followed for safety reporting and all assessments.
- Receipt of any vaccine (licensed or investigational) other than licensed influenza vaccines within 30 days prior to and after administration of study intervention (eg, Dose 1 and Dose 2). Thirty (30) days after the second vaccination, other routine vaccinations are permitted as clinically indicated.
- Glucocorticoids at a dose ≥ 20 mg/day of prednisone or equivalent given daily or on alternate days for ≥ 14 consecutive days between randomization and the participant's scheduled final visit
- Other systemically administered drugs with significant immunosuppressive activity, such as azathioprine, tacrolimus, cyclosporine, methotrexate, or cytotoxic chemotherapy between randomization and the participant's scheduled final visit
- Immunoglobulins and/or any blood product

6.6 Dose Modification

Study intervention will be administered as described in Section 6.1.2. Dose modification is not permitted.

6.7 Intervention After the End of the Study

There is no intervention after the end of the study (see definition in Section 4.4).

7 DISCONTINUATION OF STUDY INTERVENTION AND PARTICIPANT DISCONTINUATION/WITHDRAWAL

7.1 Discontinuation of Study Intervention

Each participant will receive 2 doses of study intervention (see Section 6). An individual participant will not receive the first or second dose of study intervention if any of the following occur in the participant in question:

1. Withdrawal of consent after signing informed consent
2. Participant meets one or more of the exclusion criteria or fails to meet all inclusion criteria for study participation
3. Laboratory-confirmed SARS-CoV-2 infection
4. Participant is pregnant or nursing
5. Any allergic reaction including anaphylaxis that is assessed as related to study intervention
6. Any SAE assessed as related to study intervention
7. Any AE that, in the judgment of the site investigator, is related to study intervention and may jeopardize the safety of the study participant
8. Receipt of a prohibited concomitant medication that may jeopardize the safety of the study participant or interpretation of the data

Each participant who has received at least one dose of study intervention will be followed for the full study period unless consent is withdrawn specifically from further study participation, or the participant is lost to follow-up. Participants who have not received study intervention, regardless of reason, will not be followed.

7.1.1 Study Suspension or Termination

The Sponsor reserves the right to temporarily suspend or permanently terminate this study or a component of the study at any time. The reasons for temporarily suspending the study may include, but are not limited, to the following:

- Any death, SAE, or other safety finding assessed as related to study intervention that in the opinion of the Sponsor may preclude further administration of study intervention

No additional participants will be randomized or treated with study intervention until review by the COVID-19 Vaccine DSMB is complete (see Appendix A 5 for COVID-19 Vaccine DSMB information).

7.2 Participant Withdrawal from the Study

- A participant may withdraw from the study at any time at his/her own request, or may be withdrawn at any time at the discretion of the investigator for safety, behavioral, compliance, or administrative reasons. This is expected to be uncommon.
- A participant who considers withdrawing from the study must be informed by the investigator about modified follow-up options (eg, telephone contact, a contact with a relative or treating physician, or information from medical records).
- If the participant withdraws consent for disclosure of future information, the Sponsor may retain and continue to use any data collected before such a withdrawal of consent.
- If a participant withdraws from the study, it should be confirmed if he/she still agrees for existing samples to be used in line with the original consent. If he/she requests withdrawal of consent for use of samples, destruction of any samples taken should be carried out in line with what was stated in the informed consent and local regulation. The investigator must document the decision on use of existing samples in the site study records and inform the Sponsor Study Team.

7.3 Lost to Follow-up

A participant will be considered lost to follow-up if he or she repeatedly fails to return for scheduled visits and is unable to be contacted by the study site.

The following actions must be taken if a participant fails to return to the clinic for a required study visit:

- The study site must attempt to contact the participant and reschedule the missed visit as soon as possible and counsel the participant on the importance of maintaining the assigned visit schedule and ascertain whether or not the participant wishes to and/or should continue in the study.
- Before a participant is deemed lost to follow-up, the investigator or designee must make every effort to regain contact with the participant (where possible, 3 telephone calls and, if necessary, a certified letter to the participant’s last known mailing address or local equivalent methods). These contact attempts should be documented in the participant’s medical record.
- Should the participant continue to be unreachable, he/she will be considered to have withdrawn from the study.

Discontinuation of specific sites or of the study as a whole are handled as part of Appendix A.

8 STUDY ASSESSMENTS AND PROCEDURES

- Study procedures and their timing are summarized in the SoA (Section 1.3). Protocol waivers or exemptions are not allowed.

- Immediate safety concerns should be discussed with the Sponsor immediately upon occurrence or awareness to determine if the participant should continue or discontinue study intervention.
- Adherence to the study design requirements, including those specified in the SoA (Section 1.3) is essential and required for study conduct.
- All screening evaluations must be completed and reviewed to confirm that potential participants meet all eligibility criteria. The investigator will maintain a screening log to record details of all participants screened and to confirm eligibility or record reasons for screening failure, as applicable.
- Procedures conducted as part of the participant's routine clinical management (eg, blood count) and obtained before signing of the ICF may be utilized for screening or baseline purposes provided the procedures met the protocol-specified criteria and were performed within the time frame defined in the SoA.

8.1 Efficacy Assessments

8.1.1 Monitoring COVID-19 Symptoms

Study sites will contact participants weekly (telephone/email/text) through Day 360 with reminders to monitor for COVID-19 symptoms. Participants who present with at least one of the COVID-19 qualifying symptoms listed below must contact the study team. With the exception of fever, shortness of breath, or difficulty breathing, the symptom must be present for 2 or more days. During the 7 days following administration of each dose of study intervention, investigator judgement should be used to determine which participants should initiate illness visits as symptoms may be due to the reactogenicity of the study intervention as opposed to potentially due to infection with SARS-CoV-2.

If a participant presents with a COVID-19 qualifying symptom(s) on Days 1-3, the nasal swab collected on Day 1 will be tested locally for SARS-CoV-2 (see Section 8.6.1.1). If positive, the participant will be instructed to initiate illness visits. If negative, the participant will continue with scheduled assessments per their assigned study (ie, main study [Table 2] or substudy [Table 3]).

Participants who present with a COVID-19 qualifying symptom(s) after Day 3 will be instructed to initiate illness visits and be tested for SARS-CoV-2.

COVID-19 Qualifying Symptoms

Participant must present with at least one of the following symptoms	
**Duration**	**Symptom**
No minimum duration	Fever
	Shortness of breath
	Difficulty breathing
Must be present for ≥ 2 consecutive days	Chills
	Cough
	Fatigue
	Muscle aches
	Body aches
	Headache
	New loss of taste
	New loss of smell
	Sore throat
	Congestion
	Runny nose
	Nausea
	Vomiting
	Diarrhea

Adapted from (CDC 2020).

8.1.2 Illness Visits

Symptomatic participants (as defined in Section 8.1.1) will be instructed to visit the study site for initiation of illness assessments (Table 4); where supported, home or mobile visits may be substituted for the site visits. Symptomatic participants will complete the Day 1 illness visit and will be instructed to continue with the home collection requirements (eg, digital health device and Illness e-Diary recordings, and saliva samples). SARS-CoV-2 RT-PCR results will be available during the home collection period and participants will be informed of their status. The results of the COVID-19 RT-PCR testing should also be reported to the participants' primary care providers. Only participants who test positive will be instructed to continue with the illness visits, including home collection of saliva samples and digital health device and Illness e-Diary recordings. Participants who test negative for SARS-CoV-2 will be instructed to stop all illness visit assessments and return the digital health device. Should symptoms persist despite a negative test result, Investigators should provide appropriate clinical follow-up of participants, as clinically indicated. Once Illness visits are discontinued,

participants will continue with follow-up visits per their assigned study (ie, main study [Table 2] or substudy [Table 3]).

8.1.2.1 SARS-CoV-2 Testing and Other Virology Assessments

At the Day 1 illness visit, mid-turbinate nasal swabs and nasopharyngeal swabs will be collected and tested for SARS-COV-2 by authorized RT-PCR assays (see Section 8.6.1.1).

Saliva will be collected during site illness visits and by self-collection at home throughout the illness visits to quantify duration of viral shedding (see Section 8.6.1.2). Other virology assessments are described in Section 8.6.

Genotypic evaluation of SARS-CoV-2 is planned at Day 1 and Day 14 illness visits but may be performed at Day 21 and Day 28 illness visits based upon logistical constraints, safety signals, or emerging data on efficacy of AZD1222 against viral variants.

8.1.2.2 Digital Health Device

At the Day 1 illness visit, participants will receive a wearable, digital health device (eg, Current Health Monitoring System) and be trained on use of the biosensor. The digital health device will continuously track biophysical parameters, including but not limited to, serial measurements of skin temperature, heart rate, respiratory rate, blood oxygen saturation, and physical activity.

Data will be obtained from the biosensor and transmitted via a wireless hub from the participant to the vendor platform. The vendor platform will be monitored remotely 24 hours a day by a team which includes registered nurses. The monitoring team will receive technical alerts if there are data or device issues, and physiologic alerts if participants' vital signs meet prespecified criteria. The physiologic alerts are intended to provide an early indication of worsening health status that would allow the monitoring team and investigator to provide appropriate follow-up. All alerts will be triaged and followed up by the monitoring team according to agreed-upon monitoring protocols. A registered nurse will review the alerts requiring clinical attention and will apply clinical judgement (based on the Schmitt-Thompson COVID-19 - Diagnosed or Suspected After Hours Telephone Triage Protocols) (Schmitt-Thompson 2020) to determine which alerts can be addressed directly with the participant via telephone, and which alerts should be escalated to the on-call clinician at the site. If escalation is required, the nurse will contact the on-call clinician by telephone and the clinician will then be responsible for subsequent follow-up. For all technical and physiologic alerts, details regarding the nature of the alert and action taken will be documented within the Current Health Monitoring System and an informational email will be sent to the investigator within 2 hours for further follow-up if required. If local regulations prohibit the monitoring team from collecting identifying information about participants, or from contacting study participants directly, the Investigator and site staff will be responsible for monitoring the transmitted data and responding to both technical and physiologic alerts 24 hours a day.

The data from the biosensor and remote monitoring are not intended to substitute for protocol-mandated standard safety monitoring, participant self-reporting, or participant oversight, which remains the responsibility of the investigator.

Along with the device, participants will be provided with a paper-based Quick Start Guide containing general instructions for the device as well as frequently asked questions. A reference copy of the document will be retained in the Site Master File.

Participants who test positive for SARS-CoV-2 will be instructed to continue wearing the digital health device until the COVID-19-associated symptoms resolve or until the Day 28 illness visit. Participants who test negative and stop the illness visits will be instructed to return the digital health device.

8.1.2.3 Illness e-Diary

An Illness e-Diary will be used to collect self-reported information about COVID-19-associated symptoms (listed below per CDC (CDC 2020). At the Day 1 illness visit, participants (or, if applicable, their caregiver, surrogate, or legally authorized representative) will be given access to the Illness e-Diary and trained by study staff on how to record the information and assess the severity of the symptoms.

Participants who test positive for SARS-CoV-2 will be instructed to continue recording in the Illness e-Diary until symptoms resolve or until the Day 28 illness visit. Participants who test negative will be instructed to stop Illness e-Diary recording.

Study sites will monitor the health status of participants via Illness e-Diary responses after the Day 1 illness visit, and will call participants as needed based on these responses.

COVID-19 Symptoms

- Fever
- Shortness of breath
- Difficulty breathing
- Chills
- Cough
- Fatigue
- Muscle aches
- Body aches
- Headache
- New loss of taste
- New loss of smell
- Sore throat
- Congestion
- Runny nose
- Nausea
- Vomiting
- Diarrhea

8.1.3 Severe COVID-19

The severity of COVID-19 will be evaluated in participants with symptoms of COVID-19. A diagnosis of severe or critical COVID-19 will include laboratory-confirmed COVID-19 (SARS-CoV-2 RT-PCR-positive symptomatic illness) plus any of the following:

- Clinical signs at rest indicative of severe systemic illness (respiratory rate ≥ 30 breaths per minute, heart rate ≥ 125 beats per minute, oxygen saturation ≤ 93% on room air at sea level, or partial pressure of oxygen to fraction of inspired oxygen ratio < 300 mm Hg)
- Respiratory failure (defined as needing high-flow oxygen, noninvasive ventilation, mechanical ventilation or extracorporeal membrane oxygenation)
- Evidence of shock (systolic blood pressure < 90 mm Hg, diastolic blood pressure < 60 mm Hg, or requiring vasopressors)
- Significant acute renal, hepatic, or neurologic dysfunction
- Admission to an intensive care unit
- Death

8.1.4 Medical Notes Review

With the participant's consent, the study team will request access to medical notes or submit a data collection form for completion by attending clinical staff on any medically-attended COVID-19 episodes. Any data relevant for assessment of the efficacy endpoints or vaccine-associated enhanced respiratory disease (see Section 8.3.9) will be collected. These are likely to include, but not limited to, information on intensive care unit admissions, clinical parameters such as oxygen saturation, respiratory rates and vital signs, need for oxygen therapy, need for ventilatory support, imaging, blood tests results, and overall outcome (survival or death).

8.1.5 Monitoring for Asymptomatic Infection

Blood samples will be collected according to the SoA (Section 1.3) for SARS-CoV-2 serology testing to monitor participants for asymptomatic infection. See description of assessment in Section 8.5.2.1.

8.2 Safety Assessments

Planned time points for all safety assessments are provided in the SoA (Section 1.3).

8.2.1 Physical Examinations

A complete physical examination will be performed at screening followed by targeted physical examinations as specified in the SoA (Section 1.3).

- A complete physical examination will include, but not be limited to, assessment of height, weight, general appearance, head, ears, eyes, nose, throat, neck, skin, as well as cardiovascular, respiratory, abdominal, and nervous systems. Each clinically significant abnormal finding at screening will be recorded in the medical history.
- A targeted physical examination will include areas suggested by the medical history. Each clinically significant abnormal finding following vaccination will be recorded as an AE.
- All physical examinations will be performed by a licensed healthcare provider (eg, physician, physician assistant, or licensed nurse practitioner).

8.2.2 Vital Signs

Vital signs, including heart rate, pulse oximetry, blood pressure, and body temperature, will be performed as specified in the SoA (Section 1.3). The participant should be resting prior to the collection of vital signs.

Data collected through the digital health device on heart rate, respiratory rate, temperature, and oxygen saturation level will be recorded as exploratory efficacy measurements and should not be reported as AEs unless resulting in an MAAE or SAE.

Situations in which vital sign results should be reported as AEs are described in Section 8.3.5.

8.2.3 Clinical Laboratory Assessments

A Laboratory Manual will be provided to the sites that specifies the procedures for collection, processing, storage, and shipment of samples, as well as laboratory contact information, specific to this clinical study.

For women participants of childbearing potential, a urine sample for pregnancy testing will be collected according to the SoA (Section 1.3). Urine pregnancy tests for β-hCG may be performed at the site using a licensed test (dipstick). If urine tests positive or indeterminate, a quantitative serum β-hCG will be performed for confirmation.

Additional safety samples may be collected if clinically indicated at the discretion of the investigator

8.3 Adverse Events and Serious Adverse Events

The principal investigator is responsible for ensuring that all staff involved in the study are familiar with the content of this section.

The definitions of an AE or SAE can be found in Appendix B.

All AEs are considered to be unsolicited AEs (collected by 'open question' at study visits) unless categorized as solicited AEs recorded in the substudy only.

Solicited AEs are local or systemic predefined events for assessment of reactogenicity. Solicited AEs will be collected in a Solicited AE e-Diary only for participants in the substudy (Section 8.3.7), and will be assessed separately from the (unsolicited) AEs collected during the study.

General information for AEs in this protocol excludes solicited AEs.

AEs will be reported by the participant (or, when appropriate, by a caregiver, surrogate, or the participant's legally authorized representative).

The investigator and any designees are responsible for detecting, documenting, and recording events that meet the definition of an AE.

8.3.1 Time Period and Frequency for Collecting AE and SAE Information

AEs will be recorded for 28 days post each dose of study intervention.

Solicited AEs will be recorded only for participants in the substudy for 7 days post each dose of study intervention.

SAEs will be recorded from the time of signature of the informed consent form through the last participant contact.

MAAEs and AESIs will be recorded from Day 1, post treatment, through the last participant contact.

See the SoA (Section 1.3) for the scheduled timepoints.

If the investigator becomes aware of an SAE with a suspected causal relationship to the study intervention that occurs after the end of the clinical study in a participant treated by him or her, the investigator shall, without undue delay, report the SAE to the Sponsor.

8.3.2 Follow-up of AEs and SAEs

Any AEs that are unresolved at the participant's last AE assessment in the study are followed up by the investigator for as long as medically indicated, but without further recording in the

eCRF. The Sponsor retains the right to request additional information for any participant with ongoing AE(s)/SAE(s) at the end of the study, if judged necessary.

AE variables

The following variables will be collected for each AE:

- AE (verbatim)
- Date when the AE started and stopped
- Severity grade/maximum severity grade/changes in severity grade
- Whether the AE is serious or not
- Investigator causality rating against the study intervention (yes or no)
- Action taken with regard to study intervention
- AE caused participant's withdrawal from study (yes or no)
- Outcome

In addition, the following variables will be collected for SAEs:

- Date AE met criteria for SAE
- Date investigator became aware of SAE
- AE is serious due to
- Date of hospitalization
- Date of discharge
- Probable cause of death
- Date of death
- Autopsy performed
- Causality assessment in relation to study procedure(s)
- Causality assessment to other medication

The grading scales from US FDA guidance for healthy volunteers enrolled in a preventive vaccine clinical study (FDA 2007) will be utilized for all unsolicited events with an assigned severity grading.

8.3.3 Causality Collection

The investigator should assess causal relationship between study intervention and each AE, and answer 'yes' or 'no' to the question 'Do you consider that there is a reasonable possibility that the event may have been caused by the investigational product?'

For SAEs, causal relationship should also be assessed for other medication and study procedures. Note that for SAEs that could be associated with any study procedure the causal relationship is implied as ‘yes.’

A guide to the interpretation of the causality question is found in Appendix B.

8.3.4 Adverse Events Based on Signs and Symptoms

All AEs spontaneously reported by the participant or reported in response to the open question from the study site staff: ‘Have you had any health problems since the previous visit/you were last asked?’, or revealed by observation will be collected and recorded in the eCRF. When collecting AEs, the recording of diagnoses is preferred (when possible) to recording a list of signs and symptoms. However, if a diagnosis is known and there are other signs or symptoms that are not generally part of the diagnosis, the diagnosis and each sign or symptom will be recorded separately.

8.3.5 Adverse Events Based on Examinations and Tests

The results from the Clinical Study Protocol-mandated vital signs will be summarized in the CSR.

Deterioration as compared to baseline in protocol-mandated vital signs should therefore only be reported as AEs if they fulfil any of the SAE or MAAE criteria or are considered to be clinically relevant as judged by the investigator (which may include but not limited to consideration as to whether treatment or non-planned visits were required).

If deterioration in a vital sign is associated with clinical signs and symptoms, the sign or symptom will be reported as an SAE or MAAE, and the associated vital sign will be considered as additional information.

8.3.6 Hy’s Law

Cases where a participant shows elevations in liver biochemistry may require further evaluation. Any occurrences of AST or ALT $\geq 3 \times$ ULN together with total bilirubin $\geq 2 \times$ ULN *and* confirmed as a Hy's Law case should be reported as an SAE.

Hy’s Law

AST or ALT $\geq 3 \times$ ULN together with TBL $\geq 2 \times$ ULN, where no other reason, other than the study intervention, can be found to explain the combination of increases, eg, elevated ALP indicating cholestasis, viral hepatitis, another drug. The elevation in transaminases must precede or be coincident with (ie, on the same day) the elevation in TBL, but there is no specified timeframe within which the elevations in transaminases and TBL must occur.

8.3.7 Solicited Adverse Events (Only for Substudy)

Local and systemic predefined solicited AEs for reactogenicity assessment (Table 8) will be collected in a Solicited AE e-Diary for 7 days following administration of each dose of study intervention only from participants in the substudy.

Solicited AEs should not be reported as unsolicited AEs (see Section 8.3). However, solicited AEs should be reported as SAEs or MAAEs if they fulfil the criteria (see Sections 8.3 and 8.3.8, respectively).

Table 8 List of Predefined Solicited Adverse Events for Reactogenicity Assessment

Local	Systemic
Pain at the site of injection	Fever (> 100 °F [> 37.8 °C]) [a]
Erythema/redness at the site of injection [b]	Chills
Tenderness	Muscle pains
Induration/swelling at the site of the injection [b]	Fatigue
	Headache
	Malaise
	Nausea
	Vomiting

[a] Fever measured by any route. Investigators who consider a temperature lower than this cutoff as a fever or a 'fever' reported by participants without documentation by a thermometer should record the event as 'elevated body temperature.'

[b] Swelling and redness must be ≥ 0.25 inches (≥ 0.6 centimeters) in diameter.

Solicited AE e-Diary

On Day 1, participants in the substudy (or, if applicable, their caregiver, surrogate, or legally authorized representative) will be given an oral thermometer, tape measure, and access to the Solicited AE e-Diary, with instructions on use, along with the emergency 24-hour telephone number to contact the on-call study physician if needed.

Participants will be instructed to record for 7 days following administration of each dose of study intervention, the timing and severity of local and systemic solicited AEs, if applicable, and whether medication was taken to relieve the symptoms.

Severity Assessment of Solicited AEs

Severity will be assessed for solicited AEs by the participant (or, if applicable, their caregiver, surrogate, or legally authorized representative) according to toxicity grading scales modified and abridged from the US FDA guidance (FDA 2007) as defined in Appendix D. Because

solicited AEs are expected to occur after vaccination, they will not be assessed for relationship to study intervention.

8.3.8 Medically Attended Adverse Events

MAAEs will be collected according to the timepoints specified in the SoA (Section 1.3).

MAAEs are defined as AEs leading to medically-attended visits that were not routine visits for physical examination or vaccination, such as an emergency room visit, or an otherwise unscheduled visit to or from medical personnel (medical doctor) for any reason. AEs, including abnormal vital signs, identified on a routine study visit or during the scheduled illness visits will not be considered MAAEs.

8.3.9 Adverse Events of Special Interest

AESIs will be collected according to the timepoints specified in the SoA (Section 1.3).

AESIs are events of scientific and medical interest specific to the further understanding of study intervention safety profile and require close monitoring and rapid communication by the investigators to the Sponsor. AESIs for AZD1222 are based on Brighton Collaboration case definitions (SPEAC 2020), clinical experience, and scientific interest. See Appendix E for a listing and description of AZD1222 AESIs.

An AESI can be serious or non-serious. All AESIs will be recorded in the eCRF. If any AESI occurs in the course of the study, investigators or other site personnel will inform the appropriate Sponsor representatives within one day ie, immediately but **no later than 24 hours** of when he or she becomes aware of it. Serious AESIs will be recorded and reported as per Section 8.3.10.

8.3.9.1 Potential Neurological AESIs

If a participant experiences new onset (acute or subacute) motor and sensory disturbances (eg, weakness, numbness, paresthesias, hypoesthesia, hyperesthesia, dysesthesias), bowel/bladder dysfunction, gait impairment, visual disturbance, or any event of myelitis, encephalomyelitis, transverse myelitis, or other sudden neurological deficit, there should be prompt neurological evaluation, including referral to a neurology specialist for further evaluation and testing, as clinically indicated. Testing can include evaluation for peripheral demyelinating conditions (eg, EMG). In cases of concern for spinal cord disease, see Figure 2 for a recommended testing algorithm.

An independent Neurological AESI Expert Committee will review and provide advice on the diagnosis and causality assessment of selected neurological AESIs occurring in the AZD1222 clinical development program (see Appendix A 5).

Figure 2 Neurology Testing Algorithm

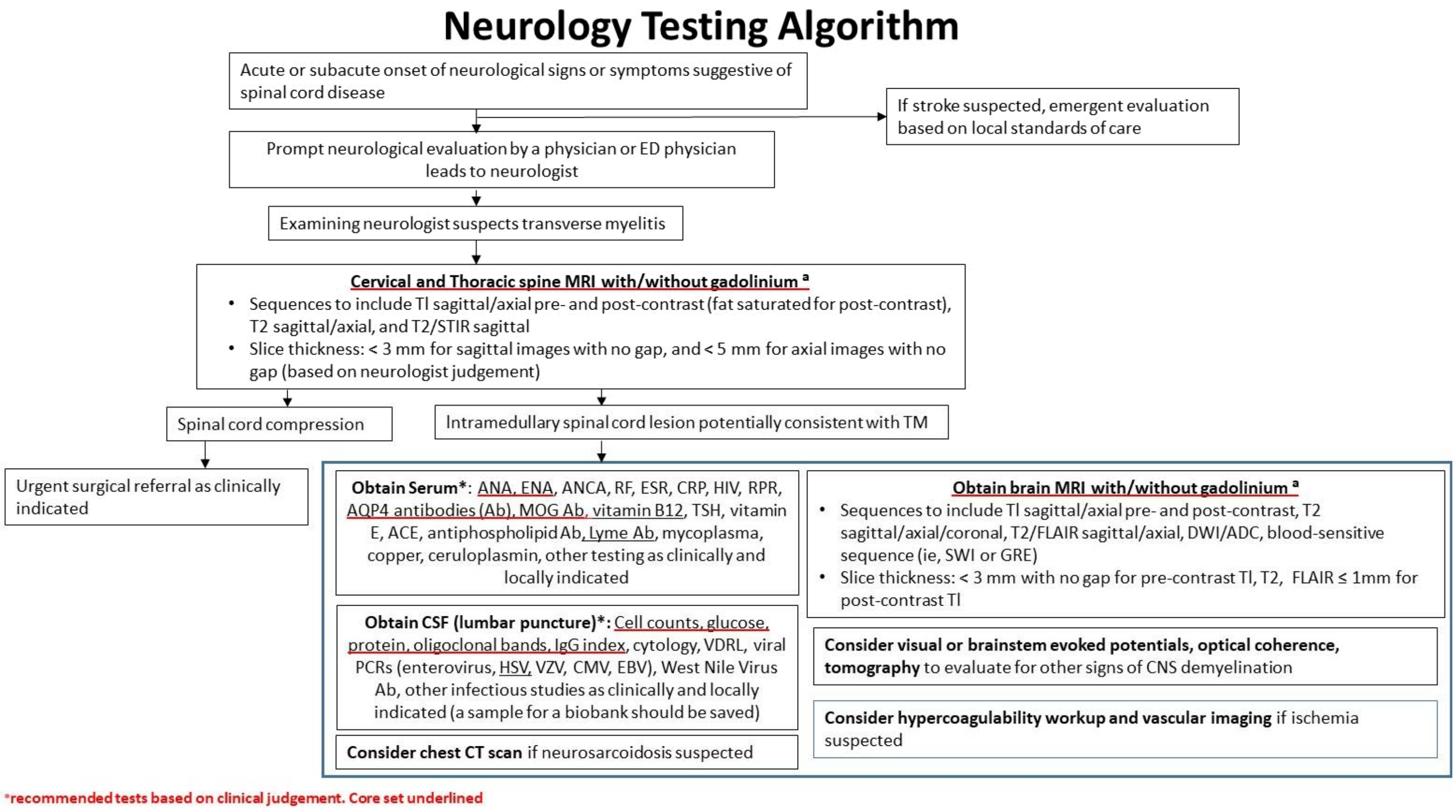

[a] Adapted from (Rovira et al 2015)

Ab = antibody; ACE = angiotensin converting enzyme; ADC = apparent diffusion coefficient; ANA = antinuclear antibody; ANCA = antineutrophil cytoplasmic antibodies; AQP4 = aquaporin 4; CMV = cytomegalovirus; CNS = central nervous system; CRP = c-reactive protein; CSF = cerebral spinal fluid; CT = computed tomography; DWI = diffusion-weighted image; EBV = Epstein-Barr virus; ED = emergency department; ENA = extractable nuclear antigen antibodies; ESR = erythrocyte sedimentation rate; FLAIR = fluid-attenuated inversion recovery; GRE = gradient echo; HIV = human immunodeficiency virus; HSV = herpes simplex virus; IgG = immunoglobulin G; MOG = myelin oligodendrocyte glycoprotein; MRI = magnetic resonance image; PCR = polymerase chain reaction; RF = rheumatoid factor; RPR = rapid plasma reagin; STIR = short T1 inversion recovery; SWI = susceptibility-weighted imaging; TSH = thyroid stimulating hormone; TM = transverse myelitis; VDRL = Venereal Disease Research Laboratories; VZV = varicella-zoster virus.

8.3.10 Reporting of Serious Adverse Events

All SAEs have to be reported, whether or not considered causally related to the study intervention, or to the study procedure(s). All SAEs will be recorded in the eCRF.

If any SAE occurs in the course of the study, investigators or other site personnel will inform the appropriate Sponsor representatives within one day ie, immediately but **no later than 24 hours** of when he or she becomes aware of it.

The designated Sponsor representative will work with the investigator to ensure that all the necessary information is provided to the AstraZeneca Patient Safety data entry site **within one calendar day** of initial receipt for fatal and life-threatening events **and within 5 calendar days** of initial receipt for all other SAEs.

For fatal or life-threatening AEs where important or relevant information is missing, active follow-up will be undertaken immediately. Investigators or other site personnel will inform Sponsor representatives of any follow-up information on a previously reported SAE within one calendar day, ie, immediately but no later than 24 hours of when he or she becomes aware of it.

Once the investigators or other site personnel indicate an AE is serious in the EDC system, an automated email alert is sent to the designated Sponsor representative.

If the EDC system is not available, then the investigator or other study site staff reports an SAE to the appropriate Sponsor representative by telephone or other method.

The Sponsor representative will advise the investigator/study site staff how to proceed.

For further guidance on the definition of an SAE, see Appendix B.

The reference document for definition of expectedness is the AZD1222 IB, Section 5.6.

8.3.11 Pregnancy

All pregnancies and outcomes of pregnancy with conception dates following administration of study intervention should be reported to the Sponsor, except if the pregnancy is discovered before the participant has received any study intervention.

8.3.11.1 Maternal Exposure

Female participants who are pregnant or have a confirmed positive pregnancy test at screening or Day 1 will be excluded from the study (see Section 5.2). Pregnancy itself is not regarded as an AE unless there is a suspicion that the study intervention may have interfered with the effectiveness of a contraceptive medication. Congenital abnormalities/birth defects and spontaneous miscarriages should be reported and handled as SAEs. Elective abortions without

complications should not be handled as AEs. The outcome of all pregnancies (spontaneous miscarriage, elective termination, ectopic pregnancy, normal birth or congenital abnormality) should be followed up and documented even if the participant was discontinued from the study.

If any pregnancy occurs in the course of the study, then the investigator or other site personnel informs the appropriate Sponsor representatives within **1 day**, ie, immediately but **no later than 24 hours** of when he or she becomes aware of it.

The designated Sponsor representative works with the investigator to ensure that all relevant information is provided to the AstraZeneca Patient Safety data entry site **within 1 or 5 calendar days** for SAEs (see Section 8.3.10) and **within 30 days** for all other pregnancies that are not associated with an SAEs.

The same timelines apply when outcome information is available.

The PREGREP module in the eCRF is used to report the pregnancy and the paper-based PREGOUT module may be used to report the outcome of the pregnancy.

8.3.12 Medication Error

If a medication error occurs, then the investigator or other site personnel informs the appropriate Sponsor representatives within **1 day**, ie, immediately but **no later than 24 hours** of when he or she becomes aware of it.

The designated Sponsor representative works with the investigator to ensure that all relevant information is completed within **1** (Initial Fatal/Life-Threatening or follow up Fatal/Life-Threatening) **or 5** (other serious initial and follow up) **calendar days** if there is an SAE associated with the medication error (see Section 8.3.10) and **within 30 days** for all other medication errors.

The definition of a Medication Error can be found in Appendix B 4.

8.3.13 Medical Device Deficiencies

Any deficiency observed with the digital health device (third-party medical device) will be collected and reported to the manufacturer by the investigators or other site personnel within one day ie, immediately but no later than 24 hours of when he or she becomes aware of it.

A medical device deficiency is an inadequacy of a medical device with respect to its identity, quality, durability, reliability, safety, or performance. Medical device deficiencies include malfunctions, use errors, and information supplied by the manufacturer.

The manufacturer's medical device complaint report will be used to collect the deficiency.

8.4 Overdose

For this study, any dose of study intervention exceeding that specified in the protocol will be considered an overdose.

There is no specific treatment for an overdose with AZD1222. If overdose occurs, the participant should be treated supportively with appropriate monitoring as necessary.

- An overdose with associated AEs is recorded as the AE diagnosis/symptoms on the relevant AE modules in the eCRF and on the Overdose eCRF module
- An overdose without associated symptoms is only reported on the Overdose eCRF module

If an overdose occurs in the course of the study, the investigator or other site personnel inform appropriate Sponsor representatives immediately, but **no later than 24 hours** after when he or she becomes aware of it.

The designated Sponsor representative works with the investigator to ensure that all relevant information is provided to the AstraZeneca Patient Safety data entry site **within one or 5 calendar days** for overdoses associated with an SAE (see Section 8.3.10) and **within 30 days** for all other overdoses.

8.5 Human Biological Samples

Instructions for the collection and handling of biological samples will be provided in the study-specific laboratory manual. Samples should be stored in a secure storage space with adequate measures to protect confidentiality. Further details on Handling of Human Biological Samples are provided in Appendix C.

Samples will be stored for a maximum of 15 years from the date of the issue of the CSR in line with consent and local requirements, after which they will be destroyed/repatriated.

Remaining biological sample aliquots will be retained at the Sponsor or its designee for a maximum of 15 years following issue of the CSR. Additional use excludes genetic analysis and includes but is not limited to, analysis of COVID-19 and other coronavirus-related diseases or vaccine-related responses, eg, exploratory immunology, such as systems serology and profiling of B- and T-cell repertoire. The results from further analysis will not be reported in the CSR.

8.5.1 Pharmacokinetics

Pharmacokinetic parameters are not evaluated in this study.

8.5.2 Immunogenicity Assessments

Serum samples for immunogenicity assessments will be collected according to the SoA (Section 1.3). Samples will be collected, labelled, stored, and shipped as detailed in the Laboratory Manual. Results for exploratory immunogenicity analyses may be reported separately from the CSR.

8.5.2.1 SARS-CoV-2 Serology Assessments

Serum samples will be collected to assess SARS-CoV-2 antigen-specific antibody levels from all participants according to the SoA (Section 1.3). Authorized laboratories will assess serologic responses to AZD1222 by the rate of participants seroconverting from negative to positive as defined by a validated immunoassay directed at the SARS-CoV-2 S antigen. The rate of asymptomatic SARS-CoV-2 infection in participants receiving AZD1222 vs saline placebo will be determined by seroconversion in a SARS-CoV-2 Nucleocapsid assay operated by an authorized laboratory. Serologic assessment to S, RBD, and Nucleocapsid antigens will also be assessed quantitatively using a validated multiplexed MSD immunoassay.

8.5.2.2 SARS-CoV-2 Neutralizing Antibody Assessments

Serum samples to measure SARS-CoV-2 neutralizing antibody levels will be collected from participants in the substudy according to the timepoints specified in the SoA (Section 1.3). Authorized laboratories will measure neutralizing antibodies to SARS-CoV-2 using validated wild-type neutralization assay or pseudo-neutralization assays.

8.5.2.3 Assessment of Mucosal Responses

Nasal samples to evaluate SARS-CoV-2 antigen-specific antibody responses in nasal secretions will be collected from participants in the substudy, from participants enrolled in the immunogenicity cohort, and during illness visits according to the SoA (Section 1.3). Nasal adsorption specimens will be collected by synthetic absorptive matrix sampling as outlined in the laboratory manual. Antibody responses to SARS-CoV-2 S, RBD, and Nucleocapsid antigens may be assessed in a qualified multiplexed MSD immunoassay and stratified by antibody isotype (IgA, IgG, IgM).

8.5.2.4 Assessment of Cell-mediated Immune Responses

Cell-mediated immune responses (ie, B-cell and T-cell responses) will be assessed by characterizing PBMCs using methods that may include T-cell ELISpot assays to SARS-CoV-2 antigens, flow cytometry after intracellular cytokine staining, single-cell RNA sequencing, B-cell and T-cell receptor sequencing, and other methodology as determined by the Sponsor and/or authorized laboratories. Data on Th1/Th2 polarization after AZD1222 vaccination will be provided and may be reported separately from the CSR. Samples will be collected from up to 300 participants in the substudy, from approximately 300 participants in the immunogenicity cohort, and from up to approximately the first 3 000 participants, where

operationally feasible, during the Day 1 illness visit, as well as participants who have a positive SARS-CoV-2 RT-PCR result at timepoints specified in the SoA (Section 1.3).

Additionally, plasma will be isolated from the whole blood samples collected to isolate PBMCs, which may be utilized for exploratory immunogenicity and biomarker analyses as outlined in Section 8.6.2.

8.5.2.5 Additional Serum Immunogenicity

Additional serum samples for exploratory immunogenicity evaluation will be obtained according to the SoA (Section 1.3). Serologic assessment to seasonal coronavirus antigens will also be assessed quantitatively using a qualified multiplexed MSD immunoassay, while anti-vector immune responses (ie, ChAdOx1 neutralizing antibody responses) will characterize the induction of antibodies to the ChAdOx1vector and the persistence of these antibodies over time. Exploratory sera samples may be utilized to investigate additional humoral and cellular immune responses as well as potential correlates of protection as determined by the Sponsor and/or authorized laboratories based upon emerging safety, efficacy, and immunogenicity data.

8.5.3 Pharmacodynamics

Pharmacodynamics are not evaluated in this study.

8.6 Human Biological Sample Biomarkers

8.6.1 Collection of Mandatory Samples for Biomarker Analysis

By consenting to participate in the study, the participant consents to participate in the mandatory research components of the study.

Samples for biomarker research are required and will be collected from participants during illness visits as specified in the SoA (Section 1.3). Mid-turbinate nasal and nasopharyngeal swabs will be collected at site illness visits for virologic assessments. Saliva samples will be collected at site illness visits and by the participants during the home-collection period. These biomarker measurements will support our understanding of potential correlates of protection, duration of immune responses, and correlations between immunogenicity and reactogenicity. Details for sample collection, processing, and testing will be provided in the Laboratory Manual.

Any results from such analyses may be reported separately from the CSR.

8.6.1.1 Virologic Assessments

Instructions for obtaining and processing mid-turbinate nasal swabs and nasopharyngeal swab samples are provided in the Laboratory Manual. Mid-turbinate nasal and nasopharyngeal swabs will be assessed by authorized RT-PCR assays for the detection of SARS-CoV-2 by

local and central laboratories, respectively. Genotypic analysis of the viral S protein will be performed by next generation sequencing modalities. Additionally, a validated multiplexed respiratory panel may be utilized to assess for the presence of other respiratory pathogens in nasopharyngeal swabs in a central laboratory operated on behalf of the Sponsor.

8.6.1.2 Assessment of Viral Shedding

Viral shedding will be assessed in saliva samples collected using a Spectrum DNA (SDNA-1000) collection kit or other saliva collection devices at site illness visits or self-collected at home, by an authorized RT-PCR assay for the measurement of SARS-CoV-2. Collection of saliva sample for viral shedding is optional in non-USA sites and may occur after a feasibility assessment has been completed.

8.6.2 Other Study-related Biomarker Research

Already collected samples may be analyzed for different biomarkers thought to play a role in COVID-19 severity or outcomes including, but not limited to serum, plasma or mucosal cytokines, quantification of RNA, micro-RNA, and/or non-coding RNA using quantitative RT-PCR, microarray, sequencing, or other technology in blood, PBMCs, or mucosal specimens to evaluate their association with observed clinical responses to AZD1222. Other study-related biomarker research excludes genetic analysis.

For storage, re-use, and destruction of biomarker samples see Section 8.5.

8.7 Optional Genomics Initiative Sample

Optional Genomics Initiative research is not applicable anymore to this study.

8.8 Medical Resource Utilization and Health Economics

Medical resource utilization and health economics are not applicable in this study.

9 STATISTICAL CONSIDERATIONS

9.1 Statistical Hypotheses

The primary efficacy endpoint is a binary response, whereby a participant is defined as a COVID-19 case if their first case of SARS-CoV-2 RT-PCR-positive symptomatic illness occurs ≥ 15 days post second dose of study intervention. Otherwise, a participant is not defined as a COVID-19 case. VE will be calculated as 1-relative risk, which is the incidence in the vaccine group relative to the incidence in the control group. The null hypothesis is: VE is equal to 30%. Whereas, the alternative hypothesis is: VE is not equal to 30%. That is:

- Null hypothesis: VE = 30%
- Alternative hypothesis: VE ≠ 30%

The primary efficacy endpoint will be formally assessed at 2 time points during the study, giving an interim analysis and a primary analysis. A Lan-DeMets alpha-spending function has been used to control the overall type I error at 5% with 0.31% alpha at the interim and 4.9% at the primary analysis. At the interim analysis, the VE will be presented with a 2-sided 99.69% CI, and statistical significance will be achieved if the 2-sided 99.69% CI is > 30%. The success criterion for the interim analysis will be statistical significance with an observed VE point estimate of at least 50%. At the primary analysis VE will be presented with a 2-sided 95.10% CI, and statistical significance will be achieved if the 2-sided 95.10% CI is > 30%. The success criterion for the primary analysis of the study will be statistical significance with an observed VE point estimate of at least 50%.

In the event that the results of the interim or primary analysis are statistically significant for the primary efficacy endpoint, an additional analysis with 5% alpha will be performed once all participants have completed their first year of follow up.

9.2 Sample Size Determination

Approximately 33 000 participants will be screened such that approximately 30 000 participants will be randomized in a 2:1 ratio to receive 2 IM doses of either 5×10^{10} vp (nominal, $\pm 1.5 \times 10^{10}$ vp) AZD1222 (the active group, n = approximately 20 000) or saline placebo (the control group, n = approximately 10 000) 4 weeks apart.

Note: 'Enrolled' means a participant's, or their legally acceptable representative's, agreement to participate in a clinical study following completion of the informed consent process. Potential participants who are screened for the purpose of determining eligibility for the study, but are not randomly assigned/assigned in the study, are considered 'screen failures', unless otherwise specified by the protocol.

The sample size calculations are based on the primary efficacy endpoint and were derived following a modified Poisson regression approach (Zou 2004). The calculations account for an interim and primary analysis, and the timing of these analyses will be driven by the number of events observed in the study. The interim analysis will be carried out when approximately 50% of the total amount of statistical information is available. A Lan-DeMets alpha-spending function has been used to account for multiplicity, where the Type I error at the interim and primary analyses is with 0.31% alpha at the interim analysis and 4.9% at the primary analysis such that the overall Type I error is controlled at 5%. The calculations assume minimal loss to follow-up as it is anticipated that participants will remain engaged in the study. All participants will be followed for the entire duration of the study.

For the primary efficacy analysis, approximately 150 events meeting the primary efficacy endpoint definition are required across the active and control groups within the population of participants who are seronegative at baseline to detect a VE of 60% with > 90% power. These

calculations assume an observed attack rate of approximately 0.8% and are based on a 2-sided test, where the lower bound of the 2-sided 95.10% CI for VE is required to be greater than 30% with an observed point estimate of at least 50%.

An interim efficacy analysis will be conducted when approximately 75 events meeting the primary efficacy endpoint definition have been reported across the active and control groups within the population of participants who are seronegative at baseline, which will give > 70% power to detect a VE of 70% and > 90% power to detect a VE of 75%. These calculations assume an observed attack rate of approximately 0.4% and are based on a 2-sided test, where the lower bound of the 2-sided 99.69% CI for VE is required to be greater than 30% with an observed point estimate of at least 50%. A statistically significant finding at the interim analysis will not be considered a reason to stop the study, but instead will be interpreted as early assessment of efficacy.

9.3 Populations for Analyses

The following populations are defined:

Table 9 Populations for Analysis

Population	Description
All participants analysis set	All participants screened for the study, to be used for reporting disposition and screening failures.
Full analysis set	All randomized participants who received at least one dose of study intervention, irrespective of their protocol adherence and continued participation in the study. Participants will be analyzed according to their randomized treatment irrespective of whether or not they have prematurely discontinued, according to the intent-to-treat principle. Participants who withdraw consent or assent to participate in the study will be included up to the date of their study withdrawal.
Fully vaccinated analysis set	The fully vaccinated analysis set will include all participants in the full analysis set who are seronegative at baseline, receive 2 doses of study intervention, and who remain on-study 15 days after their second dose without having had a prior SARS-CoV-2 RT-PCR-positive confirmed COVID-19 infection.
Per-protocol analysis set	The per-protocol analysis set will include participants in the fully vaccinated analysis set who receive the correct dose of randomized treatment and who do not have a serious protocol deviation. Detailed criteria defining this analysis set will be documented in the Statistical Analysis Plan.<br>Erroneously-treated participants who receive one dose of active study intervention and one dose of saline placebo, regardless of the sequence, will be excluded from this analysis set.

Table 9 Populations for Analysis

Population	Description
Safety analysis set	The safety analysis set consists of all participants who have received at least one dose of study intervention.<br>Erroneously-treated participants (eg, those randomized to treatment A but were actually given treatment B) are accounted for in this analysis set by assigning them to the treatment they actually received.<br>A participant who has on one or several occasions received active study intervention is classified as active for all summaries, including summaries by dose.
Immunogenicity analysis set	The immunogenicity analysis population will include all participants in the safety analysis set who have no protocol deviations judged to have the potential to interfere with the generation or interpretation of an immune response. Examples of protocol violations will be documented in the Statistical Analysis Plan.

COVID-19 = coronavirus disease 2019; RT-PCR = reverse transcriptase polymerase chain reaction; SARS-CoV-2 = severe acute respiratory syndrome-coronavirus 2.

9.4 Statistical Analyses

The primary DBL will occur after approximately 150 events have been observed for the primary endpoint (see Section 9.2) within the population of participants who are seronegative at baseline. All participants in the study will be assessed for efficacy and safety for 2 years following the first dose of study intervention (Day 730). The final DBL will occur when all participants have completed the study.

This section is a summary of the planned statistical analyses of the most important endpoints, including primary and key secondary endpoints. A more technical and detailed description of the statistical analyses will be described in the SAP, and an approved version will be finalized prior to the interim analyses (see Section 9.5).

All personnel involved in the analyses of the study will remain blinded until the primary DBL and protocol deviations are identified, unless the DSMB determines that the efficacy and safety have been definitively established during interim analysis. In this case, the DSMB will notify the Oversight Group who will then determine the course of action for the study, which may include unblinding personnel from the Sponsor or its representatives associated with the analyses and regulatory submission of interim results.

Analyses will be performed by the Sponsor or its representatives.

Categorical variables will be summarized using frequency and percentages, where the denominator for calculation is the underlying analysis set population unless otherwise stated.

Continuous variables will be summarized with descriptive statistics of number of available observations, mean, standard deviation, median, minimum and maximum, and quartiles where more appropriate.

All point estimates will be presented together with a 95% CI, unless otherwise stated. P-values, corresponding to a 2-sided test, will be presented for comparisons between treatments. Methods for controlling multiplicity across endpoints are discussed in Section 9.4.4.

9.4.1 General Considerations

The primary efficacy analysis will be based on the double-blind, placebo-controlled phase of the study, and will compare participants randomized to receive 2 nominal doses of 5×10^{10} vp ($\pm 1.5 \times 10^{10}$ vp) AZD1222 against participants randomized to saline placebo.

The primary estimand will be used for the analysis of the primary efficacy endpoint. It will be based on participants in the fully vaccinated analysis set, defined as all randomized participants who are seronegative at baseline, receive 2 doses of study intervention, and who remain on-study 15 days after their second dose without having had a prior SARS-CoV-2 RT-PCR-positive confirmed COVID-19 infection, analyzed according to their randomized treatment. For participants with multiple events, only the first occurrence will be used for the primary efficacy endpoint analysis. The set of intercurrent events for this estimand consists of participants who withdraw from the study or are unblinded to treatment assignment prior to having met the primary efficacy endpoint. The intercurrent event of early study withdrawal will be handled using the treatment policy strategy and the absence of data following these participants' withdrawal will be treated as missing (ie, counted as not having met the criteria). Participants unblinded to treatment assignment prior to having met the criteria for the primary efficacy endpoint will be censored at the date of unblinding. Participants who withdraw before 15 days post second dose or who have a case prior to 15 days post second dose will be excluded from primary endpoint analysis.

Additional estimands will be specified for the primary efficacy endpoint to carry out sensitivity analyses for assessing the robustness of results. These sensitivity analyses will explore different methods for handling intercurrent events and different assumptions for missing data. Estimands will also be specified for the analysis of secondary endpoints. Full details will be provided in the SAP.

Demography and baseline characteristics will be summarized by treatment for the fully vaccinated analysis set, safety analysis set, and immunogenicity analysis set.

9.4.2 Efficacy

An overview of the primary and secondary efficacy objectives, endpoints, and the associated case definitions is presented in Appendix F.

A blinded independent efficacy adjudication committee will review relevant data of potential cases for the COVID-19-related efficacy endpoint evaluations. More detail on this process will be provided in the SAP and the Efficacy Adjudication Committee Charter (see also Appendix A 5).

9.4.2.1 Primary Endpoint

The primary endpoint is the first case of SARS-CoV-2 RT-PCR-positive symptomatic illness occurring ≥ 15 days post second dose of study intervention, in a participant with negative serostatus at baseline. Participants will be included in the primary endpoint if they have RT-PCR-confirmed SARS-CoV-2 and meet the following criteria at any point from their initial illness visit at the site (Day 1) through their second illness visit (Day 14):

1 One or more Category A findings

OR

2 Two or more Category B findings

Category A:

- Pneumonia diagnosed by chest x-ray, or computed tomography scan
- Oxygen saturation of ≤ 94% on room air or requiring either new initiation or escalation in supplemental O_2
- New or worsening dyspnea/shortness of breath

Category B:

- Fever > 100 °F (> 37.8 °C) or feverishness
- New or worsening cough
- Myalgia/muscle pain
- Fatigue that interferes with activities of daily living
- Vomiting and/or diarrhea (only one finding to be counted toward endpoint definition)
- Anosmia and/or ageusia (only one finding to be counted toward endpoint definition)

The primary efficacy endpoint will be assessed at 2 milestones during the study, giving an interim analysis (see Section 9.5) and a primary analysis. The timing of these analyses will be driven by the number of events observed in the study within the subset of participants who are seronegative at baseline. The interim analysis will be carried out when approximately

75 events meeting the primary efficacy endpoint definition have been observed, and the primary analysis will be carried out when approximately 150 events meeting the primary efficacy endpoint definition have been observed (see Section 9.2). A final analysis will also be carried out when all participants have completed the 2-year study. However, the final analysis will not be controlled for multiplicity and statistical hypotheses will be tested at a nominal 5% significance level (based on a 2-sided test).

As the primary efficacy analysis, the plan is to use the primary estimand and a Poisson regression model with robust variance (Zou 2004) to analyze the primary efficacy endpoint, including study arm and age as covariates as well as the log of the follow-up time as an offset. The VE will be estimated from the model, which will give the RRR in the incidence of SARS-CoV-2 RT-PCR-positive symptomatic illness. VE is calculated as RRR = 100*(1-relative risk), which is the incidence in the vaccine group relative to the incidence in the control group expressed as a percentage. For the interim analysis the VE will be presented with a 2-sided 99.69% CI, and statistical significance will be achieved if the 2-sided 99.69% CI is > 30%. The success criterion for the interim analysis will be statistical significance with an observed VE point estimate of at least 50%. At the primary analysis VE will be presented with a 2-sided 95.10% CI, and statistical significance will be achieved if the 2-sided 95.10% CI is > 30%. The success criterion for the primary analysis of the study will be statistical significance with an observed VE point estimate of at least 50%. The CIs are based on a Lan-DeMets alpha-spending function for 2-group sequential tests.

In the event that the results of the interim or primary analysis are statistically significant for the primary efficacy endpoint, an additional analysis will be performed once all participants have completed their first year of follow up. For this analysis, VE will be presented with a 2-sided 95% CI, and statistical significance will be achieved if the 2-sided 95% CI is > 0%.

Model assumptions will be checked and the robustness of the primary analysis will be assessed. The Poisson regression model with robust variance has the flexibility for exploring multiple imputation approaches using, eg, the observed placebo attack rate to impute missing data. If the Poisson regression model with robust variance fails to converge, an alternative approach will be implemented. Full details will be documented in the SAP.

To support the primary analysis, a Cox proportional hazard model will be fitted to the data as well as Kaplan-Meier curves presented for the active and control groups, showing the cumulative incidence of the first case of SARS-CoV-2 RT-PCR-positive symptomatic illness occurring ≥ 15 days post second dose of study intervention. In addition, descriptive statistics for the vaccine and control groups will also be produced. Full details will be documented in the SAP.

9.4.2.2 Secondary Endpoints

The set of secondary endpoints include the following summary measures, derived from binary outcomes:

- Incidence of the first post-treatment response (negative at baseline to positive post treatment with study intervention) for SARS-CoV-2 Nucleocapsid antibodies occurring ≥ 15 days post second dose of study intervention (key secondary endpoint)
- Incidence of the first case of SARS-CoV-2 RT-PCR-positive symptomatic illness occurring ≥ 15 days post second dose of study intervention using CDC criteria (see Section 8.1.1 for definition)
- Incidence of the first case of SARS-CoV-2 RT-PCR-positive symptomatic illness occurring ≥ 15 days post second dose of study intervention using University of Oxford-defined symptom criteria. Cases are defined as RT-PCR-confirmed SARS-CoV-2 and having at least one of the following symptoms
  1. New onset of fever (> 100 °F [> 37.8 °C]), OR
  2. Cough, OR
  3. Shortness of breath, OR
  4. Anosmia/ageusia
- Incidence of the first case of SARS-CoV-2 RT-PCR-positive symptomatic illness occurring ≥ 15 days post second dose of study intervention regardless of evidence of prior SARS-CoV-2 infection (key secondary endpoint)
- Incidence of SARS-CoV-2 RT-PCR-positive severe or critical symptomatic COVID-19 occurring ≥ 15 days post second dose of study intervention (see Section 8.1.3 for definition) (key secondary endpoint)
- Incidence of SARS-CoV-2 RT-PCR-positive severe or critical symptomatic COVID-19 occurring post first dose of study intervention
- Incidence of COVID-19-related Emergency Department visits occurring ≥ 15 days post second dose of study intervention (key secondary endpoint)
- Post-treatment GMTs and GMFRs from day of dosing baseline value to 28 days post each dose in SARS-CoV-2 S, RBD antibodies (MSD serology assay)
- The proportion of participants who have a post-treatment seroresponse (≥ 4-fold rise in titers from day of dosing baseline value to 28 days post each dose) to the S, RBD antigens of AZD1222 (MSD serology assay)
- Post-treatment GMTs and GMFRs from day of dosing baseline value to 28 days post each dose in SARS-CoV-2 neutralizing antibodies (wild-type assay or pseudo-neutralization assay)
- Proportion of participants who have a post-treatment seroresponse (≥ 4-fold rise in titers from day of dosing baseline value to 28 days post each dose) to AZD1222 as measured by SARS-CoV-2 neutralizing antibodies (wild-type assay or pseudo-neutralization assay)
- The incidence of SARS-CoV-2 RT-PCR-positive symptomatic illness occurring post first dose of study intervention

Following the same methodology outlined for the primary endpoint, each of the key secondary endpoints will be analysed by a separate Poisson regression model with robust variance (Zou 2004), including study arm and age as covariates according to the hierarchical approach for multiplicity protection (refer to Section 9.4.4).

For non-key secondary endpoints, the same methodology outlined for the primary endpoint will be applied however the RRR will be estimated from each model, with a corresponding 95% CI. A p-value, corresponding to a 2-sided test, will be presented to compare the vaccine against the control. The p-value will be nominal as non-key secondary endpoints are not controlled for multiplicity. To support these analyses, descriptive statistics will be produced for the vaccine and control groups. Full details will be documented in the SAP.

To assess immune response, the set of secondary endpoints also includes:

- Post-treatment GMTs and GMFRs from day of dosing baseline values to 28 days post each dose in SARS-CoV-2 S, RBD antibodies (MSD serology assay)
- Proportion of participants who have a post-treatment seroresponse (≥ 4-fold rise in titers from day of dosing baseline values to 28 days post each dose) to the S, RBD antigens of AZD1222 (MSD serology assay)
- Post-treatment GMTs and GMFRs from day of dosing baseline values to 28 days post each dose in SARS-CoV-2 neutralizing antibodies (wild-type assay or pseudo-neutralization assay)
- Proportion of participants who have a post-treatment seroresponse (≥ 4-fold rise in titers from day of dosing baseline value to 28 days post each dose) to AZD1222 as measured by SARS-CoV-2 neutralizing antibodies (wild-type assay or pseudo-neutralization assay)

The proportion of participants who have a post-treatment seroresponse to the S and RBD antigens of AZD1222 will be derived for the vaccine and control groups, with corresponding 95% Clopper-Pearson exact CIs. Similarly, the proportion of participants who have a post-treatment seroresponse to AZD1222 as measured by SARS-CoV-2 neutralizing antibodies will be derived for the vaccine and control groups, with corresponding 95% Clopper-Pearson exact CIs.

The GMT and GMFR endpoints will be analysed on the log (base 2) scale by separate analysis of variance (ANOVA) models, and will include study arm and age as categorical covariates. On the log scale, the models will be used to estimate a mean response for the vaccine and control groups and the difference (vaccine - control), with corresponding 95% confidence limits. These values will then be back-transformed to give geometric means for the vaccine and control groups and a ratio of geometric means (vaccine/control), with corresponding 95% confidence limits. A p-value, corresponding to a 2-sided test, will be presented to

compare the vaccine against the control. The p-value will be nominal as secondary endpoints are not controlled for multiplicity.

To support these analyses, descriptive statistics will be produced for the vaccine and control groups. Full details will be documented in the SAP.

9.4.2.3 Exploratory Endpoints

Full details of the analyses for the exploratory endpoints will be specified in the SAP.

9.4.3 Safety

9.4.3.1 Primary Endpoints

Overview

The safety of AZD1222 will primarily be assessed by:

- Incidence of AEs for 28 days post each dose of study intervention
- Incidence of SAEs from Day 1 post treatment through Day 730
- Incidence of MAAE (defined in Section 8.3.8) and AESIs (defined in Section 8.3.9) from Day 1 post treatment through Day 730
- Incidence of local and systemic solicited AEs for 7 days post each dose of study intervention

AE severity will be graded according to the US FDA guidance (FDA 2007) and coded using the most recent version of the Medical Dictionary for Regulatory Activities. AEs will be presented for each treatment group by system organ class and preferred term. Summaries will include the number and percentage of participants reporting at least one event, number of events and exposure adjusted rates, where appropriate.

An overview of AEs will be presented for each treatment group, including the number and percentage of participants with any AE and SAEs. Summaries will present the relationship to study intervention as assessed by the investigator, maximum intensity, seriousness, and death.

A listing will cover details for each individual AE. Full details of all AE analyses will be provided in the SAP, including intercurrent events for safety due to potential unblinding of participants for administration of licensed and/or approved SARS-CoV-2 or COVID-19 vaccine.

9.4.3.2 Other Safety Endpoints

Vital Signs

For SARS-CoV-2-positive participants, vital sign measurements will be performed as specified in the SoA (Section 1.3). The set of assessments will include pulse oximetry, blood pressure, and body temperature.

Details of all vital sign analyses will be provided in the SAP, which will include descriptive statistics presented for observed and change from baseline values for all vital sign parameters.

9.4.4 Methods for Multiplicity Control

The primary efficacy endpoint and 4 key secondary endpoints will be assessed at 2 time points during the study, given an interim analysis and a primary analysis. A Lan-DeMets alpha-spending function has been used to account for multiplicity of the primary endpoint across the 2 time points, with 0.31% alpha at the interim analysis and 4.9% at the primary analysis. Thus, the interim and primary analyses will present estimates with 2-sided 99.69% and 95.10% CIs, respectively, and statistical significance will be achieved if the 2-sided CIs are > 30%. At the interim or primary analysis, the success criterion for the study will be statistical significance with an observed VE point estimate of at least 50%. If the primary endpoint achieves statistical significance at the 0.31% level at the interim (or at the 4.9% level at the primary), a hierarchical approach will be used to control for multiplicity of the primary and key secondary efficacy endpoints. That is, the null hypotheses for these efficacy endpoints will be tested in a hierarchical order, and the subsequent null hypothesis will be tested at a significance level of 0.31% or 4.9% (2-sided), at the interim and primary analysis, respectively, only if the prior null hypothesis is rejected.

A formal assessment of the key secondary efficacy endpoints at the interim or primary will only be conducted if the statistical significance of the primary efficacy endpoint is demonstrated at 2-sided alpha of 0.31% at the interim or 4.9% at the primary analysis. With that, the overall Type I error is controlled at 0.05. Therefore, no further multiplicity adjustment is necessary.

The testing strategy at the interim analysis or primary analysis will be as follows:

Step 1:

1 Interim analysis: Perform the test of primary endpoint with 0.31% alpha level. If the 2-sided 99.69% CI is > 30% with VE point estimate ≥ 50%, then proceed to Step 2. Otherwise no null hypothesis is rejected for the interim analysis.

2 Primary analysis: Perform the test of primary endpoint with 4.9% alpha level. If the 2-sided 95.10% CI is > 30% with VE point estimate ≥ 50%, then proceed to Step 2. Otherwise no null hypothesis is rejected for the primary analysis.

Step 2:

1 Interim analysis: Test the 4 key secondary endpoints at the significance level of 0.31% using hierarchical fixed-sequence testing in the order below. If the 2-sided 99.69% CI is > 0%, then proceed to the next endpoint.

2 Primary analysis: Test the 4 key secondary endpoints at the significance level of 4.9% using hierarchical fixed-sequence testing in the order below. If the two-sided 95.10% CI is > 0%, then proceed to the next endpoint.

(a) Key Secondary Endpoint 1: Incidence of the first case of SARS-CoV-2 RT-PCR-positive symptomatic illness occurring ≥ 15 days post second dose of study intervention regardless of evidence of prior SARS-CoV-2 infection.

(b) Key Secondary Endpoint 2: Incidence of the first case of SARS-CoV-2 RT-PCR-positive severe or critical symptomatic COVID-19 occurring ≥ 15 days post second dose of study intervention.

(c) Key Secondary Endpoint 3: Incidence of COVID-19-related Emergency Department visits occurring ≥ 15 days post second dose of study intervention.

(d) Key Secondary Endpoint 4: Incidence of the first post-treatment response (negative at baseline to positive post treatment with study intervention) for SARS-CoV-2 Nucleocapsid antibodies occurring ≥ 15 days post second dose of study intervention.

In the event that the results of the interim or primary analysis are statistically significant for the primary efficacy endpoint based on the above testing strategy, an additional analysis will be performed once all participants have completed their first year of follow up. This analysis will use the same testing strategy described above for primary and key secondary endpoints, with a 5% alpha level.

9.4.5 Sensitivity Analyses

Sensitivity analyses will be explored to assess the robustness of treatment effects for the primary efficacy endpoint, where different missing data mechanisms will be explored using multiple imputation approaches. Full details of the sensitivity analyses will be specified in the SAP, and documented prior to the primary DBL.

9.4.6 Subgroup Analyses

Subgroup analyses will be carried out to assess the consistency of the treatment effect across key, pre-defined, subgroups. These analyses will focus on the primary efficacy endpoint, and they may be performed on secondary and exploratory endpoints if deemed appropriate. The list of subgroups includes, but may not be limited to: gender, age, and serostatus at baseline. Full details of all subgroup analyses will be described in the SAP, including hypotheses that will be tested and the covariates and interaction terms to be included in the statistical models.

9.5 Interim Analyses

The study has been powered to include an interim efficacy analysis, based on the primary efficacy endpoint. The statistical analysis described in Section 9.4.2.1 will be carried out by the COVID-19 Vaccine DSMB when approximately 75 events have been reported across the

active and control groups (ie, when approximately 50% of the total amount of statistical information is available) within the population of participants who are seronegative at baseline. A statistically significant finding at the interim analysis (ie, 2-sided 99.69% CI is > 30%) will not be considered a reason to stop the study, but instead will be interpreted as early assessment of efficacy. The SAP will describe the planned interim analyses in greater detail.

9.6 Data Safety Monitoring Committee

An independent COVID-19 Vaccine DSMB will provide oversight, to ensure safe and ethical conduct of the study. During the study, the benefit/risk assessment will be continuously monitored by the COVID-19 Vaccine DSMB to ensure that the balance remains favorable. Further details, composition, and operation of the COVID-19 Vaccine DSMB will be described in a separate COVID-19 Vaccine DSMB charter.

For details on the COVID-19 Vaccine DSMB, refer to Appendix A 5.

10 SUPPORTING DOCUMENTATION AND OPERATIONAL CONSIDERATIONS

Appendix A Regulatory, Ethical, and Study Oversight Considerations

A 1 Regulatory and Ethical Considerations

- This study will be conducted in accordance with the protocol and with the following:
  - Consensus ethical principles derived from international guidelines including the Declaration of Helsinki and Council for International Organizations of Medical Sciences International Ethical Guidelines
  - Applicable ICH GCP Guidelines
  - Applicable laws and regulations
- The protocol, protocol amendments, ICF, IB, and other relevant documents (eg, advertisements) must be submitted to an IRB/IEC by the investigator and reviewed and approved by the IRB/IEC before the study is initiated.
- Any amendments to the protocol will require IRB/IEC and applicable Regulatory Authority approval before implementation of changes made to the study design, except for changes necessary to eliminate an immediate hazard to study participants.
- The Sponsor will be responsible for obtaining the required authorizations to conduct the study from the concerned Regulatory Authority. This responsibility may be delegated to a CRO but the accountability remains with the Sponsor.
- The investigator will be responsible for providing oversight of the conduct of the study at the site and adherence to requirements of 21 CFR, ICH guidelines, the IRB/IEC, European Regulation 536/2014 for clinical studies (if applicable), European Medical Device Regulation 2017/745 for clinical device research (if applicable), and all Food and Drug Administration (FDA) Regulations, as applicable and all other applicable local regulations

Regulatory Reporting Requirements for SAEs

- Prompt notification by the investigator to the Sponsor of an SAE is essential so that legal obligations and ethical responsibilities towards the safety of participants and the safety of a study intervention under clinical investigation are met.
- The Sponsor has a legal responsibility to notify both the local regulatory authority and other regulatory agencies about the safety of a study intervention under clinical investigation. The Sponsor will comply with country-specific regulatory requirements relating to safety reporting to the regulatory authority, IRBs/IECs, and investigators.
- For all studies except those utilizing medical devices, investigator safety reports must be prepared for suspected unexpected serious adverse reactions (SUSAR) according to local regulatory requirements and Sponsor policy and forwarded to investigators as necessary.
  - European Medical Device Regulation 2017/745 for clinical device research (if applicable), and all other applicable local regulations
- An investigator who receives an investigator safety report describing an SAE or other specific safety information (eg, summary or listing of SAEs) from the Sponsor will

review and then file it along with the Investigator's Brochure and will notify the IRB/IEC, if appropriate according to local requirements.

A 2 Financial Disclosure

Investigators and sub-investigators will provide the Sponsor with sufficient, accurate financial information as requested to allow the Sponsor to submit complete and accurate financial certification or disclosure statements to the appropriate regulatory authorities. Investigators are responsible for providing information on financial interests during the course of the study and for 1 year after completion of the study.

A 3 Informed Consent Process

The investigator or his/her representative will explain the nature of the study to the participant or his/her legally authorized representative and answer all questions regarding the study.

- Participants must be informed that their participation is voluntary and they are free to refuse to participate and may withdraw their consent at any time and for any reason during the study. Participants or their legally authorized representative will be required to sign a statement of informed consent that meets the requirements of 21 CFR 50, local regulations, ICH guidelines, Health Insurance Portability and Accountability Act requirements, where applicable, and the IRB/IEC or study center.
- The study medical record must include a statement that written informed consent was obtained before the participant was enrolled in the study and the date the written consent was obtained. The authorized person obtaining the informed consent must also sign the ICF.
- Participants must be re-consented to the most current version of the ICF(s) during their participation in the study if required by the IRB.
- A copy of the ICF(s) must be provided to the participant or the participant's legally authorized representative.

Participants who are rescreened are required to sign a new ICF.

The ICF will contain a separate section that addresses and documents the collection and use of any mandatory and/or optional human biological samples. The investigator or authorized designee will explain to each participant the objectives of the analysis to be done on the samples and any potential future use. Participants will be told that they are free to refuse to participate in any optional samples or the future use and may withdraw their consent at any time and for any reason during the retention period.

A 4 Data Protection

- Participants will be assigned a unique identifier by the Sponsor. Any participant records or datasets that are transferred to the Sponsor will contain the identifier only; participant

names or any information which would make the participant identifiable will not be transferred.

- The participant must be informed that his/her personal study-related data will be used by the Sponsor in accordance with local data protection law. The level of disclosure and use of their data must also be explained to the participant in the informed consent.
- The participant must be informed that his/her medical records may be examined by Clinical Quality Assurance auditors or other authorized personnel appointed by the Sponsor, by appropriate IRB/IEC members, and by inspectors from regulatory authorities.

A 5 Committee Structure

The safety of all Sponsor clinical studies is closely monitored on an ongoing basis by Sponsor representatives in consultation with Patient Safety. Issues identified will be addressed; for instance, this could involve amendments to the Clinical Study Protocol and letters to investigators.

A PSRT comprised of Sponsor, COVID-19 Prevention Network, Biomedical Advanced Research and Development Authority, and NIAID medical officers will be convened to oversee blinded safety surveillance of participants during the study. Further details, composition, and operation of the PSRT will be described in a separate COVID-19 Prevention Network PSRT Charter.

A COVID-19 Vaccine DSMB organized by the National Institutes of Health, National Institute for Allergy and Infectious Diseases, comprised of independent experts will be convened to provide oversight, to ensure safe and ethical conduct of the study. The COVID-19 Vaccine DSMB will facilitate the interim analysis for safety and efficacy and have the responsibility of evaluating cumulative safety and other clinical study data at regular intervals and making appropriate recommendations based on the available data. During the study, the benefit/risk assessment will be continuously monitored by the COVID-19 Vaccine DSMB to ensure that the balance remains favorable. For example, events of potential vaccine-associated enhanced respiratory disease will be evaluated by periodic reviews of COVID-19 cases by the DSMB. Harm for severe COVID-19 cases is any $VE \leq 0$ for which Fisher's exact test (1-sided) is statistically significant at the 5% level. This assessment will begin after 8 cases of severe COVID-19 have accrued in the study and will be performed in real time as events occur. Harm monitoring will include all COVID-19 cases and all severe COVID-19 cases from Day 1 for participants in the full analysis set. Harm monitoring for overall COVID-19 cases will use the same boundary as severe COVID-19 cases (ie, $VE \leq 0$ for which Fisher's exact test [1-sided] is statistically significant at the 5% level) but will be performed on a weekly basis. Full details of the COVID-19 Vaccine DSMB composition and operations can be found in the COVID-19 Vaccine DSMB Charter.

An independent Neurological AESI Expert Committee will be available to review and provide advice to the PSRT and the COVID-19 Vaccine DSMB on request about the diagnosis and causality assessment of selected neurological AESIs occurring in the AZD1222 clinical development program. Details on the composition and operation of this committee are described in the Neurological AESI Expert Committee Charter.

A blinded independent efficacy adjudication committee will review relevant data of potential cases for the COVID-19-related efficacy endpoint evaluations. More detail on this process will be provided in the SAP and the Efficacy Adjudication Committee Charter.

A 6 Dissemination of Clinical Study Data

A description of this clinical study will be available on http://astrazenecagrouptrials.pharmacm.com and http://www.clinicaltrials.gov as will the summary of the study results when they are available. The clinical study and/or summary of study results may also be available on other websites according to the regulations of the countries in which the study is conducted.

A 7 Data Quality Assurance

- All participant data relating to the study will be recorded on eCRF unless transmitted to the Sponsor or designee electronically (eg, laboratory data). The investigator is responsible for verifying that data entries are accurate and correct by electronically signing the eCRF.
- The investigator must maintain accurate documentation (source data) that supports the information entered in the eCRF.
- The investigator must permit study-related monitoring, audits, IRB/IEC review, and regulatory agency inspections and provide direct access to source data documents.
- Monitoring details describing strategy (eg, risk-based initiatives in operations and quality such as Risk Management and Mitigation Strategies and Analytical Risk-Based Monitoring), methods, responsibilities and requirements, including handling of noncompliance issues and monitoring techniques (central, remote, or on-site monitoring) are provided in the relevant study plans.
- The Sponsor or designee is responsible for the data management of this study including quality checking of the data.
- The Sponsor assumes accountability for actions delegated to other individuals (eg, Contract Research Organizations).
- Study monitors will perform ongoing source data verification to confirm that data entered into the eCRF by authorized site personnel are accurate, complete, and verifiable from source documents; that the safety and rights of participants are being protected; and that the study is being conducted in accordance with the currently approved protocol and any other study agreements, ICH GCP, and all applicable regulatory requirements.

- Records and documents, including signed ICFs, pertaining to the conduct of this study must be retained by the investigator for 15 years after study completion unless local regulations or institutional policies require a longer retention period. No records may be destroyed during the retention period without the written approval of the Sponsor. No records may be transferred to another location or party without written notification to the sponsor.

A 8 Source Documents

- Source documents provide evidence for the existence of the participant and substantiate the integrity of the data collected. Source documents are filed at the investigator's site.
- Data entered in the eCRF that are transcribed from source documents must be consistent with the source documents or the discrepancies must be explained. The investigator may need to request previous medical records or transfer records, depending on the study. Also, current medical records must be available.

A 9 Study and Site Start and Closure

The first act of recruitment is the first participant screened and will be the study start date.

The Sponsor designee reserves the right to close the study site or terminate the study at any time for any reason at the sole discretion of the Sponsor. Study sites will be closed upon study completion. A study site is considered closed when all required documents and study supplies have been collected and a study-site closure visit has been performed.

The investigator may initiate study-site closure at any time, provided there is reasonable cause and sufficient notice is given in advance of the intended termination.

Reasons for the early closure of a study site by the Sponsor or investigator may include but are not limited to:

- Failure of the investigator to comply with the protocol, the requirements of the IRB/IEC or local health authorities, the Sponsor's procedures, or GCP guidelines
- Inadequate recruitment of participants by the investigator
- Discontinuation of further study intervention development

If the study is prematurely terminated or suspended, the Sponsor shall promptly inform the investigators, the IECs/IRBs, the regulatory authorities, and any contract research organization(s) used in the study of the reason for termination or suspension, as specified by the applicable regulatory requirements. The investigator shall promptly inform the participant and should assure appropriate participant therapy and/or follow-up.

Participants from terminated sites may have the opportunity to be transferred to another site to continue the study.

A 10 Publication Policy

- The results of this study may be published or presented at scientific meetings. If this is foreseen, the investigator agrees to submit all manuscripts or abstracts to the Sponsor before submission. This allows the Sponsor to protect proprietary information and to provide comments.
- The Sponsor will comply with the requirements for publication of study results. In accordance with standard editorial and ethical practice, the Sponsor will generally support publication of multicenter studies only in their entirety and not as individual site data. In this case, a coordinating investigator will be designated by mutual agreement.
- Authorship will be determined by mutual agreement and in line with International Committee of Medical Journal Editors authorship requirements.

Appendix B Adverse Events: Definitions and Procedures for Recording, Evaluating, Follow-up, and Reporting

B 1 Definition of Adverse Events

An AE is the development of any untoward medical occurrence in a patient or clinical study participant administered a medicinal product and which does not necessarily have a causal relationship with this treatment. An AE can therefore be any unfavorable and unintended sign (eg, an abnormal laboratory finding), symptom (for example nausea, chest pain), or disease temporally associated with the use of a medicinal product, whether or not considered related to the medicinal product.

The term AE is used to include both SAEs and non-SAEs and can include a deterioration of a pre-existing medical occurrence. An AE may occur at any time, including run-in or washout periods, even if no study intervention has been administered.

B 2 Definition of Serious Adverse Events

An SAE is an AE occurring during any study phase (ie, run-in, treatment, washout, follow-up), that fulfils one or more of the following criteria:

- Results in death
- Is immediately life-threatening
- Requires in-participant hospitalization or prolongation of existing hospitalization
- Results in persistent or significant disability or incapacity
- Is a congenital abnormality or birth defect
- Is an important medical event that may jeopardize the participant or may require medical treatment to prevent one of the outcomes listed above.

AEs for **malignant tumors** reported during a study should generally be assessed as **SAEs**. If no other seriousness criteria apply, the 'Important Medical Event' criterion should be used. In certain situations, however, medical judgement on an individual event basis should be applied to clarify that the malignant tumor event should be assessed and reported as a **non-SAE**. For example, if the tumor is included as medical history and progression occurs during the study, but the progression does not change treatment and/or prognosis of the malignant tumor, the AE may not fulfil the attributes for being assessed as serious, although reporting of the progression of the malignant tumor as an AE is valid and should occur. Also, some types of malignant tumors, which do not spread remotely after a routine treatment that does not require hospitalization, may be assessed as non-serious; examples in adults include Stage 1 basal cell carcinoma and Stage 1A1 cervical cancer removed via cone biopsy.

Life Threatening

'Life-threatening' means that the participant was at immediate risk of death from the AE as it occurred or it is suspected that use or continued use of the study intervention would result in the participant's death. 'Life-threatening' does not mean that had an AE occurred in a more severe form it might have caused death (eg, hepatitis that resolved without hepatic failure).

Hospitalization

Outpatient treatment in an emergency room is not in itself an SAE, although the reasons for it may be (eg, bronchospasm, laryngeal oedema). Hospital admissions and/or surgical operations planned before or during a study are not considered AEs if the illness or disease existed before the participant was enrolled in the study, provided that it did not deteriorate in an unexpected way during the study.

Important Medical Event or Medical Treatment

Medical and scientific judgement should be exercised in deciding whether a case is serious in situations where important medical events may not be immediately life threatening or result in death, hospitalization, disability, or incapacity but may jeopardize the participant or may require medical treatment to prevent one or more outcomes listed in the definition of serious. These should usually be considered as serious.

Simply stopping the suspect drug does not mean that it is an important medical event; medical judgement must be used.

Examples of important medical events include such events as listed below:

- Angioedema not severe enough to require intubation but requiring iv hydrocortisone treatment
- Hepatotoxicity caused by acetaminophen overdose requiring treatment with N-acetylcysteine
- Intensive treatment in an emergency room or at home for allergic bronchospasm
- Blood dyscrasias (eg, neutropenia or anaemia requiring blood transfusion, etc.) or convulsions that do not result in hospitalisation
- Development of drug dependency or drug abuse

Intensity Rating Scale

The grading scales found in the US FDA guidance for healthy volunteers enrolled in a preventive vaccine clinical study (FDA 2007) will be utilized for all events with an assigned severity grading.

It is important to distinguish between serious and severe AEs. Severity is a measure of intensity whereas seriousness is defined by the criteria in Appendix B 2. An AE of severe

intensity need not necessarily be considered serious. For example, nausea that persists for several hours may be considered severe nausea, but not an SAE unless it meets the criteria shown in Appendix B 2. On the other hand, a stroke that results in only a limited degree of disability may be considered a mild stroke but would be an SAE when it satisfies the criteria shown in Appendix B 2.

B 3 A Guide to Interpreting the Causality Question

When making an assessment of causality consider the following factors when deciding if there is a 'reasonable possibility' that an AE may have been caused by the IMP.

- Time Course. Exposure to suspect drug. Has the participant actually received the suspect drug? Did the AE occur in a reasonable temporal relationship to the administration of the suspect IMP?
- Consistency with known IMP profile. Was the AE consistent with the previous knowledge of the suspect IMP (pharmacology and toxicology) or drugs of the same pharmacological class? Or could the AE be anticipated from its pharmacological properties?
- De-challenge experience. Did the AE resolve or improve on stopping or reducing the dose of the suspect IMP?
- No alternative cause. The AE cannot be reasonably explained by another aetiology such as the underlying disease, other drugs, other host or environmental factors.
- Re-challenge experience. Did the AE reoccur if the suspected IMP was reintroduced after having been stopped? AstraZeneca would not normally recommend or support a re-challenge.
- Laboratory tests. A specific laboratory investigation (if performed) has confirmed the relationship.

In difficult cases, other factors could be considered such as:

- Is this a recognized feature of overdose of the IMP?
- Is there a known mechanism?

Causality of 'related' is made if following a review of the relevant data, there is evidence for a 'reasonable possibility' of a causal relationship for the individual case. The expression 'reasonable possibility' of a causal relationship is meant to convey, in general, that there are facts (evidence) or arguments to suggest a causal relationship.

The causality assessment is performed based on the available data including enough information to make an informed judgment. With no available facts or arguments to suggest a causal relationship, the event(s) will be assessed as 'not related'.

Causal relationship in cases where the disease under study has deteriorated due to lack of effect should be classified as no reasonable possibility.

B 4 Medication Error

For the purposes of this clinical study a medication error is an unintended failure or mistake in the treatment process for an AstraZeneca study intervention that either causes harm to the participant or has the potential to cause harm to the participant.

A medication error is not lack of efficacy of the IMP, but rather a human or process related failure while the IMP is in control of the study site staff or participant.

Medication error includes situations where an error.

- Occurred
- Was identified and intercepted before the participant received the IMP
- Did not occur, but circumstances were recognised that could have led to an error

Examples of events to be reported in clinical studies as medication errors:

- IMP name confusion
- Dispensing error eg, medication prepared incorrectly, even if it was not actually given to the participant
- IMP not administered as indicated, for example, wrong route or wrong site of administration
- IMP not taken as indicated eg, tablet dissolved in water when it should be taken as a solid tablet
- IMP not stored as instructed eg, kept in the fridge when it should be at room temperature
- Wrong participant received the medication (excluding IRT errors)
- Wrong IMP administered to participant (excluding IRT errors)

Examples of events that **do not** require reporting as medication errors in clinical studies:

- Errors related to or resulting from IRT - including those which lead to one of the above listed events that would otherwise have been a medication error
- Participant accidentally missed IMP dose(s) eg, forgot to take medication
- Accidental overdose (will be captured as an overdose)
- Errors related to background and rescue medication, or standard of care medication in open label studies, even if an AstraZeneca product

Medication errors are not regarded as AEs but AEs may occur as a consequence of the medication error.

Appendix C Handling of Human Biological Samples

C 1 Chain of Custody

A full chain of custody is maintained for all samples throughout their lifecycle.

The investigator at each study site keeps full traceability of collected biological samples from the participants while in storage at the study site until shipment or disposal (where appropriate) and records relevant processing information related to the samples whilst at site.

The sample receiver keeps full traceability of the samples while in storage and during use until used or disposed of or until further shipment and keeps record of receipt of arrival and onward shipment or disposal.

The Sponsor or delegated representatives will keep oversight of the entire life cycle through internal procedures, monitoring of study sites, auditing or process checks, and contractual requirements of external laboratory providers

Samples retained for further use will be stored in the AstraZeneca-assigned biobanks or other sample archive facilities and will be tracked by the appropriate AstraZeneca Team during for the remainder of the sample life cycle.

C 2 Withdrawal of Informed Consent for Donated Biological Samples

The Sponsor ensures that biological samples are destroyed at the end of a specified period as described in the informed consent.

If a participant withdraws consent to the use of donated biological samples, the samples will be disposed of/destroyed/repatriated, and the action documented. If samples are already analyzed, the Sponsor is not obliged to destroy the results of this research.

Following withdrawal of consent for biological samples, further study participation should be considered in relation to the withdrawal processes.

The investigator:

- Ensures participant's withdrawal of informed consent to the use of donated samples is highlighted immediately to the Sponsor or delegate.
- Ensures that relevant human biological samples from that participant, if stored at the study site, are immediately identified, disposed of as appropriate, and the action documented.
- Ensures that the participant and the Sponsor are informed about the sample disposal.

The Sponsor ensures the organization(s) holding the samples is/are informed about the withdrawn consent immediately and that samples are disposed of or repatriated as appropriate, and the action is documented and study site is notified.

C 3 International Airline Transportation Association 6.2 Guidance Document

LABELLING AND SHIPMENT OF BIOHAZARD SAMPLES

International Airline Transportation Association (IATA) (**https://www.iata.org/whatwedo/cargo/dgr/Pages/download.aspx**) classifies infectious substances into 3 categories: Category A, Category B or Exempt

Category A Infectious Substances are infectious substances in a form that, when exposure to it occurs, is capable of causing permanent disability, life-threatening or fatal disease in otherwise healthy humans or animals.
Category A Pathogens are, eg, Ebola, Lassa fever virus. Infectious substances meeting these criteria which cause disease in humans or both in humans and animals must be assigned to UN 2814. Infectious substances which cause disease only in animals must be assigned to UN 2900.

Category B Infectious Substances are infectious Substances that do not meet the criteria for inclusion in Category A. Category B pathogens are, eg, Hepatitis A, C, D, and E viruses. They are assigned the following UN number and proper shipping name:

- UN 3373 – Biological Substance, Category B
- are to be packed in accordance with UN 3373 and IATA 650

Exempt - Substances which do not contain infectious substances or substances which are unlikely to cause disease in humans or animals are not subject to these Regulations unless they meet the criteria for inclusion in another class.

- Clinical study samples will fall into Category B or exempt under IATA regulations
- Clinical study samples will routinely be packed and transported at ambient temperature in IATA 650 compliant packaging (https://www.iata.org/whatwedo/cargo/dgr/Documents/DGR-60-EN-PI650.pdf).
- Biological samples transported in dry ice require additional dangerous goods specification for the dry-ice content

Appendix D Toxicity Grading Scales for Solicited Adverse Events

The toxicity grading scales for the solicited AEs were modified and abridged from the US FDA Guidance on Toxicity Grading Scale for Healthy Adult and Adolescent Volunteers Enrolled in Preventive Vaccine Clinical Trials (FDA 2007).

- Table 10: Clinical Abnormalities, Local Reactions to Injectable Product
- Table 11: Clinical Abnormalities, Vital Signs
- Table 12: Clinical Abnormalities, Systemic (General or Illness)

Table 10 Tables for Clinical Abnormalities: Local Reactions to Injectable Product

Local Reaction to Injectable Product	Reaction Grade			
	**Mild (Grade 1)**	**Moderate (Grade 2)**	**Severe (Grade 3)**	**Life Threatening (Grade 4)**
Pain	Does not interfere with activity	Repeated use of non-narcotic pain reliever > 24 hours or interferes with activity	Any use of narcotic pain reliever or prevents daily activity	ER visit or hospitalization
Tenderness	Mild discomfort to touch	Discomfort with movement	Significant discomfort at rest	ER visit or hospitalization
Erythema/redness [a, b]	1-2 inches (2.5–5 cm)	> 2-4 inches (5.1–10 cm)	> 4 inches (> 10 cm)	Necrosis or exfoliative dermatitis
Induration/swelling [a, b]	1-2 inches (2.5–5 cm)	>2-4 inches (5.1–10 cm)	> 4 inches (> 10 cm)	Necrosis

[a] In addition to grading the measured local reaction at the greatest single diameter, the measurement should be recorded as a continuous variable. Reactions < 0.25 inches (< 0.6 centimeters) in diameter will not be recorded.

[b] Grade 4 erythema or induration is determined by study site with participant input rather than being recorded directly in Solicited AE e-Diary.

ER = emergency room.

Table 11 Tables for Clinical Abnormalities: Vital Signs

Vital Sign [a]	Vital Signs Grade			
	**Mild (Grade 1)**	**Moderate (Grade 2)**	**Severe (Grade 3)**	**Potentially Life Threatening (Grade 4)**
Fever (°C) [b]<br>(°F) [b]	37.9-38.4<br>100.1-101.1	38.5-38.9<br>101.2-102.0	39.0-40<br>102.1-104	> 40<br>> 104
Tachycardia (beats/minute)	101-115	116- 130	> 130	ER visit or hospitalization for arrhythmia
Bradycardia (beats/minute) [c]	50-54	45-49	< 45	ER visit or hospitalization for arrhythmia
Hypertension; systolic (mm Hg)	141-150	151-155	> 155	ER visit or hospitalization for malignant hypertension
Hypertension; diastolic (mm Hg)	91-95	96-100	> 100	ER visit or hospitalization for malignant hypertension
Hypotension; systolic (mm Hg)	85-89	80-84	< 80	ER visit or hospitalization for hypotensive shock
Respiratory rate (breaths/minute)	17-20	21-25	> 25	Intubation

Note: Record vital signs as adverse events only if clinically relevant and changed from baseline.

[a] Participant should be at rest for vital signs measurements

[b] No recent hot or cold beverages or smoking

[c] Use clinical judgment when characterizing bradycardia among some healthy participant populations, for example, conditioned athletes

ER = emergency room; Hg = mercury.

Table 12 Tables for Clinical Abnormalities: Systemic (General or Illness)

Systemic (General)	Systemic Grade			
	**Mild (Grade 1)**	**Moderate (Grade 2)**	**Severe (Grade 3)**	**Potentially Life Threatening (Grade 4)**
Nausea/vomiting	No interference with activity or 1-2 episodes/24 hrs	Some interference with activity or > 2 episodes/24 hrs	Prevents daily activity, required outpatient IV hydration	ER visit or hospitalization for hypotensive shock
Chills	No interference with activity	Some interference with activity	Significant; prevents daily activity	ER visit or hospitalization
Headache	No interference with activity	Repeated use of non-narcotic pain reliever > 24 hrs or some interference with activity	Significant; any use of narcotic pain reliever or prevents daily activity	ER visit or hospitalization
Fatigue	No interference with activity	Some interference with activity	Significant; prevents daily activity	ER visit or hospitalization
Myalgia	No interference with activity	Some interference with activity	Significant; prevents daily activity	ER visit or hospitalization
**Systemic Illness**				
Illness or clinical adverse event (as defined according to applicable regulations)	No interference with activity	Some interference with activity not requiring intervention	Prevents daily activity and required medical intervention	ER visit or hospitalization

ER = emergency room; hrs = hours; IV = intravenous.

Appendix E Adverse Events of Special Interest

AZD1222 AESIs are based on Brighton Collaboration case definitions (SPEAC 2020), clinical experience, and scientific interest. There is no current evidence to suggest that AZD1222 is associated with these AESIs.

Table 13 Adverse Events of Special Interest

AESI	Medical Concept
Neurologic	Generalized convulsion: Seizures are episodes of neuronal hyperactivity most commonly resulting in sudden, involuntary muscular contractions. They may also manifest as sensory disturbances, autonomic dysfunction and behavioral abnormalities, and impairment or loss of consciousness.
	Guillain-Barré syndrome: GBS is a peripheral nerve demyelinating disease, which can present as temporary ascending paralysis.
	Acute disseminated encephalomyelitis: ADEM is defined as a uniphasic syndrome of brain inflammation and demyelination occurring in temporal association with an antecedent immunologic challenge, such as infection or an immunization. ADEM most commonly occurs in the pediatric population.
	Other neurologic events: These events would include new onset event (acute or subacute) motor and sensory disturbances (eg, weakness, numbness, paresthesias, hypoesthesia, hyperesthesia, dysesthesias), bowel/bladder dysfunction, gait impairment, or visual disturbance, or any event of myelitis, encephalomyelitis, myelitis transverse, or other sudden neurological deficit.
Vascular	Thrombotic, thromboembolic, and neurovascular events: These are events that can manifest as transient or permanent vision problems, dizziness, trouble understanding, facial droop, slurred speech, unilateral weakness, deep vein thrombosis with swollen, warm or painful leg, pulmonary embolism with shortness of breath, chest pain or irregular heart rate.
Hematologic	Thrombocytopenia: Thrombocytopenia is a disorder in which there is an abnormally low platelet count; a normal platelet count ranges from 150 000 to 450 000 platelets per μL.
Immunologic	Vasculitides: Vasculitides are a group of related disorders characterized by inflammation of blood vessels (vasculitis) leading to tissue or end-organ injury.
	Anaphylaxis: Anaphylaxis an acute hypersensitivity reaction with multi-organ-system involvement that can present as, or rapidly progress to, a severe life-threatening reaction requiring immediate medical attention.
	Vaccine-associated enhanced respiratory disease: The pathogenicity of VAERD has been linked to a vaccine immune response characterized by induction of non-neutralizing antibodies, and a T-cell response of the Th2 type with hypereosinophilia (Lambert et al 2020). VAERD may manifest as a severe form of respiratory disease with prolonged fever, and diverse clinical manifestations of disease severity and pathological changes marked by increased areas of lung consolidation, broncho-interstitial pneumonia, and necrotizing bronchiolitis (Rajão et al 2016).

Table 13 Adverse Events of Special Interest

AESI	Medical Concept
	Potential immune-mediated conditions: These conditions are a group of autoimmune inflammatory disorders characterized by an alteration in cellular homeostasis, which may or may not have an autoimmune aetiology. A list of events is provided in Table 14.

ADEM = acute disseminated encephalomyelitis; AESI = adverse event of special interest; GBS = Guillain-Barré syndrome; VAERD = vaccine-associated enhanced respiratory disease.

Table 14 List of Potential Immune-mediated Medical Conditions

Category	Condition
Gastrointestinal disorders	Celiac disease
	Crohn’s disease
	Ulcerative colitis
	Ulcerative proctitis
Liver disorders	Autoimmune cholangitis
	Autoimmune hepatitis
	Primary biliary cirrhosis
	Primary sclerosing cholangitis
Metabolic diseases	Addison’s disease
	Autoimmune thyroiditis (including Hashimoto thyroiditis)
	Diabetes mellitus type I
	Grave's or Basedow’s disease
Musculoskeletal disorders	Antisynthetase syndrome
	Dermatomyositis
	Juvenile chronic arthritis (including Still’s disease)
	Mixed connective tissue disorder
	Polymyalgia rheumatic
	Polymyositis
	Psoriatic arthropathy
	Relapsing polychondritis
	Rheumatoid arthritis
	Scleroderma, including diffuse systemic form and CREST syndrome
	Spondyloarthritis, including ankylosing spondylitis, reactive arthritis (Reiter's Syndrome) and undifferentiated spondyloarthritis
	Systemic lupus erythematosus
	Systemic sclerosis

Table 14 List of Potential Immune-mediated Medical Conditions

Category	Condition
Neuroinflammatory disorders	Acute disseminated encephalomyelitis, including site specific variants (eg, non-infectious encephalitis, encephalomyelitis, myelitis, radiculomyelitis)
	Cranial nerve disorders, including paralyses/paresis (eg, Bell's palsy)
	Guillain-Barré syndrome, including Miller Fisher syndrome and other variants
	Immune-mediated peripheral neuropathies and plexopathies, including chronic inflammatory demyelinating polyneuropathy, multifocal motor neuropathy and polyneuropathies associated with monoclonal gammopathy
	Multiple sclerosis
	Neuromyelitis optica spectrum disorder
	Narcolepsy
	Optic neuritis
	Transverse myelitis
	Myasthenia gravis, including Eaton-Lambert syndrome
Skin disorders	Alopecia areata
	Autoimmune bullous skin diseases, including pemphigus, pemphigoid and dermatitis herpetiformis
	Cutaneous lupus erythematosus
	Erythema nodosum
	Morphoea
	Lichen planus
	Psoriasis
	Rosacea
	Sweet's syndrome
	Vitiligo
Vasculitides	Large vessels vasculitis including: giant cell arteritis such as Takayasu's arteritis and temporal arteritis
	Medium sized and/or small vessels vasculitis including: polyarteritis nodosa, Kawasaki's disease, microscopic polyangiitis, Wegener's granulomatosis, Churg– Strauss syndrome (allergic granulomatous angiitis), Buerger's disease, thromboangiitis obliterans, necrotizing vasculitis and anti-neutrophil cytoplasmic antibody (ANCA) positive vasculitis (type unspecified), Henoch-Schonlein purpura, Behcet's syndrome, leukocytoclastic vasculitis

Table 14 List of Potential Immune-mediated Medical Conditions

Category	Condition
Other	Antiphospholipid syndrome
	Autoimmune hemolytic anemia
	Autoimmune glomerulonephritis (including IgA nephropathy, glomerulonephritis rapidly progressive, membranous glomerulonephritis, membranoproliferative glomerulonephritis, and mesangioproliferative glomerulonephritis)
	Autoimmune myocarditis/cardiomyopathy
	Autoimmune thrombocytopenia
	Goodpasture syndrome
	Idiopathic pulmonary fibrosis
	Pernicious anemia
	Raynaud's phenomenon
	Sarcoidosis
	Sjögren's syndrome
	Stevens-Johnson syndrome
	Uveitis

Appendix F Overview of Primary and Secondary Efficacy Objectives, Endpoints, and Associated Case Definitions

Table 15 Overview of Primary and Secondary Efficacy Objectives, Endpoints, and Associated Case Definitions

Objective		Endpoint	Case Definition
Primary	To estimate the efficacy of 2 IM doses of AZD1222 compared to saline placebo for the prevention of COVID-19	A binary response, whereby a participant with negative serostatus at baseline is defined as a COVID-19 case if their first case of SARS-CoV-2 RT-PCR-positive symptomatic illness occurs ≥ 15 days post second dose of study intervention. Otherwise, a participant is not defined as a COVID-19 case.	Participant must have RT-PCR-confirmed SARS-CoV-2 and meet the following criteria at any point from their initial illness visit at the site (Day 1) through their second illness visit (Day 14):<br>1 One or more Category A findings<br>**OR**<br>2 Two or more Category B findings<br><br>*Category A:*<br>• Pneumonia diagnosed by chest x-ray, or computed tomography scan<br>• Oxygen saturation of ≤ 94% on room air or requiring either new initiation or escalation in supplemental O_2<br>• New or worsening dyspnea/shortness of breath<br><br>*Category B:*<br>• Fever > 100 °F (> 37.8 °C) or feverishness<br>• New or worsening cough<br>• Myalgia/muscle pain<br>• Fatigue that interferes with activities of daily living<br>• Vomiting and/or diarrhea (only one finding to be counted toward endpoint definition)<br>• Anosmia and/or ageusia (only one finding to be counted toward endpoint definition)

Table 15 Overview of Primary and Secondary Efficacy Objectives, Endpoints, and Associated Case Definitions

Objective		Endpoint	Case Definition
Secondary	To estimate the efficacy of 2 IM doses of AZD1222 compared to saline placebo for the prevention of SARS-CoV-2 infection	The incidence of the first post-treatment response (negative at baseline to positive post treatment with study intervention) for SARS-CoV-2 Nucleocapsid antibodies occurring ≥ 15 days post second dose of study intervention (key secondary endpoint)	The first post-treatment response (negative at baseline to positive post treatment with study intervention) for SARS-CoV-2 Nucleocapsid antibodies occurring ≥ 15 days post second dose of study intervention
	To estimate the efficacy of 2 IM doses of AZD1222 compared to saline placebo for the prevention of symptomatic COVID-19 using CDC criteria	The incidence of the first case of SARS-CoV-2 RT-PCR-positive symptomatic illness occurring ≥ 15 days post second dose of study intervention using CDC criteria	The first case of SARS-CoV-2 RT-PCR-positive symptomatic illness for a participant occurring ≥ 15 days post second dose of study intervention using criteria from the CDC (CDC 2020):<br>Fever<br>Shortness of breath<br>Difficulty breathing<br>Chills<br>Cough<br>Fatigue<br>Muscle aches<br>Body aches<br>Headache<br>New loss of taste<br>New loss of smell<br>Sore throat<br>Congestion<br>Runny nose<br>Nausea<br>Vomiting<br>Diarrhea

Table 15 Overview of Primary and Secondary Efficacy Objectives, Endpoints, and Associated Case Definitions

Objective		Endpoint	Case Definition
Secondary	To estimate the efficacy of 2 IM doses of AZD1222 compared to saline placebo for the prevention of University of Oxford-defined symptomatic COVID-19	The incidence of the first case of SARS-CoV-2 RT-PCR-positive symptomatic illness occurring ≥ 15 days post second dose of study intervention using University of Oxford-defined symptom criteria	First case of SARS-CoV-2 RT-PCR-positive symptomatic illness for a participant occurring ≥ 15 days post second dose of study intervention using University of Oxford-defined symptom criteria. Cases are defined as RT-PCR-confirmed SARS-CoV-2 and having at least one of the following symptoms:<br>1 New onset of fever (> 100 °F [> 37.8 °C]), OR<br>2 Cough, OR<br>3 Shortness of breath, OR<br>4 Anosmia/ageusia

Table 15 Overview of Primary and Secondary Efficacy Objectives, Endpoints, and Associated Case Definitions

Objective		Endpoint	Case Definition
Secondary	To estimate the efficacy of 2 IM doses of AZD1222 compared to saline placebo in the prevention of COVID-19 in all study participants, regardless of evidence of prior SARS-CoV-2 infection	The incidence of the first case of SARS-CoV-2 RT-PCR-positive symptomatic illness occurring ≥ 15 days post second dose of study intervention regardless of evidence of prior SARS-CoV-2 infection (key secondary endpoint	Participant must have RT-PCR-confirmed SARS-CoV-2 and meet the following criteria at any point from their initial illness visit at the site (Day 1) through their second illness visit (Day 14):<br>1 One or more Category A findings<br>**OR**<br>2 Two or more Category B findings<br><br>*Category A:*<br>• Pneumonia diagnosed by chest x-ray, or computed tomography scan<br>• Oxygen saturation of ≤ 94% on room air or requiring either new initiation or escalation in supplemental O_2<br>• New or worsening dyspnea/shortness of breath<br><br>*Category B:*<br>• Fever > 100 °F (> 37.8 °C) or feverishness<br>• New or worsening cough<br>• Myalgia/muscle pain<br>• Fatigue that interferes with activities of daily living<br>• Vomiting and/or diarrhea (only one finding to be counted toward endpoint definition)<br>• Anosmia and/or ageusia (only one finding to be counted toward endpoint definition)

Table 15 Overview of Primary and Secondary Efficacy Objectives, Endpoints, and Associated Case Definitions

Objective		Endpoint	Case Definition
Secondary	To estimate the efficacy of 2 IM doses of AZD1222 compared to saline placebo for the prevention of severe or critical symptomatic COVID-19	a) The incidence of SARS-CoV-2 RT-PCR-positive severe or critical symptomatic illness occurring ≥ 15 days post second dose of study intervention (key secondary endpoint)<br>b) The incidence of SARS-CoV-2 RT-PCR positive severe or critical symptomatic illness occurring post first dose of study intervention	Participant must have laboratory-confirmed COVID-19 (SARS-CoV-2 RT-PCR-positive symptomatic illness) plus any of the following:<br>• Clinical signs at rest indicative of severe systemic illness (respiratory rate ≥ 30 breaths per minute, heart rate ≥ 125 beats per minute, oxygen saturation ≤ 93% on room air at sea level, or partial pressure of oxygen to fraction of inspired oxygen ratio < 300 mm Hg)<br>• Respiratory failure (defined as needing high-flow oxygen, noninvasive ventilation, mechanical ventilation or extracorporeal membrane oxygenation)<br>• Evidence of shock (systolic blood pressure < 90 mm Hg, diastolic blood pressure < 60 mm Hg, or requiring vasopressors)<br>• Significant acute renal, hepatic, or neurologic dysfunction<br>• Admission to an intensive care unit<br>• Death
	To estimate the efficacy of 2 IM doses of AZD1222 compared to saline placebo for the prevention of COVID-19-related Emergency Department visits	The incidence of COVID-19-related Emergency Department visits occurring ≥ 15 days post second dose of study intervention (key secondary endpoint)	COVID-19-related Emergency Department visits occurring ≥ 15 days post second dose of study intervention

Table 15 Overview of Primary and Secondary Efficacy Objectives, Endpoints, and Associated Case Definitions

Objective		Endpoint	Case Definition
Secondary	To assess antibody responses to AZD1222 S antigen following 2 IM doses of AZD1222 or saline placebo<br>(Substudy and Illness Visits only)	a) Post-treatment GMTs and GMFRs from day of dosing baseline value to 28 days post each dose in SARS-CoV-2 S, RBD antibodies (MSD serology assay)	a) Post-treatment GMTs and GMFRs from day of dosing baseline value to 28 days post each dose in SARS-CoV-2 S, RBD antibodies (MSD serology assay)
		b) The proportion of participants who have a post-treatment seroresponse (≥ 4-fold rise in titers from day of dosing baseline value to 28 days post each dose) to the S, RBD antigens of AZD1222 (MSD serology assay)	b) Post-treatment seroresponse (≥ 4-fold rise in titers from day of dosing baseline value to 28 days post each dose) to the S, RBD antigens of AZD1222 (MSD serology assay)
	To determine anti-SARS-CoV-2 neutralizing antibody levels in serum following 2 IM doses of AZD1222 or saline placebo<br>(Substudy and Illness Visits only)	a) Post-treatment GMTs and GMFRs from day of dosing baseline value to 28 days post each dose in SARS-CoV-2 neutralizing antibodies (wild-type assay or pseudo-neutralization assay)	a) Post-treatment GMTs and GMFRs from day of dosing baseline value to 28 days post each dose in SARS-CoV-2 neutralizing antibodies (wild-type assay or pseudo-neutralization assay)
		b) Proportion of participants who have a post-treatment seroresponse (≥ 4-fold rise in titers from day of dosing baseline value to 28 days post each dose) to AZD1222 as measured by SARS-CoV-2 neutralizing antibodies (wild-type assay or pseudo-neutralization assay)	b) Post-treatment seroresponse (≥ 4-fold rise in titers from day of dosing baseline value to 28 days post each dose) to AZD1222 as measured by SARS-CoV-2 neutralizing antibodies (wild-type assay or pseudo-neutralization assay)

Table 15 Overview of Primary and Secondary Efficacy Objectives, Endpoints, and Associated Case Definitions

Objective		Endpoint	Case Definition
Secondary	To estimate the efficacy of AZD1222 compared to saline placebo for the prevention of COVID-19 following the first dose	The incidence of SARS-CoV-2 RT-PCR-positive symptomatic illness occurring post first dose of study intervention	Participant must have RT-PCR-confirmed SARS-CoV-2 and meet the following criteria at any point from their initial illness visit at the site (Day 1) through their second illness visit (Day 14):<br>1 One or more Category A findings<br>**OR**<br>2 Two or more Category B findings<br><br>*Category A:*<br>• Pneumonia diagnosed by chest x-ray, or computed tomography scan<br>• Oxygen saturation of ≤ 94% on room air or requiring either new initiation or escalation in supplemental O_2<br>• New or worsening dyspnea/shortness of breath<br><br>*Category B:*<br>• Fever > 100 °F (> 37.8 °C) or feverishness<br>• New or worsening cough<br>• Myalgia/muscle pain<br>• Fatigue that interferes with activities of daily living<br>• Vomiting and/or diarrhea (only one finding to be counted toward endpoint definition)<br>• Anosmia and/or ageusia (only one finding to be counted toward endpoint definition)

CDC = Centers for Disease Control and Prevention; COVID-19 = coronavirus disease 2019; GMFR = geometric mean fold rise; GMT = geometric mean titer; IM = intramuscular; MSD = Meso Scale Discovery; S =Spike; RBD = receptor binding domain; RT-PCR = reverse transcriptase polymerase chain reaction; SARS-CoV-2 = severe acute respiratory syndrome-coronavirus-2.

Appendix G Abbreviations

Abbreviation or special term	Explanation
ACE2	angiotensin-converting enzyme 2
AE	adverse event
AESI	adverse event of special interest
ALT	alanine aminotransferase
AST	aspartate aminotransferase
β-hCG	beta-human chorionic gonadotropin
CDC	Centers for Disease Control and Prevention
ChAdOx1 MERS	chimpanzee adenovirus Ox1 with MERS Spike antigen
ChAdOx1 nCoV-19	name of AZD1222 when initially developed by the University of Oxford
CI	confidence interval
CoV	coronavirus
COVID-19	coronavirus disease 2019
CRO	contract research organization
CSR	clinical study report
DBL	database lock
DSMB	Data Safety Monitoring Board
E	envelope
eCRF	electronic case report form
EDC	electronic data capture
e-Diary	electronic diary
ELISA	enzyme-linked immunosorbent assay
ELISpot	enzyme-linked immunospot
FIH	first-in-human
GCP	Good Clinical Practice
GMFR	geometric mean fold rise
GMT	geometric mean titer
IB	Investigator's Brochure
ICF	informed consent form
ICH	International Council for Harmonisation
IEC	Independent Ethics Committee
IgA	immunoglobulin A
IgG	immunoglobulin G
IgM	immunoglobulin M
IM	intramuscular

Abbreviation or special term	Explanation
IMP	investigational medicinal product
IND	Investigational New Drug
IRB	Institutional Review Board
IRT	Interactive Response Technology
IV	intravenous
M	membrane
MAAE	medically attended adverse event
MERS	Middle East respiratory syndrome
MERS-CoV	Middle East respiratory syndrome coronavirus
MSD	Meso Scale Discovery
NHP	non-human primate
PBMC	peripheral blood mononuclear cell
PSRT	Protocol Safety Review Team
RBD	receptor binding domain
RRR	relative risk reduction
RT-PCR	reverse transcriptase polymerase chain reaction
S	Spike
SAE	serious adverse event
SAP	Statistical Analysis Plan
SARS-CoV	severe acute respiratory syndrome-coronavirus
SARS-CoV-2	severe acute respiratory syndrome-coronavirus 2
SoA	Schedule of Activities
TBL	total bilirubin
tPA	tissue plasminogen activator
ULN	upper limit of normal
US FDA	United States Food and Drug Administration
USA	United States of America
VE	vaccine efficacy
vp	viral particles
WHO	World Health Organization
w/v	weight/volume

Appendix H Protocol Amendment History

The Protocol Amendment Summary of Changes Table for the current amendment is located directly before the Table of Contents.

Amendment 5 (18 January 2021)

This amendment is considered to be substantial based on the criteria set forth in Article 10(a) of Directive 2001/20/EC of the European Parliament and the Council of the European Union.

Overall Rationale for the Amendment:

The principal reason for this amendment was to add an extension to the study to permit evaluation of safety, immunogenicity, and efficacy of 2 doses of AZD1222 administered 8 to 12 weeks apart to adults and adolescents. The extended dosing interval is supported by enhanced immunogenicity data, as well as a high level of protection (VE 73%) against COVID-19 demonstrated from a single dose (COVID-19 Vaccine AstraZeneca SmPC 2021). Adolescents have been added to the study population to generate valuable data on safety and immunogenicity in this age group.

Note that the new addition to the study was labeled as Part 2 (and included in a separate Appendix G) and the existing main study and its corresponding substudy and immunogenicity cohort were relabelled as Part 1.

<table>
<tr><th>Section # and Name</th><th>Description of Change</th><th>Brief Rationale</th><th>Substantial/
Non-substantial</th></tr>
<tr><td>Title Page,
1.1 Synopsis</td><td>Added “and Adolescents” to the title</td><td rowspan="2">To generate evidence to lower the indication to persons 12 years of age</td><td rowspan="2">Substantial</td></tr>
<tr><td>1.1 Synopsis,
2.1 Study Rationale</td><td>Added text to the study rationale regarding adolescents</td></tr>
<tr><td rowspan="3">1.1 Synopsis,
3 Objectives and Endpoints,
Appendix F Overview of Primary and Secondary Efficacy Objectives, Endpoints, and Associated Case Definitions (Table 15)</td><td>Removed “in adults ≥ 18 years of age” from primary objectives 1-3</td><td rowspan="2">To make the objectives applicable to both Part 1 and the newly added Part 2</td><td rowspan="2">Non-substantial</td></tr>
<tr><td>Specified that primary objective 3, secondary objectives 7-8, and exploratory objectives 6-14 apply to both Part 1 and Part 2</td></tr>
<tr><td>Added a second endpoint for secondary objective 5 and exploratory objectives 3 and 4</td><td>To show efficacy after 1 dose in severe COVID-19 illness</td><td>Substantial</td></tr>
</table>

Section # and Name	Description of Change	Brief Rationale	Substantial/ Non-substantial
1.1 Synopsis,<br>1.2 Schema,<br>1.3 Schedule of Activities<br>3 Objectives and Endpoints,<br>4.1 Overall Design,<br>4.2 Scientific Rationale for Study Design,<br>4.3 Justification for Dose,<br>5 Study Population,<br>6.1.2 Dosing Instructions,<br>6.3 Measures to Minimize Bias: Randomization and Blinding,<br>7.1 Discontinuation of study intervention<br>8 Study Assessments and Procedures,<br>9 Statistical Considerations,<br>Appendix G Part 2	Added Appendix G to describe the new Part 2 and corresponding Part 2 Substudy. Added cross-references to Appendix G.	To permit evaluation of safety, immunogenicity, and efficacy of 2 doses of AZD1222 administered 8 to 12 weeks apart in adults and adolescents	Substantial
	Relabelled the existing main study and its corresponding substudy and immunogenicity cohort as Part 1	For clarity and consistency, given the addition of Part 2	Non-substantial
1.1 Synopsis,<br>9.2 Sample Size Determination,<br>9.4 Statistical Analyses	Changed “not seropositive” to “seronegative”	Clarification	Non-substantial
3 Objectives and Endpoints,<br>Appendix G Part 2	Added footnote d to Table 5 to indicate that exploratory endpoint 5 is an exploratory endpoint for Part 1, but a secondary endpoint for Part 2.<br>Included exploratory endpoint 5 for Part 1 as a secondary endpoint for Part 2 in Appendix G	To support efficacy post first dose given the extension of the dosing interval in Part 2	Substantial

Section # and Name	Description of Change	Brief Rationale	Substantial/ Non-substantial
Appendix E Adverse Events of Special Interest (Table 13)	Added additional terms to “immunologic” of multisystem inflammatory syndrome in children and adults and added a new category “respiratory” with term of acute respiratory distress syndrome	To add new Brighton Collaboration case definitions	Non-substantial

Amendment 4 (14 December 2020)

This amendment is considered to be substantial based on the criteria set forth in Article 10(a) of Directive 2001/20/EC of the European Parliament and the Council of the European Union.

Overall Rationale for the Amendment:

The principal reason for this amendment was to incorporate revisions based on FDA feedback and clarifications of study processes. In addition, an immunogenicity cohort was added to expand upon cell-mediated immunity data.

Section # and Name	Description of Change	Brief Rationale	Substantial/ Non-substantial
Title Page	Corrected EudraCT number	Correction	Non-substantial
1.1 Synopsis,<br>3 Objectives and Endpoints,<br>9.4.1 General Considerations	Added text to indicate that participants unblinded to treatment assignment prior to having met the criteria for the primary efficacy endpoint will be censored at the date of unblinding.	Clarification	Non-substantial
1.1 Synopsis,<br>3 Objectives and Endpoints,<br>9.4.2.2 Secondary Endpoints,<br>Appendix G (Table 15)	Revised the first secondary endpoint	To permit formal statistical comparison and inclusion as key secondary endpoint	Substantial
	Identified the 4 key secondary endpoints (added footnote c)	To indicate these are the endpoints that are multiplicity protected	
1.1 Synopsis,<br>4.1 Overall Design,<br>8.5.2.3 Assessment of Mucosal Responses,<br>8.5.2.4 Assessment of Cell-mediated Immune Response	Added an immunogenicity cohort of approximately 300 participants	To expand upon cell-mediated immunity data	Substantial

Section # and Name	Description of Change	Brief Rationale	Substantial/ Non-substantial
1.1 Synopsis,<br>4.1 Overall Design,<br>9.2 Sample Size Determination	Reduced the sample size from 40 000 back to the original number of 30 0000	Based on the efficacy already seen in the pooled analysis from the Oxford studies, and the large outbreak in the United States has increased the attack rate	Substantial
1.1 Synopsis,<br>9.2 Sample Size Determination	Changed attack rate for primary analysis from 0.6% to 0.8%	Given the reduction in sample size from 40 000 to 30 000	Non-substantial
1.3 Schedule of Activities (Table 2 and Table 3)	Revised Table 2 and Table 3 captions to include "Immunogenicity Cohort"	To address addition of immunogenicity cohort to study	Non-substantial
	Added the collection of PBMCs at Day 180 for substudy and immunogenicity cohort participants to Table 3	To build on data regarding durability of cell-mediated immunity	
	Revised footnote f of Table 3 to indicate that PBMCs will be isolated from up to (instead of approximately) 300 participants in the substudy and approximately 300 participants in the immunogenicity cohort	To include the newly added immunogenicity cohort	
	In Table 3, indicated that assessment of nasal adsorption for SARS-CoV-2 mucosal responses is optional	Clarification	
	In Table 3, indicated that local and systemic predefined solicited AEs apply to the substudy only	Clarification	
1.3 Schedule of Activities (Table 2 and Table 3, footnote c),<br>6.5.2 Prohibited Concomitant Medications	Added text to indicate that experimental vaccinations, other than AZD1222, for prevention of SARS-CoV-2 or COVID-19 are prohibited. Also, noted that participants who choose to receive a licensed and/or authorized COVID-19 vaccine should inform the Investigator so it can be properly documented and they should be encouraged to continue study conduct.	To exclude experimental vaccinations other than AZD1222 and to allow participants access to emerging standard of care given the rapidly evolving COVID-19 vaccines landscape	Non-substantial

Section # and Name	Description of Change	Brief Rationale	Substantial/ Non-substantial
1.3 Schedule of Activities,<br>8.5 Human Biological Samples,<br>8.6.2 Other Study-related Biomarker Research,<br>8.7 Optional Genomics Initiative Sample,<br>Appendix D	Removed Optional Genomics Initiative assessment	Due to kit build issues during the COVID-19 pandemic and lack of sample tube availability	Non-substantial
4.1 Overall Design,<br>8.1.1 Monitoring COVID-19 Symptoms	In the COVID-19 Qualifying Symptoms table, revised "must be present for ≥ 2 days" to "must be present for ≥ 2 consecutive days"	Clarification	Non-substantial
5.1 Inclusion Criteria (Table 6)	Under combined hormonal methods, changed "injectable" to "intravaginal"	Correction	Non-substantial
	Revised footnote c to specify that sexual abstinence is considered a highly effective method only if defined as refraining from heterosexual intercourse from 28 days prior to Day 1 through 60 days following administration of the second dose of study intervention… (instead of 'during the entire period of the study')	Clarification	
5.2 Exclusion Criteria	Added a note to Exclusion Criterion 10 to indicate that the AESIs outlined in Appendix F should be considered when evaluating a participant for this exclusion criterion	The presence of these AESIs may be a safety risk to the participant, affect the ability of the participant to participate in the study, or impair interpretation of the study data	Non-substantial
6.3.3 Procedures for Unblinding	Specified the procedures in the event a study participant is contacted about receiving a licensed and/or authorized COVID-19 vaccine	Clarification provided due to recent Emergency Use Authorizations for COVID-19 vaccines	Non-substantial
6.5.2 Prohibited Concomitant Medications	Clarified that routine vaccinations are permitted (as clinically indicated) 30 days after the second vaccination	Clarification	Non-substantial

Section # and Name	Description of Change	Brief Rationale	Substantial/ Non-substantial
8.1.2 Illness Visits	Added text to indicate that if symptoms persist despite a negative test result, Investigators should provide appropriate clinical follow-up of participants, as clinically indicated	PCR test is not 100% accurate and Investigators need to provide appropriate clinical follow-up of any ill participants	Non-substantial
8.1.3 Severe COVID-19	Revised statement to indicate that the severity of COVID-19 will be evaluated in participants with symptoms of COVID-19 (instead of participants who test positive for SARS-CoV-2 by RT-PCR)	To be consistent with CRF completion guidelines asking sites to complete the endpoint CRF regardless of the local PCR result	Non-substantial
8.3.9.1 Potential Neurological AESIs	Added clarification that the Investigator should evaluate the participant and refer to a neurologist as clinically indicated	Based on Investigator feedback	Non-substantial
9.1 Statistical Hypotheses,<br>9.4.2.1 Primary Endpoint,<br>9.4.4 Methods for Multiplicity Control	Specified that if the primary endpoint results of the interim or primary analysis are statistically significant, an additional analysis with 5% alpha will be performed once all participants have completed the first year of follow-up	To evaluate long-term efficacy based on 1 year of follow-up before the final analysis	Substantial
9.3 Populations for Analyses (Table 9)	For Per-protocol analysis set, specified that erroneously-treated participants who receive one dose of active study intervention and one dose of placebo, regardless of the sequence, will be excluded from this analysis set	Clarification	Non-substantial
	For the safety analysis set, specified that a participant who has on one or several occasions received active study intervention is classified as active for all summaries, including summaries by dose	Clarification	

Section # and Name	Description of Change	Brief Rationale	Substantial/ Non-substantial
9.4 Statistical Analyses	Added text to indicate when unblinding of personnel involved in the analyses of the study may occur	Clarification for timing of Sponsor unblinding	Non-substantial
9.4.2.1 Primary Endpoint,<br>9.5 Interim Analyses	Changed “not seropositive” to “seronegative”	Clarification	Non-substantial
9.4.3.1 Primary Endpoints	Clarified that AE analyses will include intercurrent events for safety due to potential unblinding of participants for administration of licensed and/or approved SARS-CoV-2 or COVID-19 vaccine	Clarification	Non-substantial
9.4.4 Methods for Multiplicity Control	Revised to indicate how the primary efficacy endpoints and 4 key secondary endpoints will be assessed	To address the Agency’s request for having a secondary endpoint testing strategy	Substantial

Amendment 3 (26 October 2020)

This amendment is considered to be substantial based on the criteria set forth in Article 10(a) of Directive 2001/20/EC of the European Parliament and the Council of the European Union.

Overall Rationale for the Amendment:

The principal reason for this amendment was to expand AESI evaluation and oversight. Other key revisions included updating the AZD1222 safety profile, increasing the sample size, extending the beyond-use-date for the study intervention, and specifying that the substudy will only be conducted in the USA.

Section # and Name	Description of Change	Brief Rationale	Substantial/ Non-substantial
Title Page	Added ClinicalTrials.gov and EudraCT numbers	To align with updated information	Non-substantial
1.1 Synopsis	Updated Objectives and Endpoints, Overall Design, Number of Participants, Statistical Methods	To align with edits in the protocol body	Non-substantial

Section # and Name	Description of Change	Brief Rationale	Substantial/ Non-substantial
1.2 Schema, Figure 1 Study Design	Noted that the substudy will only be conducted in the USA	Due to logistical issues	Non-substantial
1.3 Schedule of Activities, Table 2	Extended Day 29 visit window from ± 3 days to -3 to + 7 days	To provide greater scheduling flexibility to sites and participants	Non-substantial
1.3 Schedule of Activities, Table 3	a) Noted that the substudy will only be conducted in the USA<br>b) Extended Day 29 visit window from ± 3 days to -3 to + 7 days<br>c) For participants who received the first dose between 28 August 2020 and 06 September 2020, extended the window for receiving the second dose	a) Due to logistical issues<br>b) To provide greater scheduling flexibility to sites and participants<br>c) To accommodate receipt of the second dose of study intervention for participants who received their first dose prior to the clinical hold	Non-substantial
1.3 Schedule of Activities, Table 4	a) Broadened optional saliva sample collection for viral shedding to all non-USA sites<br>b) Noted that PBMCs will be collected from participants where operationally feasible	Clarifications due to logistical issues	Non-substantial
2.2 Background	Updated information on the nonclinical and clinical AZD1222 development program	To align with information in the updated AZD1222 IB	Non-substantial
2.3.1 Risk Assessment	Revised important potential risks		Substantial
3 Objectives and Endpoints, Table 5	a) Revised estimand description for the primary efficacy endpoint to be conducted in the fully vaccinated analysis set, and added a secondary objective to estimate efficacy in all participants regardless of prior SARS-CoV-2 infection<br>b) Noted that the substudy will only be conducted in the USA	a) To align with the statistical plan<br>b) Due to logistical issues	Non-substantial

Section # and Name	Description of Change	Brief Rationale	Substantial/ Non-substantial
4.1 Overall Design	a) Increased the sample size from 30 000 to 40 000 participants<br>b) For participants who received the first dose between 28 August 2020 and 06 September 2020, extended the window for receiving the second dose<br>c) Noted that the substudy will be conducted only in the USA<br>d) Added a description of the independent Neurological AESI Expert Committee	a) Increased based on updated attack rates and public health emergency<br>b) To accommodate receipt of the second dose of study intervention for participants who received their first dose prior to the clinical hold<br>c) Due to logistical issues<br>d) New committee to provide advice on selected neurological AESIs	a) Substantial<br>b) Non-substantial<br>c) Non-substantial<br>d) Substantial
4.2.1 Rationale for Study Design and Participant Population	Revised enrollment status for Study COV002	Aligned with updated study status	Non-substantial
6.2.1.1 AZD1222,<br>6.2.1.2 Placebo	Extended the beyond-use-date from 4 hours to 6 hours	To align with revised use hold times based on recent microbial challenge study data	Non-substantial
8.3.9 Adverse Events of Special Interest	Expanded the AESI list and specified that AESIs will be reported to the Sponsor within one day of becoming aware of the event	To permit close monitoring and rapid communication of safety information, and further understanding of the AZD1222 safety profile	Substantial
8.3.9.1 Potential Neurological AESIs	New section for evaluation of potential neurological AESIs	For additional safety monitoring	Substantial
8.5.2.4 Assessment of Cell-mediated Immune Responses	Noted that PBMCs will be collected from participants where operationally feasible	Clarifications due to logistical issues	Non-substantial
8.6.1.2 Assessment of Viral Shedding	Broadened optional saliva sample collection for viral shedding to all non-USA sites		
9.2 Sample Size Determination	Updated to incorporate the increased sample size from 30 000 to 40 000 participants as well as the underlying assumptions	Increased based on updated attack rates and public health emergency	Substantial

Section # and Name	Description of Change	Brief Rationale	Substantial/ Non-substantial
9.3 Populations for Analyses	Added a fully vaccinated analysis set and revised the per-protocol analysis set to be based on participants in the fully vaccinated analysis set	To align with the revised statistical analyses for the primary endpoint	Non-substantial
9.4.1 General Considerations<br>9.4.2.1 Primary Endpoint<br>9.4.2.2 Secondary Endpoints	Revised analyses	To align with the current analysis plan	Non-substantial
Appendix A5 Committee Structure	Added a description of the independent Neurological AESI Expert Committee	New committee to provide advice on selected neurological AESIs	Substantial
Appendix F Adverse Events of Special Interest	a) Expanded the AESI list and provided a description of the events<br>b) Added new table listing potential immune-mediated conditions to provide more detail on this new AESI	To permit close monitoring and rapid communication of safety information and further understand the AZD1222 safety profile	Substantial
Appendix G	a) Primary endpoint: Revised to note that the statistical analysis will be conducted in participants with negative serostatus at baseline<br>b) Secondary endpoint evaluating symptomatic COVID-19 per CDC criteria: Revised the case definition to remove a minimum duration for any of the symptoms<br>c) Added a secondary objective to estimate efficacy in all participants regardless of prior SARS-CoV-2 infection	To align with statistical analyses	Non-substantial

Amendment 2 (17 September 2020)

Overall Rationale for the Amendment:

The principal reason for this amendment was to address US FDA feedback to the Investigational New Drug application. Additional changes included statistical revisions per DSMB request and other revisions to provide clarity or correct inadvertent errors. All the changes were considered non-substantial.

Section # and Name	Description of Change	Brief Rationale	Substantial/ Non-substantial
1.1 Synopsis	Updated Objectives and Endpoints, Overall Design, Statistical Methods	To align with edits in the protocol body	Non-substantial
1.3 Schedule of Activities, Table 3	Removed 'at least' to clarify that PBMCs will be isolated from approximately 300 participants	Clarification	Non-substantial
1.3 Schedule of Activities, Table 4	a) Added new footnote 'b' to indicate that collection of saliva sample for viral shedding assessment is optional in Chile/Peru and may occur after a feasibility assessment has been completed<br>b) Footnote 'c': removed the requirement for the RT-PCR test to be a rapid test	a) Feasibility of collection of saliva samples in Chile/Peru is underway<br>b) Not a requirement per protocol and provides more flexibility to the sites	Non-substantial
3 Objectives and Endpoints, Table 5	a) Revised definition of intercurrent event<br>b) Revised secondary objective 1 to assess for all SARS-COV-2 infection<br>c) Added an exploratory objective to estimate the efficacy of 2 IM doses of AZD1222 compared to placebo for COVID-19-related deaths<br>d) Added an exploratory objective to estimate the efficacy of AZD1222 compared to placebo for the prevention of COVID-19 following the first dose<br>e) Renumbered exploratory objective 12: removed 'serum' prior to B- and T-cell responses	a) To align with changes in the primary analysis<br>b) To align with analyses<br>c) Per US FDA request<br>d) Per DSMB request<br>e) To correct a misstatement	Non-substantial

Section # and Name	Description of Change	Brief Rationale	Substantial/ Non-substantial
4.1 Overall Design	Removed requirement to limit initial enrollment to participants 18 to 55 years of age	The US FDA reviewed the accrued data from the ongoing University of Oxford studies and approved enrollment in all age groups prior to enrollment of the first subject.	Non-substantial
6.2.1 Dose Preparation and Administration	Clarified description for use of AZD1222 and placebo within the beyond-use-date	To avoid confusion in the time period from the first needle puncture of the AZD1222 vial or placebo vial/IV bag to dose administration	Non-substantial
6.2.1.1 AZD1222 (Additional change)	Removed specification for a 1 mL syringe to be used for AZD1222 dose preparation	To provide flexibility in dose administration	Non-substantial
6.5 Concomitant Therapy	Specified that all concomitant medication must be recorded in the eCRF	Per US FDA request	Non-substantial
6.5.1 Permitted Concomitant Medications	Clarified the concomitant medications permitted for the treatment of COVID-19 after receiving study intervention	Per US FDA request	Non-substantial
6.5.2 Prohibited Concomitant Medications	a) For participants who received a prohibited concomitant medication, described the potential impact on participation in the study, including study withdrawal, discontinuation of study intervention, or evaluability in analysis data sets<br>b) Added immunoglobulins and/or blood product	a) Per US FDA request<br>b) For alignment with exclusion criterion 13	Non-substantial
7.1 Discontinuation of Study Intervention	a) Added new criterion for laboratory-confirmed SARS-CoV-2 infection<br>b) Revised criterion that any allergic reaction related to study intervention, regardless of severity grade, will result in study discontinuation	a) Clarification<br>b) Per US FDA request<br>c) Per US FDA request	Non-substantial

Section # and Name	Description of Change	Brief Rationale	Substantial/ Non-substantial
	c) Added new criterion for receipt of prohibited concomitant medication that may jeopardize the safety of the participant or interpretation of the data		
8.1.2 Illness Visits	Clarified that home collection requirements include digital health device and Illness e-Diary recordings, and saliva samples	Per US FDA request	Non-substantial
8.1.2.2 Digital Health Device	Described the monitoring protocols for triage and follow-up of alerts from the digital health device	Per US FDA request	Non-substantial
8.5.2.4 Assessment of Cell-mediated Immune Responses	Clarified the number of participants who will provide PBMC samples, ie, approximately 300 in the substudy and from up to approximately the first 3 000 during the Day 1 illness visit	To align with the SoA Table 3 and Table 4	Non-substantial
8.6.1.1 Virologic Assessments (additional change)	Clarified that a validated multiplexed respiratory panel may be utilized to assess the presence of respiratory pathogens	Multiplexed respiratory panel testing is not an absolute requirement	Non-substantial
8.6.1.2 Assessment of Viral Shedding	a) Clarified that saliva collection is performed utilizing a Spectrum DNA (SDNA-1000) collection kit<br>b) Removed ‘qualitative’ to describe the type of shedding assessment<br>c) Added information that collection of saliva sample for viral shedding assessment is optional in Chile/Peru and may occur after a feasibility assessment has been completed	a) Clarification<br>b) Updated due to an assay improvement<br>c) Feasibility of collection of saliva samples in Chile/Peru is underway	Non-substantial
9 Statistical Considerations<br>9.1, 9.2, 9.4, 9.4.2.1, 9.4.4, and 9.5	Amended the testing strategy, sample sizing, as well as number of events required for the interim and primary analyses	Per DSMB request	Non-substantial

Section # and Name	Description of Change	Brief Rationale	Substantial/ Non-substantial
9.3 Populations for Analysis, Table 9	Added definition for immunogenicity analysis set	To describe the population to be used for the immunogenicity assessments	Non-substantial
9.4.1 General Considerations	Revised definition of intercurrent event	To align with changes in the primary analysis	Non-substantial
9.4.2 Efficacy	a) Added reference to new Appendix G (see description for Appendix G below)<br>b) Added description of an adjudication committee that will review data of potential cases for the COVID-19-related efficacy endpoints	a) Per US FDA request<br>b) To provide clarity on the process for determining cases to be included in the COVID-19-related efficacy evaluations	Non-substantial
9.4.2.1 Primary Endpoint<br>(additional change)	Clarified a supportive analysis of the primary endpoint	Incorrect statement about censored observations removed	Non-substantial
9.4.2.2 Secondary Endpoints	a) Bullet 1: Clarified that the endpoint will assess the proportion of participants who have a post-treatment response (negative at baseline to positive post treatment with study intervention) for SARS-CoV-2 Nucleocapsid antibodies over time<br>b) Bullet 2: Included the endpoint to assess the incidence of SARS-CoV-2 RT-PCR positive symptomatic illness using the CDC criteria<br>c) Bullet 3: Clarified the University of Oxford symptom criteria used to assess the incidence of SARS-CoV-2 RT-PCR positive symptomatic illness<br>d) Specified the analyses for the incidence endpoints, and added a description of the analysis for participants positive for SARS-CoV-2 Nucleocapsid antibodies	a) To align with analyses<br>b) Corrected an inadvertent omission<br>c) Per US FDA request<br>d) Clarification	Non-substantial

Section # and Name	Description of Change	Brief Rationale	Substantial/ Non-substantial
Appendix A5 Committee Structure	Added description of an adjudication committee that will review data of potential cases for the COVID-19-related efficacy endpoints	To provide clarity on the process for determining cases to be included in the COVID-19-related efficacy evaluations	
Appendix G Overview of Primary and Secondary Efficacy Objectives, Endpoints, and Associated Case Definitions	New overview table presenting the primary and secondary efficacy objectives, endpoints, and associated case definitions	Per US FDA request	Non-substantial
Appendix I Protocol Amendment History	Moved summary of changes for Protocol Amendment 1 to this new appendix	Per protocol template	Non-substantial

CDC = Centers for Disease Control and Prevention; COVID-19 = coronavirus disease 2019; eCRF = electronic case report form; DSMB = Data Safety Monitoring Board; e-Diary = electronic diary; IM = intramuscular; NP = nasopharyngeal; PBMC = peripheral blood mononuclear cell; RT-PCR = reverse transcriptase polymerase chain reaction; SARS-CoV-2 = severe acute respiratory syndrome-coronavirus 2; SoA = schedule of activities; US FDA = United States Food and Drug Administration.

Amendment 1 (10 August 2020)

Overall Rationale for the Amendment:

Section # and Name	Description of Change	Brief Rationale	Substantial/ Non-substantial
1.1 Synopsis	Updated Objectives and Endpoints, Overall Design, and Statistical Methods	To align with edits in main body	Non-substantial
1.2 Schema	Revised the footnote to identify the number of randomized participants in each age group to participate in the substudy	To identify by age group the 3 000 randomized participants who will participate in the substudy	Non-substantial
1.3 Schedule of Activities<br>Table 2 Treatment and Follow-up Period - Main Study	Added assessment for recording any concomitant medication administered for the treatment of an SAE, MAAE, or AESI from Day 90 through Day 730	To support evaluation of SAEs, MAAEs, or AESIs	Non-substantial
1.3 Schedule of Activities<br>Table 3 Treatment and Follow-up Period - Substudy	a) Added assessment for recording any concomitant medication administered for the treatment of an SAE, MAAE, or AESI from Day 90 through Day 730	a) To support evaluation of SAEs, MAAEs, or AESIs<br>b) To help ensure PBMCs can be collected in	Non-substantial

Section # and Name	Description of Change	Brief Rationale	Substantial/ Non-substantial
	b) Footnote d: identified that 'at least 300' participants in the substudy can have PBMCs collected	participants across age groups	
2.2 Background	a) Corrected the study number for the University of Oxford South African clinical study<br>b) Updated preliminary clinical data from Study COV001 and Study COV002	To align with:<br>a) The study number correction made by the University of Oxford<br>b) The updated AZD1222 IB, Edition 1.1	Non-substantial
2.3.1 Risk Assessment	Updated common solicited AE information	To align with the updated AZD1222 IB, Edition 1.1	Non-substantial
3 Objectives and Endpoints	a) Clarified that secondary objective 6 is performed in substudy and illness visits only<br>b) Footnote a: identified the number of randomized participants in each age group to participate in the substudy	a) Humoral responses to AZD1222 are not required for main study participants<br>b) To identify by age group the 3 000 randomized participants who will participate in the substudy	Non-substantial
4.1 Overall Design	a) Clarified that enrollment will begin with participants 18 to 55 years of age, followed by older age groups after FDA review of accruing clinical data<br>b) Identified the number of randomized participants in each age group to participate in the substudy	a) To provide for review of immunogenicity data in older subjects prior to enrollment'<br>b) To identify by age group the 3 000 randomized participants who will participate in the substudy	Non-substantial
4.2.1 Rationale for Study Design and Participant Population	Updated the number of participants 56 to 69 and ≥ 70 years of age anticipated to have received AZD1222 at time of study initiation	To align with updated study status	Non-substantial
5.1 Inclusion Criteria	Corrected number formatting to separate Criteria 3 and 4	Inclusion criterion 4 was erroneously included under inclusion criterion 3	Non-substantial

Section # and Name	Description of Change	Brief Rationale	Substantial/ Non-substantial
5.2 Exclusion Criteria	a) Clarified Criterion 2 to include participants with any other demyelinating condition<br>b) Clarified Criterion 6 to state ≥ 20 mg per day vs ≥ 20 mg/kg/day of prednisone	Per NIH request<br>Correct typographical error	Non-substantial
6.1.2 Dosing Instructions	Deleted instructions to cover the injection site with a sterile dressing	Erroneously included in original protocol	Non-substantial
6.2.1 Dose Preparation and Administration;<br>6.2.1.1 AZD1222<br>6.2.1.2 Placebo	Clarified that AZD1222 and placebo doses drawn into syringes for administration must be administered according to the beyond-use-date of the vial or IV bag	To align with the beyond-use-date of the vial or IV bag as AZD1222 and placebo do not contain preservatives	Non-substantial
7.2 Participant Withdrawal from the Study	Clarified that all collected samples will be destroyed if the participant withdraws consent for use of existing samples	To align with current process	Non-substantial
8.1.1 Monitoring COVID-19 Symptoms	Clarified that investigator judgement should be used when determining which participants should initiate illness visits during the 7 days following administration of each dose of study intervention	To help ensure that qualifying symptoms for illness visits are due to potential infection with SARS-CoV-2 and not to reactogenicity of the study intervention	Non-substantial
8.1.2.2 Digital Health Device	Clarified that the monitoring team will receive and triage alerts associated with participants' digital health device data rather than the investigator	To align with current process	Non-substantial
9.1 Statistical Hypotheses<br>9.4.2.1 Primary Endpoint<br>9.4.4 Methods for Multiplicity Control<br>9.5 Interim Analyses	Clarified the statistical significance for determining vaccine efficacy	To add greater detail to the statistical analysis approach for the primary estimand	Non-substantial
9.4 Statistical Analyses	Clarified that the SAP will be approved prior to the interim analysis vs the primary analysis	Per standard practice	Non-substantial

Section # and Name	Description of Change	Brief Rationale	Substantial/ Non-substantial
Appendix A 5 Committee Structure	Added criteria for pausing the study for DSMB review	To ensure safety oversight	Non-substantial
Appendix E Toxicity Grading Scales for Solicited Adverse Events			
Table 11 Clinical Abnormalities: Vital Signs	Revised the temperature range for fever	To align with definition of fever throughout the main body	Non-substantial
Table 12 Clinical Abnormalities: Systemic	Added information for assessing severity grade of chills and deleted diarrhea	To align with solicited adverse events being collected, which includes chills but not diarrhea	Non-substantial

AESI = adverse event of special interest; MAAE = medically attended adverse event; NIH = National Institutes of Health; SAE = serious adverse event; SAP = Statistical Analysis Plan.

11 REFERENCES

CDC. (Centers for Disease Control and Prevention). Coronavirus Disease 2019 (COVID-19), Symptoms of Coronavrus. https://www.cdc.gov/coronavirus/2019-ncov/symptoms-testing/symptoms.html. Published 2020. Accessed 01 July 2020.

FDA. (Food and Drug Administration). Guidance for Industry. Toxicity grading scale for healthy adult and adolescent volunteers enrolled in preventive vaccine clinical trials. . https://www.fda.gov/media/73679/download. Published 2007. Accessed 20 June 2020.

Folegatti PM, Bittaye M, Flaxman A, Lopez FR, Bellamy D, Kupke A et al. Safety and immunogenicity of a candidate Middle East respiratory syndrome coronavirus viral-vectored vaccine: a dose-escalation, open-label, non-randomised, uncontrolled, Phase 1 trial. Lancet Infecti Dis. 2020a;20(7):816-26.

Folegatti PM, Ewer KJ, Aley PK, Angus B, Becker S, Belij-Rammerstorfer S et al. Safety and immunogenicity of the ChAdOx1 nCoV-19 vaccine against SARS-CoV-2: a preliminary report of a phase 1/2, single-blind, randomised controlled trial. Lancet. 2020b;S0140-6736(20):31604-4.

Graham SP, McLean RK, Spencer AJ, Belij-Rammerstorfer S, Wright D, Ulaszewska M et al. Evaluation of the immunogenicity of prime-boost vaccination with the replication-deficient viral vectored COVID-19 vaccine candidate ChAdOx1 nCoV-19. npj Vaccines. 2020;5(1):69.

Lambert P-H, Ambrosino DM, Andersen SR, Baric RS, Black SB, Chen RT et al. Consensus summary report for CEPI/BC March 12–13, 2020 meeting: Assessment of risk of disease enhancement with COVID-19 vaccines. Vaccine. 2020;38(31):4783-91.

Li F. Structure, Function, and Evolution of Coronavirus Spike Proteins. Annu Rev Virol. 2016;3(1):237-61.

Lu R, Zhao X, Li J, Niu P, Yang B, Wu H et al. Genomic characterisation and epidemiology of 2019 novel coronavirus: implications for virus origins and receptor binding. Lancet. 2020;395(10224):565-74.

NIH. (National Institutes of Health). Guidelines for the use of antiretroviral agents in adults and adolescents with HIV. https://aidsinfo.nih.gov/guidelines. Published 2020. Accessed 10 August 2020.

Rajão DS, Chen H, Perez DR, Sandbulte MR, Gauger PC, Loving CL et al. Vaccine-associated enhanced respiratory disease is influenced by haemagglutinin and neuraminidase in whole inactivated influenza virus vaccines. The Journal of general virology. 2016;97(7):1489-99.

Rovira À, Wattjes MP, Tintoré M, Tur C, Yousry TA, Sormani MP et al. MAGNIMS consensus guidelines on the use of MRI in multiple sclerosis—clinical implementation in the diagnostic process. Nature Reviews Neurology. 2015;11(8):471-82.

Schmitt-Thompson. Clinical Content. COVID-19 Resources. 2020. https://www.stcc-triage.com/covid-19-resources. Published 2020. Accessed 01 September 2020.

SPEAC. (Safety Platform for Emergency Vaccines) D2.3 Priority list of adverse events of special interest: COVID-19. Work Package: WP2 Standards and Tools. v1.1. 05 March 2020. https://media.tghn.org/articles/COVID-19_AESIs_SPEAC_V1.1_5Mar2020.pdf. Published 2020. Accessed 14 June 2020.

van Doremalen N, Lambe T, Spencer A, Belij-Rammerstorfer S, Purushotham JN, Port JR et al. ChAdOx1 nCoV-19 vaccine prevents SARS-CoV-2 pneumonia in rhesus macaques. Nature [Internet]. 2020 30 July 2020 [cited Access Date]. Available from: https://doi.org/10.1038/s41586-020-2608-y.

Waldrop G, Doherty M, Vitoria M, Ford N. Stable patients and patients with advanced disease: consensus definitions to support sustained scale up of antiretroviral therapy. Tropical medicine & international health : TM & IH. 2016;21(9):1124-30.

WHO. (World Health Organization) COVID-19 weekly epidemiological update. 18 October 2020. https://www.who.int/docs/default-source/coronaviruse/situation-reports/20201020-weekly-epi-update-10.pdf?sfvrsn=58786643_26&download=true. Published 2020. Accessed 23 October 2020.

Zhou P, Yang X-L, Wang X-G, Hu B, Zhang L, Zhang W et al. A pneumonia outbreak associated with a new coronavirus of probable bat origin. Nature. 2020.

Zhu N, Zhang D, Wang W, Li X, Yang B, Song J et al. A novel coronavirus from patients with pneumonia in China, 2019. N Engl J Med. 2020;382(8):727-33.

Zou G. A modified poisson regression approach to prospective studies with binary data. Am J Epidemiol. 2004;159(7):702-6.

SIGNATURE PAGE

This is a representation of an electronic record that was signed electronically and this page is the manifestation of the electronic signature

**Document Name:** d8110c00001-csp-amendment-6		
**Document Title:**	D8110C00001 Clinical Study Protocol Amendment 6	
**Document ID:**	Doc ID-004315165	
**Version Label:**	9.0 CURRENT LATEST APPROVED	
**Server Date (dd-MMM-yyyy HH:mm 'UTC'Z)**	**Signed by**	**Meaning of Signature**
22-Feb-2021 20:22 UTC	[redacted]	Content Approval
19-Feb-2021 15:52 UTC	[redacted]	Content Approval
22-Feb-2021 18:48 UTC	[redacted]	Content Approval
19-Feb-2021 16:45 UTC	[redacted]	Content Approval

Notes: (1) Document details as stored in ANGEL, an AstraZeneca document management system.

STATISTICAL ANALYSIS PLAN

D8110C00001

A PHASE III RANDOMIZED, DOUBLE-BLIND, PLACEBO-CONTROLLED MULTICENTER STUDY IN ADULTS TO DETERMINE THE SAFETY, EFFICACY, AND IMMUNOGENICITY OF AZD1222, A NON-REPLICATING CHADOX1 VECTOR VACCINE, FOR THE PREVENTION OF COVID-19

AUTHOR: [redacted]

VERSION NUMBER AND DATE: V3.0, 28FEB2021

Copyright © 2009, 2010, 2012, 2016, 2018, 2019 IQVIA. All rights reserved. The contents of this document are confidential and proprietary to IQVIA Holdings Inc. and its subsidiaries. Unauthorized use, disclosure or reproduction is strictly prohibited.

STATISTICAL ANALYSIS PLAN SIGNATURE PAGE

Statistical Analysis Plan v3.0 (dated 28Feb2021) for protocol D8110C00001.

	Name	Signature	Date (DDMmmYYYY)
**Author:**	[redacted]	Refer to eSignature	
**Position:**	[redacted]		
**Company:**	IQVIA		

Upon review of this document, the undersigned approves this version of the Statistical Analysis Plan, authorizing that the content is acceptable for the reporting of this study.

	Name	Signature	Date (DDMmmYYYY)
**Approved by:**	[redacted]	Refer to eSignature	
**Position:**	[redacted]		
**Company:**	AstraZeneca		
**Approved by:**	[redacted]	Refer to eSignature	
**Position:**	[redacted]		
**Company:**	AstraZeneca		

Copyright © 2009, 2010, 2012, 2016, 2018, 2019 IQVIA. All rights reserved. The contents of this document are confidential and proprietary to IQVIA Holdings Inc. and its subsidiaries. Unauthorized use, disclosure or reproduction is strictly prohibited.

MODIFICATION HISTORY

Date of the Document Modification / Version	Author	Significant Changes from Previous Version
06Oct2020/<br>Version 1.0	[redacted]	Initial version.
03Feb2020/<br>Version 2.0	[redacted]	Note: editorial updates are not listed.<br>• Section 2.2: New secondary endpoint is added per PA3 to evaluate efficacy regardless of prior infection; Revised the first secondary endpoint per PA4 to permit formal statistical comparison; Identified the 4 key secondary endpoints per PA4 to indicate these are the endpoints that are multiplicity protected.<br>• Section 2.4: Population definitions are updated to clarify that participant must be seronegative; Updates to align with Section 2.2; Added details for inclusion of unblinding/EUA vaccination as intercurrent event.<br>• Sections 3.1-3.2: Updates to align with PA3 and PA4.<br>• Section 4: Description of an analysis performed by OWS to evaluate immunological markers as correlates of risk among vaccinees and towards the development of surrogates of protection .<br>• Sections 4.1-4.3: Updated to align with PA3 and PA4. Clarification added for role of DSMB, including oversight of the interim analysis. Additional details for interim and primary analyses, including new analysis planned once all participants have completed their first year of follow up.<br>• Section 5.1: Added details for exclusion of participant enrolled at two sites and another sponsor COVID-19 vaccine trial.<br>• Section 5.3-5.5: Definition of per protocol population is updated to use FVS

Copyright © 2009, 2010, 2012, 2016, 2018, 2019 IQVIA. All rights reserved. The contents of this document are confidential and proprietary to IQVIA Holdings Inc. and its subsidiaries. Unauthorized use, disclosure or reproduction is strictly prohibited.

		per PA4. Updates to clarify approach for patients with dosing errors.<br>• Section 7.3,7.4 are updated to include additional analysis performed once all participants have completed their first year of follow up.<br>• Section 7.4: Updated to reflect multiplicity protection for key secondary endpoints per PA4.<br>• Section 7.6, 7.7: Country is removed from the statistical models. Clarification added for possible issues of non-convergence due to sparse data for subgroup levels (see also 16.1.6).<br>• Section 9.1: Disposition updated to include patients unblinded to treatment assignment.<br>• Section 10: Added demographic summary based on SAF for the substudy and IAS for the substudy.<br>• Section 11: Added summary of baseline co-morbidities for FVS.<br>• Section 15: Added summary of the interval between dose 1 and dose 2.<br>• Section 16: Updated to add details for handling discordance between local and central SARS-CoV-2 RT-PCR results. Includes plan to address unblinding/EUA vaccine as intercurrent events. Added summary of duration of FU time for FAS and FVS.<br>• Section 16.1.3: Updated to use log of total number of participants for each combination of treatments and strata as offset for stratified exact poisson model. Additional details provided for handling sparse data and non-convergence issues. Clarification added for calculation of the follow up time.<br>• Section 16.1.4: Removed multiple imputation analysis when AZD1222 imputed as having event and Placebo imputed as no event and also when AZD1222 imputed as no event and Placebo imputed as having event.<br>• Section 16.1.5: Updated to include repeat of primary endpoint analysis

Copyright © 2009, 2010, 2012, 2016, 2018, 2019 IQVIA. All rights reserved. The contents of this document are confidential and proprietary to IQVIA Holdings Inc. and its subsidiaries. Unauthorized use, disclosure or reproduction is strictly prohibited.

		excluding participants with out-of-window vaccination due to the clinical hold.<br>• Section 16.1.6: Replace FAS by FVS regardless of baseline serostatus for subgroup analysis for serostatus at baseline; Clarification added for subgroup analysis methods.<br>• Section 16.2.1.1-16.2.1.6, 16.2.3: Updated to incorporate the updates for key secondary endpoints and the new secondary endpoints per PA3 and PA4. Also added supportive analysis for severe COVID endpoint post first dose.<br>• Section 16.2.3: Added subgroup analysis by baseline serostatus for key secondary endpoints.<br>• Section 16.3.1.3, 16.3.1.4: Added supportive analysis for COVID-19-related hospitalization and COVID-19-related ICU post first dose.<br>• Section 17: Details added for treating unblinding/EUA vaccine as an intercurrent event. Updated to use log base 2 for GMT calculation. Clarification added that participants who do not receive dose 2 are excluded from summaries at timepoints post-dose 2.<br>• Section 17.1.1, Section 17.1.2: Added summaries for participants in the substudy by age group and by clinical hold status.<br>• Section 18: Details added throughout for treating unblinding/EUA vaccine as an intercurrent event, including addition of summaries of events in the double-blind, unblinded, and overall study periods for SAEs, MAAEs and AESIs.<br>• Section 18.1.7: AESIs were updated per PA3 and PA4. Summary of AESIs by severity grade and summary of related AESIs by severity grade were added. Added summaries of AESIs by age group, sex and race.<br>• Section 18.1.8: Added additional summaries of adverse event.<br>• Section 18.2: Updated to reflect solicited events are captured on days 2-8,

Copyright © 2009, 2010, 2012, 2016, 2018, 2019 IQVIA. All rights reserved. The contents of this document are confidential and proprietary to IQVIA Holdings Inc. and its subsidiaries. Unauthorized use, disclosure or reproduction is strictly prohibited.

		with 24 hour recall period. Added summaries by age groups.<br>• Appendix 6: Updated to reflect symptom criteria for CDC endpoint definition.<br>• Appendix 7: New appendix for AESI.
28Feb2020/ Version 3.0	[redacted]	Note: editorial updates are not listed.<br>• Updates to align with protocol amendment 6. Exploratory endpoint (the incidence of SARS-CoV-2 RT-PCR positive symptomatic illness occurring post first dose of study intervention) promoted to a secondary endpoint.<br>• Updated the threshold for frequent adverse events from 2% to 1%.<br>• Clarifications added regarding the calculation of follow-up time.

Copyright © 2009, 2010, 2012, 2016, 2018, 2019 IQVIA. All rights reserved. The contents of this document are confidential and proprietary to IQVIA Holdings Inc. and its subsidiaries. Unauthorized use, disclosure or reproduction is strictly prohibited.

TABLE OF CONTENTS

1. INTRODUCTION 11

2. STUDY OBJECTIVES 11

2.1. Primary Objectives 11

2.2. Secondary Objectives 12

2.3. Exploratory Objectives 12

2.4. Estimands 15

3. STUDY DESIGN 27

3.1. General Description 27

3.2. Schedule of Events 28

3.3. Changes to Analyses from Protocol 28

4. PLANNED ANALYSES 29

4.1. Data Monitoring Committee 29

4.2. Interim Analysis (IA) 29

4.3. Primary Analysis 30

4.4. Final Analysis 30

5. ANALYSIS SETS 31

5.1. All Participants Analysis Set 31

5.2. Full Analysis Set 31

5.3. Fully Vaccinated Analysis Set 31

5.4. Per-protocol Analysis Set 32

5.5. Safety Analysis Set 32

Copyright © 2009, 2010, 2012, 2016, 2018, 2019 IQVIA. All rights reserved. The contents of this document are confidential and proprietary to IQVIA Holdings Inc. and its subsidiaries. Unauthorized use, disclosure or reproduction is strictly prohibited.

5.6. Immunogenicity Analysis Set 32

6. GENERAL CONSIDERATIONS 33

6.1. Reference Start Date and Study Day 33

6.2. Baseline 33

6.3. Unscheduled Visits, Retests, and Early Termination Data 34

6.4. Windowing Conventions 34

6.5. Common Calculations 35

7. STATISTICAL CONSIDERATIONS 36

7.1. Sample Size Calculation 36

7.2. Missing data 37

7.3. Statistical Tests 37

7.4. Multiple Comparisons/ Multiplicity 38

7.5. Multicenter Studies 40

7.6. Adjustments for Covariates and Factors to be Included in Analyses 40

7.7. Examination of Subgroups 40

7.8. Software Version 41

8. OUTPUT PRESENTATIONS 41

9. DISPOSITION AND WITHDRAWALS 41

9.1. Disposition 41

9.2. Protocol Deviations 42

10. DEMOGRAPHIC AND OTHER BASELINE CHARACTERISTICS 42

10.1. Derivations 43

Copyright © 2009, 2010, 2012, 2016, 2018, 2019 IQVIA. All rights reserved. The contents of this document are confidential and proprietary to IQVIA Holdings Inc. and its subsidiaries. Unauthorized use, disclosure or reproduction is strictly prohibited.

11. MEDICAL HISTORY....................43

12. CONCOMITANT ILLNESSES....................44

13. MEDICATIONS....................44

14. EXPOSURE TO STUDY INTERVENTION....................45

15. COMPLIANCE WITH STUDY INTERVENTION....................45

16. EFFICACY ENDPOINTS....................45

16.1. Primary Efficacy....................46
16.1.1. Primary Efficacy Endpoint....................46
16.1.2. Missing Data Imputation Method for Primary Efficacy Endpoint....................47
16.1.3. Primary Analysis of Primary Efficacy Endpoint....................48
16.1.4. Sensitivity Analyses for Primary Efficacy Endpoint....................50
16.1.5. Supplementary Analyses for Primary Efficacy Endpoint....................51
16.1.6. Subgroup Analyses for Primary Efficacy Endpoint....................52

16.2. Secondary Efficacy....................53
16.2.1. Secondary Efficacy Endpoints....................53
16.2.1.1. The Incidence of the First Post-treatment Response (Negative at Baseline to Positive Post Treatment with Study Intervention) for SARS-CoV-2 Nucleocapsid Antibodies Occurring ≥ 15 Days Post Second Dose of Study Intervention....................53
16.2.1.2. The Incidence of the First Case of SARS-CoV-2 RT-PCR Positive Symptomatic Illness Occurring ≥ 15 Days Post Second Dose of Study Intervention Using CDC Criteria....................53
16.2.1.3. Incidence of the First Case of SARS-CoV-2 RT-PCR-positive Symptomatic Illness Occurring ≥ 15 Days Post Second Dose of Study Intervention Using University of Oxford Defined Symptom Criteria....................54
16.2.1.4. Incidence of the First Case of SARS-CoV-2 RT-PCR Positive Symptomatic Illness Occurring ≥ 15 Days Post Second Dose of Study Intervention Regardless of Evidence of Prior SARS-CoV-2 Infection....................54
16.2.1.5. Incidence of SARS-CoV-2 RT-PCR-positive Severe or Critical Symptomatic Illness Occurring ≥ 15 Days Post Second Dose of Study Intervention....................54
16.2.1.6. Incidence of COVID-19-related Emergency Department Visits Occurring ≥ 15 Days Post Second Dose of Study Intervention....................55
16.2.1.7. The Incidence of COVID-19 SARS-CoV-2 RT-PCR-positive Symptomatic Illness Occurring Post First Dose of Study Intervention....................55
16.2.2. Missing Data Imputation Method for Secondary Efficacy Endpoints....................55
16.2.3. Analysis of Secondary Efficacy Endpoints....................56

16.3. Exploratory Efficacy....................56
16.3.1. Analysis of Exploratory Efficacy Endpoints....................57
16.3.1.1. The Incidence of All-cause Mortality Occurring from Day 1 through Day 730....................57
16.3.1.2. The Incidence of COVID-19-related Deaths Occurring from Day 1 through Day 730....................58

Copyright © 2009, 2010, 2012, 2016, 2018, 2019 IQVIA. All rights reserved. The contents of this document are confidential and proprietary to IQVIA Holdings Inc. and its subsidiaries. Unauthorized use, disclosure or reproduction is strictly prohibited.

16.3.1.3. The Incidence of COVID-19-related Hospitalizations Occurring ≥ 15 Days Post Second Dose of Study Intervention 58
16.3.1.4. The Incidence of COVID-19-related ICU Admissions Occurring ≥ 15 Days Post Second Dose of Study Intervention 59
16.3.1.5. Viral Genome Copies in NP Swabs Collected at Illness Visits as Determined by qRT-PCR 59
16.3.1.6. Genotypic Analysis of SARS-CoV-2 from NP Swabs Collected on Day 1 Illness visit 59
16.3.1.7. Duration of SARS-CoV-2 Shedding in Saliva over Time 60
16.3.1.8. Biophysical Parameters, including but not limited to Serial Measurements of Skin Temperature, Heart rate, Respiratory Rate, Blood Oxygen Saturation, and Physical Activity, Recorded Using a Biosensor from Illness Visits Day 1 through Day 28 61
16.3.1.9. Symptoms Recorded by Participants in an Illness e-Diary from Illness Visits Day 2 through Day 28 61

17. IMMUNOGENICITY ENDPOINTS 61

17.1. Analysis of Immunogenicity Endpoints 63
17.1.1. Geometric Mean Titers and Geometric Mean Fold Rise 63
17.1.2. Seroresponse Rate 64
17.1.3. Missing Data Imputation Method for Immunogenicity Endpoints 65

17.2. Analysis of Cell-mediated Immune Response Endpoints 65

18. SAFETY ENDPOINTS 66

18.1. Adverse Events 66
18.1.1. Adverse Events 68
18.1.1.1. Severity for AEs 68
18.1.1.2. Relationship to Study Intervention/Non-Study Intervention/Procedure 68
18.1.2. Serious Adverse Events 69
18.1.3. AEs Leading to Discontinuation of Study Intervention 69
18.1.4. AEs Leading to Discontinuation of Study 70
18.1.5. AEs with Outcome of Death 70
18.1.6. Medically Attended Adverse Events 71
18.1.7. Adverse Events of Special Interest 71
18.1.8. Additional Summaries of Adverse events 72

18.2. Solicited Adverse Events (Only for Substudy) 72

18.3. Vital Signs 73

18.4. Physical Examination 74
18.4.1. General Physical Examination 74
18.4.2. Chest Imaging 74

19. REFERENCES 75

Copyright © 2009, 2010, 2012, 2016, 2018, 2019 IQVIA. All rights reserved. The contents of this document are confidential and proprietary to IQVIA Holdings Inc. and its subsidiaries. Unauthorized use, disclosure or reproduction is strictly prohibited.

APPENDIX 1. **Partial Date Conventions** 76

APPENDIX 2. **Programming Conventions for Outputs** 79

APPENDIX 3. **Clinical Abnormalities** 82

APPENDIX 4. **Clinical Abnormalities** 83

APPENDIX 5. **Clinical Abnormalities: Systemic** 84

APPENDIX 6. **COVID-19 Symptoms Per CDC Criteria** 85

APPENDIX 7. **Adverse Events of Special Interest** 86

1. Introduction

This statistical analysis plan (SAP) describes the rules and conventions to be used in the presentation and analysis of efficacy and safety data for protocol D8110C00001. It describes the data to be summarized and analyzed, including specifics of the statistical analyses to be performed.

This SAP is based on protocol Amendment 6.0, dated 19Feb2021.

2. Study Objectives

2.1. Primary Objectives

The primary objectives are:

- To estimate the efficacy of 2 IM doses of AZD1222 compared to saline placebo for the prevention of COVID-19
- To assess the safety and tolerability of 2 IM doses of AZD1222 compared to saline placebo
- To assess the reactogenicity of 2 IM doses of AZD1222 compared to saline placebo (Substudy only)

Copyright © 2009, 2010, 2012, 2016, 2018, 2019 IQVIA. All rights reserved. The contents of this document are confidential and proprietary to IQVIA Holdings Inc. and its subsidiaries. Unauthorized use, disclosure or reproduction is strictly prohibited.

2.2. Secondary Objectives

The secondary objectives are:

- To estimate the efficacy of 2 IM doses of AZD1222 compared to saline placebo for the prevention of SARS-CoV-2 infection*
- To estimate the efficacy of 2 IM doses of AZD1222 compared to saline placebo for the prevention of symptomatic COVID-19 using CDC criteria
- To estimate the efficacy of 2 IM doses of AZD1222 compared to saline placebo for the prevention of University of Oxford-defined symptomatic COVID-19
- To estimate the efficacy of 2 IM doses of AZD1222 compared to saline placebo in the prevention of COVID-19 in all study participants, regardless of evidence of prior SARS-CoV-2 infection*
- To estimate the efficacy of 2 IM doses of AZD1222 compared to saline placebo for the prevention of severe or critical symptomatic COVID-19*
- To estimate the efficacy of 2 IM doses of AZD1222 compared to saline placebo for the prevention of COVID-19-related Emergency Department visits*
- To assess antibody responses to AZD1222 S antigen following 2 IM doses of AZD1222 or saline placebo (Substudy and Illness Visits only)
- To determine anti-SARS-CoV-2 neutralizing antibody levels in serum following 2 IM doses of AZD1222 or saline placebo (Substudy and Illness Visits only)
- To estimate the efficacy of AZD1222 compared to saline placebo for the prevention of COVID-19 following the first dose

* Key secondary endpoints, multiplicity protected.

2.3. Exploratory Objectives

The exploratory objectives are:

- To estimate the efficacy of 2 IM doses of AZD1222 compared to saline placebo for the all-cause

Copyright © 2009, 2010, 2012, 2016, 2018, 2019 IQVIA. All rights reserved. The contents of this document are confidential and proprietary to IQVIA Holdings Inc. and its subsidiaries. Unauthorized use, disclosure or reproduction is strictly prohibited.

mortality

- To estimate the efficacy of 2 IM doses of AZD1222 compared to saline placebo for COVID-19-related deaths
- To estimate the efficacy of 2 IM doses of AZD1222 compared to saline placebo for the prevention of COVID-19-related hospitalizations
- To estimate the efficacy of 2 IM doses of AZD1222 compared to saline placebo for the prevention of COVID-19-related ICU admissions
- To quantify SARS-Cov-2 viral loads in infected participants treated with 2 IM doses of AZD1222 or saline placebo (Illness Visits only)
- To characterize sequence variations in SARS-CoV-2 through genotypic analyses in participants treated with 2 IM doses of AZD1222 or saline placebo (Illness Visits only)
- To quantify duration of viral shedding in symptomatic SARS-CoV-2 infected participants treated with 2 IM doses of AZD1222 or saline placebo (Illness Visits only)
- To assess the biometric profiles associated with COVID-19 using a biosensor in participants treated with 2 IM doses of AZD1222 or saline placebo (Illness Visits only)
- To assess symptoms associated with COVID-19 using an e-Diary in participants treated with 2 IM doses of AZD1222 or saline placebo (Illness Visits only)
- To assess SARS-CoV-2 specific antibodies in an ACE2 competition assay following 2 IM doses of AZD1222 or saline placebo (Substudy only)
- To assess B- and T-cell responses following 2 IM doses of AZD1222 or saline placebo (Substudy only)
- To assess SARS-CoV-2 antibodies in nasal secretions following 2 IM doses of AZD1222 or saline placebo (Substudy only)
- To assess anti-vector responses to the ChAdOx-1 adenovirus vector following 2 IM doses of AZD1222 or saline placebo (Substudy only)
- To assess additional immune responses following 2 IM doses of AZD1222 or saline placebo

Copyright © 2009, 2010, 2012, 2016, 2018, 2019 IQVIA. All rights reserved. The contents of this document are confidential and proprietary to IQVIA Holdings Inc. and its subsidiaries. Unauthorized use, disclosure or reproduction is strictly prohibited.

Copyright © 2009, 2010, 2012, 2016, 2018, 2019 IQVIA. All rights reserved. The contents of this document are confidential and proprietary to IQVIA Holdings Inc. and its subsidiaries. Unauthorized use, disclosure or reproduction is strictly prohibited.

2.4. Estimands

Table A: List of Estimands

Label	Attributes				
	**Definition Population**	**Treatment Condition of Interest**	**Variable/Endpoint**	**Intercurrent event handling strategy**	**Population-level summary measure**
The efficacy of 2 IM doses of AZD1222 compared to saline placebo for the prevention of COVID-19	Adults ≥ 18 years of age who are healthy or have medically-stable chronic diseases and are at increased risk for SARS-CoV-2 acquisition and COVID-19, and who are seronegative at baseline	2 IM doses of AZD1222 or Placebo	A binary response, whereby a participant is defined as a COVID-19 case if their first case of SARS-CoV-2 RT-PCR-positive symptomatic illness occurs ≥ 15 days post second dose of study intervention. Otherwise, a participant is not defined as a COVID-19 case.	For participants who withdraw from the study prior to having met the criteria for the primary efficacy endpoint, absence of data following these participants' withdrawal will be treated as missing (i.e. counted as not having met the criteria); Participants who withdraw before 15 days post second dose or who have a case prior to 15 days post second dose will be excluded from primary endpoint analysis. Participants who (1) were unblinded to treatment assignment or (2) received COVID-19 licensed vaccine but were not	Vaccine Efficacy (VE), calculated as 1-relative risk. (Relative risk is the incidence in the vaccine group relative to the incidence in the control group.)

Copyright © 2009, 2010, 2012, 2016, 2018, 2019 IQVIA. All rights reserved. The contents of this document are confidential and proprietary to IQVIA Holdings Inc. and its subsidiaries. Unauthorized use, disclosure or reproduction is strictly prohibited.

Label	Attributes				
	**Definition Population**	**Treatment Condition of Interest**	**Variable/Endpoint**	**Intercurrent event handling strategy**	**Population-level summary measure**
				unblinded to treatment assignment, in both cases prior to having met the criteria for the primary efficacy endpoint will be censored at the date of unblinding/ licensed COVID-19 vaccine administration, whichever is earlier.	
The safety and tolerability of 2 IM doses of AZD1222 compared to saline placebo	Adults ≥ 18 years of age who are healthy or have medically-stable chronic diseases, and are at increased risk for SARS-CoV-2 acquisition and COVID-19	2 IM doses of AZD1222 or Placebo	a) Incidence of AEs for 28 days post each dose of study intervention<br><br>b) Incidence of SAEs, MAAEs, and AESIs from Day 1 post treatment through Day 730	Participants unblinded to treatment assignment or participants who received licensed COVID-19 vaccine but were not unblinded will be censored at the date of unblinding/ COVID-19 licensed vaccine administration, whichever is earlier.	Number and percentages of participants who have the incidence; Number of the events.

Copyright © 2009, 2010, 2012, 2016, 2018, 2019 IQVIA. All rights reserved. The contents of this document are confidential and proprietary to IQVIA Holdings Inc. and its subsidiaries. Unauthorized use, disclosure or reproduction is strictly prohibited.

Label	Attributes				
	**Definition Population**	**Treatment Condition of Interest**	**Variable/Endpoint**	**Intercurrent event handling strategy**	**Population-level summary measure**
The reactogenicity of 2 IM doses of AZD1222 compared to saline placebo (Substudy only)	Adults ≥ 18 years of age who are healthy or have medically-stable chronic diseases and are at increased risk for SARS-CoV-2 acquisition and COVID-19	2 IM doses of AZD1222 or Placebo	Incidence of local and systemic solicited AEs for 7 days post each dose of study intervention	Participants unblinded to treatment assignment/received licensed COVID-19 vaccine but not unblinded prior to having completed 7 days of follow up post each dose will be censored at the date of unblinding/ licensed COVID-19 vaccine administration, whichever is earlier.	Number and percentages of participants who have the incidence.

Copyright © 2009, 2010, 2012, 2016, 2018, 2019 IQVIA. All rights reserved. The contents of this document are confidential and proprietary to IQVIA Holdings Inc. and its subsidiaries. Unauthorized use, disclosure or reproduction is strictly prohibited.

The efficacy of 2 IM doses of AZD1222 compared to saline placebo for the prevention of SARS-CoV-2 infection	Adults ≥ 18 years of age who are healthy or have medically-stable chronic diseases and are at increased risk for SARS-CoV-2 acquisition and COVID-19, and who are seronegative at baseline.	2 IM doses of AZD1222 or Placebo	A binary response, whereby a participant has at least one post-treatment response (negative at baseline to positive post treatment with study intervention) for SARS-CoV-2 Nucleocapsid antibodies occurring ≥ 15 days post second dose of study intervention.	For participants who withdraw from the study prior to having met the criteria for the endpoint, absence of data following these participants' withdrawal will be treated as missing (i.e. counted as not having met the criteria); Participants who withdraw before 15 days post second dose or who have a case prior to 15 days post second dose will be excluded from analysis. Participants who (1) were unblinded to treatment assignment or (2) received COVID-19 licensed vaccine but were not unblinded to treatment assignment, in both cases prior to having met the criteria for the primary efficacy endpoint will be censored at the date of unblinding/ licensed COVID-19 vaccine administration, whichever is earlier.	Vaccine Efficacy (VE), calculated as 1-relative risk. (Relative risk is the incidence in the vaccine group relative to the incidence in the control group.)
The efficacy of 2 IM doses of AZD1222	Adults ≥ 18 years of age who are healthy or have medically-stable chronic	2 IM doses of AZD1222 or Placebo	A binary response, whereby a participant has least one case of SARS-CoV-2 RT-PCR	For participants who withdraw from the study prior to having met the criteria for the endpoint, absence of data following	Vaccine Efficacy (VE), calculated as 1-relative risk. (Relative risk is the

Copyright © 2009, 2010, 2012, 2016, 2018, 2019 IQVIA. All rights reserved. The contents of this document are confidential and proprietary to IQVIA Holdings Inc. and its subsidiaries. Unauthorized use, disclosure or reproduction is strictly prohibited.

	Attributes				
**Label**	**Definition Population**	**Treatment Condition of Interest**	**Variable/Endpoint**	**Intercurrent event handling strategy**	**Population-level summary measure**
compared to saline placebo for the prevention of symptomatic COVID-19 using CDC criteria	diseases and are at increased risk for SARS-CoV-2 acquisition and COVID-19, and who are seronegative at baseline.		positive symptomatic illness occurring ≥ 15 days post second dose of study intervention using CDC criteria.	these participants' withdrawal will be treated as missing (i.e. counted as not having met the criteria); Participants who withdraw before 15 days post second dose or who have a case prior to 15 days post second dose will be excluded from analysis. Participants who (1) were unblinded to treatment assignment or (2) received COVID-19 licensed vaccine but were not unblinded to treatment assignment, in both cases prior to having met the criteria for the primary efficacy endpoint will be censored at the date of unblinding/ licensed COVID-19 vaccine administration, whichever is earlier.	incidence in the vaccine group relative to the incidence in the control group.)

Copyright © 2009, 2010, 2012, 2016, 2018, 2019 IQVIA. All rights reserved. The contents of this document are confidential and proprietary to IQVIA Holdings Inc. and its subsidiaries. Unauthorized use, disclosure or reproduction is strictly prohibited.

The efficacy of 2 IM doses of AZD1222 compared to saline placebo for the prevention of University of Oxford-defined symptomatic COVID-19	Adults ≥ 18 years of age who are healthy or have medically-stable chronic diseases and are at increased risk for SARS-CoV-2 acquisition and COVID-19, and who are seronegative at baseline.	2 IM doses of AZD1222 or Placebo	A binary response, whereby a participant has the at least one case of SARS-CoV-2 RT-PCR positive symptomatic illness occurring ≥ 15 days post second dose of study intervention using University of Oxford-defined symptom criteria.	For participants who withdraw from the study prior to having met the criteria for the endpoint, absence of data following these participants' withdrawal will be treated as missing (i.e. counted as not having met the criteria); Participants who withdraw before 15 days post second dose or who have a case prior to 15 days post second dose will be excluded from analysis. Participants who (1) were unblinded to treatment assignment or (2) received COVID-19 licensed vaccine but were not unblinded to treatment assignment, in both cases prior to having met the criteria for the primary efficacy endpoint will be censored at the date of unblinding/ licensed COVID-19 vaccine administration, whichever is earlier.	Vaccine Efficacy (VE), calculated as 1-relative risk. (Relative risk is the incidence in the vaccine group relative to the incidence in the control group.)
The efficacy of 2 IM doses of AZD1222	Adults ≥ 18 years of age who are healthy or have medically-stable chronic	2 IM doses of AZD1222 or Placebo	A binary response, whereby a participant is defined as a COVID-19 case if their first	For participants who withdraw from the study prior to having met the criteria for the endpoint, absence of data following	Vaccine Efficacy (VE), calculated as 1-relative risk. (Relative risk is the

Copyright © 2009, 2010, 2012, 2016, 2018, 2019 IQVIA. All rights reserved. The contents of this document are confidential and proprietary to IQVIA Holdings Inc. and its subsidiaries. Unauthorized use, disclosure or reproduction is strictly prohibited.

	Attributes				
**Label**	**Definition Population**	**Treatment Condition of Interest**	**Variable/Endpoint**	**Intercurrent event handling strategy**	**Population-level summary measure**
compared to saline placebo for the prevention of COVID-19 in all study participants, regardless of evidence of prior SARS-CoV-2 infection	diseases and are at increased risk for SARS-CoV-2 acquisition and COVID-19.		case of SARS-CoV-2 RT-PCR-positive symptomatic illness occurs ≥ 15 days post second dose of study intervention regardless of evidence of prior SARS-CoV-2 infection. Otherwise, a participant is not defined as a COVID-19 case.	these participants' withdrawal will be treated as missing (i.e. counted as not having met the criteria); Participants who withdraw before 15 days post second dose will be excluded from primary endpoint analysis. Participants who (1) were unblinded to treatment assignment or (2) received COVID-19 licensed vaccine but were not unblinded to treatment assignment, in both cases prior to having met the criteria for the primary efficacy endpoint will be censored at the date of unblinding/ licensed COVID-19 vaccine administration, whichever is earlier.	incidence in the vaccine group relative to the incidence in the control group.)

Copyright © 2009, 2010, 2012, 2016, 2018, 2019 IQVIA. All rights reserved. The contents of this document are confidential and proprietary to IQVIA Holdings Inc. and its subsidiaries. Unauthorized use, disclosure or reproduction is strictly prohibited.

The efficacy of 2 IM doses of AZD1222 compared to saline placebo for the prevention of severe or critical symptomatic COVID-19	Adults ≥ 18 years of age who are healthy or have medically-stable chronic diseases and are at increased risk for SARS-CoV-2 acquisition and COVID-19, and who are seronegative at baseline.	a) 2 IM doses of AZD1222 or Placebo<br><br>b) At least one dose of AZD1222 or Placebo	a) A binary response, whereby a participant has at least one case of SARS-CoV-2 RT-PCR-positive severe or critical symptomatic illness occurring ≥ 15 days post second dose of study intervention.<br><br>b) A binary response, whereby a participant has at least one case of SARS-CoV-2 RT-PCR-positive severe or critical symptomatic illness occurring post first dose of study intervention.	For participants who withdraw from the study prior to having met the criteria for the endpoint, absence of data following these participants' withdrawal will be treated as missing (i.e. counted as not having met the criteria); Participants who withdraw before 15 days post second dose or who have a case prior to 15 days post second dose will be excluded from analysis (endpoint a only). Participants who (1) were unblinded to treatment assignment or (2) received COVID-19 licensed vaccine but were not unblinded to treatment assignment, in both cases prior to having met the criteria for the primary efficacy endpoint will be censored at the date of unblinding/ licensed COVID-19 vaccine administration, whichever is earlier.	Vaccine Efficacy (VE), calculated as 1-relative risk. (Relative risk is the incidence in the vaccine group relative to the incidence in the control group.)
The efficacy of 2 IM doses of	Adults ≥ 18 years of age who are healthy or have	2 IM doses of AZD1222	A binary response, whereby a participant has at least one	For participants who withdraw from the study prior to having met the criteria for	Vaccine Efficacy (VE), calculated as 1-relative

Copyright © 2009, 2010, 2012, 2016, 2018, 2019 IQVIA. All rights reserved. The contents of this document are confidential and proprietary to IQVIA Holdings Inc. and its subsidiaries. Unauthorized use, disclosure or reproduction is strictly prohibited.

	Attributes				
**Label**	**Definition Population**	**Treatment Condition of Interest**	**Variable/Endpoint**	**Intercurrent event handling strategy**	**Population-level summary measure**
AZD1222 compared to saline placebo for the prevention of COVID-19-related Emergency Department visits	medically-stable chronic diseases, and are at increased risk for SARS-CoV-2 acquisition and COVID-19, and who are seronegative at baseline.	or Placebo	case of COVID-19-related Emergency Department visit occurring ≥ 15 days post second dose of study intervention.	the endpoint, absence of data following these participants' withdrawal will be treated as missing (i.e. counted as not having met the criteria); Participants who withdraw before 15 days post second dose or who have a case prior to 15 days post second dose will be excluded from analysis. Participants who (1) were unblinded to treatment assignment or (2) received COVID-19 licensed vaccine but were not unblinded to treatment assignment, in both cases prior to having met the criteria for the primary efficacy endpoint will be censored at the date of unblinding/ licensed COVID-19 vaccine administration, whichever is earlier.	risk. (Relative risk is the incidence in the vaccine group relative to the incidence in the control group.)

Copyright © 2009, 2010, 2012, 2016, 2018, 2019 IQVIA. All rights reserved. The contents of this document are confidential and proprietary to IQVIA Holdings Inc. and its subsidiaries. Unauthorized use, disclosure or reproduction is strictly prohibited.

Antibody responses to AZD1222 S antigen following 2 IM doses of AZD1222 or saline placebo (Substudy and Illness Visits only)	Adults ≥ 18 years of age who are healthy or have medically-stable chronic diseases, and are at increased risk for SARS-CoV-2 acquisition and COVID-19, and who are seronegative at baseline and who don't have exclusionary important protocol deviations (IPDs).	2 IM doses of AZD1222 or Placebo	a) Post-treatment GMTs and GMFRs in SARS-CoV-2 S, RBD antibodies (MSD serology assay)<br><br>b) A binary response, whereby participants who have a post-treatment seroresponse (≥ 4-fold rise in titers from day of baseline value to 28 days post each dose) to the S, RBD antigens of AZD1222 (MSD serology assay)	For participants who withdraw from the study or who use restricted medications judged to have the potential to interfere with the generation or interpretation of an immune response, absence of data following these participants' withdrawal or subsequent data following the IPDs will be treated as missing. Participants who (1) were unblinded to treatment assignment or (2) received COVID-19 licensed vaccine but were not unblinded to treatment assignment, in both cases prior to having met the criteria for the primary efficacy endpoint will be censored at the date of unblinding/ licensed COVID-19 vaccine administration, whichever is earlier.	a) Post-treatment GMTs and GMFRs values from day of baseline value to 28 days post each dose in SARS-CoV-2 S, RBD antibodies<br><br>b) Number and percentage of participants who have a post-treatment seroresponse (≥ 4-fold rise in titers from day of baseline value to 28 days post each dose) to the S, RBD antigens of AZD1222
Anti-SARS-CoV-2 neutralizing antibody levels	Adults ≥ 18 years of age who are healthy or have medically-stable chronic diseases, and are at	2 IM doses of AZD1222 or Placebo	a) Post-treatment GMTs and GMFRs in SARS-CoV-2 neutralizing antibodies (wild-type assay or pseudo-	For participants who withdraw from the study or use restricted medications judged to have the potential to interfere with the generation or interpretation of an immune	a) Post-treatment GMTs and GMFRs values from day of baseline value to 28 days post each dose in

Copyright © 2009, 2010, 2012, 2016, 2018, 2019 IQVIA. All rights reserved. The contents of this document are confidential and proprietary to IQVIA Holdings Inc. and its subsidiaries. Unauthorized use, disclosure or reproduction is strictly prohibited.

Label	Attributes				
	**Definition Population**	**Treatment Condition of Interest**	**Variable/Endpoint**	**Intercurrent event handling strategy**	**Population-level summary measure**
in serum following 2 IM doses of AZD1222 or saline placebo (Substudy and Illness Visits only)	increased risk for SARS-CoV-2 acquisition and COVID-19, and who are seronegative at baseline, and who don't have exclusionary important protocol deviations (IPDs).		neutralization assay)<br><br>b) A binary response, whereby participants who have a post-treatment seroresponse (≥ 4-fold rise in titers from day of baseline value to 28 days post each dose) to AZD1222 as measured by SARS-CoV-2 neutralizing antibodies (wild-type assay or pseudo-neutralization assay)	response, absence of data following these participants' withdrawal or subsequent data following the IPDs will be treated as missing. Participants who (1) were unblinded to treatment assignment or (2) received COVID-19 licensed vaccine but were not unblinded to treatment assignment, in both cases prior to having met the criteria for the primary efficacy endpoint will be censored at the date of unblinding/ licensed COVID-19 vaccine administration, whichever is earlier.	SARS-CoV-2 neutralizing antibodies<br><br>b) Number and percentage of participants who have a post-treatment seroresponse (≥ 4-fold rise in titers from day of baseline value to 28 days post each dose) to AZD1222 as measured by SARS-CoV-2 neutralizing antibodies

Copyright © 2009, 2010, 2012, 2016, 2018, 2019 IQVIA. All rights reserved. The contents of this document are confidential and proprietary to IQVIA Holdings Inc. and its subsidiaries. Unauthorized use, disclosure or reproduction is strictly prohibited.

Label	Attributes				
	Definition Population	Treatment Condition of Interest	Variable/Endpoint	Intercurrent event handling strategy	Population-level summary measure
The efficacy of AZD1222 compared to saline placebo for the prevention of COVID-19 following the first dose	Adults ≥ 18 years of age who are healthy or have medically-stable chronic diseases and are at increased risk for SARS-CoV-2 acquisition and COVID-19, and who are seronegative at baseline	At least one IM dose of AZD1222 or Placebo	A binary response, whereby a participant is defined as a COVID-19 case if their first case of SARS-CoV-2 RT-PCR-positive symptomatic illness occurs post first dose of study intervention. Otherwise, a participant is not defined as a COVID-19 case.	For participants who withdraw from the study prior to having met the criteria for the primary efficacy endpoint, absence of data following these participants' withdrawal will be treated as missing (i.e. counted as not having met the criteria). Participants who (1) were unblinded to treatment assignment or (2) received COVID-19 licensed vaccine but were not unblinded to treatment assignment, in both cases prior to having met the criteria for the primary efficacy endpoint will be censored at the date of unblinding/ licensed COVID-19 vaccine administration, whichever is earlier.	Vaccine Efficacy (VE), calculated as 1-relative risk. (Relative risk is the incidence in the vaccine group relative to the incidence in the control group.)

Copyright © 2009, 2010, 2012, 2016, 2018, 2019 IQVIA. All rights reserved. The contents of this document are confidential and proprietary to IQVIA Holdings Inc. and its subsidiaries. Unauthorized use, disclosure or reproduction is strictly prohibited.

3. Study Design

3.1. General Description

D8110C00001 is a Phase III randomized, double-blind, placebo-controlled multicenter study assessing the safety, efficacy, and immunogenicity of AZD1222 compared to placebo for the prevention of COVID-19. Approximately 300 sites globally will participate in this study.

Participants will be adults ≥ 18 years of age who are healthy or have medically-stable chronic diseases and are at increased risk for SARS-CoV-2 acquisition and COVID-19. Approximately 30 000 participants will be randomized in a 2:1 ratio to receive 2 IM doses of either 5×10^{10} vp (nominal, $\pm 1.5 \times 10^{10}$ vp) AZD1222 (n = approximately 20 000) or saline placebo (n = approximately 10 000) 4 weeks apart, on Days 1 and 29. Randomization will be stratified by age (≥ 18 to < 65 years, and ≥ 65 years), with at least 25% of participants to be enrolled in the older age stratum. Participants who received their first dose of study intervention between 28 August 2020 and 06 September 2020 will receive their second dose of study intervention outside of the study window.

All participants will be assessed for efficacy and safety. The first participants randomized in each age group in the USA, including 1 500 participants 18 to 55 years of age, 750 participants 56 to 69 years of age, and 750 participants ≥ 70 years of age, will also participate in a substudy assessing the reactogenicity and immunogenicity of AZD1222. To further investigate cell-mediated immunogenicity, in particular Th1/Th2 responses, after AZD1222 or placebo administration, an immunogenicity cohort of approximately 300 participants will be added.

Participants will remain on study for 2 years following administration of the first dose of study intervention (Day 730). If AZD1222 is proven to be safe and efficacious based on the primary endpoint analysis, following discussion at that time with the US FDA, other Regulators if appropriate, and the COVID-19 Vaccine DSMB, participants allocated to the placebo group will be offered AZD1222 when doses are available. Placebo participants treated with AZD1222 will continue to be followed in the study.

Copyright © 2009, 2010, 2012, 2016, 2018, 2019 IQVIA. All rights reserved. The contents of this document are confidential and proprietary to IQVIA Holdings Inc. and its subsidiaries. Unauthorized use, disclosure or reproduction is strictly prohibited.

Table B: Study Design

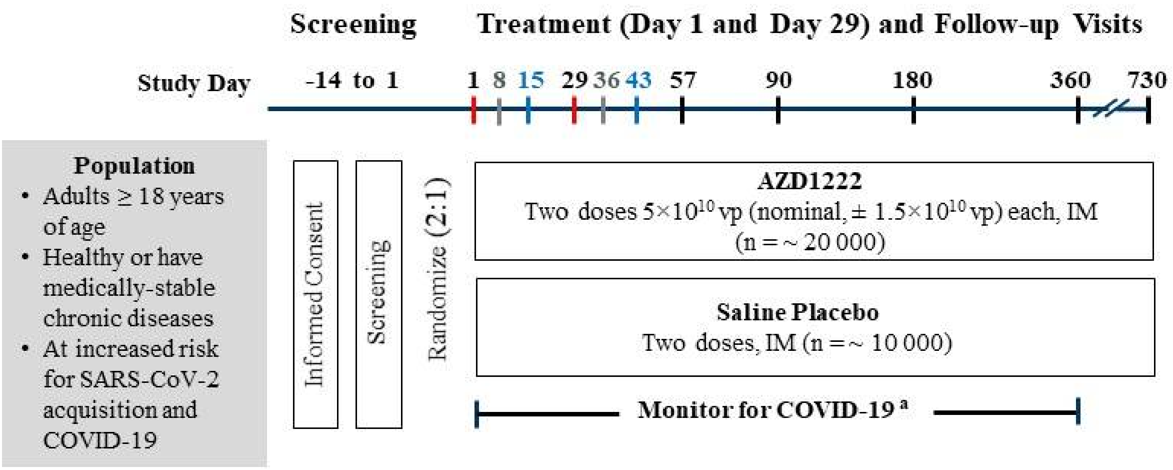

[a] Participants who present with qualifying symptoms will be tested for SARS-CoV-2 and if positive, will complete illness visits.

Red bars (Day 1 and Day 29): Administration of study intervention.

Gray bars (Day 8 and Day 36): Visits will be telephone contacts, not study site visits.

Blue bars (Day 15 and Day 43): Visits will only be for participants in the substudy. The first participants randomized in each age group in the USA, including 1 500 participants 18 to 55 years of age, 750 participants 56 to 69 years of age, and 750 participants ≥ 70 years of age, will also participate in a substudy assessing the reactogenicity and immunogenicity of AZD1222.

COVID-19 = coronavirus disease 2019; IM = intramuscular; SARS-CoV-2 = severe acute respiratory syndrome-coronavirus-2; USA = United States of America; vp = viral particles.

3.2. Schedule of Events

Schedule of events can be found in Section 1.3 of the protocol.

3.3. Changes to Analyses from Protocol

There are no changes to the analyses planned in the protocol.

Copyright © 2009, 2010, 2012, 2016, 2018, 2019 IQVIA. All rights reserved. The contents of this document are confidential and proprietary to IQVIA Holdings Inc. and its subsidiaries. Unauthorized use, disclosure or reproduction is strictly prohibited.

4. Planned Analyses

In addition to the planned analyses described below, an analysis to evaluate immunological markers as correlates of risk among vaccinees and towards the development of surrogates of protection will be performed by Operation Warp Speed (OWS). This analysis and corresponding sampling plan will be described in a separate stand-alone statistical analysis plan.

4.1. Data Monitoring Committee

An independent COVID-19 Vaccine DSMB will provide oversight, to ensure safe and ethical conduct of the study. Full details of the COVID-19 Vaccine DSMB can be found in the COVID-19 Vaccine DSMB Charter. An independent Neurological AESI Expert Committee will be available to review and provide advice to the Protocol Safety Review Team (PSRT) and the COVID-19 Vaccine DSMB on request about the diagnosis and causality assessment of selected neurological AESIs occurring in the AZD1222 clinical development program.

The COVID19 Vaccine DSMB will facilitate the interim analysis for safety and efficacy and have the responsibility of evaluating cumulative safety and other clinical study data at regular intervals and making appropriate recommendations based on the available data. During the study, the benefit/risk assessment will be continuously monitored by the COVID-19 Vaccine DSMB to ensure that the balance remains favorable. For example, events of potential vaccine associated enhanced respiratory disease will be evaluated by periodic reviews of COVID-19 cases by the DSMB. Harm for severe COVID-19 cases is any vaccine efficacy (VE) ≤0 for which Fisher's exact test (1-sided) is statistically significant at the 5% level. This assessment will begin after 8 cases of severe COVID-19 have accrued in the study and will be performed in real time as events occur. Harm monitoring will include all COVID-19 cases and all severe COVID-19 cases from day 1 for participants in the full analysis set. Harm monitoring for overall COVID-19 cases will use the same boundary as severe COVID-19 cases (ie, VE ≤0 for which Fisher's exact test (1-sided) is statistically significant at the 5% level) but will be performed on a weekly basis.

Based on the output of harm monitoring reviews by the DSMB, the study could be paused for further evaluation of the potential signal. Full details of the COVID-19 Vaccine DSMB composition and operations can be found in the COVID-19 Vaccine DSMB Charter.

4.2. Interim Analysis (IA)

All planned analyses for the interim efficacy analysis will be conducted by an independent statistics group providing

Copyright © 2009, 2010, 2012, 2016, 2018, 2019 IQVIA. All rights reserved. The contents of this document are confidential and proprietary to IQVIA Holdings Inc. and its subsidiaries. Unauthorized use, disclosure or reproduction is strictly prohibited.

support to the DSMB and IA when approximately 75 events meet the primary endpoint definition (i.e., when approximately 50% of the total amount of statistical information is available) have been reported across the active and control groups within the population of participants who are seronegative at baseline. The interim analysis will be used to support early assessment of efficacy and cumulative safety based on the available data. Detail of this interim analysis will be described in the DSMB plan, but methodology will be consistent with that described in this document.

In the event that the DSMB determines that the efficacy and safety have been definitively established based on review of the interim efficacy analysis and the cumulative safety data, a decision may be made by the Oversight Group and Unblinded Review Committee (URC) to proceed with regulatory submission based on the IA data cut (details are provided in the URC charter). Personnel from the Sponsor and its representatives (including IQVIA) associated with the analyses and regulatory submission of interim results will be unblinded and the full set of analyses described in this SAP will be performed.

4.3. Primary Analysis

All planned analyses for the primary analysis are detailed in this SAP and will be performed by IQVIA Biostatistics following Sponsor authorization of this SAP, Sponsor authorization of the analysis sets, database lock (DBL), and general study unblinding.

The primary DBL will occur after approximately 150 events that meet the primary endpoint definition have been observed across the active and control groups within the population of participants who are seronegative at baseline. All personnel involved in the analyses of the study will remain blinded until the primary DBL and protocol deviations are identified, unless early evidence of efficacy is demonstrated during interim analysis (refer to Section 4.2 for details).

In the event that the results of the interim or primary analysis are statistically significant for the primary efficacy endpoint, an additional analysis will be performed once all participants have completed their first year of follow up.

4.4. Final Analysis

All planned analyses for the final analysis are detailed in this SAP and will be performed by IQVIA Biostatistics following Sponsor authorization of this SAP, Sponsor authorization of the analysis sets, and DBL. All participants in the study will be assessed for efficacy, immunogenicity and safety for 2 years following the first dose of study

Copyright © 2009, 2010, 2012, 2016, 2018, 2019 IQVIA. All rights reserved. The contents of this document are confidential and proprietary to IQVIA Holdings Inc. and its subsidiaries. Unauthorized use, disclosure or reproduction is strictly prohibited.

intervention (Day 730). The final DBL will occur when all participants have completed the study.

5. Analysis Sets

5.1. All Participants Analysis Set

The all participants analysis (PAS) set will contain all participants screened for the study. All participants analysis set is to be used for reporting disposition and screening failures.

All participants screened are those who provide informed consent.

One participant was discovered through monitoring activities to have enrolled into the study at two separate clinical sites under two subject identification numbers ([redacted] & [redacted]). This participant was randomized at both sites and received both doses of study intervention, for a total of four doses. This participant is also believed to have received at least one dose of study intervention for a separate sponsor study of a COVID-19 vaccine candidate. Details related to this issue are under investigation at the time of approval for this analysis plan. This participant will be included in the PAS but will be excluded from all other analysis sets, and thus excluded from all analyses. A separate listing of this participant's data will be prepared.

5.2. Full Analysis Set

The full analysis set (FAS) will contain all randomized participants who received at least one dose of study intervention, irrespective of their protocol adherence and continued participation in the study. Participants will be analyzed according to their randomized treatment irrespective of whether or not they have prematurely discontinued, according to the intent-to-treat principle. Participants who withdraw consent to participate in the study will be included up to the date of their study withdrawal.

For analyses and displays based on full analysis set, participants will be classified according to randomized treatment regardless of what treatment they actually received.

5.3. Fully Vaccinated Analysis Set

The fully vaccinated analysis set (FVS) will include all participants in the FAS who are seronegative at baseline, receive two doses of study intervention, and who remain on-study 15 days after their second dose without having

Copyright © 2009, 2010, 2012, 2016, 2018, 2019 IQVIA. All rights reserved. The contents of this document are confidential and proprietary to IQVIA Holdings Inc. and its subsidiaries. Unauthorized use, disclosure or reproduction is strictly prohibited.

had a prior SARS-CoV-2 RT-PCR-positive confirmed COVID-19 infection.

The FVS will be used for the primary endpoint analysis as well as for applicable secondary and exploratory endpoints.

For analyses and displays based on the FVS, participants will be classified according to randomized study intervention regardless of what study intervention they actually received.

5.4. Per-protocol Analysis Set

The per-protocol analysis set (PPS) will include participants in the FVS who receive the correct dose of randomized study intervention and who do not have a serious protocol deviation.

Erroneously-treated participants who receive one dose of active study intervention and one dose of placebo, regardless of the sequence, will be excluded from this analysis set.

Exclusionary serious protocol deviations are those judged to potentially interfere with the generation of an immune response. Details will be defined in a separate protocol deviation (PD) plan (See Section 9.2).

5.5. Safety Analysis Set

The safety analysis set (SAF) consists of all participants who have received at least one dose of study intervention.

Erroneously-treated participants (e.g., participants who are given treatment different to their randomized treatment) are accounted for in this analysis set by assigning them to the treatment they actually received.

A participant who has on one or several occasions received active study intervention is classified as active for all summaries, including summaries by dose.

5.6. Immunogenicity Analysis Set

The immunogenicity analysis set (IAS) will include all participants in the SAF who have no protocol deviations judged to have the potential to interfere with the generation or interpretation of an immune response. Details of exclusionary protocol deviations will be defined in a separate PD plan (See Section 9.2).

Copyright © 2009, 2010, 2012, 2016, 2018, 2019 IQVIA. All rights reserved. The contents of this document are confidential and proprietary to IQVIA Holdings Inc. and its subsidiaries. Unauthorized use, disclosure or reproduction is strictly prohibited.

6. GENERAL CONSIDERATIONS

6.1. REFERENCE START DATE AND STUDY DAY

Study Day will be calculated from the reference start date and will be used to show start/stop day of assessments and events. Reference start date is defined as the day of the first dose of study intervention i.e., Day 1.

Study Day will be computed as follows:

- Study Day = (Date of event – Date of first dose of study intervention) + 1 if the date of the event is on or after the date of the first dose of study intervention;
- Study Day = (Date of event – Date of first dose of study intervention) if the date of the event is prior to the date of the first dose of study intervention.

In addition, day relative to vaccination will be derived for each vaccination dose. For example, day relative to the first dose will be equal to the Study Day. Day relative to the second dose will start with a value of 1 on the day of the second dose.

In the situation where the event date is partial or missing, Study Day and any corresponding durations will be displayed as missing in the listings.

6.2. BASELINE

Baseline is defined as the last non-missing measurement taken prior to the first dose of study intervention (including unscheduled assessments). In the case where the last non-missing measurement and the date and time of the first dose of study intervention coincide, that measurement will be considered to be baseline, but adverse events (AEs) and medications commencing on the date of the first dose of study intervention will be considered post-baseline.

Illness visit baseline is defined as the first non-missing measurement taken on illness visit day 1. If there is no non-missing measurement available on illness visit day 1, illness visit baseline is considered as missing.

Copyright © 2009, 2010, 2012, 2016, 2018, 2019 IQVIA. All rights reserved. The contents of this document are confidential and proprietary to IQVIA Holdings Inc. and its subsidiaries. Unauthorized use, disclosure or reproduction is strictly prohibited.

6.3. Unscheduled Visits, Retests, and Early Termination Data

For by-visit summaries, except for immunogenicity, data recorded at the nominal visit will be presented. That is, unscheduled, retest (same visit number assigned), and early termination measurements will not be included in by-visit summaries but might contribute to the baseline timepoint and/or maximum value, where required. Visits for immunogenicity data will follow a windowing convention as described in Section 6.4.

Listings will include scheduled, unscheduled, retest and early discontinuation data.

6.4. Windowing Conventions

A windowing convention will be used to determine the analysis value for a given study visit for immunogenicity data analyses. The window definitions as following will be used for the immunogenicity (serology and cell-mediated immunity (CMI)). The window conventions are:

1. A window of + /- 7 days from the target day is applied to the following visits: Study Days 15, 29, 43 and 57;

2. A window of +/- 28 days from the target day is applied to the following visits: Study Days 180, 360 and 730;

3. For illness visits, a window of + /- 7 days is applied to the following visits: illness visit Days 14 and 28.

Table C: Analysis windows for Immunogenicity by Visit (Main Study and Substudy)

Dosing Period	Visit	Day Relative to Dose within the Dosing Period [(b)]	Visit Window (Study Day) Relative to the Dosing Period
**Period 1 (Relative to Dose 1)**	Baseline [(a)]	≤ 1	≤ 1
	Day 15	15	8 - 21
	Day 29	29	22 - 35
**Period 2 (Relative to Dose 2)**	Day 43	15	8 - 21
	Day 57	29	22 - 35
	Day 180	152	124 - 180
	Day 360	332	304 - 360
	Day 730	702	674 - 730

(a) Where time is available, the time of the collection must be prior to the first dose of study intervention. Day 1 observations taken after the first dose are considered post-baseline values.

Copyright © 2009, 2010, 2012, 2016, 2018, 2019 IQVIA. All rights reserved. The contents of this document are confidential and proprietary to IQVIA Holdings Inc. and its subsidiaries. Unauthorized use, disclosure or reproduction is strictly prohibited.

(b) For each dosing period, the administration of the study intervention is designated as Study Day 1. For analyses within a period, the study day value is incremented by 1 for each date following the vaccine administration. Dates prior to the vaccine administration are decremented by 1, with the date preceding the vaccine administration designated as Study Day -1 (there is no Study Day 0).

Table D: Analysis windows for Immunogenicity by Illness Visit

Visit	Scheduled Illness Visit	Visit Window (Illness Day)
**Baseline**	1	1
**Illness Day 14**	14	8 - 21
**Illness Day 28**	28	22 - 35

One or more results for a particular immunogenicity variable may be obtained in the same visit window. In such an event, the result with the date closest to the expected visit date will be used in the analysis. In the event that two observations are equidistant from the expected visit date, the later observation will be used in the analysis.

6.5. Common Calculations

Change from baseline will be calculated as:

- Change from baseline = Test value at post baseline visit – baseline value

Percent change from baseline will be calculated as:

- Percent change from baseline (%) = (Change from baseline at post baseline visit / Baseline value) * 100%

Change from illness visit baseline will be calculated as:

- Change from illness visit baseline = Test value at post baseline illness visit – Illness visit baseline value

If baseline is not available, the change from baseline and percent change from baseline will not be calculated and will remain missing.

Copyright © 2009, 2010, 2012, 2016, 2018, 2019 IQVIA. All rights reserved. The contents of this document are confidential and proprietary to IQVIA Holdings Inc. and its subsidiaries. Unauthorized use, disclosure or reproduction is strictly prohibited.

7. Statistical Considerations

For continuous data, descriptive statistics (i.e., n [number of participants with available data], mean or geometric mean, standard deviation [SD] or geometric standard deviation [GSD], median, minimum and maximum, and quartiles values) will be presented by study arm and visit, when applicable.

For categorical data, the number and percentages of participants in each category will be presented by study arm and visit, when applicable. The denominator for percentage calculation is the underlying analysis set population unless otherwise stated.

The 2-sided p-value testing null hypothesis that the VE is equal to 30% will be presented for primary analysis of primary endpoint as well as supportive analysis of primary endpoint on PPS. All other tests regarding VE will present p-value testing null hypothesis that the VE is equal 0.

7.1. Sample Size Calculation

Approximately 33 000 participants will be screened such that approximately 30 000 participants will be randomized in a 2:1 ratio to receive 2 IM doses of either 5×10^{10} vp (nominal, $\pm 1.5 \times 10^{10}$ vp) AZD1222 (the active group, n = approximately 20 000) or saline placebo (the control group, n = approximately 10 000) 4 weeks apart.

Note: ‘Enrolled’ means a participant’s, or their legally acceptable representative’s, agreement to participate in a clinical study following completion of the informed consent process. Potential participants who are screened for the purpose of determining eligibility for the study, but are not randomly assigned in the study, are considered ‘screen failures,’ unless otherwise specified.

The sample size calculations are based on the primary efficacy endpoint and were derived following a modified Poisson regression approach (Zou, 2004). The calculations account for an interim and primary analysis, and the timing of primary analysis will be driven by the number of events observed in the study. The interim analysis will be carried out when approximately 50% of the total amount of statistical information is available. A Lan-DeMets alpha-spending function has been used to account for multiplicity, with 0.31% alpha at the interim analysis and 4.9% at the primary analysis such that the overall Type I error is controlled at 5%. The calculations assume minimal loss to follow-up as it is anticipated that participants will remain engaged in the study. All participants will be followed for the entire duration of the study.

For the primary efficacy analysis, approximately 150 events meeting the primary efficacy endpoint definition are

Copyright © 2009, 2010, 2012, 2016, 2018, 2019 IQVIA. All rights reserved. The contents of this document are confidential and proprietary to IQVIA Holdings Inc. and its subsidiaries. Unauthorized use, disclosure or reproduction is strictly prohibited.

required across the active and control groups within the population of participants who are seronegative at baseline to detect a VE of 60% with > 90% power. These calculations assume an observed attack rate of approximately 0.8% and are based on a 2-sided test, where the lower bound of the 2-sided 95.10% CI for VE is required to be greater than 30% with an observed point estimate of at least 50%.

An interim efficacy analysis will be conducted when approximately 75 events meeting the primary efficacy endpoint definition have been reported across the active and control groups within the population of participants who are seronegative at baseline, which will give > 70% power to detect a VE of 70% and > 90% power to detect a VE of 75%. These calculations assume an observed attack rate of approximately 0.4% and are based on a 2-sided test, where the lower bound of the 2-sided 99.69% CI for VE is required to be greater than 30% and an observed point estimate of at least 50%. A statistically significant finding at the interim analysis will not be considered a reason to stop the study, but instead will be interpreted as early assessment of efficacy.

7.2. MISSING DATA

Missing efficacy data will be handled as described in Sections 16.1.2, 16.2.2 of this analysis plan.

Partial or completely missing medication dates will be handled as described in APPENDIX 1.

7.3. STATISTICAL TESTS

The null hypothesis for the primary endpoint is: VE is equal to 30%. Whereas, the alternative hypothesis is: VE is not equal to 30%. That is:

- Null hypothesis: VE = 30%
- Alternative hypothesis: VE ≠ 30%

The primary efficacy endpoint will be formally assessed at 2 time points during the study, giving an interim analysis and a primary analysis. A Lan-DeMets alpha-spending function has been used to control the overall type I error at 5% with 0.31% alpha at the interim and 4.9% at the primary analysis. At the interim analysis the VE will be presented with a 2-sided 99.69% CI, and statistical significance will be achieved if the 2-sided 99.69% CI is > 30%. The success criterion for the interim analysis will be statistical significance with an observed VE point estimate of at least 50%. At the primary analysis VE will be presented with a 2-sided 95.10% CI, and statistical significance will be achieved if the 2-sided 95.10% CI is > 30%. The success criterion for the primary analysis of the study will be

Copyright © 2009, 2010, 2012, 2016, 2018, 2019 IQVIA. All rights reserved. The contents of this document are confidential and proprietary to IQVIA Holdings Inc. and its subsidiaries. Unauthorized use, disclosure or reproduction is strictly prohibited.

statistical significance with an observed VE point estimate of at least 50%.

In the event that the results of the interim or primary analysis are statistically significant for the primary efficacy endpoint, an additional analysis will be performed once all participants have completed their first year of follow up. For this analysis, VE will be presented with a 2-sided 95% CI, and statistical significance will be achieved if the 2-sided 95% CI is > 0%.

For the final analysis, a nominal type I error of 5% will be used for all analyses.

7.4. Multiple Comparisons/ Multiplicity

The primary efficacy endpoint and 4 key secondary endpoints will be assessed at 2 time points during the study, giving an interim analysis and a primary analysis. A Lan-DeMets alpha-spending function has been used to account for multiplicity of the primary endpoint across the 2 time points, with 0.31% alpha at the interim analysis and 4.9% at the primary analysis. Thus, the interim and primary analyses will present estimates with 2 sided 99.69 and 95.10% CIs, respectively, and statistical significance will be achieved if the 2-sided CIs are > 30%. At the interim or primary analysis, the success criterion for the study will be statistically significant with an observed VE point estimate of at least 50%. If the primary endpoint achieves statistical significance at the 0.31% level at the interim (or at the 4.9% level at the primary), a hierarchical approach will be used to control for multiplicity of the primary and key secondary efficacy endpoints. That is, the null hypotheses for these efficacy endpoints will be tested in a hierarchical order, and the subsequent null hypothesis will be tested at a significance level of 0.31% or 4.9% (2-sided), at the interim and primary analysis, respectively, only if the prior null hypothesis is rejected..

A formal assessment of the key secondary efficacy endpoints at the interim or primary analysis will only be conducted if the statistical significance of the primary efficacy endpoint is demonstrated at 2-sided alpha of 0.31% at the interim or 4.9% at the primary analysis. With that, the overall Type I error is controlled at 0.05. Therefore, no further multiplicity adjustment is necessary.

The testing strategy at the interim analysis or primary analysis will be as follows:

- Step 1

    1. Interim analysis: Perform the test of primary endpoint with 0.31% alpha level. If the two-sided 99.69% CI is > 30% with VE point estimate ≥ 50%, then proceed to step 2. Otherwise no null hypothesis is rejected for the interim analysis.

Copyright © 2009, 2010, 2012, 2016, 2018, 2019 IQVIA. All rights reserved. The contents of this document are confidential and proprietary to IQVIA Holdings Inc. and its subsidiaries. Unauthorized use, disclosure or reproduction is strictly prohibited.

2. Primary analysis: Perform the test of primary endpoint with 4.9% alpha level. If the two-sided 95.10% CI is > 30% with VE point estimate ≥ 50%, then proceed to step 2. Otherwise no null hypothesis is rejected for the primary analysis.

- Step 2:

1. Interim analysis: Test the 4 key secondary endpoint at the significance level of 0.31% using hierarchical fixed-sequence testing in the order below. If the two-sided 99.69% CI is > 0%, then proceed to the next endpoint.

2. Primary analysis: Test the 4 key secondary endpoint at the significance level of 4.9% using hierarchical fixed-sequence testing in the order below. If the two-sided 95.10% CI is > 0%, then proceed to the next endpoint.

   - Key Secondary Endpoint 1: Incidence of the first case of SARS-CoV-2 RT-PCR-positive symptomatic illness occurring ≥ 15 days post second dose of study intervention regardless of evidence of prior SARS-CoV-2 infection.

   - Key Secondary Endpoint 2: Incidence of the first case of SARS-CoV-2 RT-PCR-positive severe or critical symptomatic COVID-19 occurring ≥ 15 days post second dose of study intervention.

   - Key Secondary Endpoint 3: Incidence of COVID-19-related emergency department visits occurring ≥ 15 days post second dose of study intervention.

   - Key Secondary Endpoint 4: Incidence of the first post-treatment response (negative at baseline to positive post treatment with study intervention) for SARS-CoV-2 Nucleocapsid antibodies occurring ≥ 15 days post second dose of study intervention.

In the event that the results of the interim or primary analysis are statistically significant for the primary efficacy endpoint based on the above testing strategy, an additional analysis will be performed once all participants have completed their first year of follow up. This analysis will use the same testing strategy described above for primary and key secondary endpoints, with a 5% alpha level. Non-key secondary and exploratory endpoints will not be controlled for multiplicity. Thus, nominal 2-sided p-values will be presented to compare the vaccine against the control, alongside 2-sided 95% CIs.

Copyright © 2009, 2010, 2012, 2016, 2018, 2019 IQVIA. All rights reserved. The contents of this document are confidential and proprietary to IQVIA Holdings Inc. and its subsidiaries. Unauthorized use, disclosure or reproduction is strictly prohibited.

7.5. Multicenter Studies

This study will be conducted by multiple investigators at multiple centers internationally. Data from all centers will be pooled together in the analyses and there are no plans to perform an analysis of homogeneity of the results across centers.

7.6. Adjustments for Covariates and Factors to be Included in Analyses

The analyses will be adjusted for the following covariates and factors. For details of their inclusion in the models, refer the Sections 16.1.3, 16.2.3, and 16.3.1.

- Age group at informed consent (≥ 18 to < 65 years and ≥ 65 years) derived based on date of birth recorded in the interactive response technology (IRT) system.

7.7. Examination of Subgroups

Subgroup analyses will be conducted as stated in Sections 16.1.6.

The subgroups are:

- Age group at informed consent (≥ 18 to < 65 years and ≥ 65 years);
- Gender (male and female);
- Serostatus at baseline (negative and positive), where seropositive is defined by a positive Nucleocapsid antibody level as measured by Roche Elecsys Anti-SARS-CoV-2 serology test;
- Race (American Indian or Alaska Native, Asian, Black or African American, Native Hawaiian or Other Pacific Islanders, White);
- Ethnicity (Hispanic or Latino, Not Hispanic or Latino)
- Country
- COVID-19 Co-morbidities at baseline (at least one co-morbidity, no co-morbidity)

Copyright © 2009, 2010, 2012, 2016, 2018, 2019 IQVIA. All rights reserved. The contents of this document are confidential and proprietary to IQVIA Holdings Inc. and its subsidiaries. Unauthorized use, disclosure or reproduction is strictly prohibited.

If models of subgroup analysis do not converge due to sparse data, changes to planned subgroup analysis, including omission of the analysis for a subgroup or subgroup level where appropriate, will be described in the CSR.

7.8. Software Version

All analyses will be conducted using SAS version 9.4.

8. Output Presentations

APPENDIX 2 shows conventions for presentation of data in outputs.

The templates provided with this SAP describe the presentations for this study and therefore, the format and content of the summary tables, figures, and listings to be provided by IQVIA Biostatistics.

9. Disposition and Withdrawals

All participants who provide informed consent will be accounted for in this study.

9.1. Disposition

Number of participants screened will be presented overall for the PAS. Number and percentage of participants with screen failure and reason for screen failure will also be presented overall based on the PAS. Number of participants randomized will be presented overall and by study arm for the PAS. Number of participants randomized but not vaccinated will be presented overall and by study arm for the PAS as well.

Number and percentages of participants vaccinated for first dose, ongoing in study (for interim and/or primary analysis only) after first dose, who discontinued early from the study (including reason for withdrawal) before second dose and who discontinued study intervention (including reason for withdrawal) will be provided overall and by study arm based on the all randomized participants. Number and percentages of participants vaccinated for second dose, ongoing in study (for interim and/or primary analysis only) after second dose, remained in study for at least 15 days post second dose, and remained in study for at least 28 days post second dose will be provided overall and by study arm based on the all randomized participants. Additionally, the number of participants unblinded to treatment at the given timepoints and number of participants who received licensed COVID-19 vaccine but were not

Copyright © 2009, 2010, 2012, 2016, 2018, 2019 IQVIA. All rights reserved. The contents of this document are confidential and proprietary to IQVIA Holdings Inc. and its subsidiaries. Unauthorized use, disclosure or reproduction is strictly prohibited.

unblinded will be provided.

Additionally, the disposition summary table will be repeated respectively for participants who are seronegative at baseline and participants who are seropositive at baseline.

The number of participants included and excluded from each analysis set (including reason for exclusion) will be summarized overall and by study arm based on the all randomized participants. Number of participants included in the sub-study will be presented overall and by study arm.

A listing showing inclusion and exclusion of each participant from each analysis set, including reason for exclusion, will be provided.

The number and percentage of participants enrolled by country and site will be provided, including the dates when the first and last participants were enrolled at each site, overall and by study arm.

9.2. PROTOCOL DEVIATIONS

All important and exclusionary protocol deviations (separately for the per protocol and immunogenicity analysis sets) will be identified prior to study unblinding from review of study monitoring deviation logs and through programmatic review of blinded data, based on pre-specified criteria. Clinical judgment from the Sponsor will be necessary to classify each deviation as important or exclusionary; reviews will be performed on an ongoing basis throughout the study. Complete details of the pre-defined criteria and clinical review frequency will be documented in a separate PD plan.

The number and percentage of participants with important protocol deviations will be summarized overall and by study arm based on the all participants analysis set, overall and by protocol deviation category. The number and percentage of participants with exclusionary protocol deviations for the per-protocol and immunogenicity analysis sets will also be provided overall and by study arm. Protocol deviation categories are defined in the PD plan. A listing of important protocol deviations will also be provided. Non-important protocol deviations are documented in the clinical trial management system and will be filed in the study trial master file. However, they will not be included in summaries and listings within the scope of this analysis plan.

10. DEMOGRAPHIC AND OTHER BASELINE CHARACTERISTICS

The following demographic and other baseline characteristics will be reported for this study:

Copyright © 2009, 2010, 2012, 2016, 2018, 2019 IQVIA. All rights reserved. The contents of this document are confidential and proprietary to IQVIA Holdings Inc. and its subsidiaries. Unauthorized use, disclosure or reproduction is strictly prohibited.

- Age (years) – calculated relative to date of consent
- Age groups (refer to Section 7.7)
- Sex
- Race
- Ethnicity
- Weight (kg)
- Height (cm)
- Body Mass Index (BMI) (kg/m^2)
- Country
- Subgroups specified in Section 7.7

Continuous demographic and other baseline characteristics will be summarized using descriptive statistics overall and by study arm based on the SAF. For categorical demographic and other baseline characteristics, number and percentage of participants in each category will be provided overall and by study arm based on the SAF. No statistical testing will be carried out for demographic or other baseline characteristics.

Additionally, the demographic and other baseline characteristics summary will be repeated by baseline serostatus.

The demographic and other baseline characteristics summary will also be repeated on the FVS, IAS, SAF for the substudy and IAS for the substudy.

10.1. Derivations

BMI, in kg/m^2, will be calculated as follows:

- BMI (kg/ m^2) = weight (kg)/ [height (m)^2]

11. Medical History

Medical histories are defined as any medical conditions that happened before the first dose of study intervention and

Copyright © 2009, 2010, 2012, 2016, 2018, 2019 IQVIA. All rights reserved. The contents of this document are confidential and proprietary to IQVIA Holdings Inc. and its subsidiaries. Unauthorized use, disclosure or reproduction is strictly prohibited.

any medical conditions/diseases that started and stopped before the first dose of study intervention.

Medical history will be coded using the Medical Dictionary for Regulatory Activities (MedDRA), version 23.0 or higher, and will be summarized by System Organ Class (SOC) and Preferred Term (PT) overall and by study arm based on the SAF. A participant having more than one surgery/medical condition/disease within the same SOC/PT will be counted only once for that SOC or PT. All medical history will be listed.

Baseline co-morbidities solicited on the eCRF page will be summarized separately from medical histories above overall and by study arm based on the SAF and repeated for the FVS. It will also be summarized by serostatus at baseline.

12. CONCOMITANT ILLNESSES

Concomitant conditions/illnesses are defined as any medical conditions/illnesses that started before the first dose of study intervention AND were ongoing at the time of the first dose of study intervention or ended on the first dose day of study intervention.

Concomitant conditions/illnesses will be coded using the MedDRA, version 23.0 or higher, and will be summarized by SOC and PT overall and by study arm based on the SAF. A participant having more than one medical condition/illness within the same SOC or PT will be counted only once for that SOC or PT.

All concomitant conditions/illnesses will be listed.

13. MEDICATIONS

Prior medications are defined as any medication that started and stopped prior to the first dose of study intervention.

Concomitant medications are defined as:

- Any medication that started before the first dose of study intervention AND was ongoing at the time of the first dose of study intervention or ended on the date of first dose of study intervention;
- Any medication that started on or after the first dose of study intervention.

Partially or completely missing medication start and stop dates will be handled as described in APPENDIX 1.

All medications will be coded using the World Health Organization (WHO) Drug Global dictionary, version B3

Copyright © 2009, 2010, 2012, 2016, 2018, 2019 IQVIA. All rights reserved. The contents of this document are confidential and proprietary to IQVIA Holdings Inc. and its subsidiaries. Unauthorized use, disclosure or reproduction is strictly prohibited.

March 2020 or more recent version.

Prior and concomitant medications will be summarized by Anatomical Therapeutic Class (ATC) level 2 and preferred drug name overall and by study arm based on the SAF. A participant having more than one medication within the same ATC Level 2 or preferred drug name will be counted only once for that ATC Level 2 or preferred drug name.

All collected prior, concomitant medications will be listed.

14. Exposure to Study Intervention

Due to the simplicity of dosing for this study, exposure is summarized in the participant disposition table. No other summary will be reported.

15. Compliance with Study Intervention

Compliance will not be calculated since participants are vaccinated within clinic.

A summary of the interval between dose 1 and dose 2 of study intervention will be provided overall, and separated for participants randomized prior to the clinical hold and participants randomized after the clinical hold. The dosing interval is calculated as the date of dose 2 – date of dose 1 +1 for all participants who received two doses of study intervention. This analysis will be based on the SAF as well as the IAS for the substudy. The summary based on IAS for the study will also be repeated by age group (18-55 years, 56-69 years, and ≥70 years).

16. Efficacy Endpoints

Unless otherwise indicated, all efficacy summaries and figures will be presented based on the FVS.

All data from participants with SARS-CoV-2 RT-PCR-positive results will be assessed by a blinded independent efficacy endpoint adjudication committee, to classify each participant for inclusion in the primary and secondary COVID-19-related outcomes. Additional details are provided in the Efficacy Adjudication Committee Charter.

The central laboratory test result will be used for the determination of a SARS-CoV-2 RT-PCR-positive result. In case the central laboratory result is not available, then the local laboratory test result will be used. In the event that the results of the local and central laboratory are discordant (i.e. local result is positive and central result is negative

Copyright © 2009, 2010, 2012, 2016, 2018, 2019 IQVIA. All rights reserved. The contents of this document are confidential and proprietary to IQVIA Holdings Inc. and its subsidiaries. Unauthorized use, disclosure or reproduction is strictly prohibited.

or unknown due to insufficient quantity), the result of the saliva SARS-CoV-2 RT-PCR collected from the illness visit day 1 will be considered.

For all efficacy endpoints which are derived as incidences from binary outcomes, participants who (1) were unblinded to treatment assignment and (2) received licensed COVID-19 vaccine but were not unblinded to treatment assignment, in both cases prior to having met the criteria for the efficacy endpoint will not be counted as having the event. Their follow up time will be censored at the date of unblinding or licensed COVID-19 vaccine administration, whichever is earlier. For all secondary and exploratory endpoints summarized by visit, participants will be excluded for all visits after the date of unblinding/licensed COVID-19 vaccine administration. All data regardless of unblinding/licensed COVID-19 vaccine administration will be listed for all participants, with unblinded/post licensed COVID-19 vaccine administration assessments flagged.

An overall summary of number and percentage of participants who are SARS-CoV-2 RT-PCR-positive, number and percentage of participants who had illness visits overall and by SARS-CoV-2 RT-PCR status, as well as the number of illness visits will be provided by study arm for the FAS and FVS. Number and percentage of participants with secondary and exploratory endpoints for Covid-19 illness will also be included.

Summary statistics for the duration of follow up time from first dose, and from second dose, as well as the duration of follow up time from 15 days post second dose by study arm will be provided for the FAS and FVS.

An overall summary of the primary and key secondary endpoints analysis will be provided on FVS regardless of baseline serostatus. Number and percentage of events, Vaccine Efficacy and corresponding CIs as well as p-value from the model will be presented.

16.1. PRIMARY EFFICACY

16.1.1. PRIMARY EFFICACY ENDPOINT

The primary endpoint is the first case of SARS-CoV-2 RT-PCR-positive symptomatic illness occurring ≥ 15 days post second dose of study intervention, in a participant with negative serostatus at baseline. Participants will be included in the primary endpoint if they have RT-PCR-confirmed SARS-CoV-2 and meet the following criteria at any point from their initial illness visit at the site (Day 1) through their second illness visit (Day 14):

1. One or more Category A findings

OR

Copyright © 2009, 2010, 2012, 2016, 2018, 2019 IQVIA. All rights reserved. The contents of this document are confidential and proprietary to IQVIA Holdings Inc. and its subsidiaries. Unauthorized use, disclosure or reproduction is strictly prohibited.

2. Two or more Category B findings

Category A:

- Pneumonia diagnosed by chest x-ray, or computed tomography scan
- Oxygen saturation of ≤ 94% on room air or requiring either new initiation or escalation in supplemental O_2
- New or worsening dyspnea/shortness of breath

Category B:

- Fever > 100 ˚F (> 37.8 ˚C) or feverishness
- New or worsening cough
- Myalgia/muscle pain
- Fatigue that interferes with activities of daily living
- Vomiting and/or diarrhea (only one finding to be counted toward endpoint definition)
- Anosmia and/or ageusia (only one finding to be counted toward endpoint definition)

For participants with negative serostatus at baseline who died ≥ 15 days after second dose, if there was a SARS-CoV-2 RT-PCR-positive result from central lab before death, the participant would be considered as having met the primary endpoint; if there was not a SARS-CoV-2 RT-PCR-positive result from central lab before death, but COVID19 symptoms identified and the participant had a SARS-CoV-2 RT-PCR-positive result from any lab, the participant would be considered as having met the primary endpoint as well.

16.1.2. Missing Data Imputation Method for Primary Efficacy Endpoint

No missing data imputation method will be used for primary efficacy analysis. For participants who withdraw from the study prior to having met the criteria for the primary efficacy endpoint, absence of data following these participants' withdrawal (or lost to follow-up, death not caused by SARS-CoV-2) will be treated as missing.

Copyright © 2009, 2010, 2012, 2016, 2018, 2019 IQVIA. All rights reserved. The contents of this document are confidential and proprietary to IQVIA Holdings Inc. and its subsidiaries. Unauthorized use, disclosure or reproduction is strictly prohibited.

16.1.3. PRIMARY ANALYSIS OF PRIMARY EFFICACY ENDPOINT

The primary efficacy analysis of the primary endpoint will be performed on the FVS population. For participants who withdraw from the study prior to having met the criteria for the primary efficacy endpoint, absence of data following these participants' withdrawal (or lost to follow-up, death not caused by SARS-CoV-2) will be treated as missing.

A Poisson regression model with robust variance (Zou, 2004) adjusting for follow-up time, will be used as the primary efficacy analysis model to estimate the relative risk (RR) on the incidence of SARS-CoV-2 RT-PCR-positive symptomatic illness occurring ≥ 15 days post second dose of study intervention between the AZD1222 and the placebo groups. The model contains the term of study arm and age group at the time of informed consent (i.e., ≥ 18 to < 65 years, and ≥ 65 years) as covariates. The logarithm of the participant's corresponding monitoring period at risk starting from 15 days post second dose of study intervention up to the study day 360 visit will be used as an offset variable in the model to adjust for participants having different exposure times during which the events occur. Participants who withdraw or have a non-COVID-19 related death prior to having met the criteria for the primary efficacy endpoint will not be counted as having the event and the follow up time for these participants will be at that time from 15 days post second dose. Participants who (1) were unblinded to treatment assignment and (2) received licensed COVID-19 vaccine but were not unblinded to treatment assignment, in both cases prior to having met the criteria for the efficacy endpoint will not be counted as having the event. Their follow up time will be censored at the date of unblinding/licensed COVID-19 vaccine administration, whichever is earlier. Calculation of follow-up time is detailed as follows:

- For participants who meet the primary endpoint before the end of monitoring period, the follow up time will be calculated as (Date of Onset of Primary Endpoint) – (Date of Second Dosing+15) + 1. Date of Onset of Primary Endpoint is defined as the collection date of central lab positive COVID-19 test, or local lab if central is not available.
- For participants who do not experience a primary endpoint event before the end of monitoring period, the efficacy follow-up time will be determined based on the following:
    - If a SARS-CoV-2 RT-PCR positive symptomatic or asymptomatic event not meeting the primary endpoint criteria occurs during the COVID-19 monitoring period, the efficacy follow-up time will be calculated as (Date of positive COVID-19 test) – (Date of Second Dosing+15) + 1.
    - If an end of study date occurs during the COVID-19 monitoring period, the efficacy follow-up time will be calculated as (Date of End of Study) – (Date of Second Dosing+15) + 1.

Copyright © 2009, 2010, 2012, 2016, 2018, 2019 IQVIA. All rights reserved. The contents of this document are confidential and proprietary to IQVIA Holdings Inc. and its subsidiaries. Unauthorized use, disclosure or reproduction is strictly prohibited.

- If an end of study date occurs after the COVID-19 monitoring period, the efficacy follow-up will be censored at the end of COVID-19 monitoring period.

For participants who continue to participate in the study at the time of Interim or Primary Analysis, the data cut-off date will be used as their last assessment date. Vaccine efficacy (VE), which is the incidence in the vaccine group relative to the incidence in the control group expressed as a percentage, will be calculated as relative risk reduction (RRR) = 1- relative risk. RRR and its corresponding 2-sided 99.69 or 95.10% CI depending on if efficacy is declared at the interim or primary analysis, respectively (95% will be used for primary efficacy analysis at the time of final DBL), will be estimated from the Poisson regression model with robust variance. In addition, the 2-sided p-value testing null hypothesis that the VE is equal to 30% will be obtained from the model. Statistical significance will be achieved if the 95.10 or 99.69% CI for VE is > 30%. The success criterion for the study will be statistical significance with an observed VE point estimate of at least 50%.

The Poisson regression with robust variance analysis will be implemented by using the SAS PROC GENMOD procedure with the REPEATED statement for participant ID and logarithm link as well as OFFSET option. The estimated parameter $\hat{\beta}$ [i.e., $\log(\widehat{RR})$], 2-sided 99.69 or 95.10% (5% will be used for primary efficacy analysis at final DBL) confidence interval (CI) for $\hat{\beta}$, and the 2-sided p-value will be obtained from the SAS outputs. The estimated RR and corresponding CI for the RR is given by exponentiating $\hat{\beta}$ and its confidence limits. Therefore, the percent of RRR is given by [(1 – exp($\hat{\beta}$) * 100%]. The CI for the percent of RRR is given by ([1 – exp(upper confidence limit for $\hat{\beta}$) * 100%], [1 – exp(lower confidence limit for $\hat{\beta}$) * 100%]).

If the number of participants in any stratum is too small and/or convergence cannot be achieved with the Poisson regression analysis model, the model will be reduced to exclude the age group covariate. If convergence is not achieved by excluding the age group covariate, a stratified Exact Poisson Regression model will be used as the primary analysis model to test the treatment effect on SARS-CoV-2 RT-PCR-positive symptomatic illness between AZD1222 and placebo groups. The number of events for each combination of treatment and strata will be used as the response variable. The logarithm of total number of participants for each combination of treatments and strata will be used as an offset variable in the model. The Exact Poisson Regression test will be stratified by age group at the time of informed consent (i.e., ≥ 18 to < 65 years, and ≥ 65 years). SAS procedure of PROC GENMOD with EXACT statement will be used to perform the analysis. The RR of AZD1222 over placebo for the incidence of SARS-CoV-2 RT-PCR-positive symptomatic illness occurring ≥ 15 days post second dose of study intervention and the 99.69 or 95.10% CI will be obtained from the SAS procedure. The percent of RRR and the 99.69 or 95.10% CI will be reported following the relationship of RRR (%) = (1- RR) * 100%. In the event that active study arm has 0 events and Placebo has ≥ 1 event, the maximum likelihood estimate (MLE) for the RR is zero, corresponding to VE=100%, however PROC GENMOD gives a median unbiased estimate instead of the MLE and the upper

Copyright © 2009, 2010, 2012, 2016, 2018, 2019 IQVIA. All rights reserved. The contents of this document are confidential and proprietary to IQVIA Holdings Inc. and its subsidiaries. Unauthorized use, disclosure or reproduction is strictly prohibited.

confidence limit of VE cannot be estimated in this extreme situation. In such cases, the VE will be set to the MLE (100%) and the 1-sided (99.845% for interim, 97.55% for primary, and 97.5% for final analysis) confidence interval will be presented for completeness. The inverse will be treated similarly when there are 0 events in the placebo arm and ≥ 1 event in the active arm, such that the VE will be set to -Infinity and the one-sided confidence interval will be presented.

The number and percentages of participants with SARS-CoV-2 RT-PCR-positive symptomatic illness will also be presented for the following intervals: participants prior to 15 days post the first dose, between 15 days post the first dose and prior to the second dose, between the second dose and 15 days post the second dose, between 15 days post the second dose and prior to month 6, between month 6 and month 12, overall after first dose.

16.1.4. Sensitivity Analyses for Primary Efficacy Endpoint

As a sensitivity analysis to the handling of missing data in the analysis of the primary efficacy endpoint, the primary analysis of the primary efficacy endpoint (refer to Section 16.1.3) will be repeated with multiple imputation for intercurrent events, without using log follow-up time as offset. For participants who are in FVS but (1) do not have a SARS-CoV-2 RT-PCR-positive symptomatic illness status occurring ≥ 15 days post second dose of study intervention and withdraw from the study prior to the time of analysis, or (2) are in FVS and were unblinded to treatment assignment prior to having met the criteria for the efficacy endpoint or (3) received licensed COVID-19 vaccine but were not unblinded to treatment assignment prior to having met the criteria for the efficacy endpoint, their event status will be imputed assuming the observed placebo attack rate conditional on stratification factor using multiple imputation techniques as described in the following paragraphs.

The primary analysis uses Poisson regression with robust variance requires a participant-level dataset. A repeated imputation approach is introduced to impute the status of SARS-CoV-2 RT-PCR-positive symptomatic illness occurring ≥ 15 days post second dose of study intervention for missing observations at the participant-level for the model fitting. By incorporating the between-imputation variance, a reliable statistical inference in both hypothesis testing and CI estimation of the treatment effect is expected through the repeated imputation (Little and Rubin, 2002). In the primary analysis the missing outcome for participants who drop out (e.g., withdrawal, lost to follow-up, death not caused by SARS-CoV-2, etc) prior to reaching cut-off time for analysis without a SARS-CoV-2 RT-PCR-positive symptomatic illness occurring ≥ 15 days post second dose of study intervention will be imputed per stratum determined by the stratification factor using placebo event rate. The imputation and subsequent analysis will be carried out using SAS PROC MI (Monotone Logistic Regression Method) and SAS PROC MIANALYZE. The detailed imputation steps are described as follows.

Copyright © 2009, 2010, 2012, 2016, 2018, 2019 IQVIA. All rights reserved. The contents of this document are confidential and proprietary to IQVIA Holdings Inc. and its subsidiaries. Unauthorized use, disclosure or reproduction is strictly prohibited.

- Step 1: For the FVS participants in the AZD1222 arm who (1) do not have an SARS-CoV-2 RT-PCR-positive symptomatic illness and are not followed through cut-off time of analysis or (2) were unblinded to treatment assignment prior to having met the criteria for the efficacy endpoint or (3) received licensed COVID-19 vaccine but were not unblinded prior to having met the criteria for the efficacy endpoint, their treatment code of "AZD1222" will be substituted with "placebo" to ensure the placebo SARS-CoV-2 RT-PCR-positive symptomatic illness rate is applied in the imputation for the AZD1222 dropouts adjusted for their stratification values. The imputation will be executed using SAS Proc MI (e.g., logistic regression with the recoded treatment term and stratification factor). The random seed is 12345.

- Step 2: The original treatment code will be restored after the SARS-CoV-2 RT-PCR-positive symptomatic illness statuses have been imputed. A complete dataset comprises the imputed SARS-CoV-2 RT-PCR-positive symptomatic illness status and observed SARS-CoV-2 RT-PCR-positive symptomatic illness status.

- Step 3: Analyse the complete dataset using a Poisson regression model with robust variance to estimate the RR on the incidence of SARS-CoV-2 RT-PCR-positive symptomatic illness between AZD1222 and placebo, with the term of study arm and the stratification factor. The point estimate of log-transformed RR and its variance will be extracted from the model.

- Steps 2-3 will be repeated 20 times. SAS procedure PROC MIANALYZE will be used to combine inferences from the 20 completed datasets, that will result in a combined point estimate of log-transformed RR and the variance.

Additional sensitivity analyses will be carried out as given below

- Multiple imputation as described above using the observed event rate per treatment group for their event status

16.1.5. Supplementary Analyses for Primary Efficacy Endpoint

To support the primary analysis, a Cox Proportional Hazards model using the same covariates as for the primary analyses as well as Kaplan-Meier curves will be presented for the active and control groups based on observed events, showing the cumulative incidence of the first case of SARS-CoV-2 RT-PCR-positive symptomatic illness occurring ≥ 15 days post second dose of study intervention. Time to event, i.e., the duration in days since 15 days

Copyright © 2009, 2010, 2012, 2016, 2018, 2019 IQVIA. All rights reserved. The contents of this document are confidential and proprietary to IQVIA Holdings Inc. and its subsidiaries. Unauthorized use, disclosure or reproduction is strictly prohibited.

post second study dose to event or censoring, will be fit using the PH model with study arm as a factor and age group as stratum. Hazards ratios for each study arm along with the two-sided 95% CI will be obtained from the PH model. The number of participants with primary endpoint and the number of censored participants will also be provided. The censoring timing at each month will be displayed.

Another supplementary analysis is to break down the primary analysis described in Section 16.1.3 by repeating it for events occurring < 6 months from first dose of study intervention and ≥ 6 months from the first dose of study intervention up to the end of the COVID-19 monitoring period to show the efficacy over time.

The primary analysis described in Section 16.1.3 will also be repeated on PPS as a supplementary analysis. Same 2-sided p-value testing null hypothesis that the VE is equal to 30% will be present.

Additionally, the primary analysis will be repeated, excluding participants with out-of-window vaccination due to the clinical hold, i.e., participants who received their first dose of study intervention between 28 August 2020 and 06 September 2020.

The primary analysis will be repeated and will include participants in the FVS who (1) were unblinded to treatment assignment or (2) received licensed COVID-19 vaccine but were not unblinded, in both cases prior to having met the criteria for the efficacy endpoint. Censoring at study unblinding or licensed COVID-19 vaccine administration will not be performed.

16.1.6. Subgroup Analyses for Primary Efficacy Endpoint

Subgroup analysis will be performed for the primary efficacy endpoint, SARS-CoV-2 RT-PCR-positive symptomatic illness occurring ≥ 15 days post second dose of study intervention. For subgroup analysis, FVS set is used except for the subgroup analysis for serostatus at baseline which will use the FVS regardless of baseline serostatus. Treatment-by-subgroup interaction will be tested using the Poisson regression with robust variance model adjusting for follow-up time with the terms of treatment, age group, subgroup, and treatment-by-subgroup interaction, which will be implemented using PROC GENMOD procedure. If this full model does not achieve convergence, a reduced model of treatment, subgroup, and treatment-by-subgroup interaction will be used. Within each level of a subgroup, the RRR and its corresponding 95% CI will be estimated using a Poisson regression model with robust variance with the term of treatment, age group at informed consent and adjusting for follow-up time. A forest plot of the RRR and the 95% CI will be presented.

The subgroup analysis will be conducted for the subgroups in Section 7.7, provided there are sufficient events observed for each subgroup level. In the case of sparse data for one or more levels of a subgroup, alternative

Copyright © 2009, 2010, 2012, 2016, 2018, 2019 IQVIA. All rights reserved. The contents of this document are confidential and proprietary to IQVIA Holdings Inc. and its subsidiaries. Unauthorized use, disclosure or reproduction is strictly prohibited.

analysis approaches may be used, such as combining subgroup levels or fitting separate models for each subgroup, using the methods described in Section 16.1.3 for Exact Poisson Regression. Alternative approaches will be described in the study report.

For subgroups corresponding to one of the stratification factors included in the analysis model, the corresponding factor will not be included in the model.

16.2. Secondary Efficacy

The secondary efficacy endpoints which are derived from binary outcomes are described in following sections.

16.2.1. Secondary Efficacy Endpoints

16.2.1.1. The Incidence of the First Post-treatment Response (Negative at Baseline to Positive Post Treatment with Study Intervention) for SARS-CoV-2 Nucleocapsid Antibodies Occurring ≥ 15 Days Post Second Dose of Study Intervention

A key secondary endpoint, included in the multiplicity algorithm, is the incidence of the first post-treatment response (negative at baseline to positive post treatment with study intervention) for SARS-CoV-2 Nucleocapsid antibodies occurring ≥ 15 days post second dose of study intervention.

Blood samples will be collected as per the schedule of events (refer to protocol Section 1.3) for SARS-CoV-2 serology testing to monitor participants for infection.

16.2.1.2. The Incidence of the First Case of SARS-CoV-2 RT-PCR Positive Symptomatic Illness Occurring ≥ 15 Days Post Second Dose of Study Intervention Using CDC Criteria

A secondary endpoint is the incidence of the first case of SARS-CoV-2 RT-PCR-positive symptomatic illness occurring ≥ 15 days post second dose of study intervention using adapted CDC criteria (Appendix 6 in this SAP), with no minimum duration of symptoms required to satisfy the endpoint criteria.

Copyright © 2009, 2010, 2012, 2016, 2018, 2019 IQVIA. All rights reserved. The contents of this document are confidential and proprietary to IQVIA Holdings Inc. and its subsidiaries. Unauthorized use, disclosure or reproduction is strictly prohibited.

16.2.1.3. Incidence of the First Case of SARS-CoV-2 RT-PCR-positive Symptomatic Illness Occurring ≥ 15 Days Post Second Dose of Study Intervention Using University of Oxford Defined Symptom Criteria

A secondary endpoint is the Incidence of the first case of SARS-CoV-2 RT-PCR-positive symptomatic illness occurring ≥ 15 days post second dose of study intervention using University of Oxford defined symptom criteria: new onset of fever (> 100 °F [> 37.8 °C]), cough, shortness of breath, or anosmia/ageusia.

16.2.1.4. Incidence of the First Case of SARS-CoV-2 RT-PCR Positive Symptomatic Illness Occurring ≥ 15 Days Post Second Dose of Study Intervention Regardless of Evidence of Prior SARS-CoV-2 Infection

A key secondary endpoint, included in the multiplicity algorithm, is the incidence of the first case of SARS-CoV-2 RT-PCR-positive symptomatic illness occurring ≥ 15 days post second dose of study intervention regardless of evidence of prior SARS-CoV-2 infection.

16.2.1.5. Incidence of SARS-CoV-2 RT-PCR-positive Severe or Critical Symptomatic Illness Occurring ≥ 15 Days Post Second Dose of Study Intervention

A key secondary endpoint, included in the multiplicity algorithm, is the incidence of SARS-CoV-2 RT-PCR-positive severe or critical symptomatic illness occurring ≥ 15 days post second dose of study intervention. The severity of COVID-19 will be evaluated in participants with symptoms of COVID-19. A diagnosis of severe or critical COVID-19 will include laboratory-confirmed COVID-19 (SARS-CoV-2 RT-PCR-positive symptomatic illness) plus any of the findings presented as following. The calculation of the follow up time (included as offset in model) will be calculated by using the date symptoms become severe (as assessed by the endpoint adjudication committee) as the reference date.

Findings Regarding Severe/Critical Symptomatic COVID-19 are listed as below.

- Clinical signs at rest indicative of severe systemic illness (respiratory rate ⩾ 30 breaths per minute, heart rate ⩾ 125 beats per minute, oxygen saturation ⩽ 93% on room air at sea level, or partial pressure of oxygen to fraction of inspired oxygen ratio < 300 mm Hg)
- Respiratory failure (defined as needing high-flow oxygen, noninvasive ventilation, mechanical ventilation or extracorporeal membrane oxygenation)

Copyright © 2009, 2010, 2012, 2016, 2018, 2019 IQVIA. All rights reserved. The contents of this document are confidential and proprietary to IQVIA Holdings Inc. and its subsidiaries. Unauthorized use, disclosure or reproduction is strictly prohibited.

- Evidence of shock (systolic blood pressure < 90 mm Hg, diastolic blood pressure < 60 mm Hg, or requiring vasopressors)
- Significant acute renal, hepatic, or neurologic dysfunction
- Admission to an intensive care unit
- Death

The incidence of SARS-CoV-2 RT-PCR-positive severe or critical symptomatic illness occurring post first dose of study intervention will also be presented for participants in the FAS who are seronegative at baseline.

16.2.1.6. Incidence of COVID-19-related Emergency Department Visits Occurring ≥ 15 Days Post Second Dose of Study Intervention

A key secondary endpoint, included in the multiplicity algorithm, is the incidence of COVID-19-related emergency department visits occurring ≥ 15 days post second dose of study intervention. Adjudicated results as assessed by the endpoint adjudication committee will be used to identify COVID-19-related emergency department visits.

16.2.1.7. The Incidence of COVID-19 SARS-CoV-2 RT-PCR-positive Symptomatic Illness Occurring Post First Dose of Study Intervention

The incidence of COVID-19 SARS-CoV-2 RT-PCR-positive symptomatic illness occurring post first dose of study intervention will be analyzed in the same manner as the primary analysis of the primary efficacy endpoint (refer to Section 16.1.3) based on participants in FAS who were seronegative at baseline.

Kaplan-Meier curves will be presented for the active and control groups based on observed events, showing the cumulative incidence of the event. The Time to event is calculated as the duration in days since first study dose to event or censoring.

All incidence of COVID-19 SARS-CoV-2 RT-PCR-positive symptomatic illness occurring post first dose of study intervention will be listed.

16.2.2. Missing Data Imputation Method for Secondary Efficacy Endpoints

No imputation method will be used for secondary efficacy endpoints.

Copyright © 2009, 2010, 2012, 2016, 2018, 2019 IQVIA. All rights reserved. The contents of this document are confidential and proprietary to IQVIA Holdings Inc. and its subsidiaries. Unauthorized use, disclosure or reproduction is strictly prohibited.

16.2.3. Analysis of Secondary Efficacy Endpoints

Incidence of event secondary efficacy endpoints in sections above will be analyzed in the same manner as the primary analysis of the primary efficacy endpoint (refer to Section 16.1.3) using the FVS, except for the key secondary endpoint of the incidence of the first case of SARS-CoV-2 RT-PCR-positive symptomatic illness occurring ≥ 15 days post second dose of study intervention regardless of evidence of prior SARS-CoV-2 infection, which will be evaluated using the FVS regardless of prior SARS-CoV-2 infection, where infection refers to both RT-PCR and serology.

Follow-up time for secondary endpoints based on symptom criteria (CDC, University of Oxford, and severe or critical COVID-19, symptomatic COVID-19 post first dose) will be derived as described in section 16.1.3. Follow-up time for other COVID-19-related secondary and exploratory endpoints will be derived similarly, however no censoring will occur at a SARS-CoV-2 RT-PCR positive symptomatic or asymptomatic event not meeting the endpoint.

For incidence of event secondary efficacy endpoints in sections above, Kaplan-Meier curves will also be presented for the active and control groups based on observed events, showing the cumulative incidence of the event. The time to event is calculated as Section 16.1.5.

Additionally, the proportion of participants with post-treatment response (negative at baseline to positive post treatment with study intervention) for SARS-CoV-2 Nucleocapsid Antibodies will also be summarized based on FAS by study arm and visit, overall and by PCR result. The proportion of participants will be derived for the vaccine and control group, with corresponding 95% Clopper-Pearson exact CIs at each nominal timepoint in Protocol Section 1.3.

For key secondary endpoints based on FVS, a subgroup analysis by baseline serostatus will be performed using the same methods described for the primary efficacy analysis in Section 16.1.6.

16.3. Exploratory Efficacy

The exploratory efficacy endpoints are:

- The incidence of all-cause mortality from Day 1 through Day 730
- The incidence of COVID-19-related deaths occurring from Day 1 through Day 730

Copyright © 2009, 2010, 2012, 2016, 2018, 2019 IQVIA. All rights reserved. The contents of this document are confidential and proprietary to IQVIA Holdings Inc. and its subsidiaries. Unauthorized use, disclosure or reproduction is strictly prohibited.

- The incidence of COVID-19-related hospitalizations occurring ≥ 15 days post second dose of study intervention
- The incidence of COVID-19-related ICU admissions occurring ≥ 15 days post second dose of study intervention
- Viral genome copies in NP swabs collected at Illness Visits as determined by qRT-PCR (Illness Visits only)
- Genotypic analysis of SARS-CoV-2 from NP swabs collected on Day 1 illness visit (Illness Visits only)
- Duration of SARS-CoV-2 shedding in saliva over time (Illness Visits only)
- Biophysical parameters, including but not limited to serial measurements of skin temperature, heart rate, respiratory rate, blood oxygen saturation, and physical activity, recorded using a biosensor from illness visits Day 1 through Day 28 (Illness Visits only)
- Symptoms recorded by participants in an Illness e-Diary from illness visits Day 2 through Day 28 (Illness Visits only)

An overview of SARS-CoV-2 RT-PCR-positive symptomatic illness occurring ≥ 15 days post second dose will be provided and will include:

- Number of illness visits
- Number of SARS-CoV-2 RT-PCR-positive results for each visit

These summaries will be repeated for the following intervals: second dose date + 15 days to < 6 months from the first dose date and ≥ 6 months from first dose date to end of COVID-19 monitoring period.

16.3.1. Analysis of Exploratory Efficacy Endpoints

16.3.1.1. The Incidence of All-cause Mortality Occurring from Day 1 through Day 730

The incidence of all-cause mortality from Day 1 through Day 730 will be analyzed in the same manner as the primary analysis of the primary efficacy endpoint (refer to Section 16.1.3) based on participants in FAS who were seronegative at baseline. Follow-up time will be derived as described in section 16.2.3 for COVID-19-related

Copyright © 2009, 2010, 2012, 2016, 2018, 2019 IQVIA. All rights reserved. The contents of this document are confidential and proprietary to IQVIA Holdings Inc. and its subsidiaries. Unauthorized use, disclosure or reproduction is strictly prohibited.

secondary and exploratory events.

Kaplan-Meier curves will be presented for the active and control groups based on observed events, showing the cumulative incidence of the event. The time to event is calculated as Section 16.1.5.

All incidence of all-cause mortality from Day 1 through Day 730 will be listed.

16.3.1.2. The Incidence of COVID-19-related Deaths Occurring from Day 1 through Day 730

The incidence of COVID-19 related deaths occurring from Day 1 through Day 730 will be analyzed in the same manner as the primary analysis of the primary efficacy endpoint (refer to Section 16.1.3) based on participants in FAS who were seronegative at baseline. Follow-up time will be derived as described in section 16.2.3 for COVID-19-related secondary and exploratory events.

Kaplan-Meier curves will be presented for the active and control groups based on observed events, showing the cumulative incidence of the event. The time to event is calculated as Section 16.1.5.

All incidence of COVID-19 related deaths occurring from Day 1 through Day 730 will be listed.

16.3.1.3. The Incidence of COVID-19-related Hospitalizations Occurring ≥ 15 Days Post Second Dose of Study Intervention

The incidence of COVID-19-related hospitalizations occurring ≥ 15 days post second dose of study intervention will be analyzed in the same manner as the primary analysis of the primary efficacy endpoint (refer to Section 16.1.3) based on participants in FVS. Follow-up time will be derived as described in section 16.2.3 for COVID-19-related secondary and exploratory events.

Kaplan-Meier curves will be presented for the active and control groups based on observed events, showing the cumulative incidence of the event. The time to event is calculated as Section 16.1.5.

All incidence of COVID-19-related hospitalizations occurring ≥ 15 days post second dose of study intervention will be listed.

As a supportive analysis, the incidence of COVID-19-related hospitalizations occurring post first dose of study intervention will also be analyzed for participants in the FAS who are seronegative at baseline.

Copyright © 2009, 2010, 2012, 2016, 2018, 2019 IQVIA. All rights reserved. The contents of this document are confidential and proprietary to IQVIA Holdings Inc. and its subsidiaries. Unauthorized use, disclosure or reproduction is strictly prohibited.

16.3.1.4. The Incidence of COVID-19-related ICU Admissions Occurring ≥ 15 Days Post Second Dose of Study Intervention

The incidence of COVID-19-related ICU admissions occurring ≥ 15 days post second dose of study intervention will be analyzed in the same manner as the primary analysis of the primary efficacy endpoint (refer to Section 16.1.3) based on participants in FVS. Follow-up time will be derived as described in section 16.2.3 for COVID-19-related secondary and exploratory events.

Kaplan-Meier curves will be presented for the active and control groups based on observed events, showing the cumulative incidence of the event. The time to event is calculated as Section 16.1.5. All incidence of COVID-19-related ICU admissions occurring ≥ 15 days post second dose of study intervention will be listed.

All incidence of COVID-19-related ICU admissions occurring ≥ 15 days post second dose of study intervention will be listed.

As a supportive analysis, the incidence of COVID-19-related ICU admissions occurring post first dose of study intervention will also be analyzed for participants in the FAS who are seronegative at baseline.

16.3.1.5. Viral Genome Copies in NP Swabs Collected at Illness Visits as Determined by qRT-PCR

An exploratory efficacy endpoint is the Viral genome copies which will be collected via SARS-CoV-2 qRT-PCR test at central laboratory as scheduled in protocol section 1.3. Observed and change from illness visit baseline in viral load will be summarized by study arm and time points for the Illness Visits. If a participant has multiple sets of illness visits, the first set of illness visits with positive RT-PCR test result will be used for the summary. The analysis would be based on participants in IAS.

A listing will be provided for viral genome copies.

16.3.1.6. Genotypic Analysis of SARS-CoV-2 from NP Swabs Collected on Day 1 Illness visit

An exploratory efficacy endpoint is the Genotypic analysis of SARS-CoV-2 from NP swabs collected on illness visits. This analysis will be conducted by another party and will not be covered in this SAP.

Copyright © 2009, 2010, 2012, 2016, 2018, 2019 IQVIA. All rights reserved. The contents of this document are confidential and proprietary to IQVIA Holdings Inc. and its subsidiaries. Unauthorized use, disclosure or reproduction is strictly prohibited.

16.3.1.7. Duration of SARS-CoV-2 Shedding in Saliva over Time

- **Viral Shedding**

An exploratory efficacy endpoint is the duration of SARS-CoV-2 shedding in saliva over time. If a participant has multiple sets of illness visits, the first set of illness visits with positive RT-PCR test result will be used for the summary. Saliva samples for viral shedding will be collected at illness visits as indicated in Protocol section 1.3. The number and proportion of participants shedding on illness visit days 1, 3, 5, 8, 11, 14, 21 and 28 will be summarized. Exact 95% CIs will be computed. The analysis would be based on participants in IAS.

The duration of SARS-CoV-2 shedding in saliva will be calculated as following:

Duration (days) = (Date of illness visit when viral shedding first tested as persistently negative or date of last illness visit when test was positive, if no negative test available) – Date of first positive + 1. A cumulative incidence plot for the time to clearance for shedding will be provided for participants with positive SARS-CoV-2 in saliva.

The number of days of shedding will be summarized by descriptive statistics.

A listing will be provided for viral shedding.

- **Viral Quantitation**

For values reported as lower than the lower limit of quantitation (LLoQ), a value equal to half of the limit of quantification will be imputed in viral quantitation summaries. Missing values will not be imputed in viral quantitation summaries.

For the subset of participants in IAS who shed, viral quantities as measured by qRT-PCR will be summarized on illness visit days 1, 3, 5, 8, 11, 14, 21 and 28. Summary statistics will be presented describing the mean, standard deviation, median, minimum and maximum of Log_{10} (viral copies/mL) at baseline (Date of first positive) and each post-baseline time-points. Change from baseline at each post-baseline time point will also be summarized.

Time weighted change from baseline to each post-baseline time-point is derived on a by-participant basis using the linear trapezoidal rule with all available data from baseline to that specific time-point minus the baseline value. This is defined as (Area Under the Curve [AUC])/number of days – baseline value, between baseline to that specific post-baseline time-point. AUC from baseline to each post-baseline time-point will be reported as well.

Figures such as Log_{10} (viral copies/mL) over time (mean ± SD), AUC and time weighted change from baseline of Log_{10} (viral copies/mL) over time (box plots) will be provided.

Copyright © 2009, 2010, 2012, 2016, 2018, 2019 IQVIA. All rights reserved. The contents of this document are confidential and proprietary to IQVIA Holdings Inc. and its subsidiaries. Unauthorized use, disclosure or reproduction is strictly prohibited.

16.3.1.8. Biophysical Parameters, including but not limited to Serial Measurements of Skin Temperature, Heart rate, Respiratory Rate, Blood Oxygen Saturation, and Physical Activity, Recorded Using a Biosensor from Illness Visits Day 1 through Day 28

A group of efficacy endpoints are biophysical parameters collected from Current Health wearable device. The analysis of these exploratory results will be conducted by another party and are not covered by this SAP.

16.3.1.9. Symptoms Recorded by Participants in an Illness e-Diary from Illness Visits Day 2 through Day 28

A group of exploratory endpoints are symptoms collected by participants in an illness e-Diary. Symptoms from the first SARS-CoV-2 RT-PCR-positive symptomatic illness occurring ≥ 15 days post second dose will be summarized. If a participant has multiple sets of illness visits, the first set of illness visits with positive RT-PCR test result will be used for the summary. The number and percentage of participants with these symptoms, onset study day of these symptoms as well as the duration days will be summarized by study arm. Percentage is based on the number of participants with illness visits. The analysis would be based on participants in FVS.

All symptoms from each illness visit will be listed.

17. IMMUNOGENICITY ENDPOINTS

Unless otherwise indicated, all immunogenicity summaries will be presented by study arm and visit (visits from substudy and separately for visits from illness visits), when appropriate, based on the IAS.

All participants will be assessed for serum samples for SARS-COV-2 serology testing. The first participants randomized in each age group in the USA, including 1,500 participants 18 to 55 years of age, 750 participants 56 to 69 years of age, and 750 participants ≥ 70 years of age will also participate in a substudy assessing the immunogenicity of AZD1222.

For all immunogenicity endpoints, participants will be censored at the date of unblinding/licensed COVID-19 vaccine administration, whichever occurs first, such that data from all visits after the date of unblinding/licensed COVID-19 vaccine administration will be excluded from derivations and all by-visit summaries. All immunogenicity data regardless of unblinding/licensed COVID-19 vaccine administration will be listed for all participants, with unblinded/post licensed COVID-19 vaccine administration assessments flagged.

For summaries over time in participants from the substudy, participants who do not receive dose 2 of study

Copyright © 2009, 2010, 2012, 2016, 2018, 2019 IQVIA. All rights reserved. The contents of this document are confidential and proprietary to IQVIA Holdings Inc. and its subsidiaries. Unauthorized use, disclosure or reproduction is strictly prohibited.

intervention will be excluded from all time points post dose 2.

The immunogenicity secondary endpoints are:

- Post-treatment GMTs and GMFRs from day of dosing baseline value to 28 days post each dose in SARS-CoV-2 S, RBD antibodies (MSD serology assay) (Substudy and Illness Visits only)
- Proportion of participants who have a post-treatment seroresponse (≥ 4-fold rise in titers from day of dosing baseline value to 28 days post each dose) to the S, RBD antigens of AZD1222 (MSD serology assay) (Substudy and Illness Visits only)
- Post-treatment GMTs and GMFRs from day of dosing baseline value to 28 days post each dose in SARS-CoV-2 neutralizing antibodies (wild-type assay or pseudo-neutralization assay) (Substudy and Illness Visits only)
- Proportion of participants who have a post-treatment seroresponse (≥ 4-fold rise in titers from day of dosing baseline value (see Section 17.1.3) to 28 days post each dose) to AZD1222 as measured by SARS-CoV-2 neutralizing antibodies (wild-type assay or pseudo-neutralization assay) (Substudy and Illness visits only)

The exploratory immunogenicity endpoints are:

- Post-treatment GMTs and GMFRs from Day 1 baseline value to 28 days post each dose in ACE2 competing antibodies from serum samples (Substudy only)
- Proportion of participants who have a post-treatment seroresponse (≥ 4-fold rise in titers from day of dosing baseline value to 28 days post each dose) in ACE2 competing antibodies (Substudy only)
- Post-treatment GMTs and GMFRs from Day 1 baseline value to 28 days post each dose in SARS-CoV-2 S, RBD, and Nucleocapsid antibodies (MSD serology assay) (Substudy only, from participants enrolled in the immunogenicity cohort, from nasal secretions)
- Proportion of participants who have a post-treatment seroresponse (≥ 4-fold rise in titers from Day 1 baseline value to 28 days post each dose) to SARS-CoV-2 S, RBD, and Nucleocapsid antigens (MSD serology assay) (Substudy only, from participants enrolled in the immunogenicity cohort, from nasal secretions)
- Post-treatment GMTs and GMFRs from Day 1 baseline value to 28 days post each dose in ChAdOx1

Copyright © 2009, 2010, 2012, 2016, 2018, 2019 IQVIA. All rights reserved. The contents of this document are confidential and proprietary to IQVIA Holdings Inc. and its subsidiaries. Unauthorized use, disclosure or reproduction is strictly prohibited.

neutralizing antibodies (Substudy only)

- Proportion of participants who have a post-treatment seroresponse (≥ 4-fold rise in titers from Day 1 baseline value to 28 days post each dose) to AZD1222 as measured by ChAdOx1 neutralizing antibodies (Substudy only)

The exploratory cell-mediated immune (CMI) response endpoints are:

- Quantification of (IFN-γ) ELISpot responses to SARS-CoV-2 S protein from day of dosing baseline to 14 days post each dose (Substudy only, from participants in the immunogenicity cohort)
- Intracellular cytokine staining and flow cytometry for B- and T-cell responses from day of dosing baseline to 14 days post each dose (Substudy only, from participants in the immunogenicity cohort)

Other exploratory assays for humoral and cellular immune responses may be performed based upon emerging safety, efficacy, and immunogenicity data and will be described in a separate document.

17.1. Analysis of Immunogenicity Endpoints

Individual titer values for each endpoint, seroresponse and fold rise compared to baseline will be presented in a data listing.

17.1.1. Geometric Mean Titers and Geometric Mean Fold Rise

GMTs and GMFRs will be calculated for the vaccine and control groups and will be summarized at each scheduled visit as per protocol section 1.3 for the following titer measurements and participants:

- SARS-CoV-2 S, RDB antibodies from serum samples for participants in substudy and separately for participants positive for SARS-CoV-2
- SARS-CoV-2 neutralizing antibodies from serum samples for participants in substudy and separately for participants positive for SARS-CoV-2
- ACE2 competing antibodies from serum samples for participants in substudy
- SARS-CoV-2 S, RDB and Nucleocapsid antibodies from nasal samples by antibody isotype (IgG, IgA) for participants in substudy and enrolled in the immunogenicity cohort.

Copyright © 2009, 2010, 2012, 2016, 2018, 2019 IQVIA. All rights reserved. The contents of this document are confidential and proprietary to IQVIA Holdings Inc. and its subsidiaries. Unauthorized use, disclosure or reproduction is strictly prohibited.

Descriptive statistics for GMTs and GMFRs will include number of participants, geometric mean, 95% CI, minimum and maximum.

The GMT will be calculated as the antilogarithm of Σ(log base 2 transformed titer/n), i.e. as the antilogarithm transformation of the mean of the log-transformed titer, where n is the number of participants with titer information. The 95% CI will be calculated as the anti-logarithm transformation of the upper and lower limits for a two-sided CI for the mean of the log-transformed titers.

The fold rise is calculated as the ratio of the post-vaccination titer level to the pre-vaccination titer level. GMFR will be calculated as anti-logarithm of Σ (log base 2 transformed (post-vaccination titer/ pre-vaccination titer)/n). The 95% CIs for GMFR will be calculated similarly to those for GMT.

The GMT and GMFR endpoints will be analyzed using an analysis of variance (ANOVA) model which includes the log base 2-transformed value of titer (or log base 2-transformed value of fold rise for GMFR) as the dependent variable and study arm and age group as factors. On the log scale, the models will be used to estimate a mean response for the vaccine and control groups and the difference (vaccine - control), with corresponding 95% confidence limits. These values will then be back-transformed to give geometric means for the vaccine and control groups and a ratio of geometric means (vaccine/control), with corresponding 95% confidence limits. A p-value, corresponding to a 2-sided test, will be presented to compare the vaccine against the control. The p-value will be nominal as secondary endpoints are not controlled for multiplicity. This analysis will be performed on participants who are seronegative at baseline (i.e. participants having a titer value < LLoQ at baseline).

The above analyses for serum samples of SARS-CoV-2 S, RBD and Nucleocapsid antibodies as well as SARS-CoV-2 neutralizing antibodies will also be performed separately by baseline serostatus. Additionally, summaries for participants in the substudy will be presented by age group (18-64 years, ≥65 years) and by clinical hold status (randomized prior to clinical hold, randomized after clinical hold).

17.1.2. Seroresponse Rate

Seroresponse is a binary outcome where a success is when the fold rise in titers compared to baseline is ≥4. Seroresponse will be calculated for the vaccine and control groups and will be summarized at each scheduled visit as per protocol section 1.3 for the following titer measurements and participants:

- SARS-CoV-2 S, RDB antibodies from serum samples for participants in substudy and separately for participants positive for SARS-CoV-2

Copyright © 2009, 2010, 2012, 2016, 2018, 2019 IQVIA. All rights reserved. The contents of this document are confidential and proprietary to IQVIA Holdings Inc. and its subsidiaries. Unauthorized use, disclosure or reproduction is strictly prohibited.

- SARS-CoV-2 neutralizing antibodies from serum samples for participants in substudy and separately for participants positive for SARS-CoV-2
- ACE2 competing antibodies from serum samples for participants in substudy
- SARS-CoV-2 S, RDB and Nucleocapsid antibodies from nasal samples by antibody isotype (IgG, IgA) for participants in substudy and enrolled in the immunogenicity cohort
- ChAdOx1 neutralizing antibodies from serum samples for participants in substudy

The number and percentage of participants with post-vaccination seroresponse, and 95% CIs will be provided and the 95% CI of seroresponse rate will be calculated using the Clopper-Pearson exact method. These seroresponse summaries for serum samples of SARS-CoV-2 S, RBD and Nucleocapsid antibodies as well as SARS-CoV-2 neutralizing antibodies will also be performed separately by baseline serostatus. Additionally, summaries for participants in the substudy will be presented by age group (18-64 years, ≥65 years) and by clinical hold status (randomized prior to clinical hold, randomized after clinical hold).

17.1.3. Missing Data Imputation Method for Immunogenicity Endpoints

A titer value measured below the lower limit of quantification (LLoQ) will be imputed to a value that is half of the LLoQ in summaries and analyses, but will be listed as reported in the raw serology data. For example, a serologic assay with LLoQ = 30 generally reports values below LLoQ as "<30". The data listings will present the values as "<30", while values of 15 (i.e., 30/2) are to be used in the summaries and analyses.

Titer values measured as above ULoQ will be imputed at the ULoQ value.

17.2. Analysis of Cell-mediated Immune Response Endpoints

CMI responses (i.e., B-cell and T-cell responses) will be collected from up to 300 participants in the substudy, approximately 300 participants in the immunogenicity cohort and from up to 3 000 participants, where operationally feasible, during the Day 1 illness visit, and any participants with a Day 1 SARS-CoV-2 positive test result by local RT-PCR test. CMI data will be reported separately from CSR.

Copyright © 2009, 2010, 2012, 2016, 2018, 2019 IQVIA. All rights reserved. The contents of this document are confidential and proprietary to IQVIA Holdings Inc. and its subsidiaries. Unauthorized use, disclosure or reproduction is strictly prohibited.

18. Safety Endpoints

The safety of AZD1222 will primarily be assessed by:

- Incidence of AEs for 28 days post each dose of study intervention
- Incidence of SAEs from Day 1 post treatment through Day 730
- Incidence of MAAE (defined in Protocol Section 8.3.8) and AESIs (defined in Protocol Section 8.3.9) from Day 1 post treatment through Day 730
- Incidence of local and systemic solicited AEs for 7 days post each dose of study intervention (substudy only)

There are also other safety endpoints such as vital signs.

All safety summaries will be presented by study arm based on the SAF. There will be no statistical comparisons between the study arms for safety data.

For participants who are unblinded to treatment assignment during the study and received licensed COVID-19 vaccine but were not unblinded, the unblinding/licensed COVID-19 vaccine administration will be treated as an intercurrent event. Safety data collected during the unblinded/post licensed COVID-19 vaccine administration follow up period will be excluded from all summaries directly comparing the AZD1222 and placebo study arms, using exposure adjusted rates to account for differences in duration of double-blinded follow up for SAEs, MAAEs, and AESIs which are collected through the full 2-year study period. SAEs, MAAEs, and AESIs will also be summarized for the double-blinded period, unblinded period/post licensed COVID-19 vaccine period, and overall, separately for each study arm.

Summary statistics of follow up time, as well as number and percentage of participants at risk at each month post first study intervention will also be provided on SAF. This summary will include the follow-up time post first dose as well as the follow-up time post second dose.

18.1. Adverse Events

All AEs are considered to be unsolicited AEs (collected by ‘open question’ at study visits) unless categorized as solicited AEs recorded in the substudy only.

Copyright © 2009, 2010, 2012, 2016, 2018, 2019 IQVIA. All rights reserved. The contents of this document are confidential and proprietary to IQVIA Holdings Inc. and its subsidiaries. Unauthorized use, disclosure or reproduction is strictly prohibited.

Non-serious AEs will be recorded for 28 days post each dose of study intervention. SAEs, MAAEs, and AESIs will be recorded from the time of signature of the informed consent form through the last participant contact.

All AEs will be coded using the MedDRA dictionary, version 23.0 or higher.

Unless specified, event summary refers to the summary of number of participants with the corresponding adverse event.

All AE summary tables listed below will be repeated respectively for participants who are seronegative at baseline and participants who are seropositive at baseline.

Overall summaries of number and percentage of participants, and number of events with following AE categories will be provided by study arm based on the SAF:

- All AEs
- All SAEs
- Related AEs by severity
- Related SAEs
- AEs leading to discontinuation from study intervention
- Related AEs leading to discontinuation from study intervention
- AEs leading to study discontinuation
- Related AEs leading to study discontinuation
- MAAEs
- AEs with outcome of death
- AESIs

Should a participant experience multiple events within a category, the participant will be counted only once for that category.

An overall summary of number and percentage of participants, and number of events within each of the categories described above will be provided for the period from 1-28 days post any dose by study arm based on the SAF.

Copyright © 2009, 2010, 2012, 2016, 2018, 2019 IQVIA. All rights reserved. The contents of this document are confidential and proprietary to IQVIA Holdings Inc. and its subsidiaries. Unauthorized use, disclosure or reproduction is strictly prohibited.

An overall summary of number and percentage of participants, including exposure adjusted rates, and number of events within categories of all SAEs, related SAEs, related SAEs by severity, AEs leading to study discontinuation, related AEs leading to study discontinuation, MAAEs, AEs with outcome of death and AESIs during the entire period of study will be provided by study arm based on the SAF. Exposure adjusted rate is calculated as number of participants with AEs in categories above/total patient-year exposure to investigational study intervention. Patient years is determined by summing the total number of follow-up days of each participant, and then dividing by 365.25. The exposure period is calculated from time of first intervention to end of study.

18.1.1. Adverse Events

Adverse events post the first study intervention occurring within 28 days of each dose will be summarized by SOC and PT by study arm. Specific AEs will be counted once for each participant for calculating percentages.

Non-serious AEs reported by > 1% participants in any study arm, including exposure adjusted rates, will be summarized by PT. AEs reported by > 1% participants in any study arm within 28 days of each study intervention will also be summarized by PT.

Summary of AEs occurring within 28 days post each intervention will be broken down further by post each dose, maximum severity and events related to study intervention. If the same AE occurs multiple times within a particular participant, the highest severity observed will be reported.

Listings of AEs will be provided, with events occurring after unblinding/licensed COVID-19 vaccine administration flagged.

18.1.1.1. Severity for AEs

Severity will be classified as mild, moderate or severe (increasing severity) by using FDA Grading for AEs. Severity for AEs will be collected on AE form of eCRF. Should a participant experience multiple events within a SOC or PT, only the participant's worst severity FDA grade will be counted for that SOC or PT.

18.1.1.2. Relationship to Study Intervention/Non-Study Intervention/Procedure

Relationship to study intervention/procedure, as indicated by the Investigator, will be classified as not related, related.

Copyright © 2009, 2010, 2012, 2016, 2018, 2019 IQVIA. All rights reserved. The contents of this document are confidential and proprietary to IQVIA Holdings Inc. and its subsidiaries. Unauthorized use, disclosure or reproduction is strictly prohibited.

18.1.2. Serious Adverse Events

Serious adverse events (SAEs) are those events recorded as "Serious" on the AE page of the eCRF.

SAEs post the first study intervention, including exposure adjusted rates, will be summarized by SOC and PT by study arm. Specific SAEs will be counted once for each participant for calculating percentages.

Summary of SAEs post the first study intervention, including exposure adjusted rates, will also be broken down further by post each dose, maximum severity and events related to study intervention. If the same SAE occurs multiple times within a particular participant, the highest severity observed will be reported. A summary of related SAEs post the first study intervention, including exposure adjusted rates, will be presented by SOC and PT by study arm as well.

SAEs will also be summarized for the double-blinded period, unblinded period/post COVID-19 licensed vaccine administration, and overall, separately for each study arm. This summary will be repeated for related SAEs.

Listings of SAEs will be provided, with events occurring after unblinding/licensed COVID-19 vaccine administration flagged. SAEs prior to the first study intervention will only be presented in the listings. For SAEs with partial dates, if the known part of the date indicates that SAE stopped before the first study intervention, it will be considered as SAE prior to the first study intervention. Otherwise, it will be considered as SAE post the first study intervention.

18.1.3. AEs Leading to Discontinuation of Study Intervention

AEs leading to permanent discontinuation of study intervention are those participants recorded as not complete the full course of study intervention on the "Adverse Event" pages of the eCRF. AEs leading to permanent discontinuation of study intervention will be recorded within 28 days post first dose of study intervention. A summary of AEs leading to permanent discontinuation of study intervention by SOC and PT will be prepared.

A summary of related AEs leading to permanent discontinuation of study intervention by SOC and PT will be prepared.

A listing of all AEs leading to permanent discontinuation of study intervention will be provided, with events occurring after unblinding/licensed COVID-19 vaccine administration flagged.

Copyright © 2009, 2010, 2012, 2016, 2018, 2019 IQVIA. All rights reserved. The contents of this document are confidential and proprietary to IQVIA Holdings Inc. and its subsidiaries. Unauthorized use, disclosure or reproduction is strictly prohibited.

18.1.4. AEs Leading to Discontinuation of Study

AEs leading to permanent discontinuation of study are those participants recorded as not complete the study on the "Adverse Events" pages of the eCRF. Non-serious AEs leading to permanent discontinuation of study will be recorded within 28 days post each dose of study intervention. SAEs, MAAEs, and AESIs leading to permanent discontinuation of study will be recorded through to end of study. A summary of AEs within 28 days post each dose of study intervention leading to permanent discontinuation of study by SOC and PT will be prepared. A summary of SAEs, MAAEs, and AESIs during the entire period of study leading to permanent discontinuation of study by SOC and PT will be prepared, including exposure adjusted rates.

A summary of related AEs occurring within 28 days post each dose of study intervention leading to permanent discontinuation of study by SOC and PT will be prepared. A summary of related SAEs, MAAEs, and AESIs during the entire period of study leading to permanent discontinuation of study by SOC and PT will also be prepared, including exposure adjusted rates.

SAEs, MAAEs, and AESIs leading to permanent discontinuation of study will also be summarized for the double-blinded period, unblinded period/post licensed COVID-19 vaccine administration period, and overall, separately for each study arm. This summary will be repeated for related SAEs, MAAEs, and AESIs leading to permanent discontinuation of study.

A listing of all AEs leading to permanent discontinuation of study will be provided, with events occurring after unblinding/licensed COVID-19 vaccine administration flagged.

18.1.5. AEs with Outcome of Death

AEs with outcome of death are those participants recorded as having an AE with "Fatal" outcome on the "Adverse Event" pages of the eCRF. A summary of AEs with outcome of death by SOC and PT will be prepared, including exposure adjusted rates. A summary of related AEs with outcome of death by SOC and PT will also be prepared, including exposure adjusted rates.

AEs with outcome of death will also be summarized for the double-blinded period, unblinded period/post licensed COVID-19 vaccine administration period, and overall, separately for each study arm. This summary will be repeated for related AEs with outcome of death.

A listing of all AEs with outcome of death will be provided, with events occurring after unblinding/licensed COVID-19 vaccine administration flagged.

Copyright © 2009, 2010, 2012, 2016, 2018, 2019 IQVIA. All rights reserved. The contents of this document are confidential and proprietary to IQVIA Holdings Inc. and its subsidiaries. Unauthorized use, disclosure or reproduction is strictly prohibited.

18.1.6. Medically Attended Adverse Events

Medically attended adverse events (MAAEs) are AEs leading to medically-attended visits that were not routine visits for physical examination or vaccination, such as an emergency room visit, or an otherwise unscheduled visit to or from medical personnel (medical doctor) for any reason. AEs, including abnormal vital signs, identified on a routine study visit or during the scheduled illness visits will not be considered MAAEs. MAAEs will be recorded from Day 1, post first study intervention, through the last participant's contact.

A summary of MAAEs by SOC and PT by study arm will be prepared, including exposure adjusted rates. Should a participant experience multiple events within a SOC or PT, the participant will be counted only once for that SOC or PT.

MAAEs will also be summarized for the double-blinded period, unblinded period/post licensed COVID-19 vaccine administration, and overall, separately for each study arm.

A listing of all MAAEs will be provided, with events occurring after unblinding/licensed COVID-19 vaccine administration flagged.

18.1.7. Adverse Events of Special Interest

AEs of special interest (AESIs) are events of scientific and medical interest specific to the further understanding of study intervention safety profile and require close monitoring and rapid communication by the investigators to the Sponsor. AESIs for AZD1222 are based on Brighton Collaboration case definitions (SPEAC 2020), clinical experience, and scientific interest. See Appendix 7 for a listing and description of AZD1222 AESIs.

AESIs will be recorded from Day 1, post first study intervention, through the last participant's contact. A summary of AESIs by categories and sub-categories listed in Appendix 7 and PT and by study arm will be prepared, including exposure adjusted rates. The summary will also include the number and percentage of participants with any neurologic and/or neuroinflammatory AESIs. Should a participant experience multiple events within a category or PT, the participant will be counted only once for that category or PT. A summary of related AESIs by category and PT will also be prepared, including exposure adjusted rates. A summary of AESIs by severity grade (Grade 1, Grade 2 and Grade >=3) and a summary of related AESIs by severity grade will be prepared. AESIs will also be summarized for the double-blinded period, unblinded period/post licensed COVID-19 vaccine administration period, and overall, separately for each study arm. This summary will be repeated for related AESIs.

AESIs will be summarized by the subgroups of baseline serostatus, age group, sex, and race.

Copyright © 2009, 2010, 2012, 2016, 2018, 2019 IQVIA. All rights reserved. The contents of this document are confidential and proprietary to IQVIA Holdings Inc. and its subsidiaries. Unauthorized use, disclosure or reproduction is strictly prohibited.

A listing of all AESIs will be provided, with events occurring after unblinding/licensed COVID-19 vaccine administration flagged.

18.1.8. Additional Summaries of Adverse events

A summary of adverse events in the SOC of Nervous System Disorders by PT and study arm will be prepared, including exposure adjusted rates. Should a participant experience multiple events within a PT, the participant will be counted only once for that PT. AEs in the SOC of Nervous System Disorders will also be summarized by baseline serostatus, age group, sex, and race. These events will also be summarized for the double-blinded period, unblinded period/post licensed COVID-19 vaccine administration, and overall, separately for each study arm.

The above summaries will be repeated for the following:

- Vascular Disorders of Embolism and Thrombosis.
- Potentially Immune-Mediated Conditions (see Appendix 7)
- Standard MedDRA Queries (SMQs):
    - Demyelination
    - Peripheral neuropathy
    - Guillain-Barre Syndrome
    - Embolic and thrombotic events, vessel type unspecified and mixed arterial and venous
    - Immune mediated/autoimmune disorders
    - Anaphylactic reaction
    - Hypersensitivity

18.2. Solicited Adverse Events (Only for Substudy)

For participants in the substudy, safety will be assessed daily for 7 days after vaccination (symptoms reported in the last 24 hours are collected on Days 2-8) via e-diary collection of solicited AEs. These include local events (pain, tenderness, redness/erythema, swelling) and systemic events (fever [body temperature > 100 °F or 37.8 °C], chills,

Copyright © 2009, 2010, 2012, 2016, 2018, 2019 IQVIA. All rights reserved. The contents of this document are confidential and proprietary to IQVIA Holdings Inc. and its subsidiaries. Unauthorized use, disclosure or reproduction is strictly prohibited.

muscle pains, fatigue, headache, malaise, nausea, and vomiting). Measurements of the largest diameter for each of the injection site reactions (erythema/redness, swelling) are collected. These measurements will be used to derive severity grades based on the criteria presented in Appendix 4. Severity categories for systemic solicited AE are defined in Appendix 5.

Each solicited AE will be summarized at the following time intervals: overall during the interval Days 1-7 (captured on days 2-8 post vaccination), and daily during the interval Days 1-7 (individually, captured on days 2-8 post each vaccination). For each time interval, the count and percentage of participants will be determined for each of the following categories: participants evaluated, participants without any events, participants with any event, participants with mild events, participants with moderate events, participants with severe events, and participants with potentially life-threatening events. Participants should not be double counted; therefore, the event of greatest severity will be used for participants with more than 1 episode of the same event. Similar counts and percentages will be presented for solicited local AEs "Overall" and solicited systemic AEs "Overall". Same summaries for solicited AEs will also be presented by subgroups of serostatus and age groups (18-55, 56-69, ≥70 years; 18-64, ≥65 years).

A summary of the day of first onset of each event and the number of days participants reported experiencing each event will be presented. The number of days a participant reported experiencing an event is calculated as the total of all days the participant reported the event, regardless of whether the symptom was reported on consecutive days (e.g., a headache reported on Day 2, Day 3, and Day 5 would be included with a duration of 3 days).

A listing of all solicited AEs will be provided, with events occurring after unblinding/licensed COVID-19 vaccine administration flagged.

18.3. VITAL SIGNS

The following vital sign parameters will be collected for SARS-CoV-2-positive participants for this study as per the schedule of events (refer to protocol, Section 1.3):

- Systolic blood pressure (SBP) (mmHg)
- Diastolic blood pressure (DBP) (mmHg)
- Heart rate (beats per minute [bpm])
- Pulse rate (beats per minute [bpm])

Copyright © 2009, 2010, 2012, 2016, 2018, 2019 IQVIA. All rights reserved. The contents of this document are confidential and proprietary to IQVIA Holdings Inc. and its subsidiaries. Unauthorized use, disclosure or reproduction is strictly prohibited.

- Respiratory rate (breaths/min)
- Body temperature (C/F)
- Oxygen saturation (%)

The severity grade of abnormal Vital Signs can be referred to Appendix 3.

The following summaries will be provided by study arm based on the SAF for each vital sign parameter:

- Observed and change from study baseline at the first illness visit associated with a positive SARS-CoV-2 RT-PCR result;
- Number and percentages of participants with at least one abnormal post-baseline observed value (refer to Appendix 3);

All vital sign data will be listed, with assessments after unblinding/licensed COVID-19 vaccine administration flagged.

18.4. Physical Examination

18.4.1. General Physical Examination

Physical examinations (completed and targeted) will be conducted as per the schedule of events (refer to protocol Section 1.3). Clinically significant findings at screening will be recorded on the Medical History page of the eCRF while clinically significant changes from screening will be recorded on the AEs page of the eCRF for the post-screening visits. Hence, clinically significant findings/changes will be summarized though the Medical history summary (refer to Section 11) or AE summaries (refer to Section 18.1), as appropriate. That is, no summaries will be specifically provided for the general physical examination.

18.4.2. Chest Imaging

Listing of chest imaging results for participants with a chest imaging assessment will be provided, with assessments occurring after unblinding/licensed COVID-19 vaccine administration flagged.

Copyright © 2009, 2010, 2012, 2016, 2018, 2019 IQVIA. All rights reserved. The contents of this document are confidential and proprietary to IQVIA Holdings Inc. and its subsidiaries. Unauthorized use, disclosure or reproduction is strictly prohibited.

19. REFERENCES

Zou, G. A Modified Poisson Regression Approach to Prospective Studies with Binary Data. Am J Epidemiol 2004; 159:702–706.

Little, R. J. A. and Rubin, D. B. Statistical Analysis with Missing Data, 2nd Edition, Hoboken, NJ: John Wiley & Sons 2002; 257.

Copyright © 2009, 2010, 2012, 2016, 2018, 2019 IQVIA. All rights reserved. The contents of this document are confidential and proprietary to IQVIA Holdings Inc. and its subsidiaries. Unauthorized use, disclosure or reproduction is strictly prohibited.

APPENDIX 1. PARTIAL DATE CONVENTIONS

ALGORITHM FOR PRIOR / CONCOMITANT MEDICATIONS

START DATE	STOP DATE	ACTION
Known	Known or ongoing	If medication stop date < study intervention start date, assign as prior;<br>If medication start date < study intervention start date and (medication stop date ≥ study intervention start date or medication is ongoing at study intervention start date), assign as concomitant;<br>If study intervention start date ≤ medication start date , assign as concomitant.
	Partial	If known components of medication stop date show that medication stopped before study intervention start date, assign as prior;<br>If medication start date < study intervention start date and (known components of medication stop date show that medication stopped on or after study intervention start date), assign as concomitant;<br>If study intervention start date ≤ medication start date, assign as concomitant.
	Missing, not ongoing	If medication stop date is missing, then it can never be assigned as prior only;<br>If medication start date < study intervention start date, assign as concomitant;<br>If study intervention start date ≤ medication start date, assign as concomitant.

Copyright © 2009, 2010, 2012, 2016, 2018, 2019 IQVIA. All rights reserved. The contents of this document are confidential and proprietary to IQVIA Holdings Inc. and its subsidiaries. Unauthorized use, disclosure or reproduction is strictly prohibited.

START DATE	STOP DATE	ACTION
Partial	Known or ongoing	If medication stop date < study intervention start date, assign as prior;<br>If (known components of medication start date show that medication started before study intervention start date) and (medication stop date ≥ study intervention start date or medication is ongoing at study intervention start date), assign as concomitant;<br>If known components of medication start date show that medication started on or after study intervention start date, assign as concomitant.
	Partial	If known components of medication stop date show that medication stopped before study intervention start date, assign as prior;<br>If (known components of medication start date show that medication started before study intervention start date) and (known components of medication stop date show that medication stopped on or after study intervention start date), assign as concomitant;<br>If known components of medication start date show that medication started on or after study intervention start date, assign as concomitant.
	Missing, not ongoing	Cannot be assigned as prior only;<br>If known components of medication start date show that medication started before study intervention start date, assign as concomitant;<br>If known components of medication start date show that medication started on or after study intervention start date, assign as concomitant.
Missing	Known or ongoing	If medication stop date < study intervention start date, assign as prior;<br>If medication stop date >= study intervention start date or medication is ongoing at study intervention start date, assign as concomitant.

Copyright © 2009, 2010, 2012, 2016, 2018, 2019 IQVIA. All rights reserved. The contents of this document are confidential and proprietary to IQVIA Holdings Inc. and its subsidiaries. Unauthorized use, disclosure or reproduction is strictly prohibited.

START DATE	STOP DATE	ACTION
	Partial	If known components of medication stop date show that medication stopped before study intervention start date, assign as prior;<br>If known components of medication stop date show that medication stopped on or after study intervention start date, assign as concomitant.
	Missing, not ongoing	Assign as concomitant.

Copyright © 2009, 2010, 2012, 2016, 2018, 2019 IQVIA. All rights reserved. The contents of this document are confidential and proprietary to IQVIA Holdings Inc. and its subsidiaries. Unauthorized use, disclosure or reproduction is strictly prohibited.

APPENDIX 2. PROGRAMMING CONVENTIONS FOR OUTPUTS

DATES & TIMES

Depending on data available, dates and times will take the form yyyy-mm-dd hh:mm:ss.

SPELLING FORMAT

English US.

PAPER SIZE, ORIENTATION, AND MARGINS

The size of paper will be letter and the page orientation will be landscape. Margins will provide at least 1 inch (2.54 centimeters) of white space all around the page.

FONTS

The font type 'Courier New' will be used, with a font size of 8. The font color will be black with no bolding, underlining, italics or subscripting.

PRESENTATION OF STUDY ARMS

For outputs, study arms will be represented as follows and in the given order:

Study arm	Tables and Graphs	Listings
AZD1222	1	1
Placebo	2	2
Total [1]	3	n/a
Randomized, Not Vaccinated	n/a	3
Screen Failure	n/a	4

Copyright © 2009, 2010, 2012, 2016, 2018, 2019 IQVIA. All rights reserved. The contents of this document are confidential and proprietary to IQVIA Holdings Inc. and its subsidiaries. Unauthorized use, disclosure or reproduction is strictly prohibited.

[1] Not applicable for efficacy tables, safety tables and graphs.

PRESENTATION OF NOMINAL VISITS

For outputs, analysis visits regarding non-illness visit will be represented as follows and in that order:

Long Name (default)	Short Name
Screening	Scrn
Day 1	D1
Day 8	D8
Day 15 (Substudy only)	D15
Day 29	D29
Day 36	D36
Day 43 (Substudy only)	D43
Day 57	D57
Day 90	D90
Day 180	D180
Day 360	D360
Day 730	D730

For outputs, analysis visits regarding illness visit will be represented as follows and in that order:

Long Name (default)	Short Name
Illness Visit Day X,<br><br>X=1,3,5,8,11,14,21,28	ILDXX,<br><br>X=1,3,5,8,11,14,21,28

Copyright © 2009, 2010, 2012, 2016, 2018, 2019 IQVIA. All rights reserved. The contents of this document are confidential and proprietary to IQVIA Holdings Inc. and its subsidiaries. Unauthorized use, disclosure or reproduction is strictly prohibited.

DESCRIPTIVE STATISTICS

If the original data has N decimal places, then the summary statistics will have the following decimal places:

- Minimum and maximum: N;
- Mean, median, Q1, Q3, lower and upper bounds of two-sided 95% CI: N + 1;
- SD and SE: N + 2

PERCENTAGES

Percentages will be reported to one decimal place. Rounding will be applied, except for percentages < 0.1 but > 0.0 which will be presented as '< 0.1' and percentages < 100.0 but >99.9 which will be presented as '>99.9'.

Where counts are zero, no percentages will appear in the output.

P-VALUES

p-values will be reported to three decimal places. Rounding will be applied, except for the p-values < 0.001 which will be presented as '< 0.001' and p-values < 1.000 but > 0.999 which will be presented as '> 0.999'.

LISTINGS

All listings will be ordered by the following (unless otherwise indicated in the output template):

- Randomized study arm (or study intervention received if it's a safety output);
- Participant ID;
- Parameter, when applicable;
- Date/Time, when applicable.
- Timepoint, when applicable

Copyright © 2009, 2010, 2012, 2016, 2018, 2019 IQVIA. All rights reserved. The contents of this document are confidential and proprietary to IQVIA Holdings Inc. and its subsidiaries. Unauthorized use, disclosure or reproduction is strictly prohibited.

APPENDIX 3. CLINICAL ABNORMALITIES

Clinical Abnormalities: Vital Signs

Vital Signs [a]	Vital Signs Grade			
	Mild (Grade 1)	Moderate (Grade 2)	Severe (Grade 3)	Potentially Life Threatening (Grade 4)
Fever (°C) [b]<br>(°F) [b]	37.9-38.4<br>100.1-101.1	38.5-38.9<br>101.2-102.0	39.0-40<br>102.1-104	> 40<br>> 104
Tachycardia (beats/minute)	101-115	116- 130	> 130	ER visit or hospitalization for arrhythmia
Bradycardia (beats/minute) [c]	50-54	45-49	< 45	ER visit or hospitalization for arrhythmia
Hypertension; systolic (mm Hg)	141-150	151-155	> 155	ER visit or hospitalization for malignant hypertension
Hypertension; diastolic (mm Hg)	91-95	96-100	> 100	ER visit or hospitalization for malignant hypertension
Hypotension; systolic (mm Hg)	85-89	80-84	< 80	ER visit or hospitalization for hypotensive shock
Respiratory rate (breaths/minute)	17-20	21-25	> 25	Intubation

Note: Record vital signs as adverse events only if clinically relevant and changed from baseline.

[a] Participant should be at rest for vital signs measurements.

[b] No recent hot or cold beverages or smoking.

[c] Use clinical judgment when characterizing bradycardia among some healthy participant populations, for example, conditioned athletes.

ER = emergency room; Hg = mercury.

Copyright © 2009, 2010, 2012, 2016, 2018, 2019 IQVIA. All rights reserved. The contents of this document are confidential and proprietary to IQVIA Holdings Inc. and its subsidiaries. Unauthorized use, disclosure or reproduction is strictly prohibited.

APPENDIX 4. CLINICAL ABNORMALITIES

Local Reactions to Injectable Product

Local Reaction to Injectable Product	Reaction Grade			
	**Mild (Grade 1)**	**Moderate (Grade 2)**	**Severe (Grade 3)**	**Life Threatening (Grade 4)**
Pain	Does not interfere with activity	Repeated use of non-narcotic pain reliever > 24 hours or interferes with activity	Any use of narcotic pain reliever or prevents daily activity	ER visit or hospitalization
Tenderness	Mild discomfort to touch	Discomfort with movement	Significant discomfort at rest	ER visit or hospitalization
Erythema/redness [a, b]	1-2 inches (2.5–5 cm)	> 2-4 inches (5.1–10 cm)	> 4 inches (> 10 cm)	Necrosis or exfoliative dermatitis
Induration/swelling [a, b]	1-2 inches (2.5–5 cm)	>2-4 inches (5.1–10 cm)	> 4 inches (> 10 cm)	Necrosis

[a] In addition to grading the measured local reaction at the greatest single diameter, the measurement should be recorded as a continuous variable. Reactions < 0.25 inches (< 0.6 centimeters) in diameter will not be recorded.

[b] Grade 4 erythema or induration is determined by study site with participant input rather than being recorded directly in Solicited AE e-Diary.

ER = emergency room.

Copyright © 2009, 2010, 2012, 2016, 2018, 2019 IQVIA. All rights reserved. The contents of this document are confidential and proprietary to IQVIA Holdings Inc. and its subsidiaries. Unauthorized use, disclosure or reproduction is strictly prohibited.

APPENDIX 5. CLINICAL ABNORMALITIES: SYSTEMIC

Systemic (General or Illness)

Systemic (General)	Systemic Grade			
	**Mild (Grade 1)**	**Moderate (Grade 2)**	**Severe (Grade 3)**	**Potentially Life Threatening (Grade 4)**
Nausea/vomiting	No interference with activity or 1-2 episodes/24 hrs	Some interference with activity or > 2 episodes/24 hrs	Prevents daily activity, required outpatient IV hydration	ER visit or hospitalization for hypotensive shock
Chills	No interference with activity	Some interference with activity	Significant; prevents daily activity	ER visit or hospitalization
Headache	No interference with activity	Repeated use of non-narcotic pain reliever > 24 hrs or some interference with activity	Significant; any use of narcotic pain reliever or prevents daily activity	ER visit or hospitalization
Fatigue	No interference with activity	Some interference with activity	Significant; prevents daily activity	ER visit or hospitalization
Myalgia	No interference with activity	Some interference with activity	Significant; prevents daily activity	ER visit or hospitalization
**Systemic Illness**				
Illness or clinical adverse event (as defined according to applicable regulations)	No interference with activity	Some interference with activity not requiring intervention	Prevents daily activity and required medical intervention	ER visit or hospitalization

ER = emergency room; hrs = hours; IV = intravenous.

Copyright © 2009, 2010, 2012, 2016, 2018, 2019 IQVIA. All rights reserved. The contents of this document are confidential and proprietary to IQVIA Holdings Inc. and its subsidiaries. Unauthorized use, disclosure or reproduction is strictly prohibited.

APPENDIX 6. COVID-19 SYMPTOMS PER CDC CRITERIA

COVID-19 Symptoms Per CDC Criteria

Participant must present with at least one of the following symptoms
**Symptom**
Fever
Shortness of breath
Difficulty breathing
Chills
Cough
Fatigue
Muscle aches
Body aches
Headache
New loss of taste
New loss of smell
Sore throat
Congestion
Runny nose
Nausea
Vomiting
Diarrhea

CDC, 2020.

Copyright © 2009, 2010, 2012, 2016, 2018, 2019 IQVIA. All rights reserved. The contents of this document are confidential and proprietary to IQVIA Holdings Inc. and its subsidiaries. Unauthorized use, disclosure or reproduction is strictly prohibited.

APPENDIX 7. ADVERSE EVENTS OF SPECIAL INTEREST

AZD1222 AESIs are based on Brighton Collaboration case definitions (SPEAC 2020), clinical experience, and scientific interest. There is no current evidence to suggest that AZD1222 is associated with these AESIs.

AESI	Medical Concept
Neurologic	Generalized convulsion: Seizures are episodes of neuronal hyperactivity most commonly resulting in sudden, involuntary muscular contractions. They may also manifest as sensory disturbances, autonomic dysfunction and behavioral abnormalities, and impairment or loss of consciousness.
	Guillain-Barré syndrome: GBS is a peripheral nerve demyelinating disease, which can present as temporary ascending paralysis.
	Acute disseminated encephalomyelitis: ADEM is defined as a uniphasic syndrome of brain inflammation and demyelination occurring in temporal association with an antecedent immunologic challenge, such as infection or an immunization. ADEM most commonly occurs in the pediatric population.
	Other neurologic events: These events would include new onset event (acute or subacute) motor and sensory disturbances (eg, weakness, numbness, paresthesias, hypoesthesia, hyperesthesia, dysesthesias), bowel/bladder dysfunction, gait impairment, or visual disturbance, or any event of myelitis, encephalomyelitis, myelitis transverse, or other sudden neurological deficit.
Vascular	Thrombotic, thromboembolic, and neurovascular events: These are events that can manifest as transient or permanent vision problems, dizziness, trouble understanding, facial droop, slurred speech, unilateral weakness, deep vein thrombosis with swollen, warm or painful leg, pulmonary embolism with shortness of breath, chest pain or irregular heart rate
Hematologic	Thrombocytopenia: Thrombocytopenia is a disorder in which there is an abnormally low platelet count; a normal platelet count ranges from 150 000 to 450 000 platelets per µL.
Immunologic	Vasculitides: Vasculitides are a group of related disorders characterized by inflammation of blood vessels (vasculitis) leading to tissue or end-organ injury.
	Anaphylaxis: Anaphylaxis an acute hypersensitivity reaction with multi-organ-system involvement that can present as, or rapidly progress to, a severe life-threatening reaction requiring immediate medical attention.

Copyright © 2009, 2010, 2012, 2016, 2018, 2019 IQVIA. All rights reserved. The contents of this document are confidential and proprietary to IQVIA Holdings Inc. and its subsidiaries. Unauthorized use, disclosure or reproduction is strictly prohibited.

AESI	Medical Concept
Immunologic (Continued)	Vaccine-associated enhanced respiratory disease: The pathogenicity of VAERD has been linked to a vaccine immune response characterized by induction of non-neutralizing antibodies, and a T-cell response of the Th2 type with hypereosinophilia (Lambert et al 2020). VAERD may manifest as a severe form of respiratory disease with prolonged fever, and diverse clinical manifestations of disease severity and pathological changes marked by increased areas of lung consolidation, broncho-interstitial pneumonia, and necrotizing bronchiolitis (Rajão et al 2016).
	Potential immune-mediated conditions: These conditions are a group of autoimmune inflammatory disorders characterized by an alteration in cellular homeostasis, which may or may not have an autoimmune aetiology. A list of events is provided in **Error! Reference source not found.** of the clinical study protocol.

ADEM = acute disseminated encephalomyelitis; GBS = Guillain-Barré syndrome; VAERD = vaccine-associated enhanced respiratory disease.

Copyright © 2009, 2010, 2012, 2016, 2018, 2019 IQVIA. All rights reserved. The contents of this document are confidential and proprietary to IQVIA Holdings Inc. and its subsidiaries. Unauthorized use, disclosure or reproduction is strictly prohibited.

SIGNATURE PAGE

This is a representation of an electronic record that was signed electronically and this page is the manifestation of the electronic signature

**Document Name:** d8110c00001-sap-ed-3		
**Document Title:**	Statistical Analysis Plan Edition 3	
**Document ID:**	Doc ID-004393690	
**Version Label:**	3.0 CURRENT LATEST APPROVED	
**Server Date (dd-MMM-yyyy HH:mm 'UTC'Z)**	**Signed by**	**Meaning of Signature**
01-Mar-2021 01:53 UTC	[redacted]	Content Approval
01-Mar-2021 00:25 UTC	[redacted]	Content Approval
01-Mar-2021 15:49 UTC	[redacted]	Author Approval

Notes: (1) Document details as stored in ANGEL, an AstraZeneca document management system.
